# Loss of *ACR* and Retention of Its Ancient Paralog *TMPRSS12* in the Evolution of Teleost Fertilization

**DOI:** 10.3390/biology15212040

**Published:** 2026-11-08

**Authors:** Tengfei Wu, Liangkun Wang, Ping Li, Yongming Wang, Yanye Wu, Shihan Liu, Zuogang Peng, Feng Shao

**Affiliations:** 1 Key Laboratory of Freshwater Fish Reproduction and Development (Ministry of Education), School of Life Sciences, Southwest University, No. 2 Tiansheng Road, Chongqing 400715, China; feiyuhanqing@outlook.com (T.W.); swuliangkun@email.swu.edu.cn (L.W.); wym8188@126.com (Y.W.); yiye7375@gmail.com (Y.W.); lsh666888@email.swu.edu.cn (S.L.); 2 College of Fisheries, Southwest University, Chongqing 402460, China; lp2006@swu.edu.cn; 3 Fishes Conservation and Utilization in the Upper Reaches of the Yangtze River Key Laboratory of Sichuan Province, College of Life Science, Neijiang Normal University, Neijiang 641100, China

**Keywords:** *ACR*, *TMPRSS12*, gene duplication, 2R whole-genome duplication, phylogenetics, vertebrate evolution

## Abstract

Acrosin (*ACR*) and transmembrane protease serine 12 (*TMPRSS12*) are sperm-related S1/trypsin-like serine proteases involved in mammalian male fertility, but their evolutionary relationship in vertebrates remains unclear. Here, we combined phylogenetic, synteny, selection-pressure, and transcriptomic analyses to investigate their evolutionary origin and divergence. OHNOLOGS v2 identified *ACR*–*TMPRSS12* as a candidate ohnolog pair with strong outgroup support, and OrthoFinder placed their duplication in the ancestor of jawed vertebrates. Phylogenetic analyses recovered *ACR* and *TMPRSS12* as reciprocally monophyletic sister clades, consistent with an ancient duplication that may be associated with the two rounds of whole-genome duplication (2R-WGD). Their subsequent evolutionary trajectories differed markedly. *ACR* was retained in chondrichthyans, sarcopterygians, tetrapods, and acipenseriforms, whereas its widespread loss in other actinopterygians largely coincided with acrosome loss, although bichirs retained the acrosome but lacked *ACR*. *TMPRSS12* was widely retained across jawed vertebrates but showed relaxed selection in Poeciliidae and independent losses in *Sebastes* and *Takifugu*. Both genes exhibited testis-biased transcript expression, and their proteins retained a relatively conserved trypsin-like catalytic domain. These findings reveal contrasting patterns of gene retention between *ACR* and *TMPRSS12* and lineage-specific variation in selective constraints on *TMPRSS12*, consistent with their divergence following an ancient duplication event.

## Simple Summary

Fertilization in vertebrates has changed greatly during evolution, but how reproduction-related genes have evolved along with these changes is still not fully understood. Here, we studied two related genes involved in sperm function, called the acrosin gene and the transmembrane protease serine 12 gene. By comparing genomic, evolutionary, and expression data from many vertebrate species, we found that these two genes originated from an ancient gene duplication event. The acrosin gene was mainly retained in animals whose sperm contain a cap-like structure called the acrosome, which helps sperm penetrate the egg, while many fish species lacking this structure have also lost the acrosin gene. In contrast, the transmembrane protease serine 12 gene was retained in most jawed vertebrates, although it evolved under weaker selective pressure in some fish groups and was independently lost in two fish lineages. In public databases, the fish version of this gene has long been labeled as “acrosin-like”. Our results clarify the relationship between these two genes, correct this misannotation, and show how duplicated genes can follow different evolutionary paths during changes in fertilization systems.

## 1. Introduction

Sperm–egg recognition and fusion are processes that have been extensively studied in reproductive and evolutionary biology [1]. The genes that are involved in mediating gamete interactions frequently experience selective pressures that are relatively complex and evolve rapidly across animal lineages [2]. In invertebrates, gamete recognition proteins such as molluscan lysin and sea urchin bindin exhibit amino acid substitution rates that considerably exceed the genomic average, driven by positive selection in numerous cases [3]. A similar pattern occurs in vertebrates, with approximately half of the 69 fertilization-related genes across 19 species showing evidence of positive selection, along with widespread gene duplication and pseudogenization [4]; the zona pellucida adhesion protein zonadhesin has undergone extensive lineage-specific divergence in placental mammals [5]. These findings suggest that the evolution of fertilization genes is jointly influenced by reproductive mode, sperm–egg binding mechanisms, and changes in the fertilization environment.

The acrosome is a key cellular structure involved in sperm–egg recognition and fusion. This cap-shaped membranous organelle, originating from the Golgi apparatus, covers the anterior end of the sperm head and contains various hydrolytic enzymes [6,7]. It is classified as a specialized lysosome, secretory granule, and lysosome-related organelle, which likely originates from an autophagolysosome [8]. The co-evolution of sperm and egg makes acrosome morphology highly differentiated among animal groups [6]. Among vertebrates, chondrichthyans, sarcopterygians, and tetrapods have well-developed acrosomes, whereas most teleosts lack this structure, and their sperm enter the egg cytoplasm through the micropyle [9,10]. Bichirs and acipenseriforms retain the acrosome, and certain teleosts with internal fertilization, such as the western mosquitofish, have acrosome-like structures, whereas other teleosts form acrosomal vesicles during spermatogenesis [11,12].

Acrosin (*ACR*) encodes acrosin, one of the most abundant serine proteases in the sperm acrosome and belongs to the S1/trypsin superfamily [13,14]. In tetrapods, acrosin is released during the acrosome reaction and hydrolyzes the zona pellucida or egg envelope. Functional studies in hamsters and genetic evidence from humans indicate that acrosin plays an essential role in efficient sperm penetration through the zona pellucida in these species [15,16]. Owing to the extensive loss of the acrosome in teleosts, the evolutionary trajectory of *ACR* in actinopterygians may differ from that in tetrapods. However, its broad distribution and evolutionary history across vertebrates remain unclear.

Transmembrane protease serine 12 (*TMPRSS12*) is a recently identified serine protease involved in male reproduction. *Tmprss12* knockout mice are completely infertile with defects in sperm motility, uterotubal junction migration, and spermatogenesis [17,18]. The human *TMPRSS12* transgene did not rescue the infertility phenotype of mouse mutants, indicating functional divergence of this gene among mammals [19]. *TMPRSS12*, similar to *ACR*, belongs to the S1/trypsin-like serine protease family and is associated with the sperm acrosomal region; however, it differs from *ACR* in its membrane-anchored topology [17]. In NCBI and Ensembl, fish *tmprss12* has been annotated as “acrosin-like” or “acrosin” for many years. This apparent inconsistency suggests that an evolutionary relationship exists between *ACR* and *TMPRSS12* that has not been fully characterized; however, the nature of this relationship and whether its origin can be linked to an ancient duplication event remains to be investigated systematically.

Gene duplication provides raw genetic material for neofunctionalization and subfunctionalization, and is a major driver of functional innovation [20]. Early vertebrate evolution was characterized by two rounds of whole-genome duplication (2R-WGD) [21]. 2R-WGD produces large numbers of ohnologs; approximately 25% of extant vertebrate protein-coding genes descend from these events [22], and a subset of these ohnologs has been retained over the long term through neofunctionalization or subfunctionalization [23]. The evolutionary relationship between *ACR* and *TMPRSS12* may reflect duplication during vertebrate 2R-WGD, but other possibilities, including an earlier duplication or a local duplication followed by chromosomal rearrangement, cannot be excluded. We therefore examined their phylogenetic relationships, ohnolog classification, and conserved synteny to assess whether the available evidence is consistent with a 2R-WGD origin. The retention, loss, subfunctionalization, or neofunctionalization of duplicated genes depends on selection pressure, dosage balance, and functional context [20,24]. In teleosts, the loss of the acrosome and the use of micropylar fertilization alter the fertilization process, which may reduce the selective pressure to maintain the traditional zona-penetrating function of *ACR* [9,25]. In contrast, internal fertilization occurs repeatedly across teleost lineages. These divergent evolutionary backgrounds may impose distinct selective pressures on *ACR* and *TMPRSS12*; however, the specific effects remain unknown.

In this study, we integrated large-scale phylogenetic reconstruction, comparative synteny, ohnolog evidence, selection analyses, and transcriptomic data to elucidate the evolutionary relationship between *ACR* and *TMPRSS12* across vertebrates. Specifically, we investigated whether the two genes originated from an ancient duplication associated with the vertebrate 2R-WGD, the variation in their patterns of retention and loss among major vertebrate lineages, and whether these differences were associated with shifts in reproductive traits and selective constraints.

## 2. Materials and Methods

### 2.1. Ortholog Retrieval and Genome-Wide Homology Inference

*ACR* orthologs were retrieved from NCBI Orthologs using human *ACR* (GeneID: 49) as the query, whereas *TMPRSS12* orthologs were retrieved using human *TMPRSS12* (GeneID: 283471) for tetrapods and zebrafish *tmprss12* (GeneID: 110438336) for actinopterygians. For chondrichthyans, sarcopterygians, and non-teleost actinopterygians lacking annotated orthologs in NCBI, tBLASTn searches (BLAST+ v2.9.0) [26] were performed against whole-genome assemblies with an E-value threshold of ≤1 × 10^−5^. The corresponding protein sequences were human *ACR* (UniProt: P10323), human *TMPRSS12* (UniProt: Q86WS5), and zebrafish Tmprss12 (UniProt: A0AB32TG56). The E-value threshold of 1 × 10^−5^ was used for initial candidate identification, and hits were retained only after verification of their genomic location and flanking-gene synteny, using the anchor genes and criteria described in Section 2.2. This procedure resulted in 301 *ACR* and 612 *TMPRSS12* sequences from 638 species. Several species retained multiple copies because of lineage-specific whole-genome duplication (WGD) or local duplication. To reduce the risk of missing divergent or poorly annotated homologs, we used lineage-appropriate query sequences and checked candidate genes against conserved flanking-gene synteny. Highly divergent homologs may nevertheless have escaped detection. We, therefore, also performed whole-genome searches using miniprot and tBLASTn for the 28 species included in the trait matrix, as described in Section 2.2. Whole-proteome orthogroup inference was performed using OrthoFinder v2.5.5 [27] across 30 representative species with partial overlap with the trait-matrix species set (Table S1). Orthogroups (OG0000013) containing *ACR* and *TMPRSS12* were extracted for downstream analysis (Table S2).

### 2.2. Genome-Level Presence/Absence Determination of ACR and TMPRSS12

To determine the presence/absence of *ACR* and *TMPRSS12* independently of database annotations, we performed genome-level miniprot v0.13 and tBLASTn searches across the 28 representative species, including *Homo sapiens* as a query-derived positive control. We searched the whole genome of each species using miniprot and tBLASTn (BLAST+ v2.9.0), with an E-value threshold of 1 × 10^−5^ for tBLASTn candidate retrieval; the anchor-defined interval was used only to determine genomic location and support orthology assignment. The anchor genes were human *DIP2B* (UniProt Q9P265) and human *SCN8A* (UniProt Q9UQD0) for *TMPRSS12*, and human *SHANK3* (UniProt Q9BYB0) and human *RABL2B* (UniProt Q9UNT1) for *ACR*, whose orthologs in each species were located by miniprot using these sequences (Table S3A). Query proteins were selected based on the principle of lineage proximity; for actinopterygians, zebrafish Tmprss12 (UniProt A0AB32TG56) and *Acipenser ruthenus* Acr (RefSeq XP_033888599.2) were used, and for other species, human *TMPRSS12* and human *ACR* were used. For miniprot, a qualifying hit was operationally defined as an alignment spanning at least three CDS blocks within the anchor-defined interval. This cutoff was used to require support across multiple coding segments rather than an isolated or short local match. Presence was assigned according to the tiered decision rule described in the notes to Table S3B,C. A gene was scored as lost when neither miniprot nor tBLASTn recovered an in-interval hit and no syntenic candidate was identified. Out-of-interval hits were evaluated by BLASTp best-hit and phylogenetic placement. Hits identified as other S1 serine proteases were excluded (Table S3D), whereas candidates confirmed as the bona fide *tmprss12*/*acr* ortholog were retained as exception Present calls and are marked with a dagger (†) in Table S3B,C. We also recorded whether the anchors occurred on the same scaffold and the number of intervening gaps (Table S3A). When anchors were on different scaffolds, presence was assessed using whole-genome searches and flanking-gene synteny (Table S3B,C). Loss was assigned only when the target interval was considered reliably assessable. When assembly fragmentation prevented reliable assessment of the interval, failure to recover a candidate was recorded as unresolved rather than as a definitive loss.

### 2.3. Ancestral State Reconstruction

Maximum likelihood ancestral state reconstruction was performed for four discrete traits on a time-calibrated species tree topology obtained from TimeTree [28]. The tree included 25 representative vertebrates and three outgroup taxa: *Branchiostoma floridae*, *Ciona intestinalis*, and *Petromyzon marinus* (Table S4). The three outgroup taxa were pruned from the tree before ancestral-state reconstruction. *ACR* orthologs could not be assigned confidently in these outgroups. The outgroups were excluded so that all four traits could be reconstructed using the same set of 25 jawed-vertebrate species. Ancestral states were reconstructed using the ace() function in the R package ape v5.8 [29]. For each trait, we fitted three transition models: equal rates (ER), in which all transitions among character states occur at the same rate [30]; symmetrical rates (SYM); and all rates different (ARD). Model fit was compared using the Akaike information criterion (AIC), with log-likelihood (lnL), number of parameters (K), AIC, and ΔAIC reported in Table S5A. For models with multiple free transition rates, we used multistart optimization with 50, 60, and 200 random starts for binary ARD, three-state SYM, and three-state ARD, respectively. Starting rates were sampled log-uniformly within ±3 orders of magnitude of the ER-fitted rate, with one additional start initialized at the ER rate for all parameters. The highest-likelihood solution was retained. The same procedure was used for model refits in the leave-one-out sensitivity analysis. ER and SYM are equivalent for the two binary traits, *acr* and acrosome. Ancestral-state probabilities at focal nodes under each model are reported in Table S5B. Ancestral-state probabilities at all internal nodes (reported by the ace() function as scaled ancestral-state likelihoods in lik.anc) were retained for visualization. To assess the sensitivity of ancestral-state estimates to individual species observations, we performed a leave-one-out sensitivity analysis while retaining the complete 25-species phylogeny. In each replicate, the character state of one species was treated as unknown (NA), and model parameters and ancestral-state probabilities were re-estimated using the transition model selected from the full dataset (ER for *acr*, acrosome, and micropyle; SYM for fertilization). This procedure preserves the identity of the focal ancestral nodes across all replicates. For each focal node, the maximum-likelihood state inferred from the complete dataset was designated as the reference state, and its probability was recorded across the 25 leave-one-out replicates and summarized by the mean, standard deviation, and range (Table S5C).

A formal test of correlated evolution (e.g., Pagel’s discrete test or an equivalent BayesTraits analysis) was not performed because *ACR* presence and sperm-acrosome presence showed near-complete correspondence among the sampled jawed vertebrates; bichirs were the only lineage that retained the acrosome but lacked *ACR*. The limited number of discordant observations provided insufficient replicated evolutionary changes to reliably estimate the multiple transition-rate parameters required by a correlated-evolution model. We therefore treated the observed correspondence as descriptive rather than as evidence of a causal relationship and assessed the robustness of the ancestral-state reconstruction through model comparison and leave-one-out sensitivity analyses.

### 2.4. Ohnolog Validation

The homologous relationship between *ACR* and *TMPRSS12* was validated using OHNOLOGS v2, MCScanX synteny and gene family phylogenetic analyses. The OHNOLOGS v2 database [22] was queried using human *TMPRSS12*, and all candidate ohnologs at the relaxed level (q < 0.05) were retrieved to construct a candidate ohnolog network. Synteny analysis was performed based on the expectation that ohnologs generated by vertebrate 2R-WGD might be retained in duplicated chromosomal regions with conserved macrosynteny, referred to as paralogons [31]. MCScanX [32] was applied to human chr12 (which harbors *TMPRSS12*) versus chr22 (which harbors *ACR*) at two stringency levels: standard (-s 5, -m 20, e ≤ 1 × 10^−5^) and relaxed (-s 3, -m 50, e ≤ 1 × 10^−3^). To determine the phylogenetic positions of *ACR* and *TMPRSS12* within the serine protease family, we sampled *TMPRSS12* and seven related ohnologs (*TMPRSS6*, *TMPRSS7*, *TMPRSS9*, *ST14*, *CORIN*, *ACR*, and *PRSS56*) in representative vertebrates. These included lampreys, chondrichthyans, sarcopterygians, tetrapods, non-teleost actinopterygians, and teleosts, yielding 114 sequences (Table S6). Alignment was performed using MAFFT v7.505 [33], and trimming was performed using trimAl v1.4 [34]. Maximum likelihood trees were built using IQ-TREE v2.2.3 [35], and the best-fit amino acid substitution model was selected using ModelFinder [36] under the Bayesian information criterion (BIC). Branch support was assessed using 1000 ultrafast bootstrap (UFBoot) replicates and 1000 Shimodaira–Hasegawa approximate likelihood ratio test (SH-aLRT) replicates. In addition, all relaxed-level OHNOLOGS v2 pairs between chr12 and chr22 were retrieved to evaluate whether the two chromosomes carried multiple ohnolog pairs that spanned diverse gene families, which is a hallmark of a genuine 2R paralogon.

A larger phylogeny was reconstructed using 966 sequences (Table S7), including 913 *ACR* and *TMPRSS12* sequences, and 53 outgroup sequences from OG0000013 (*Branchiostoma lanceolatum*, *C. intestinalis*, and *P. marinus*). Sequences were aligned with MAFFT, trimmed with trimAl (-gappyout), and analyzed with IQ-TREE under the best-fit model JTT+R9 and the same branch-support settings as described above. The tree was rooted using the 53 outgroup sequences, and the monophyly of the *ACR* and *TMPRSS12* clades was assessed.

### 2.5. Selection Pressure Analysis

To test whether *tmprss12* in Poeciliidae has undergone relaxed selection, a phylogeny-based selection pressure analysis was performed on 146 actinopterygian *TMPRSS12* sequences, a filtered subset of the actinopterygian sequences listed in Table S7. Protein sequences were aligned with MAFFT v7.505 (--auto) and back-translated to a codon alignment with pal2nal v14; the codon alignment was then trimmed with trimAl v1.4 (-gt 0.3); 303 codon sites were retained. A maximum-likelihood gene tree was constructed from the codon alignment using IQ-TREE v3.1.2 (-st CODON), and the best-fit codon substitution model (MG+F3X4+R6) was selected using ModelFinder, according to BIC. The most recent common ancestor (MRCA) node, which encompassed nine poeciliid species from four genera (*Gambusia*, *Poecilia*, *Poeciliopsis*, and *Xiphophorus*), was used as the anchor point, and the stem branch leading to this MRCA and all branches descending from this node (internal and terminal branches) were jointly defined as the foreground (test) branch set, while the remaining branches were treated as the background (reference).

The selection pressure analyses were performed using HyPhy v2.5.100. Three complementary codon-based selection analyses were used to characterize selection on *TMPRSS12*, including lineage-specific patterns in Poeciliidae. (1) RELAX was used to compare the strength of selection between the poeciliid foreground branch set (the stem branch leading to the poeciliid MRCA plus all descendant branches) and the background branches, and to test whether Poeciliidae experienced relaxed (K < 1) or intensified (K > 1) natural selection. (2) BUSTED was applied to all branches to test for gene-wide evidence of episodic diversifying selection somewhere in the phylogeny. (3) aBSREL was applied to all 286 branches of the tree to test for positive selection on each branch and determine whether any individual branch within Poeciliidae showed evidence of positive selection. *p*-values were corrected using Holm’s test. Three additional site-level tests were performed across the entire ray-finned fish tree, including all the branches. (4) MEME was used to identify sites under episodic diversifying selection, with *p*-values corrected using the Benjamini–Hochberg method (false discovery rate, FDR < 0.05). (5) FEL was used to estimate dN/dS at each site using maximum likelihood, and likelihood ratio tests were used to classify sites as being under purifying, neutral, or positive selection. (6) FUBAR used a Bayesian approach to estimate the posterior probabilities of purifying and positive selection at each site, with posterior probabilities > 0.9 considered significant. The sequence filtering criteria and alignment trimming parameters are listed in Table S8.

To assess the sensitivity of the RELAX results to alignment trimming, taxon sampling, and foreground definition, we repeated RELAX under alternative settings. For alignment trimming, we trimmed the alignment with -gt 0.1 or -gt 0.5 and re-estimated the gene tree from each trimmed alignment. For taxon sampling, we removed one species at a time (*Gambusia affinis*, *Fundulus heteroclitus*, *Dicentrarchus labrax*, or *Aulonocara stuartgranti*) and re-estimated the gene tree from each resulting alignment. For foreground definition, we redefined the foreground as (i) the stem branch alone, (ii) the crown branches alone (all branches descending from the poeciliid MRCA, excluding the stem), or (iii) the nine terminal poeciliid branches. The poeciliid clade remained monophyletic in every re-estimated tree (Table S9).

### 2.6. Verification of Lineage-Specific TMPRSS12 Loss

To confirm the absences determined based on the above genome searches at the transcription level, we analyzed testis RNA-seq data from four teleost species (Table S10), which included western mosquitofish (*G. affinis*), turquoise killifish (*Nothobranchius furzeri*), tiger puffer (*Takifugu rubripes*), and black rockfish (*Sebastes schlegelii*); the first two were control species with *TMPRSS12* retained, and the latter two were suspected loss species. Reads were filtered for quality control with fastp v0.20.0, then aligned to the reference genome using HISAT2 v2.1.0, and alignment results were sorted and indexed using SAMtools v1.9. Subsequently, deepTools bamCoverage v3.3.1 was used to calculate normalized coverage (log_2_(1 + counts per million [CPM])) with 10 bp windows and output in bigwig format for visualization, and StringTie v2.0 was used to assemble transcripts in reference genome-guided mode, which was limited to the *dip2ba*–*scn8a* locus.

Based on the above RNA-seq data, we further examined transcription from the expected *tmprss12* locus in two species in which *tmprss12* was not detected by the genome-level searches: tiger pufferfish and black rockfish. The genomic region spanning *dip2ba* to *scn8a* was first examined for assembly gaps, defined as continuous stretches of N bases. StringTie transcripts detected in only one sample and showing no significant similarity to *tmprss12* (tBLASTn E-value > 0.05) were considered low-confidence. To assess whether they might represent weak residual *tmprss12* transcripts, we re-screened them against the killifish Tmprss12 protein (RefSeq: XP_015823280.3) using a permissive E-value cutoff of 10.

### 2.7. Tissue Expression and Protein Domain Analysis

Multispecies tissue expression data were obtained from a cross-vertebrate multi-tissue transcriptome compendium [37], in which transcripts per million (TPM) values were log_2_-transformed with a +1 pseudocount and corrected for hidden technical biases using surrogate variable analysis (SVA) by the source authors. The resulting SVA-corrected log-transformed transcripts per million (SVA-log-TPM) values for *ACR* across 16 tetrapods and *TMPRSS12* across 21 vertebrates were extracted from the brain, heart, kidney, liver, ovary, and testis.

To further examine the tissue expression pattern of *tmprss12*, adult male western mosquitofish were collected from Chongde Lake at Southwest University (29.82° N, 106.42° E). Fish were maintained in a recirculating water system for 14 days at 26 ± 1 °C under a 14 h light:10 h dark cycle before sampling. All animal procedures were approved by the Animal Ethics Committee of the Southwest University (approval no. SWU_LAC2024030066). The collected fish were sedated using MS-222 (Macklin, Shanghai, China) at a concentration of 200 mg/L for 2 min and subsequently euthanized by pithing. Eight tissue samples, including the brain, heart, liver, spleen, kidney, intestine, testis, and muscle, were collected. Total RNA was extracted using RNAiso Plus (Takara, Dalian, China), and 1000 ng of total RNA was reverse-transcribed into cDNA using ToloScript All-in-one RT EasyMix for quantitative PCR (qPCR) (TOLOBIO, Shanghai, China). Reverse transcription quantitative PCR (RT-qPCR) was performed on a QuantStudio 1 system (Thermo Fisher, Waltham, MA, USA) using NovoStart SYBR qPCR SuperMix Plus (Novoprotein, Suzhou, China), with *β-actin* as the reference gene. The forward and reverse primers for *tmprss12* were 5′-GGAGTCGACACTTGTCAGGG-3′ and 5′-TCCACAATTCTCCCCGAAGC-3′, respectively, and those for *β-actin* were 5′-TGAAATTGCAGTTCTCGTTG-3′ and 5′-CGACCAACAATGGAGGGGAA-3′, respectively. Three adult male fish served as biological replicates for each tissue sample (*n* = 3). Relative expression was calculated using the 2^−ΔΔCt^ method, normalized to *β-actin* and calibrated to muscle. Primer sequences, amplicon sizes, melting temperatures, and amplification efficiencies of the RT-qPCR assays are summarized in Table S11. Separate primer pairs for *tmprss12* and *β-actin* (301 bp and 337 bp amplicons, respectively; Table S11) were used for RT-PCR, and the amplification products were resolved by agarose gel electrophoresis to confirm their expected sizes.

Protein domains were annotated for 33 sequences (10 *ACR* and 23 *TMPRSS12* orthologs; Table S12) using InterProScan v5.70 [38], and domain assignments were cross-validated against Pfam (PF00089) [39], the NCBI Conserved Domain Database (cd00190) [40], and SMART (SM00020) [41]. Signal peptides were predicted using DeepTMHMM [42]. The catalytic triad residues were identified by aligning the sequences with human *TMPRSS12* using MAFFT: His122, Asp171, and Ser268.

## 3. Results

### 3.1. ACR and TMPRSS12 Show Contrasting Retention Patterns Across Vertebrates

**Figure 1:**
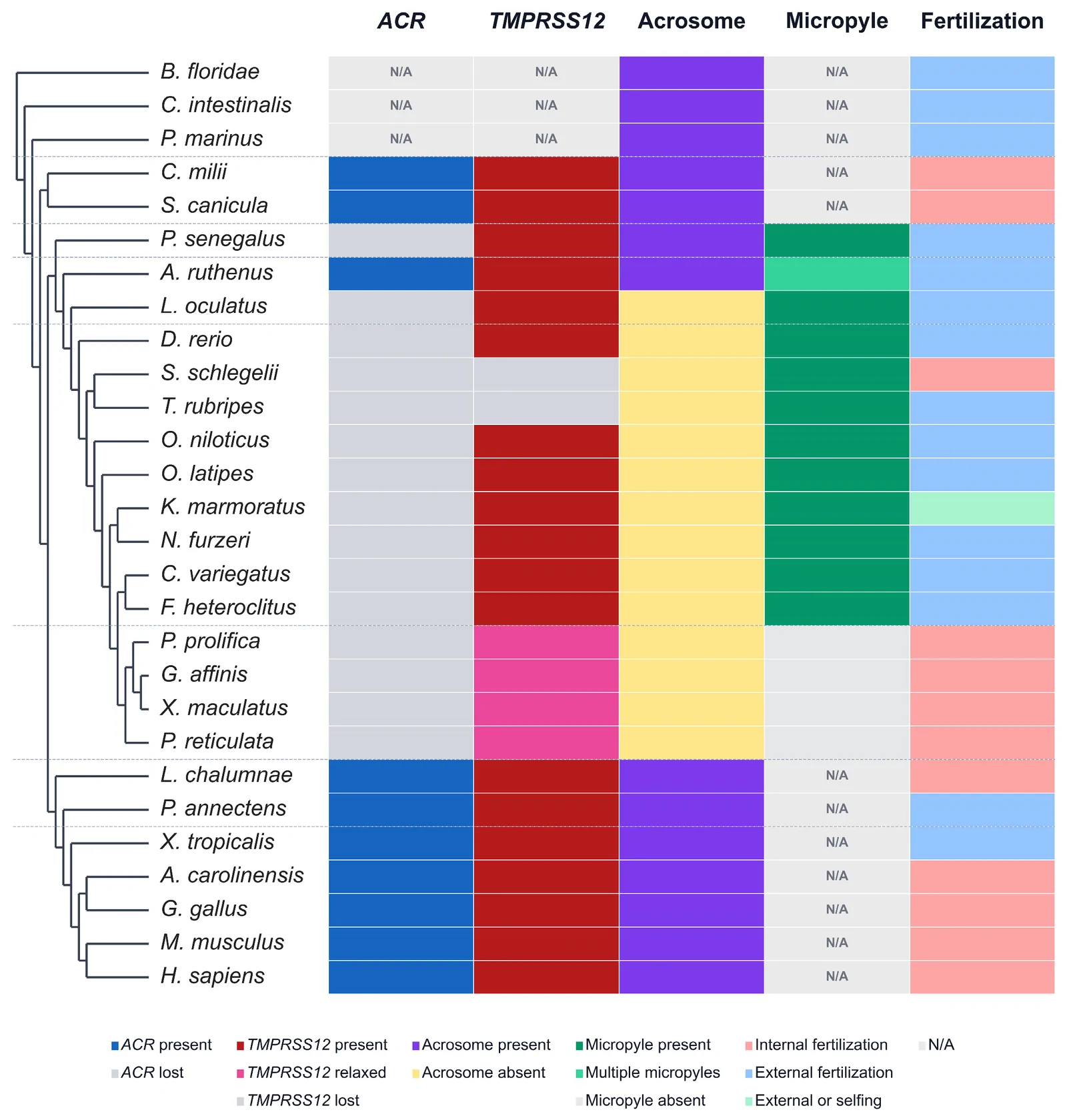
Reproductive trait matrix of 28 representative vertebrate species. Left: phylogenetic tree showing the relationships among the species. Right: character-state matrix for five traits: *ACR*, *TMPRSS12*, acrosome, micropyle, and fertilization mode. Species names are italicized and abbreviated using the genus initial followed by the specific epithet. Horizontal dashed lines separate the major clades. The color legend is shown at the bottom of the figure. For *ACR*, dark blue indicates presence and light grey indicates loss. For *TMPRSS12*, dark red indicates presence, pink indicates relaxed selection, and light grey indicates loss. For acrosome, purple indicates presence and pale yellow indicates absence. For micropyle, dark green indicates presence, light green indicates multiple micropyles, and pale grey indicates absence. For fertilization mode, light red indicates internal fertilization, light blue indicates external fertilization, and pale green indicates selfing (internal self-fertilization, with occasional external outcrossing). Grey (N/A) indicates that the trait is not applicable to the species.

The reproductive trait matrix of the 28 representative species revealed pronounced differences in the distribution of *ACR* and *TMPRSS12* across vertebrates (Figure 1). *ACR* was retained in chondrichthyans (*Callorhinchus milii* and *Scyliorhinus canicula*), sarcopterygians (*Latimeria chalumnae* and *Protopterus annectens*), and tetrapods. Among the non-teleost actinopterygians included in the trait matrix, *Acipenser ruthenus* retained *ACR*, whereas *ACR* was not detected in *Polypterus senegalus* or *Lepisosteus oculatus*. *ACR* was also not detected in any of the 13 examined teleosts, including *Danio rerio*, *Oryzias latipes*, and *Oreochromis niloticus* (Figure 1; Table S3C). These assignments were based on sequence-similarity searches together with conserved flanking-gene synteny, rather than on the absence of an annotated gene alone.

In contrast, *TMPRSS12* was present in nearly all examined jawed vertebrates, including chondrichthyans, non-teleost actinopterygians, teleosts, sarcopterygians, and tetrapods. In Poeciliidae, *tmprss12* showed evidence of relaxed selection. *TMPRSS12* was broadly conserved across the examined vertebrates but was not detected in the examined species of *Sebastes* or *Takifugu*. Neither miniprot nor tBLASTn recovered a qualifying *TMPRSS12* ortholog from the expected syntenic intervals in these species (Table S3B,C). Although a highly divergent homolog cannot be excluded absolutely, the *dip2ba–scn8a* interval in *Sebastes* and *Takifugu* was essentially gap-free, and no *tmprss12* coding sequence was recovered by the genome-level searches. These observations support lineage-specific loss. Analysis of the testis RNA-seq also detected no *tmprss12* transcript with convincing sequence similarity to *tmprss12* in the examined samples (Table S13).

Among the three outgroup species, *B. floridae*, *C. intestinalis*, and *P. marinus*, sperm acrosomes were present, and fertilization was external, whereas the micropyle was absent. Among the examined jawed vertebrates, sperm acrosomes were present in chondrichthyans, sarcopterygians, tetrapods, bichirs, and acipenseriforms, whereas they were absent in holosteans and teleosts. Regarding the fertilization mode, chondrichthyans, certain sarcopterygians, and tetrapods predominantly use internal fertilization, whereas most teleosts use external fertilization. *Kryptolebias marmoratus* is the only teleost that reproduces by internal self-fertilization. The micropyle was observed only in actinopterygians. Acipenseriform eggs contained multiple micropyles, whereas poeciliids lacked these structures.

### 3.2. The Loss of ACR Is Highly Consistent with the Loss of the Acrosome

**Figure 2:**
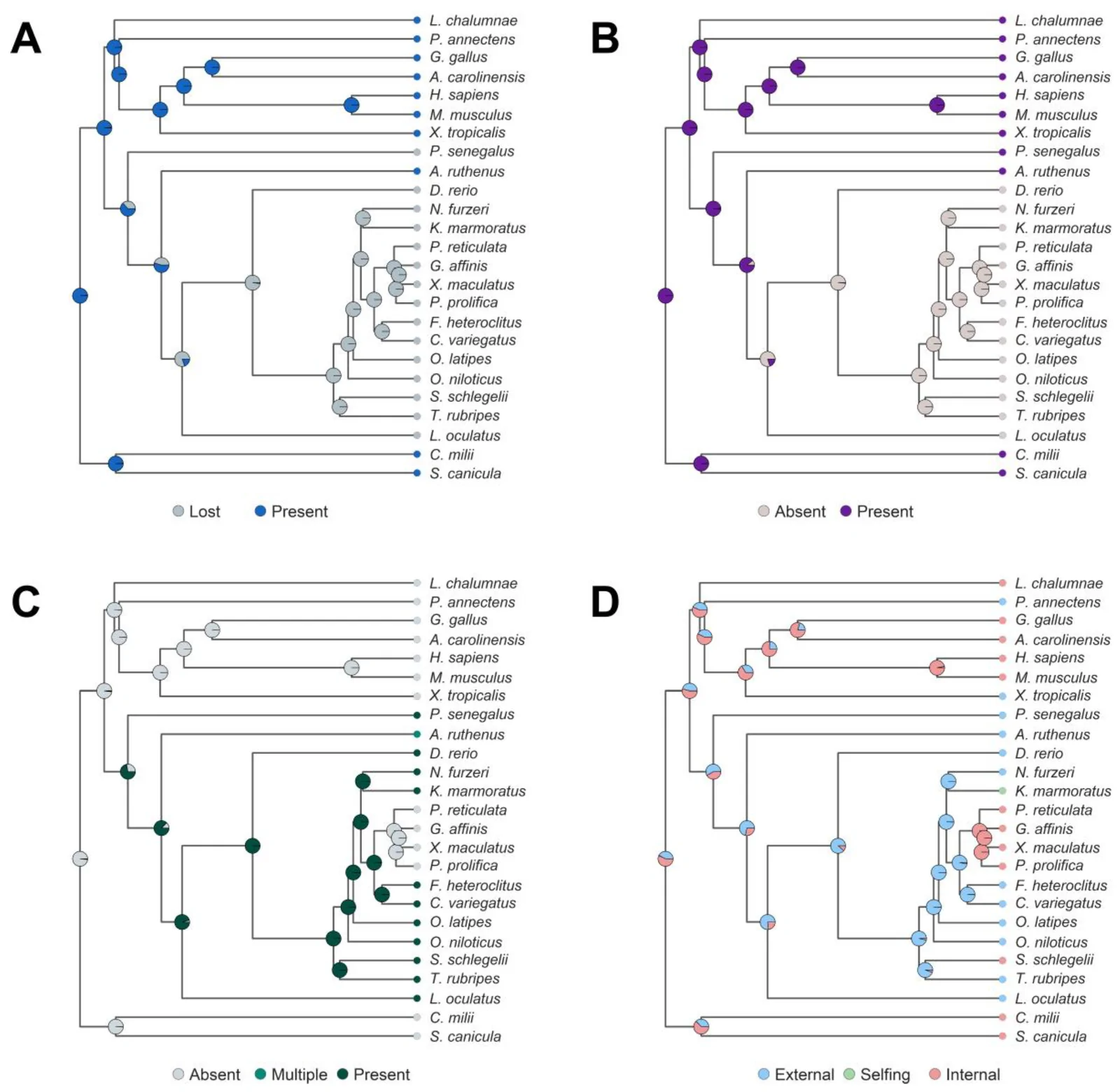
Ancestral state reconstruction of four reproductive traits. Maximum-likelihood ancestral-state reconstruction on a 25-species vertebrate phylogeny. Reconstructions used the ER model for *ACR*, Acrosome, and Micropyle and the SYM model for Fertilization, corresponding to the model with the lowest AIC for each trait; ER and SYM are equivalent for the two binary traits (Table S5A). Node pie charts show ancestral-state probabilities; terminal dots show observed states of extant species. (**A**) *ACR* gene state. Blue-grey: Lost; blue: Present. (**B**) Sperm acrosome state. Light tan: Absent; purple: Present. (**C**) Egg micropyle state. Light grey: Absent; teal: Multiple micropyles; dark green: Present. (**D**) Fertilization mode. Light blue: External; light green: Selfing (internal self-fertilization, with occasional external outcrossing); light red: Internal.

The probability of *ACR* presence in the jawed vertebrate ancestor was 0.988, and the probability of *ACR* absence in the teleost ancestor was 0.979 (Table S5C). The distribution of *ACR* in vertebrates closely corresponds to the presence of the sperm acrosome. Ancestral state reconstruction estimated a probability of 1.000 that the jawed vertebrate ancestor had an acrosome, suggesting that both the acrosome and *ACR* had already emerged in this ancestor. Chondrichthyans and sarcopterygians (including tetrapods) generally retained the acrosome and *ACR*; teleosts without acrosomes lacked *ACR*. Among non-teleost actinopterygians, bichirs retained the sperm acrosome but lacked *ACR*, gars lacked both, and acipenseriforms retained both the acrosome and the complete *ACR* gene (Figure 2A,B).

Micropyles were restricted to actinopterygians, and ancestral state reconstruction indicated a 0.985 probability that the jawed vertebrate ancestor lacked micropyles. Most actinopterygians with micropyles lacked acrosomes or *ACR* (Figure 2C). Acipenseriforms were a notable exception, retaining acrosomes and complete *ACR* despite having eggs with multiple micropyles, whereas bichirs retained the acrosome but lacked *ACR*. Compared with the acrosomes and micropyles, the correspondence between the fertilization mode and *ACR* distribution was significantly weaker. Internal fertilization occurred independently numerous times in chondrichthyans, certain teleosts, and tetrapods; however, *ACR* retention in these groups was inconsistent (Figure 2D). Leave-one-out sensitivity analysis showed that the probability of *ACR* presence in the jawed vertebrate ancestor (0.988) varied little (SD = 0.010). In contrast, *ACR* absence in the teleost (0.979; SD = 0.033) and neopterygian (0.804; SD = 0.097) ancestors and external fertilization in the actinopterygian ancestor (0.585; SD = 0.047) showed greater sensitivity to single-species observations, as did internal fertilization in the jawed vertebrate ancestor (0.574; SD = 0.047; Table S5C). For the two binary traits (*ACR* and acrosome), ancestral-state estimates at key deep nodes were consistent across transition models, whereas the multi-state traits (micropyle and fertilization) showed model-dependent estimates at some deep nodes (Table S5B). Model comparison identified ER as the lowest-AIC model for *acr*, acrosome, and micropyle, and SYM for fertilization (Table S5A). For example, *ACR* was reconstructed as present in the jawed-vertebrate ancestor under ER (0.988), SYM (0.988), and ARD (1.000).

### 3.3. ACR and TMPRSS12 Are Ancient Paralogs Consistent with a 2R-WGD Origin

**Figure 3:**
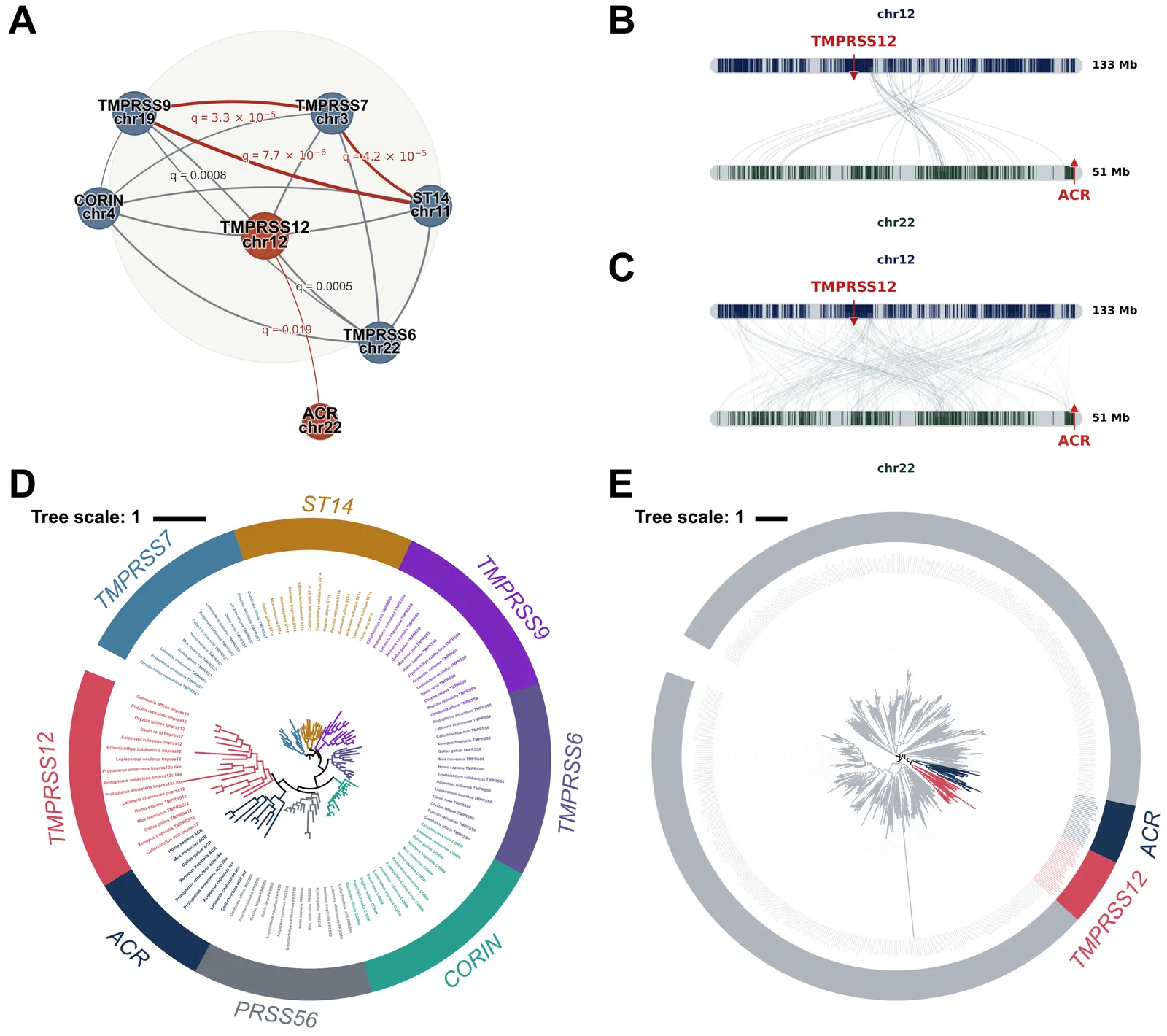
Ohnolog network, chromosomal synteny, and phylogenetic analysis of *ACR* and *TMPRSS12*. (**A**) Candidate ohnolog network centered on human *TMPRSS12*, based on OHNOLOGS v2 relaxed-level results. Nodes represent candidate ohnologs; node size scales with degree. Node colors distinguish genes. Red edges: outgroup support OS = 3; gray edges: OS = 1; edge width scales with −log_10_[q(out)]. (**B**) Standard MCScanX synteny ribbon between human chr12 (top) and chr22 (bottom). Blue and green ticks: gene positions; gray curves: anchor gene pairs within collinear blocks. Red arrows and labels mark *TMPRSS12*, *ACR*, and *TMPRSS6*. Parameters: ≥5 genes/block, ≤20 gaps, e ≤ 1 × 10^−5^. (**C**) As in (**B**) with relaxed parameters: ≥3 genes/block, ≤50 gaps, e ≤ 1 × 10^−3^. (**D**) Maximum-likelihood tree of *TMPRSS12* and its seven related ohnolog family members (114 sequences). Different gene families are distinguished by branch color and outer ring. Node support assessed by UFBoot and SH-aLRT. Scale bar: substitutions per site. (**E**) OrthoFinder gene tree for orthogroup OG0000013, inferred from 30 representative vertebrate proteomes. The *TMPRSS12* clade is highlighted in brown-red, the *ACR* clade in dark blue, and other serine protease family members in gray.

Database records, phylogenetic relationships, and chromosomal synteny were consistent with a 2R-WGD origin for *ACR* and *TMPRSS12*. *ACR*–*TMPRSS12* was identified as a candidate ohnolog pair at the relaxed level (q < 0.05). Among the six candidate ohnolog pairs associated with *TMPRSS12*, *ACR* paired exclusively with *TMPRSS12* and had the highest outgroup support score (OS = 3/3; Figure 3A). MCScanX analysis of human chr12 versus chr22 identified four collinear blocks under standard parameters (Figure 3B) and 26 blocks under relaxed parameters, covering 20.7% of the protein-coding genes on the two chromosomes (Figure 3C). Among the 20 relaxed-level candidate pairs in OHNOLOGS v2, eight were located within the collinear blocks described above. These pairs belonged to several gene families with different functions, including the intermediate filament superfamily (*PRPH*–*NEFH*), solute carrier family (*SLC5A1*–*SLC5A8*), and inwardly rectifying potassium channel family (*KCNJ4*–*KCNJ8*) (Table S14).

Gene family phylogenetics supported the closest homologous relationships. In a maximum-likelihood tree of 114 sequences from *TMPRSS12* and its seven related ohnolog family members (*PRSS56* was recorded as an ohnolog of *TMPRSS9* in OHNOLOGS v2), *ACR* and *TMPRSS12* formed a sister clade that was apparently separated from *TMPRSS6*, *ST14*, and other family members (Figure 3D). OrthoFinder orthogroup inference of 30 representative proteomes identified *TMPRSS12* as the closest homolog of *ACR* within the same orthogroup, OG0000013 (Figure 3E). OrthoFinder placed the *ACR*–*TMPRSS12* duplication event in the common ancestor of jawed vertebrates on its inferred species tree (Figure S1); this gene-tree node corresponding to the inferred duplication was strongly supported (SH-aLRT = 100; UFBoot = 100; Table S15). The phylogenetic, synteny, and ohnolog-classification evidence was considered jointly, since no single result is conclusive on its own.

**Figure 4:**
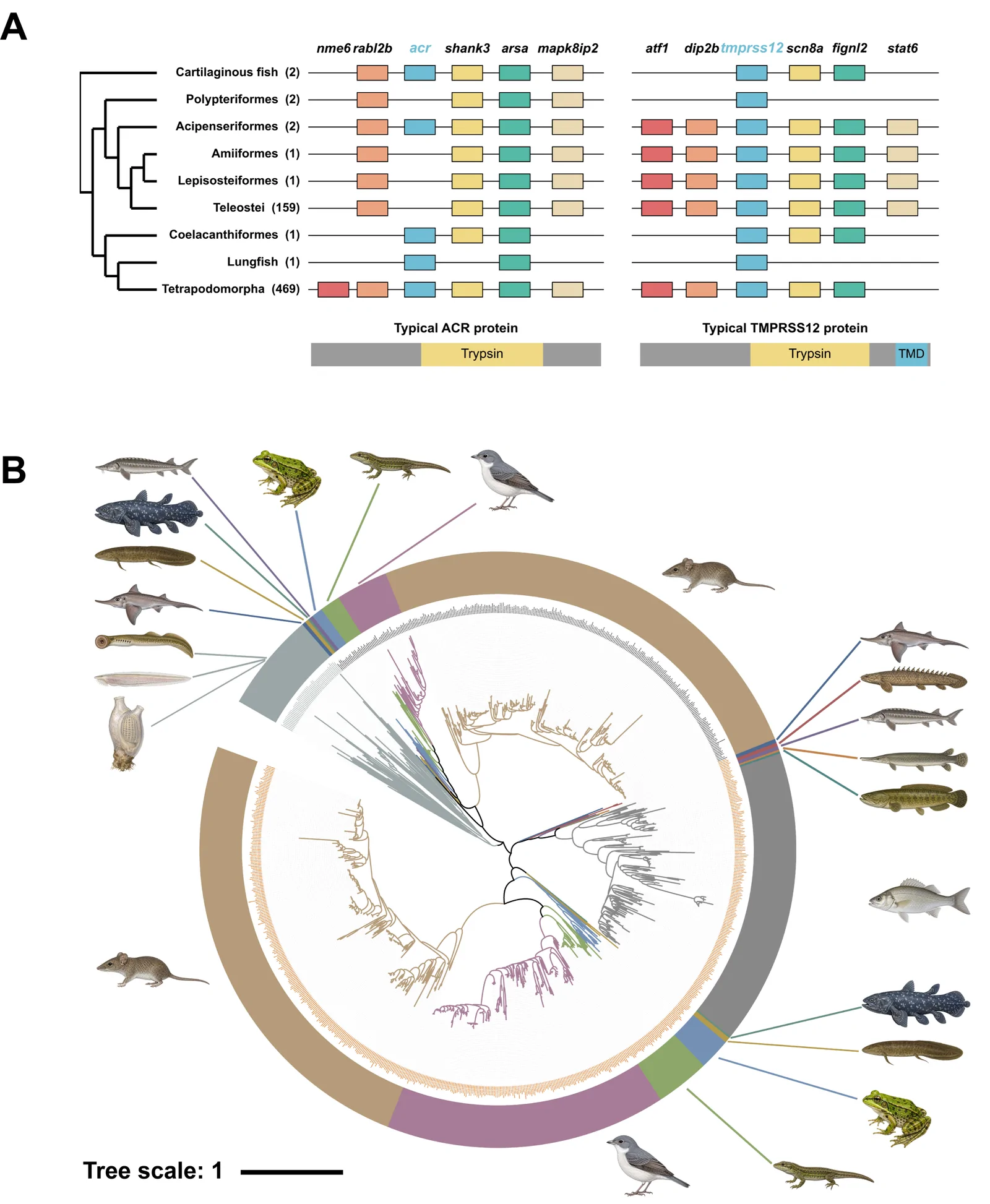
Multi-species locus synteny and large-scale phylogeny of *ACR* and *TMPRSS12*. (**A**) Schematic of *ACR* (left) and *TMPRSS12* (right) locus synteny across vertebrates. Each row represents one vertebrate group (species count in parentheses). Gray lines: chromosomal segments; colored rectangles: collinear genes, with identical colors indicating orthologs; gaps: gene not detected. Below: domain architecture of human *ACR* and *TMPRSS12* (Trypsin: catalytic domain). (**B**) Maximum-likelihood phylogeny of 966 protein sequences, including 301 *ACR* sequences and 612 *TMPRSS12* sequences from 638 extant jawed vertebrate species, together with 53 outgroup sequences from orthogroup OG0000013 (*B. lanceolatum*, *C. intestinalis*, and *P. marinus*). Tip labels: *ACR*, black; *TMPRSS12*, orange; outgroup sequences, grey. The outer ring indicates major vertebrate clades. Scale bar: substitutions per site.

Multispecies synteny showed that the *TMPRSS12* locus was highly conserved across jawed vertebrates, with flanking anchor genes (*DIP2B*, *SCN8A*, and others) retaining a conserved collinear arrangement from chondrichthyans through tetrapods to most actinopterygians. In contrast, recognizable flanking-gene synteny around the *ACR* locus was retained in actinopterygians, although no *ACR* coding sequence was recovered from most of the examined lineages. Among the sampled actinopterygians, *ACR* was retained only in acipenseriforms (Figure 4A). The conservation of the surrounding genomic region, together with the failure of genome-level searches to recover an *ACR* coding sequence, supports gene loss rather than a missing annotation, although a highly divergent homolog cannot be ruled out completely. To further analyze the phylogenetic relationship between *TMPRSS12* and *ACR*, we constructed a maximum-likelihood tree based on 966 protein sequences validated through synteny, including 301 *ACR* sequences and 612 *TMPRSS12* sequences, covering 638 extant jawed vertebrate species (Figure 4B). The rooting outgroup consisted of 53 sequences from three species in OG0000013: amphioxus (*B. lanceolatum*, 18 sequences), sea squirt (*C. intestinalis*, 21 sequences), and lamprey (*P. marinus*, 14 sequences). After rooting with the outgroup, *ACR* and *TMPRSS12* formed monophyletic groups and were sister groups with relatively high support (Table S15). Within the *ACR* clade, chondrichthyans (*Callorhinchus milii* and *Hemiscyllium ocellatum*) were positioned at the most basal position, followed by lungfish, coelacanth and acipenseriforms, and subsequently tetrapods. Within the *TMPRSS12* clade, chondrichthyans also occupied the basal position, followed by bichirs, acipenseriforms, bowfin, gar and teleosts. The internal topological relationships of both branches were consistent with the known vertebrate phylogenetic framework.

### 3.4. TMPRSS12 Undergoes Lineage-Specific Selection Shifts and Independent Losses

**Figure 5:**
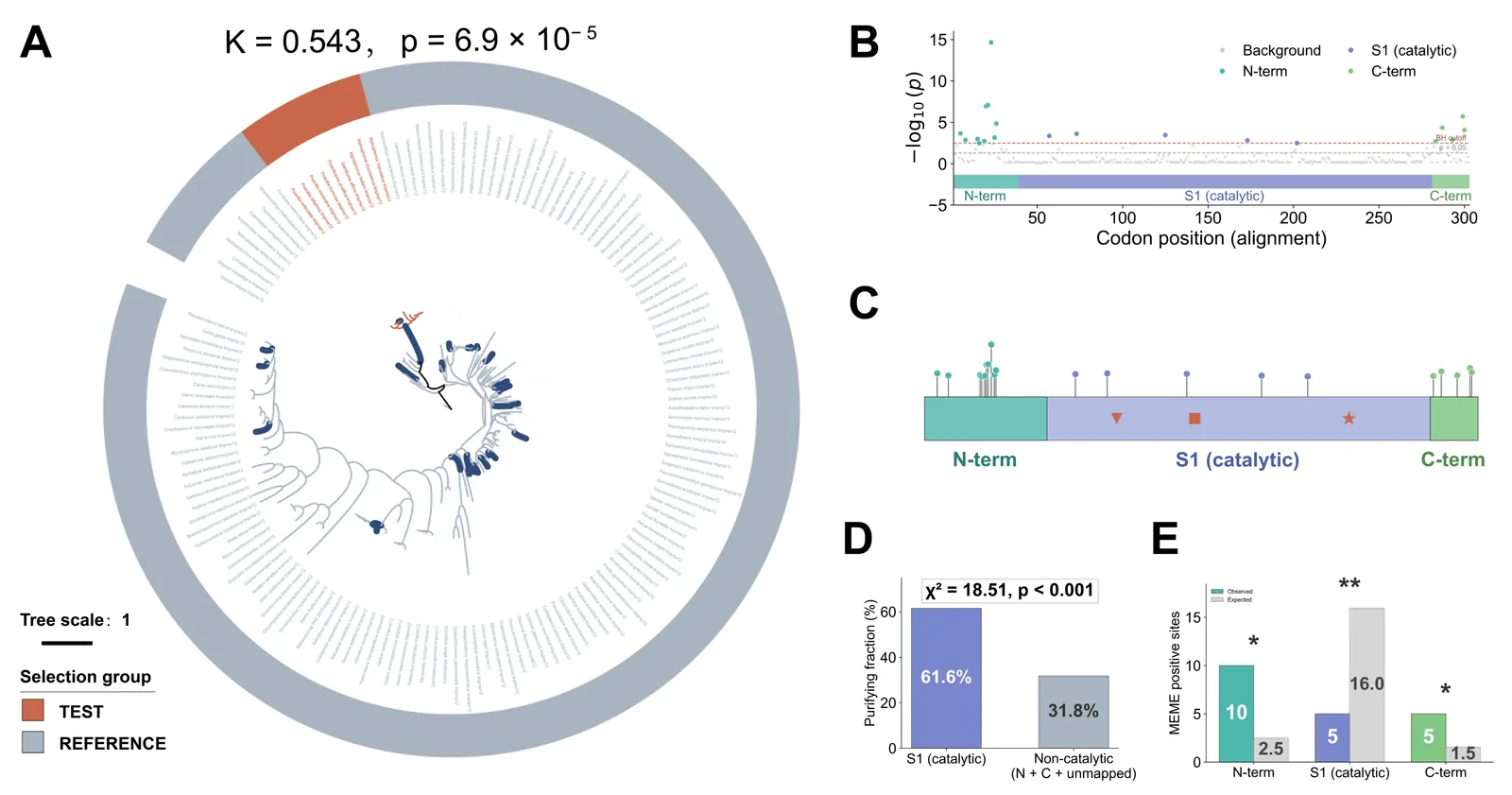
Selection pressure analyses of *tmprss12*. (**A**) Maximum-likelihood phylogenetic tree based on the *TMPRSS12* codon alignment from 146 actinopterygian species. Together, the stem branch leading to the poeciliid MRCA and all descendant branches (17 branches in total) were defined as the foreground group (TEST, brick red), whereas the remaining actinopterygian branches were defined as the background group (REFERENCE, gray) for RELAX analysis. Dark blue branches indicate significant episodic positive selection detected by aBSREL (Holm-adjusted *p* < 0.05). The RELAX K and *p* values are shown above the tree. (**B**–**E**) Site-level selection analyses were performed across the full 146-species actinopterygian tree. (**B**) Distribution of significant MEME sites along the codon alignment. Colored points indicate significant sites after Benjamini–Hochberg correction (FDR < 0.05), with colors indicating their locations in the N-terminal region, S1 catalytic domain, and C-terminal region. Gray points represent non-significant sites. Dashed lines indicate the significance thresholds, and the colored bar shows the domain positions in the alignment. (**C**) Positions of MEME-significant sites mapped to the human *TMPRSS12* protein. Lollipop heights represent −log10(P), and colors indicate domain locations. Red symbols mark the catalytic triad residues His122, Asp171, and Ser268. (**D**) Proportions of codons under purifying selection in the S1 catalytic domain and non-catalytic regions. Statistical significance was tested using a chi-square test. (**E**) Observed and expected numbers of MEME-significant sites in the N-terminal region, S1 catalytic domain, and C-terminal region. Expected values were calculated according to domain length. Asterisks indicate significance in permutation tests (* *p* < 0.05, ** *p* < 0.01).

RELAX analysis compared the selection intensity between the foreground branch set of Poeciliidae (the stem branch leading to the poeciliid MRCA plus all descendant branches) and the background branches of other actinopterygians, resulting in a relaxation parameter K = 0.543, *p* = 6.9 × 10^−5^ (Figure 5A; Table S16). This result indicates a relative reduction in selection intensity in the Poeciliidae foreground branches. The ω distribution in both background and foreground branches included three categories, corresponding to strong purifying, neutral, and positive selection. For the strong purifying category, ω was 0.014 in the background branches and 0.099 in the foreground branches. For the positive selection category, ω was 71.8 in the background branches and 10.2 in the foreground branches. The neutral category had an ω value of 1.00 in both groups (Table S17). At the gene level, BUSTED detected significant gene-wide evidence of episodic diversifying selection, indicating that at least one site on at least one branch in the phylogeny experienced ω > 1 (likelihood ratio test, LRT = 248.33, *p* < 0.001). At the branch level, aBSREL identified 28 significant branches among 286 branches in the whole tree (Holm-corrected *p* < 0.05), including 22 terminal branches and six internal branches. For the Poeciliidae lineage, significant episodic diversifying selection was detected on the stem branch leading to Poeciliidae (Node266; Holm-corrected *p* = 0.0417) and on the terminal branch of *Poecilia mexicana* (Holm-corrected *p* = 1.71 × 10^−9^), whereas no internal crown branch was significant (Figure 5A; Table S18). Across the entire ray-finned fish tree, MEME identified 20 sites under episodic positive selection (FDR < 0.05), including 10 in the N-terminal region, five in the S1 catalytic domain, and five in the C-terminal region (Figure 5B,C; Table S19). Site-level FUBAR analysis (Table S20) showed that, across all 146 ray-finned fish sequences, 61.6% of codons in the S1 catalytic domain were under purifying selection (posterior probability > 0.9; 146/237), compared with 31.8% in the non-catalytic regions (21/66; comprising the 37 N-terminal, 23 C-terminal, and six unmapped codons) (χ^2^ = 18.51, *p* < 0.001). Excluding the six unmapped codons (gambusia-specific insertions with no counterpart in human *TMPRSS12*) did not change this result (146/237 vs. 18/60, χ^2^ = 19.34, *p* < 0.001). FEL (Table S21) provided independent support for these classifications. The dN/dS values of the catalytic triad His122, Asp171, and Ser268 were all 0 (Table S21). Compared with the expected numbers based on the number of codons in each region, the observed numbers of selected sites were higher in the N- and C-terminal regions and lower in the S1 catalytic domain (N-terminus: 10 vs. 2.49; S1 catalytic domain: 5 vs. 15.96; C-terminus: 5 vs. 1.55). Coverage-stratified permutation analysis supported significant domain-specific enrichment or depletion (10,000 iterations; N-terminus: *p* = 0.042; S1 catalytic domain: *p* = 0.002; C-terminus: *p* = 0.027; Figure 5E; Table S22).

The relaxation signal remained significant under the primary stem-plus-crown foreground definition across alternative trimming thresholds (K = 0.541–0.551, *p* < 0.001) and after removing any single tested species (K = 0.515–0.547, *p* < 0.001). Relaxation was also detected when the foreground was restricted to the crown branches alone (excluding the stem; K = 0.666, *p* = 0.0029) and to the stem branch alone (K = 0.167, *p* = 4.3 × 10^−4^), whereas the terminal-only estimate, although still in the direction of relaxation (K = 0.714), did not reach statistical significance (*p* = 0.057). This pattern suggests heterogeneity in the relaxation signal across different portions of the poeciliid phylogeny (Table S9).

**Figure 6:**
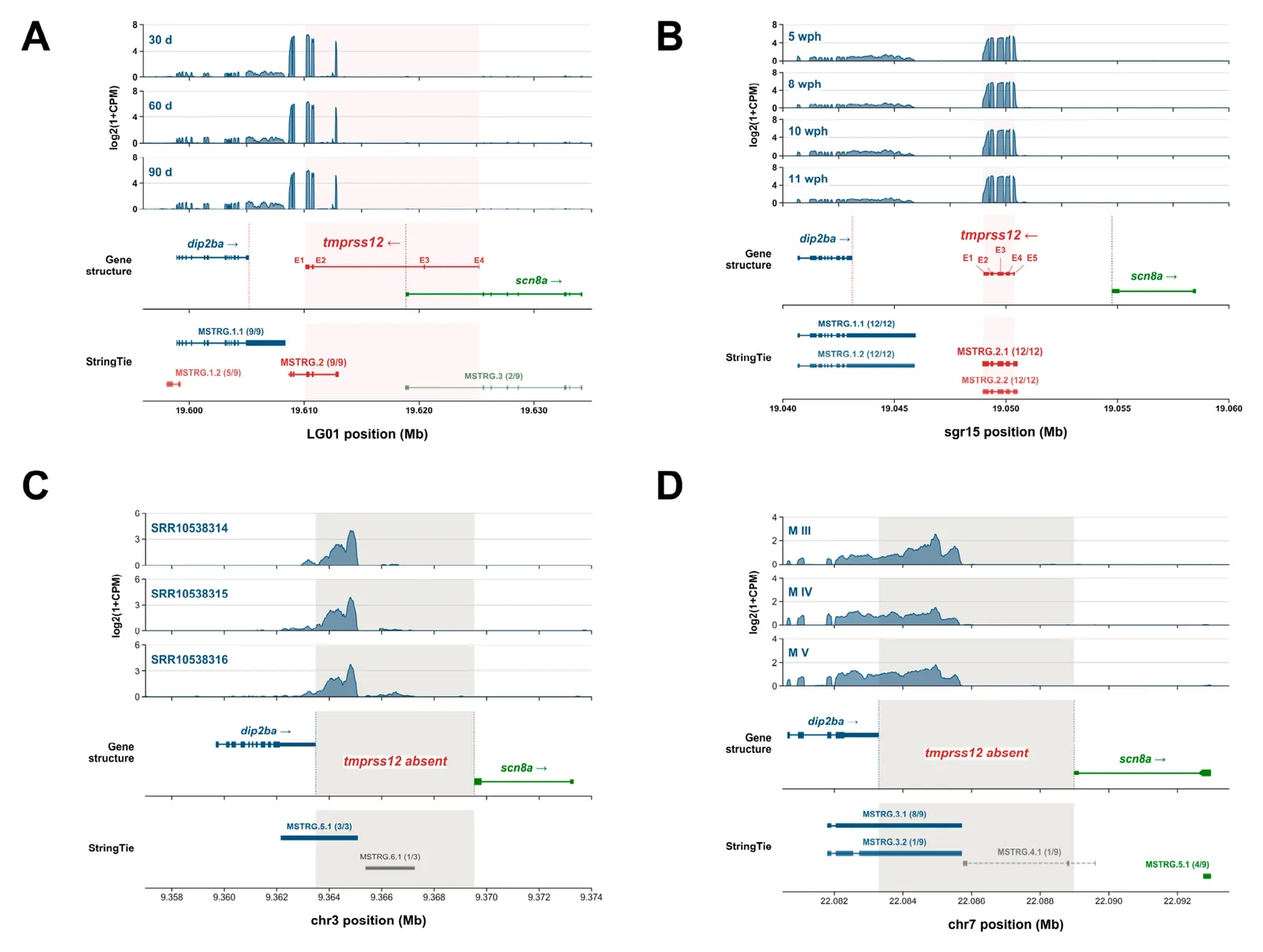
RNA-seq coverage and StringTie transcript models at the *dip2ba*–*tmprss12*–*scn8a* syntenic region in four teleost species. (**A**) *G. affinis* testis at three developmental stages (30, 60, and 90 d) on LG01. MSTRG.2 was detected in all nine samples (9/9), and the antisense transcript MSTRG.1.2 was detected in five samples (5/9). (**B**) *N. furzeri* at four developmental time points (5, 8, 10, and 11 wph) on sgr15. Two *tmprss12* transcript models, MSTRG.2.1 and MSTRG.2.2, were detected in all 12 samples. (**C**) *T. rubripes* testis from three RNA-seq samples on chr3. MSTRG.5.1 was detected in all three samples (3/3), while MSTRG.6.1 was detected in one sample (1/3). (**D**) *S. schlegelii* testis at three maturation stages (M III, M IV, and M V) on chr7. MSTRG.4.1 was detected in one of nine samples (1/9). In all panels, RNA-seq coverage is shown as log_2_(1 + CPM). Gene structure tracks show exon–intron organization, with wide rectangles representing exons and thick bars representing CDS regions. StringTie tracks show assembled transcript models, with numbers in parentheses indicating the number of samples in which each transcript was detected. Dashed vertical lines mark the *dip2ba* stop codon and *scn8a* start codon. The corresponding *tmprss12* region is highlighted by a light red background.

The genome-wide searches recovered no *ACR* or *TMPRSS12* ortholog in the *T. rubripes* or *S. schlegelii* genome; low-similarity hits outside the expected syntenic intervals were identified by BLASTp best-hit as other members of the S1 serine protease family (Table S3D). Across the *dip2ba–tmprss12–scn8a* locus, *G. affinis* (Figure 6A) and *N. furzeri* (Figure 6B) exhibited apparent RNA-seq coverage and StringTie-assembled transcripts. In contrast, *T. rubripes* (Figure 6C) and *S. schlegelii* (Figure 6D) exhibited limited transcriptional evidence in their corresponding regions. In *S. schlegelii*, one low-confidence transcript (MSTRG.4.1) was detected in one of nine samples. In *T. rubripes*, one low-confidence transcript (MSTRG.6.1) was detected in one of three samples. Neither transcript showed significant protein-level similarity to *tmprss12* by tBLASTn (Table S13).

The *dip2ba*–*scn8a* interval was contiguous in all four species, with only 0–1 N bases (Table S13). TBLASTn searches of low-confidence StringTie transcripts showed no significant protein-level similarity to *tmprss12* in *T*. *rubripes* or *S*. *schlegelii* (E-values near or above 1). Thus, neither transcriptomic nor genome-level searches recovered evidence of *tmprss12* at the expected locus. Together with the near-complete syntenic interval, these results support lineage-specific loss of *tmprss12* in *T*. *rubripes* and *S*. *schlegelii*, rather than absence caused by assembly gaps or missing annotation.

### 3.5. ACR and TMPRSS12 Show Predominantly Testis-Biased Expression

**Figure 7:**
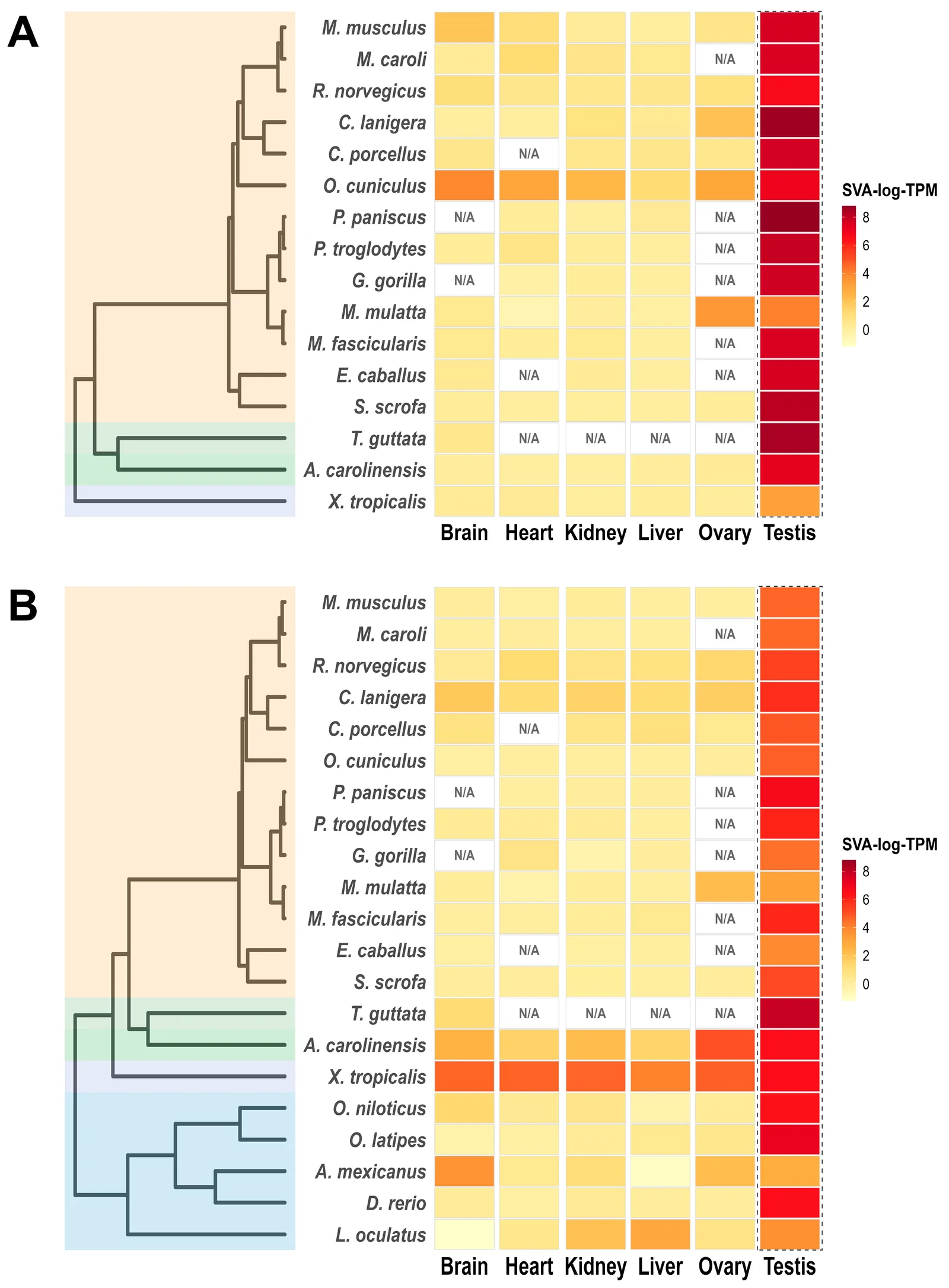
Tissue expression patterns of *ACR* and *TMPRSS12*. (**A**) *ACR* expression across six tissues (brain, heart, kidney, liver, ovary, testis) in 16 tetrapods. Left: TimeTree-based species phylogeny with clade background shading (mammals, birds, reptiles, amphibians). Columns: tissues; rows: species. Color intensity: SVA-log-TPM; N/A: data not available. Testis column outlined with dashed box. (**B**) *TMPRSS12* expression across six tissues in 21 vertebrates. Layout as in (**A**), with the addition of fish branches. Expression data (SVA-log-TPM) are from the amalgamated cross-species transcriptome compendium of Fukushima and Pollock [37], which combined 1903 RNA-seq experiments from 182 BioProjects across six organs and 21 vertebrate species. Each cell shows the mean SVA-log-TPM for the corresponding species–tissue combination across available samples; sample sizes vary by species and tissue.

Multispecies tissue transcriptomic analysis revealed that *ACR* and *TMPRSS12* exhibit pronounced testis-biased expression. *ACR* was predominantly or exclusively expressed in the testes of 16 tetrapod species, with low expression in the brain, heart, kidney, liver, and ovaries (Figure 7A). Similarly, *TMPRSS12* exhibited testis-dominant expression across 21 vertebrate species with low expression in other tissues (Figure 7B). In *G. affinis*, RT-qPCR showed the highest *tmprss12* expression in the testis (Figure S2), and RT-PCR followed by agarose gel electrophoresis detected a *tmprss12* amplification product specifically in the testis (Figure S3).

Among the 33 sequences examined (10 *ACR* and 23 *TMPRSS12* orthologs), all contained a trypsin-like serine protease domain (Pfam PF00089), and 26 had an N-terminal signal peptide comprising 10–47 amino acids. The catalytic triad was fully conserved in all 10 *ACR* sequences. Among the 23 *TMPRSS12* sequences, 21 retained the complete catalytic triad, with two exceptions: *P. senegalus* (His→Thr and Ser→Glu) and *P. annectens* (Ser→Ile) (Figures S4 and S5).

## 4. Discussion

In fish, *tmprss12* has long been annotated as “acrosin” or “acrosin-like” in databases such as NCBI and Ensembl. One study even treated zebrafish *tmprss12* and “acrosin-like” as two separate genes in knockdown experiments [43]. However, both fish “acrosin” and “acrosin-like” actually refer to *tmprss12*, which is a paralog of tetrapod *ACR* rather than its ortholog. Database records, phylogenetic analyses, and evidence from paralogon remnants support the inference that *ACR* and *TMPRSS12* are ancient paralogs, with an origin that may be associated with 2R-WGD.

### 4.1. ACR Retention Is Associated with Acrosome Presence

Ancestral state reconstruction suggested that both *ACR* and the acrosome were present in the common ancestor of jawed vertebrates, suggesting a long-term evolutionary association between the gene and sperm structure. Their distribution across the major vertebrate lineages further supports this pattern. *ACR* is retained in chondrichthyans, sarcopterygians, tetrapods, and acipenseriforms, all of which contain acrosomes. In most other actinopterygians, *ACR* was lost together with the acrosome; bichirs, however, lost *ACR* while retaining the acrosome. The simultaneous loss of *ACR* and acrosomes in several actinopterygian lineages is consistent with a long-term evolutionary association between the two traits. However, the presence of a micropyle alone is not sufficient to explain *ACR* loss. The acipenseriforms are an important exception. Their eggs have multiple micropyles; however, their sperm retain an acrosome [44,45], and their genomes contain a complete *ACR* gene. This suggests that the appearance of the micropyle does not immediately eliminate the need for acrosomal function. However, changes in fertilization biology in certain actinopterygian lineages may have gradually reduced their dependence on acrosome-mediated penetration of the egg envelope [46]. After acrosome functionality was rendered non-essential, the selective constraint on *ACR* may have weakened, enabling neutral or slightly deleterious mutations to accumulate and eventually leading to gene loss [47,48,49]. *ACR* loss in teleosts may therefore reflect reduced functional demand and relaxed selective constraints; alternatively, it may represent an adaptive gene loss driven by micropylar fertilization [50]. The available comparative data do not distinguish among the population-genetic routes to fixation of *ACR* loss, including fixation under relaxed constraint or through neutral processes. Further investigation is needed to distinguish between these hypotheses.

The leave-one-out sensitivity analysis further showed differences in the robustness of these inferences. Leave-one-out masking showed that *ACR* presence in the jawed-vertebrate ancestor was highly stable (SD = 0.010). *ACR* absence in the teleost ancestor remained the most likely state across all replicates, although its probability varied (0.82–1.00; SD = 0.033). By contrast, the neopterygian estimate was more sensitive to masking individual species observations (0.38–0.98; SD = 0.097), with the most likely state changing in at least one replicate. In contrast, the state of the root of Actinopterygii was the most uncertain. The earliest diverging bichirs lacked *ACR* but retained the acrosome, whereas the next diverging acipenseriforms retained both. These conflicting states in adjacent lineages make it challenging to resolve the ancestral state using only the extant species. We placed the earliest reliably inferred loss of *ACR* in a neopterygian ancestor based on its consistent absence in both holosteans and teleosts. The state of deeper actinopterygian nodes requires additional evidence, such as paleogenomic data, for resolution.

### 4.2. Ancient Paralogy Consistent with a 2R-WGD Origin

The ohnolog relationship between *ACR* and *TMPRSS12* was supported by three complementary lines of evidence. First, under the relaxed criteria of OHNOLOGS v2, the two genes were identified as ohnolog pairs with the highest level of outgroup support (OS = 3/3). This classification integrates evidence from conserved synteny across species and phylogenetic relationships [22]. Second, phylogenetic reconstruction based on 966 sequences from 638 jawed vertebrates and 3 outgroup species showed that *ACR* and *TMPRSS12* formed two well-supported monophyletic sister clades. OrthoFinder inferred that the duplication occurred in the common ancestor of jawed vertebrates, consistent with the timing of 2R-WGD. Third, residual paralog signals remained between human chromosomes 12 and 22, which contained *TMPRSS12* and *ACR*, respectively. OHNOLOGS v2 identified 20 candidate ohnolog pairs from eight gene families between these chromosomes, eight of which were located within the syntenic blocks detected using MCScanX. *TMPRSS12* was also connected to a broader ohnolog network that included *TMPRSS6*, *ST14*, *TMPRSS7*, *TMPRSS9*, and *CORIN* on five additional chromosomes, further supporting its position within the ancient 2R-derived paralog. Although direct local synteny between *ACR* and *TMPRSS12* is no longer evident, these signals could have been weakened by gene loss and genome rearrangement during more than 400 million years of vertebrate evolution [22,51]. The ohnolog classification and residual paralogon structure are consistent with a 2R-WGD origin, but they do not exclude an earlier duplication or a local duplication followed by chromosomal rearrangement. The precise origin of *ACR* and *TMPRSS12* therefore remains uncertain.

Across vertebrates, *ACR* and *TMPRSS12* have followed contrasting evolutionary trajectories. *ACR* was widely retained in chondrichthyans, sarcopterygians, and tetrapods, but was lost in most actinopterygians, whereas *TMPRSS12* was conserved in most jawed vertebrates despite continued sequence and structural divergence. A similar pattern has been reported for *claudin-18* ohnologs in stomachless fishes, in which *cldn18a* was repeatedly lost while *cldn18b* was retained and evolved at an accelerated rate [49]. These findings suggest that the retention of ancient paralogs need not imply functional backup of the lost copy. As morphological or ecological conditions change, one copy may become dispensable, whereas another may be retained and continue to evolve. The contrasting fates of *ACR* and *TMPRSS12* are consistent with this pattern.

### 4.3. Relaxed Selection and Modular Evolution of tmprss12

*tmprss12* showed a relative relaxation of selection intensity in Poeciliidae under the baseline RELAX model. This result reflects a shift in selection strength on the foreground branches and does not show functional adaptation or reproductive specialization. aBSREL detected significant episodic diversifying selection on the stem branch leading to Poeciliidae (Node266) and on the terminal branch of *Poecilia mexicana*, whereas no internal crown branch was significant, and BUSTED provided gene-wide evidence of episodic diversifying selection somewhere in the phylogeny. These analyses indicate variation in selective regimes, but not a particular reproductive phenotype or adaptive mechanism. Similar mixed selection patterns have been reported for other reproductive proteins. For example, a considerable proportion of seminal fluid protein genes in Drosophila show relaxed selective constraints, whereas only a small number are under positive selection [52]. This indicates that the rapid divergence of reproductive proteins need not be driven by continuous positive selection.

Relaxation of selection on *tmprss12* in Poeciliidae was supported under multiple foreground definitions. Significant evidence of relaxation was obtained under both the stem-only foreground definition (K = 0.167, *p* = 4.3 × 10^−4^) and the crown-only foreground definition (K = 0.666, *p* = 0.0029), whereas the terminal-only analysis did not reach statistical significance (K = 0.714, *p* = 0.057). The significant stem-only and crown-only results, together with the weaker support from the terminal-only analysis, are consistent with heterogeneity in the relaxation signal across the poeciliid phylogeny, with deeper branches potentially contributing more strongly to the lineage-wide pattern. In addition, the relaxation signal remained statistically significant in all single-species removal analyses (K = 0.515–0.547; Table S9).

In ray-finned fish, the distribution of selection across different protein regions suggests that Tmprss12 has followed a modular evolutionary trajectory. The positively selected sites were mainly located in the N- and C-terminal regions, whereas the S1 catalytic domain was mainly under purifying selection and retained the conserved catalytic triad. Serine protease activity depends on highly organized active sites and their conformational networks. This strong constraint on the S1 catalytic domain is consistent with the requirement for maintaining its basic catalytic function [53]. The peptidase domains of human *TMPRSS12* and mouse *Tmprss12* are relatively conserved, whereas their peripheral accessory regions exhibit increased sequence divergence [19]. This pattern is consistent with stronger evolutionary constraint on the catalytic core and greater sequence flexibility outside it. Such regional heterogeneity may provide a substrate for functional divergence, but the present sequence analyses do not establish changes in protein localization, substrate recognition, molecular interactions, or reproductive specialization. These possibilities require direct structural and functional validation. Previous studies in mice have shown that Tmprss12 is involved in several reproductive processes, including sperm motility, *ADAM3*-related migration through the female reproductive tract, meiosis, and spermatogenesis [17,18].

### 4.4. Independent Losses of tmprss12 in Sebastes and Takifugu

The absence of *tmprss12* in *Sebastes* and *Takifugu* is consistent with two independent gene loss events. This inference is based primarily on the failure of genome-level searches to recover a *tmprss12* coding sequence from the expected syntenic interval. The lack of detectable *tmprss12* transcription provides additional support for the genome-based inference. These lineage-specific losses are unlikely to be a general consequence of teleost-specific whole-genome duplication, because most other teleosts that experienced the same duplication retained *tmprss12* [54]. When the functional importance of a gene is reduced by changes in its life history, ecology, or genetic background, relaxed selective constraints enable loss-of-function mutations to accumulate and become fixed, eventually leading to gene loss [55]. In *Sebastes*, the evolution of viviparity and associated changes in the reproductive environment may have altered the functional context of *tmprss12* [56]. In contrast, *Takifugu* has a highly compact genome and the loss of *tmprss12* may be related to long-term genome remodeling in this lineage [57]. These results suggest that the independent loss of *tmprss12* may have occurred in different lineage-specific reproductive and genomic backgrounds.

### 4.5. Distinct Subcellular Localization of ACR and TMPRSS12

Both *ACR* and *TMPRSS12* show broadly testis-biased expression in the species examined and generally retain a trypsin-like serine protease domain. These observations indicate conservation of tissue-expression bias and the trypsin-like domain, but do not establish functional equivalence or shared reproductive roles. However, they differ in terms of protein localization and mode of action. *ACR* is transported into the sperm acrosome as a proacrosin precursor and is activated and redistributed during the acrosome reaction. Limited proteolysis can remodel the zona pellucida and affect sperm binding and penetration. Genetic evidence from humans and hamsters further supports the important role of *ACR* in sperm–egg interactions [15,16]. *TMPRSS12* is a membrane-anchored type II transmembrane serine protease. Its loss affects sperm motility and migration through the female reproductive tract, as well as spermatogenesis, acrosome reaction, and sperm–egg interactions [17,18]. Although *ACR* and *TMPRSS12* retain a similar catalytic domain, their different subcellular localizations could allow access to different substrates. Whether these localization differences translate into distinct substrate interactions or biological functions requires direct functional analysis.

### 4.6. Limitations and Future Directions

This study had several limitations. First, direct functional evidence for *TMPRSS12* is derived primarily from mammals, and functional data from fishes are lacking. Whether the observed testis-biased expression and trypsin-like domain translate into equivalent reproductive functions across vertebrates requires direct functional evidence. Expression patterns at the cell-type, developmental-stage, and subcellular levels require further characterization; substrates and physiological roles remain to be examined. Second, acipenseriforms retained the acrosome, micropyle, and *ACR* simultaneously; however, whether *ACR* functions beyond the acrosome in acipenseriforms is unknown. Third, ancestral state reconstruction was based on a limited species set, and leave-one-out analyses, in which each species’ character state was treated as unknown in turn, showed that several deeper ancestral states were more sensitive to individual species observations. The observed near-correspondence between *ACR* retention and acrosome presence is descriptive and does not establish causality. By contrast, associations with fertilization mode and micropyle presence were less consistent and included lineage-specific exceptions. Fourth, whether the “acrosin-like” label systematically contaminates the annotation of other sperm proteases in databases requires evaluation within a broader gene family context. Fifth, the selection analyses are limited by codon-alignment processing, taxon sampling, and foreground-branch definition. Evidence of relaxation remained significant under the tested trimming thresholds and taxon-removal analyses, as well as under the stem-only and crown-only foreground definitions, but support weakened when only terminal Poeciliidae branches were the foreground. The precise phylogenetic distribution and timing of the inferred relaxation remain uncertain. Selection tests alone cannot establish the functional consequences of *TMPRSS12* divergence, which need direct experimental validation.

## 5. Conclusions

This study shows that *ACR* and *TMPRSS12* originated from an ancient duplication event that may be associated with vertebrate 2R-WGD and later followed different evolutionary paths. The retention of *ACR* is closely associated with the presence of sperm acrosomes, while its repeated loss in acrosome-lacking lineages suggests a link between *ACR* retention and the evolution of reproductive structures. In contrast, *TMPRSS12* has been widely conserved in jawed vertebrates, although some lineages show relaxed selection pressure or independent gene loss. These results clarify the different evolutionary patterns of these two homologous serine proteases and indicate that fish *tmprss12* should not be annotated as an “acrosin-like” gene only based on sequence similarity. The genomic patterns reported here do not establish that *TMPRSS12* retained a reproductive function after *ACR* loss. This remains a hypothesis that requires direct functional validation. Together, these findings provide an evolutionary framework for understanding how reproduction-related genes are differentially retained, modified, or lost during vertebrate diversification.

## Data Availability

The raw RNA-seq reads generated in this study have been deposited in the NCBI Sequence Read Archive (SRA) under BioProject accession PRJNA1345902. The final codon alignment, phylogenetic tree files, HyPhy JSON output files, and analysis scripts are openly available in Zenodo at https://doi.org/10.5281/zenodo.23770208. All other data analyzed in this study are publicly available and are listed in the Supplementary Materials.

## Supplementary Materials

The following supporting information can be downloaded at https://www.mdpi.com/article/10.3390/biology15212040/s1, Table S1: Protein data sources for OrthoFinder analysis of 30 representative species; Table S2: OG0000013 sequences used in Figure 3E; Table S3A: Assembly integrity of the anchor-defined intervals used for gene presence/absence assessment; Table S3B: *TMPRSS12* presence/absence validation by miniprot and tBLASTn genomic search with flanking-gene anchors (*dip2ba*–*scn8a*); Table S3C: *ACR* presence/absence validation by miniprot and tBLASTn genomic search with flanking-gene anchors (*shank3*–*rabl2b*); Table S3D: BLASTp best-hit identification of out-of-interval miniprot hits for the *ACR* and *TMPRSS12* queries in gene-lost species; Table S4: Trait matrix for ancestral state reconstruction; Table S5: Model selection and leave-one-out sensitivity analysis for ancestral state reconstruction; Table S5A: Model selection; Table S5B: Model sensitivity: ancestral-state probabilities under ER, ARD, and SYM; Table S5C: Leave-one-out node sensitivity; Table S6: Eight gene family sequences used in Figure 3D; Table S7: *ACR* and *TMPRSS12* sequences used in Figure 4B and Figure 5A; Table S8: Sequence filtering and alignment quality control for *TMPRSS12* codon alignment; Table S9: Robustness of RELAX results for poeciliid *TMPRSS12* to alignment trimming, taxon sampling, and foreground definition; Table S10: Testis RNA-seq data sources for four teleost species used in *TMPRSS12* loss validation; Table S11: Primer sequences and amplification characteristics; Table S12: Domain sequences used in Figures S4 and S5; Table S13: Assembly quality and transcriptional validation of the *dip2ba*–*scn8a* interval in four teleost species; Table S14: OHNOLOGS v2 candidate pairs between human chr12 and chr22 with MCScanX synteny validation; Table S15: Node support values for the *ACR*–*TMPRSS12* duplication and major clades; Table S16: Selection pressure analysis summary for poeciliid *TMPRSS12*; Table S17: RELAX rate class distribution for poeciliid *TMPRSS12*; Table S18: Branches with significant episodic diversifying selection detected by aBSREL; Table S19: MEME significant sites (FDR < 0.05) with codon positions, human residues, and domain assignments; Table S20: FUBAR site-specific selection results across all 146 ray-finned fish *TMPRSS12* sequences; Table S21: FEL site-specific selection results across all 146 ray-finned fish *TMPRSS12* sequences; Table S22: Domain enrichment of MEME positive-selection sites across all 146 ray-finned fish *TMPRSS12* sequences; Figure S1: OrthoFinder species tree and the *ACR*–*TMPRSS12* duplication node; Figure S2: *tmprss12* mRNA expression across eight tissues of adult male *Gambusia affinis*; Figure S3: Agarose gel electrophoresis of RT-PCR amplification products of *tmprss12* and *β-actin* in eight tissues of adult male *Gambusia affinis*; Figure S4: Domain organization of *ACR* proteins from ten vertebrate species; Figure S5: Domain organization of *TMPRSS12* proteins from 23 vertebrate species. References [58,59,60,61,62,63,64,65,66,67,68,69,70,71,72,73,74,75,76,77,78,79,80,81,82,83,84,85,86,87,88,89,90,91,92,93,94,95,96,97,98,99,100,101,102] are cited in the Supplementary Materials.

## Abbreviations

The following abbreviations are used in this manuscript:

2R-WGD	two rounds of whole-genome duplication
aBSREL	adaptive branch-site random effects likelihood
*ACR*	acrosin
AIC	Akaike information criterion
ARD	all-rates-different
ASR	ancestral state reconstruction
BIC	Bayesian information criterion
BUSTED	branch-site unrestricted statistical test for episodic diversification
CDS	coding sequence
CPM	counts per million
ER	equal-rates
FDR	false discovery rate
FEL	fixed effects likelihood
FUBAR	fast unconstrained Bayesian approximation
LRT	likelihood ratio test
MEME	mixed effects model of evolution
MRCA	most recent common ancestor
OS	outgroup support
RELAX	test for relaxed or intensified selection
RT-PCR	reverse transcription PCR
RT-qPCR	reverse transcription quantitative PCR
SH-aLRT	Shimodaira–Hasegawa approximate likelihood ratio test
SRA	Sequence Read Archive
SVA-log-TPM	surrogate variable analysis-corrected log-transcripts per million
SYM	symmetrical
*TMPRSS12*	transmembrane protease serine 12
UFBoot	ultrafast bootstrap
WGD	whole-genome duplication
