# Supplementary material for: Loss of ACR and Retention of Its Ancient Paralog TMPRSS12 in the Evolution of Teleost Fertilization
Source: Biology (Basel). 2026 Nov 8;15(21):2040. doi: 10.3390/biology15212040 (PMC13762310; doi:10.3390/biology15212040)
Supplement: Supplementary file 1 [file biology-15-02040-s001.zip › biology-4631742-supplementary.pdf]

Table S1. Protein data sources for OrthoFinder analysis of 30 representative species.

Species	Clade	Subclade	Genome Assembly	Annotation Version / Source
Branchiostoma floridae	Outgroup	Cephalochordata	Bfl_VNyyK (GCF_000003815.2)	NCBI RefSeq
Branchiostoma lanceolatum	Outgroup	Cephalochordata	BraLan2	Ensembl Release 62
Ciona intestinalis	Outgroup	Urochordata	KH	Ensembl Release 115
Petromyzon marinus	Outgroup	Cyclostomata	Pmarinus_7.0	Ensembl Release 115
Callorhinchus milii	Chondrichthyes	Holocephali	Callorhinchus_milii-6.1.3	Ensembl Release 115
Scyliorhinus canicula	Chondrichthyes	Elasmobranchii	sScyCan1.1 (GCF_902713615.1)	NCBI RefSeq
Acipenser ruthenus	Actinopterygii	Chondrostei	fAciRut3.2 (GCF_902713425.1)	NCBI RefSeq
Polyodon spathula	Actinopterygii	Chondrostei	ASM1765450v1 (GCF_017654505.1)	NCBI RefSeq
Lepisosteus oculatus	Actinopterygii	Holostei	LepOcu1	Ensembl Release 115
Danio rerio	Actinopterygii	Teleostei	GRCz11	Ensembl Release 115
Gasterosteus aculeatus	Actinopterygii	Teleostei	GAculeatus_UGA_version5	Ensembl Release 115
Larimichthys crocea	Actinopterygii	Teleostei	L_crocea_2.0	Ensembl Release 115
Oryzias latipes	Actinopterygii	Teleostei	ASM223467v1	Ensembl Release 115
Fundulus heteroclitus	Actinopterygii	Teleostei (Cyprinodontiformes)	Fundulus_heteroclitus-3.0.2	Ensembl Release 115
Cyprinodon variegatus	Actinopterygii	Teleostei (Cyprinodontiformes)	C_variegatus-1.0	Ensembl Release 115
Kryptolebias marmoratus	Actinopterygii	Teleostei (Cyprinodontiformes)	ASM164957v1	Ensembl Release 115
Nothobranchius furzeri	Actinopterygii	Teleostei (Cyprinodontiformes)	Nfu_20140520	Ensembl Release 115
Poecilia formosa	Actinopterygii	Teleostei (Poeciliidae)	PoeFor_5.1.2	Ensembl Release 115
Poecilia reticulata	Actinopterygii	Teleostei (Poeciliidae)	Guppy_female_1.0_MT	Ensembl Release 115
Poeciliopsis prolifica	Actinopterygii	Teleostei (Poeciliidae)	Auburn_PproV2 (GCF_027474105.1)	NCBI RefSeq
Gambusia affinis	Actinopterygii	Teleostei (Poeciliidae)	SWU_Gaff_1.0 (GCF_019740435.1)	GigaScience [1]
Xiphophorus maculatus	Actinopterygii	Teleostei (Poeciliidae)	X_maculatus-5.0-male	Ensembl Release 115
Xiphophorus	Actinopterygii	Teleostei	Xiphophorus_hellerii-4.1	NCBI

hellerii		(Poeciliidae)	(GCF_003331165.1)	RefSeq
Latimeria chalumnae	Sarcopterygii	Coelacanthiformes	LatCha1	Ensembl Release 115
Protopterus annectens	Sarcopterygii	Dipnoi	PAN1.0 (GCF_019279795.1)	NCBI RefSeq
Xenopus tropicalis	Tetrapoda	Amphibia	UCB_Xtro_10.0	Ensembl Release 115
Anolis carolinensis	Tetrapoda	Reptilia	AnoCar2.0v2	Ensembl Release 115
Gallus gallus	Tetrapoda	Aves	bGalGal1.mat.broiler.GRCg7b	Ensembl Release 115
Mus musculus	Tetrapoda	Mammalia	GRCm39	Ensembl Release 115
Homo sapiens	Tetrapoda	Mammalia	GRCh38.p14	Ensembl Release 115

Note: Proteomes obtained from Ensembl were downloaded on April 26, 2026. Ensembl Release 115 was used for the species indicated in the table, with the BraLan2 annotation for *Branchiostoma lanceolatum* obtained from Ensembl Release 62. Proteomes listed as NCBI RefSeq were obtained from the genome assemblies and accessions specified above. The *Gambusia affinis* proteome was derived from the genome annotation reported by Shao et al. [1].

Table S2. OG0000013 sequences used in Figure 3E.

No.	Tip label	Gene	Species	Clade	Protein accession	Nucleotide accession (CDS)	GeneID	Database	Protein length (aa)
1	Branchiostoma_floridae_LOC118404123	LOC118404123	*Branchiostoma floridae*	Outgroup	XP_035659010.1	XM_035803117.1	LOC118404123	NCBI	561
2	Branchiostoma_floridae_LOC118406062	LOC118406062	*Branchiostoma floridae*	Outgroup	XP_035661811.1	XM_035805918.1	LOC118406062	NCBI	277
3	Branchiostoma_floridae_LOC118409130	LOC118409130	*Branchiostoma floridae*	Outgroup	XP_035665891.1	XM_035809998.1	LOC118409130	NCBI	709
4	Branchiostoma_floridae_LOC118411560	LOC118411560	*Branchiostoma floridae*	Outgroup	XP_035669810.1	XM_035813917.1	LOC118411560	NCBI	361
5	Branchiostoma_floridae_LOC118411832	LOC118411832	*Branchiostoma floridae*	Outgroup	XP_035670214.1	XM_035814321.1	LOC118411832	NCBI	266
6	Branchiostoma_floridae_LOC118412336	LOC118412336	*Branchiostoma floridae*	Outgroup	XP_035671024.1	XM_035815131.1	LOC118412336	NCBI	415
7	Branchiostoma_floridae_LOC118412355	LOC118412355	*Branchiostoma floridae*	Outgroup	XP_035671068.1	XM_035815175.1	LOC118412355	NCBI	763
8	Branchiostoma_floridae_LOC118412358	LOC118412358	*Branchiostoma floridae*	Outgroup	XP_035671071.1	XM_035815178.1	LOC118412358	NCBI	502
9	Branchiostoma_floridae_LOC118412363	LOC118412363	*Branchiostoma*	Outgroup	XP_035671084.1	XM_035815191.1	LOC118412363	NCBI	434

			floridae						
10	Branchiostoma_floridae_LOC118412367	LOC118412367	*Branchiostoma floridae*	Outgroup	XP_035671087.1	XM_035815194.1	LOC118412367	NCBI	402
11	Branchiostoma_floridae_LOC118412368	LOC118412368	*Branchiostoma floridae*	Outgroup	XP_035671088.1	XM_035815195.1	LOC118412368	NCBI	393
12	Branchiostoma_floridae_LOC118412369	LOC118412369	*Branchiostoma floridae*	Outgroup	XP_035671089.1	XM_035815196.1	LOC118412369	NCBI	391
13	Branchiostoma_floridae_LOC118412376	LOC118412376	*Branchiostoma floridae*	Outgroup	XP_035671101.1	XM_035815208.1	LOC118412376	NCBI	311
14	Branchiostoma_floridae_LOC118417671	LOC118417671	*Branchiostoma floridae*	Outgroup	XP_035679207.1	XM_035823314.1	LOC118417671	NCBI	307
15	Branchiostoma_floridae_LOC118420172	LOC118420172	*Branchiostoma floridae*	Outgroup	XP_035682778.1	XM_035826885.1	LOC118420172	NCBI	289
16	Branchiostoma_floridae_LOC118429716	LOC118429716	*Branchiostoma floridae*	Outgroup	XP_035696223.1	XM_035840330.1	LOC118429716	NCBI	281
17	Branchiostoma_floridae_LOC118431979	LOC118431979	*Branchiostoma floridae*	Outgroup	XP_035699330.1	XM_035843437.1	LOC118431979	NCBI	346
18	Branchiostoma_lanceolatum_BL00022	BL00022	*Branchiostoma lanceolatum*	Outgroup	BL00022_evm1	BL00022_evm1	BL00022	Ensembl	400
19	Branchiostoma_lanceolatum_BL00028	BL00028	*Branchiostoma lanceolatum*	Outgroup	BL00028_cuf1	BL00028_cuf1	BL00028	Ensembl	196
20	Branchiostoma_lanceolatum_BL00082	BL00082	*Branchiostoma lanceolatum*	Outgroup	BL00082_cuf1	BL00082_cuf1	BL00082	Ensembl	181
21	Branchiostoma_lanceolatum_BL04692	BL04692	*Branchiostoma*	Outgroup	BL04692_cuf2	BL04692_cuf2	BL04692	Ensembl	230

			lanceolatum						
22	Branchiostoma_lanceolatum_BL07121	BL07121	*Branchiostoma lanceolatum*	Outgroup	BL07121_evm2	BL07121_evm2	BL07121	Ensembl	394
23	Branchiostoma_lanceolatum_BL07494	BL07494	*Branchiostoma lanceolatum*	Outgroup	BL07494_evm0	BL07494_evm0	BL07494	Ensembl	653
24	Branchiostoma_lanceolatum_BL09271	BL09271	*Branchiostoma lanceolatum*	Outgroup	BL09271_evm0	BL09271_evm0	BL09271	Ensembl	395
25	Branchiostoma_lanceolatum_BL13011	BL13011	*Branchiostoma lanceolatum*	Outgroup	BL13011_evm0	BL13011_evm0	BL13011	Ensembl	416
26	Branchiostoma_lanceolatum_BL13663	BL13663	*Branchiostoma lanceolatum*	Outgroup	BL13663_evm0	BL13663_evm0	BL13663	Ensembl	316
27	Branchiostoma_lanceolatum_BL16600	BL16600	*Branchiostoma lanceolatum*	Outgroup	BL16600_evm0	BL16600_evm0	BL16600	Ensembl	555
28	Branchiostoma_lanceolatum_BL23320	BL23320	*Branchiostoma lanceolatum*	Outgroup	BL23320_cuf0	BL23320_cuf0	BL23320	Ensembl	542
29	Branchiostoma_lanceolatum_BL23464	BL23464	*Branchiostoma lanceolatum*	Outgroup	BL23464_evm0	BL23464_evm0	BL23464	Ensembl	116
30	Branchiostoma_lanceolatum_BL30580	BL30580	*Branchiostoma lanceolatum*	Outgroup	BL30580_evm0	BL30580_evm0	BL30580	Ensembl	781
31	Branchiostoma_lanceolatum_BL95919	BL95919	*Branchiostoma*	Outgroup	BL95919_evm1	BL95919_evm1	BL95919	Ensembl	496

			lanceolatum						
32	Branchiostoma_lanceolatum_BL95920	BL95920	*Branchiostoma lanceolatum*	Outgroup	BL95920_evm0	BL95920_evm0	BL95920	Ensembl	475
33	Branchiostoma_lanceolatum_BL96022	BL96022	*Branchiostoma lanceolatum*	Outgroup	BL96022_evm0	BL96022_evm0	BL96022	Ensembl	425
34	Branchiostoma_lanceolatum_BL96938	BL96938	*Branchiostoma lanceolatum*	Outgroup	BL96938_cuf4	BL96938_cuf4	BL96938	Ensembl	262
35	Branchiostoma_lanceolatum_BL97158	BL97158	*Branchiostoma lanceolatum*	Outgroup	BL97158_cuf1	BL97158_cuf1	BL97158	Ensembl	345
36	Ciona_intestinalis_ENSCING00000001370	ENSCING00000001370	*Ciona intestinalis*	Outgroup	ENSCINP00000002671	ENSCINT00000002671	ENSCING00000001370	Ensembl	496
37	Ciona_intestinalis_ENSCING00000001883	ENSCING00000001883	*Ciona intestinalis*	Outgroup	ENSCINP00000003791	ENSCINT00000003791	ENSCING00000001883	Ensembl	519
38	Ciona_intestinalis_ENSCING00000002556	ENSCING00000002556	*Ciona intestinalis*	Outgroup	ENSCINP00000005200	ENSCINT00000005200	ENSCING00000002556	Ensembl	389
39	Ciona_intestinalis_ENSCING00000003037	ENSCING00000003037	*Ciona intestinalis*	Outgroup	ENSCINP00000006180	ENSCINT00000006180	ENSCING00000003037	Ensembl	264
40	Ciona_intestinalis_ENSCING00000003102	ENSCING00000003102	*Ciona intestinalis*	Outgroup	ENSCINP00000025876	ENSCINT00000026122	ENSCING00000003102	Ensembl	263
41	Ciona_intestinalis_ENSCING00000005353	ENSCING00000005353	*Ciona intestinalis*	Outgroup	ENSCINP00000011002	ENSCINT00000011002	ENSCING00000005353	Ensembl	310
42	Ciona_intestinalis_ENSCING00000006812	ENSCING00000006812	*Ciona intestinalis*	Outgroup	ENSCINP00000013976	ENSCINT00000013976	ENSCING00000006812	Ensembl	256
43	Ciona_intestinalis_ENSCING00000007451	ENSCING00000007451	*Ciona intestinalis*	Outgroup	ENSCINP00000015303	ENSCINT00000015303	ENSCING00000007451	Ensembl	397
44	Ciona_intestinalis_ENSCING00000007761	ENSCING00000007761	*Ciona intestinalis*	Outgroup	ENSCINP00000015917	ENSCINT00000015917	ENSCING00000007761	Ensembl	251
45	Ciona_intestinalis_ENSCING00000008363	ENSCING00000008363	*Ciona intestinalis*	Outgroup	ENSCINP00000017059	ENSCINT00000017059	ENSCING00000008363	Ensembl	391

46	Ciona_intestinalis_ENSCING00000009644	ENSCING00000009644	*Ciona intestinalis*	Outgroup	ENSCINP00000019607	ENSCINT00000019607	ENSCING00000009644	Ensembl	251
47	Ciona_intestinalis_ENSCING00000010034	ENSCING00000010034	*Ciona intestinalis*	Outgroup	ENSCINP00000020154	ENSCINT00000020154	ENSCING00000010034	Ensembl	266
48	Ciona_intestinalis_ENSCING00000013000	ENSCING00000013000	*Ciona intestinalis*	Outgroup	ENSCINP00000024034	ENSCINT00000024280	ENSCING00000013000	Ensembl	356
49	Ciona_intestinalis_ENSCING00000013897	ENSCING00000013897	*Ciona intestinalis*	Outgroup	ENSCINP00000025317	ENSCINT00000025563	ENSCING00000013897	Ensembl	375
50	Ciona_intestinalis_ENSCING00000015598	ENSCING00000015598	*Ciona intestinalis*	Outgroup	ENSCINP00000027536	ENSCINT00000027782	ENSCING00000015598	Ensembl	190
51	Ciona_intestinalis_ENSCING00000016432	ENSCING00000016432	*Ciona intestinalis*	Outgroup	ENSCINP00000028475	ENSCINT00000028721	ENSCING00000016432	Ensembl	340
52	Ciona_intestinalis_ENSCING00000018117	ENSCING00000018117	*Ciona intestinalis*	Outgroup	ENSCINP00000035333	ENSCINT00000032729	ENSCING00000018117	Ensembl	283
53	Ciona_intestinalis_ENSCING00000019075	ENSCING00000019075	*Ciona intestinalis*	Outgroup	ENSCINP00000031261	ENSCINT00000030599	ENSCING00000019075	Ensembl	297
54	Ciona_intestinalis_ENSCING00000020357	ENSCING00000020357	*Ciona intestinalis*	Outgroup	ENSCINP00000033597	ENSCINT00000031820	ENSCING00000020357	Ensembl	349
55	Ciona_intestinalis_ENSCING00000020687	ENSCING00000020687	*Ciona intestinalis*	Outgroup	ENSCINP00000030350	ENSCINT00000032308	ENSCING00000020687	Ensembl	165
56	Ciona_intestinalis_ENSCING00000022292	ENSCING00000022292	*Ciona intestinalis*	Outgroup	ENSCINP00000036482	ENSCINT00000032908	ENSCING00000022292	Ensembl	253
57	Ciona_intestinalis_ENSCING00000022780	ENSCING00000022780	*Ciona intestinalis*	Outgroup	ENSCINP00000034632	ENSCINT00000034979	ENSCING00000022780	Ensembl	238
58	Petromyzon_marinus_ENSPMAG00000001180	ENSPMAG00000001180	*Petromyzon marinus*	Outgroup	ENSPMAP00000001309	ENSPMAT00000001315	ENSPMAG00000001180	Ensembl	328
59	Petromyzon_marinus_ENSPMAG00000002621	ENSPMAG00000002621	*Petromyzon marinus*	Outgroup	ENSPMAP00000002855	ENSPMAT00000002869	ENSPMAG00000002621	Ensembl	213
60	Petromyzon_marinus_ENSPMAG00000003338	ENSPMAG00000003338	*Petromyzon marinus*	Outgroup	ENSPMAP00000003633	ENSPMAT00000003648	ENSPMAG00000003338	Ensembl	212
61	Petromyzon_marinus_ENSPMAG00000005045	ENSPMAG00000005045	*Petromyzon marinus*	Outgroup	ENSPMAP00000005522	ENSPMAT00000005542	ENSPMAG00000005045	Ensembl	128
62	Petromyzon_marinus_ENSPMAG00000005051	ENSPMAG00000005051	*Petromyzon marinus*	Outgroup	ENSPMAP00000005527	ENSPMAT00000005547	ENSPMAG00000005051	Ensembl	98
63	Petromyzon_marinus_ENSPMAG00000005545	ENSPMAG00000005545	*Petromyzon marinus*	Outgroup	ENSPMAP00000006099	ENSPMAT00000006128	ENSPMAG00000005545	Ensembl	249

64	Petromyzon_marinus_ENSPMAG 00000006000	ENSPMAG00000 006000	*Petromyzo n marinus*	Outgroup	ENSPMAP000000 06611	ENSPMAT000000 06641	ENSPMAG000000 06000	Ensem bl	235
65	Petromyzon_marinus_ENSPMAG 00000006960	ENSPMAG00000 006960	*Petromyzo n marinus*	Outgroup	ENSPMAP000000 07674	ENSPMAT000000 07708	ENSPMAG000000 06960	Ensem bl	186
66	Petromyzon_marinus_ENSPMAG 00000008628	ENSPMAG00000 008628	*Petromyzo n marinus*	Outgroup	ENSPMAP000000 09500	ENSPMAT000000 09540	ENSPMAG000000 08628	Ensem bl	213
67	Petromyzon_marinus_ENSPMAG 00000008757	ENSPMAG00000 008757	*Petromyzo n marinus*	Outgroup	ENSPMAP000000 09645	ENSPMAT000000 09686	ENSPMAG000000 08757	Ensem bl	215
68	Petromyzon_marinus_ENSPMAG 00000009244	ENSPMAG00000 009244	*Petromyzo n marinus*	Outgroup	ENSPMAP000000 10166	ENSPMAT000000 10211	ENSPMAG000000 09244	Ensem bl	258
69	Petromyzon_marinus_ENSPMAG 00000009480	ENSPMAG00000 009480	*Petromyzo n marinus*	Outgroup	ENSPMAP000000 10421	ENSPMAT000000 10467	ENSPMAG000000 09480	Ensem bl	214
70	Petromyzon_marinus_ENSPMAG 00000009758	ENSPMAG00000 009758	*Petromyzo n marinus*	Outgroup	ENSPMAP000000 10727	ENSPMAT000000 10773	ENSPMAG000000 09758	Ensem bl	220
71	Petromyzon_marinus_ENSPMAG 00000009793	ENSPMAG00000 009793	*Petromyzo n marinus*	Outgroup	ENSPMAP000000 10763	ENSPMAT000000 10809	ENSPMAG000000 09793	Ensem bl	389
72	Callorhinchus_milii_ENSCMIG00 000000497	ENSCMIG00000 000497	*Callorhinc hus milii*	Chondrich thyes	ENSCMIP0000000 0710	ENSCMIT0000000 0758	ENSCMIG0000000 0497	Ensem bl	146
73	Callorhinchus_milii_ENSCMIG00 000000830	ENSCMIG00000 000830	*Callorhinc hus milii*	Chondrich thyes	ENSCMIP0000000 1256	ENSCMIT0000000 1319	ENSCMIG0000000 0830	Ensem bl	835
74	Callorhinchus_milii_ENSCMIG00 000003877	ENSCMIG00000 003877	*Callorhinc hus milii*	Chondrich thyes	ENSCMIP0000000 6929	ENSCMIT0000000 7148	ENSCMIG0000000 3877	Ensem bl	315
75	Callorhinchus_milii_ENSCMIG00 000005143	ENSCMIG00000 005143	*Callorhinc hus milii*	Chondrich thyes	ENSCMIP0000000 9735	ENSCMIT0000000 9996	ENSCMIG0000000 5143	Ensem bl	285
76	Callorhinchus_milii_ENSCMIG00 000006320	ENSCMIG00000 006320	*Callorhinc hus milii*	Chondrich thyes	ENSCMIP0000001 2435	ENSCMIT0000001 2723	ENSCMIG0000000 6320	Ensem bl	343
77	Callorhinchus_milii_ENSCMIG00 000007324	ENSCMIG00000 007324	*Callorhinc hus milii*	Chondrich thyes	ENSCMIP0000001 4935	ENSCMIT0000001 5248	ENSCMIG0000000 7324	Ensem bl	499
78	Callorhinchus_milii_ENSCMIG00 000007497	ENSCMIG00000 007497	*Callorhinc hus milii*	Chondrich thyes	ENSCMIP0000001 5351	ENSCMIT0000001 5670	ENSCMIG0000000 7497	Ensem bl	234
79	Callorhinchus_milii_ENSCMIG00 000010817	ENSCMIG00000 010817	*Callorhinc hus milii*	Chondrich thyes	ENSCMIP0000002 4465	ENSCMIT0000002 4872	ENSCMIG0000001 0817	Ensem bl	268
80	Callorhinchus_milii_ENSCMIG00 000016986	ENSCMIG00000 016986	*Callorhinc hus milii*	Chondrich thyes	ENSCMIP0000004 0881	ENSCMIT0000004 1457	ENSCMIG0000001 6986	Ensem bl	265
81	Callorhinchus_milii_ENSCMIG00 000017426	ENSCMIG00000 017426	*Callorhinc hus milii*	Chondrich thyes	ENSCMIP0000004 1917	ENSCMIT0000004 2515	ENSCMIG0000001 7426	Ensem bl	312

82	Callorhinchus_milii_ENSCMIG00000019681	ENSCMIG00000019681	*Callorhinchus milii*	Chondrichthyes	ENSCMIP00000048119	ENSCMIT00000048793	ENSCMIG00000019681	Ensembl	322
83	Callorhinchus_milii_ENSCMIG00000019693	ENSCMIG00000019693	*Callorhinchus milii*	Chondrichthyes	ENSCMIP00000048157	ENSCMIT00000048831	ENSCMIG00000019693	Ensembl	294
84	Callorhinchus_milii_tmprss11e	*tmprss11e*	*Callorhinchus milii*	Chondrichthyes	ENSCMIP00000040903	ENSCMIT00000041481	ENSCMIG00000017046	Ensembl	457
85	Callorhinchus_milii_tmprss3b	*tmprss3b*	*Callorhinchus milii*	Chondrichthyes	ENSCMIP00000041923	ENSCMIT00000042521	ENSCMIG00000017444	Ensembl	449
86	Scyliorhinus_canicula_LOC119954378	LOC119954378	*Scyliorhinus canicula*	Chondrichthyes	XP_038635503.1	XM_038779575.1	LOC119954378	NCBI	501
87	Scyliorhinus_canicula_LOC119954429	LOC119954429	*Scyliorhinus canicula*	Chondrichthyes	XP_038635566.1	XM_038779638.1	LOC119954429	NCBI	557
88	Scyliorhinus_canicula_LOC119963015	LOC119963015	*Scyliorhinus canicula*	Chondrichthyes	XP_038647399.1	XM_038791471.1	LOC119963015	NCBI	325
89	Scyliorhinus_canicula_LOC119968762	LOC119968762	*Scyliorhinus canicula*	Chondrichthyes	XP_038657383.1	XM_038801455.1	LOC119968762	NCBI	467
90	Scyliorhinus_canicula_LOC119968901	LOC119968901	*Scyliorhinus canicula*	Chondrichthyes	XP_038657800.1	XM_038801872.1	LOC119968901	NCBI	855
91	Scyliorhinus_canicula_LOC119970195	LOC119970195	*Scyliorhinus canicula*	Chondrichthyes	XP_038660368.1	XM_038804440.1	LOC119970195	NCBI	492
92	Scyliorhinus_canicula_LOC119970196	LOC119970196	*Scyliorhinus canicula*	Chondrichthyes	XP_038660371.1	XM_038804443.1	LOC119970196	NCBI	296
93	Scyliorhinus_canicula_LOC119970944	LOC119970944	*Scyliorhinus canicula*	Chondrichthyes	XP_038661983.1	XM_038806055.1	LOC119970944	NCBI	698
94	Scyliorhinus_canicula_LOC119973800	LOC119973800	*Scyliorhinus canicula*	Chondrichthyes	XP_038668153.1	XM_038812225.1	LOC119973800	NCBI	332
95	Scyliorhinus_canicula_LOC119975214	LOC119975214	*Scyliorhinus canicula*	Chondrichthyes	XP_038670763.1	XM_038814835.1	LOC119975214	NCBI	360

96	Scyliorhinus_canicula_tmprss3a	*tmprss3a*	*Scyliorhinus canicula*	Chondrichthyes	XP_038657340.1	XM_038801412.1	tmprss3a	NCBI	480
97	Scyliorhinus_canicula_tmprss9	*tmprss9*	*Scyliorhinus canicula*	Chondrichthyes	XP_038634190.1	XM_038778262.1	tmprss9	NCBI	676
98	Acipenser_ruthenus_LOC117397332	LOC117397332	*Acipenser ruthenus*	Actinopterygii (non-teleost)	XP_034768543.1	XM_034912652.1	LOC117397332	NCBI	468
99	Acipenser_ruthenus_LOC117397378	LOC117397378	*Acipenser ruthenus*	Actinopterygii (non-teleost)	XP_033852059.2	XM_033996168.2	LOC117397378	NCBI	468
100	Acipenser_ruthenus_LOC117398795	LOC117398795	*Acipenser ruthenus*	Actinopterygii (non-teleost)	XP_033853764.1	XM_033997873.2	LOC117398795	NCBI	327
101	Acipenser_ruthenus_LOC117398987	LOC117398987	*Acipenser ruthenus*	Actinopterygii (non-teleost)	XP_034770327.1	XM_034914436.1	LOC117398987	NCBI	205
102	Acipenser_ruthenus_LOC117400941	LOC117400941	*Acipenser ruthenus*	Actinopterygii (non-teleost)	XP_033857193.2	XM_034001302.2	LOC117400941	NCBI	328
103	Acipenser_ruthenus_LOC117401196	LOC117401196	*Acipenser ruthenus*	Actinopterygii (non-teleost)	XP_033857581.2	XM_034001690.2	LOC117401196	NCBI	796
104	Acipenser_ruthenus_LOC117405382	LOC117405382	*Acipenser ruthenus*	Actinopterygii (non-teleost)	XP_034771895.1	XM_034916004.1	LOC117405382	NCBI	901
105	Acipenser_ruthenus_LOC117407223	LOC117407223	*Acipenser ruthenus*	Actinopterygii (non-teleost)	XP_034779514.1	XM_034923623.1	LOC117407223	NCBI	508
106	Acipenser_ruthenus_LOC117414447	LOC117414447	*Acipenser ruthenus*	Actinopterygii (non-teleost)	XP_034774183.1	XM_034918292.1	LOC117414447	NCBI	333
107	Acipenser_ruthenus_LOC117414571	LOC117414571	*Acipenser ruthenus*	Actinopterygii (non-teleost)	XP_033880227.2	XM_034024336.2	LOC117414571	NCBI	668
108	Acipenser_ruthenus_LOC117419380	LOC117419380	*Acipenser ruthenus*	Actinopterygii (non-teleost)	XP_034782372.1	XM_034926481.1	LOC117419380	NCBI	496

109	Acipenser_ruthenus_LOC117419697	LOC117419697	*Acipenser ruthenus*	Actinopterygii (non-teleost)	XP_033888599.1	XM_034032708.1	LOC117419697	NCBI	311
110	Acipenser_ruthenus_LOC117420064	LOC117420064	*Acipenser ruthenus*	Actinopterygii (non-teleost)	XP_034782373.1	XM_034926482.1	LOC117420064	NCBI	276
111	Acipenser_ruthenus_LOC117428824	LOC117428824	*Acipenser ruthenus*	Actinopterygii (non-teleost)	XP_033903669.2	XM_034047778.2	LOC117428824	NCBI	796
112	Acipenser_ruthenus_LOC117429791	LOC117429791	*Acipenser ruthenus*	Actinopterygii (non-teleost)	XP_034763453.1	XM_034907562.1	LOC117429791	NCBI	298
113	Acipenser_ruthenus_LOC117432097	LOC117432097	*Acipenser ruthenus*	Actinopterygii (non-teleost)	XP_033909453.2	XM_034053562.2	LOC117432097	NCBI	1013
114	Acipenser_ruthenus_LOC117962474	LOC117962474	*Acipenser ruthenus*	Actinopterygii (non-teleost)	XP_034778711.1	XM_034922820.1	LOC117962474	NCBI	508
115	Acipenser_ruthenus_LOC117963371	LOC117963371	*Acipenser ruthenus*	Actinopterygii (non-teleost)	XP_034759180.1	XM_034903289.1	LOC117963371	NCBI	289
116	Acipenser_ruthenus_LOC117966639	LOC117966639	*Acipenser ruthenus*	Actinopterygii (non-teleost)	XP_034768976.1	XM_034913085.1	LOC117966639	NCBI	469
117	Acipenser_ruthenus_LOC117967030	LOC117967030	*Acipenser ruthenus*	Actinopterygii (non-teleost)	XP_034770006.1	XM_034914115.1	LOC117967030	NCBI	318
118	Acipenser_ruthenus_LOC117968345	LOC117968345	*Acipenser ruthenus*	Actinopterygii (non-teleost)	XP_034771896.1	XM_034916005.1	LOC117968345	NCBI	258
119	Acipenser_ruthenus_LOC117972708	LOC117972708	*Acipenser ruthenus*	Actinopterygii (non-teleost)	XP_034778588.1	XM_034922697.1	LOC117972708	NCBI	278
120	Acipenser_ruthenus_tmprss3a	*tmprss3a*	*Acipenser ruthenus*	Actinopterygii (non-teleost)	XP_034779126.1	XM_034923235.1	tmprss3a	NCBI	436
121	Lepisosteus_oculatus_ENSLOCG00000000027	ENSLOCG00000000027	*Lepisosteus oculatus*	Actinopterygii (non-teleost)	ENSLOCP00000000031	ENSLOCT00000000031	ENSLOCG00000000027	Ensembl	98

122	Lepisosteus_oculatus_ENSLOCG00000000028	ENSLOCG00000000028	*Lepisosteus oculatus*	Actinopterygii (non-teleost)	ENSLOCP00000000032	ENSLOCT00000000032	ENSLOCG00000000028	Ensembl	349
123	Lepisosteus_oculatus_ENSLOCG00000000030	ENSLOCG00000000030	*Lepisosteus oculatus*	Actinopterygii (non-teleost)	ENSLOCP00000000035	ENSLOCT00000000035	ENSLOCG00000000030	Ensembl	324
124	Lepisosteus_oculatus_ENSLOCG00000000033	ENSLOCG00000000033	*Lepisosteus oculatus*	Actinopterygii (non-teleost)	ENSLOCP00000000037	ENSLOCT00000000037	ENSLOCG00000000033	Ensembl	232
125	Lepisosteus_oculatus_ENSLOCG00000000036	ENSLOCG00000000036	*Lepisosteus oculatus*	Actinopterygii (non-teleost)	ENSLOCP00000000040	ENSLOCT00000000040	ENSLOCG00000000036	Ensembl	327
126	Lepisosteus_oculatus_ENSLOCG00000000038	ENSLOCG00000000038	*Lepisosteus oculatus*	Actinopterygii (non-teleost)	ENSLOCP00000000043	ENSLOCT00000000043	ENSLOCG00000000038	Ensembl	58
127	Lepisosteus_oculatus_ENSLOCG00000000039	ENSLOCG00000000039	*Lepisosteus oculatus*	Actinopterygii (non-teleost)	ENSLOCP00000000045	ENSLOCT00000000045	ENSLOCG00000000039	Ensembl	318
128	Lepisosteus_oculatus_ENSLOCG00000000041	ENSLOCG00000000041	*Lepisosteus oculatus*	Actinopterygii (non-teleost)	ENSLOCP00000000048	ENSLOCT00000000048	ENSLOCG00000000041	Ensembl	525
129	Lepisosteus_oculatus_ENSLOCG00000000042	ENSLOCG00000000042	*Lepisosteus oculatus*	Actinopterygii (non-teleost)	ENSLOCP00000000050	ENSLOCT00000000050	ENSLOCG00000000042	Ensembl	785
130	Lepisosteus_oculatus_ENSLOCG00000000044	ENSLOCG00000000044	*Lepisosteus oculatus*	Actinopterygii (non-teleost)	ENSLOCP00000000052	ENSLOCT00000000052	ENSLOCG00000000044	Ensembl	302
131	Lepisosteus_oculatus_ENSLOCG00000000053	ENSLOCG00000000053	*Lepisosteus oculatus*	Actinopterygii (non-teleost)	ENSLOCP00000000061	ENSLOCT00000000061	ENSLOCG00000000053	Ensembl	313
132	Lepisosteus_oculatus_ENSLOCG00000000054	ENSLOCG00000000054	*Lepisosteus oculatus*	Actinopterygii (non-teleost)	ENSLOCP00000000064	ENSLOCT00000000064	ENSLOCG00000000054	Ensembl	631
133	Lepisosteus_oculatus_ENSLOCG00000000055	ENSLOCG00000000055	*Lepisosteus oculatus*	Actinopterygii (non-teleost)	ENSLOCP00000000065	ENSLOCT00000000065	ENSLOCG00000000055	Ensembl	624
134	Lepisosteus_oculatus_ENSLOCG00000000167	ENSLOCG00000000167	*Lepisosteus oculatus*	Actinopterygii (non-teleost)	ENSLOCP00000000186	ENSLOCT00000000186	ENSLOCG00000000167	Ensembl	190

135	Lepisosteus_oculatus_ENSLOCG00000000213	ENSLOCG00000000213	*Lepisosteus oculatus*	Actinopterygii (non-teleost)	ENSLOCP00000000242	ENSLOCT00000000242	ENSLOCG00000000213	Ensembl	571
136	Lepisosteus_oculatus_ENSLOCG00000000364	ENSLOCG00000000364	*Lepisosteus oculatus*	Actinopterygii (non-teleost)	ENSLOCP00000000413	ENSLOCT00000000413	ENSLOCG00000000364	Ensembl	246
137	Lepisosteus_oculatus_ENSLOCG00000000377	ENSLOCG00000000377	*Lepisosteus oculatus*	Actinopterygii (non-teleost)	ENSLOCP00000000410	ENSLOCT00000000410	ENSLOCG00000000377	Ensembl	264
138	Lepisosteus_oculatus_ENSLOCG00000000732	ENSLOCG00000000732	*Lepisosteus oculatus*	Actinopterygii (non-teleost)	ENSLOCP00000000814	ENSLOCT00000000818	ENSLOCG00000000732	Ensembl	315
139	Lepisosteus_oculatus_ENSLOCG00000000753	ENSLOCG00000000753	*Lepisosteus oculatus*	Actinopterygii (non-teleost)	ENSLOCP00000000839	ENSLOCT00000000843	ENSLOCG00000000753	Ensembl	867
140	Lepisosteus_oculatus_ENSLOCG00000000780	ENSLOCG00000000780	*Lepisosteus oculatus*	Actinopterygii (non-teleost)	ENSLOCP00000000867	ENSLOCT00000000871	ENSLOCG00000000780	Ensembl	285
141	Lepisosteus_oculatus_ENSLOCG00000000985	ENSLOCG00000000985	*Lepisosteus oculatus*	Actinopterygii (non-teleost)	ENSLOCP00000001108	ENSLOCT00000001112	ENSLOCG00000000985	Ensembl	186
142	Lepisosteus_oculatus_ENSLOCG00000001075	ENSLOCG00000001075	*Lepisosteus oculatus*	Actinopterygii (non-teleost)	ENSLOCP00000001210	ENSLOCT00000001214	ENSLOCG00000001075	Ensembl	260
143	Lepisosteus_oculatus_ENSLOCG00000001116	ENSLOCG00000001116	*Lepisosteus oculatus*	Actinopterygii (non-teleost)	ENSLOCP00000001259	ENSLOCT00000001263	ENSLOCG00000001116	Ensembl	296
144	Lepisosteus_oculatus_ENSLOCG00000001135	ENSLOCG00000001135	*Lepisosteus oculatus*	Actinopterygii (non-teleost)	ENSLOCP00000001285	ENSLOCT00000001290	ENSLOCG00000001135	Ensembl	492
145	Lepisosteus_oculatus_ENSLOCG00000001165	ENSLOCG00000001165	*Lepisosteus oculatus*	Actinopterygii (non-teleost)	ENSLOCP00000001315	ENSLOCT00000001320	ENSLOCG00000001165	Ensembl	457
146	Lepisosteus_oculatus_ENSLOCG00000003573	ENSLOCG00000003573	*Lepisosteus oculatus*	Actinopterygii (non-teleost)	ENSLOCP00000004248	ENSLOCT00000004256	ENSLOCG00000003573	Ensembl	281
147	Lepisosteus_oculatus_ENSLOCG00000004255	ENSLOCG00000004255	*Lepisosteus oculatus*	Actinopterygii (non-teleost)	ENSLOCP00000005092	ENSLOCT00000005100	ENSLOCG00000004255	Ensembl	994

148	Lepisosteus_oculatus_ENSLOCG00000004407	ENSLOCG00000004407	*Lepisosteus oculatus*	Actinopterygii (non-teleost)	ENSLOCP00000005271	ENSLOCT00000005279	ENSLOCG00000004407	Ensembl	646
149	Lepisosteus_oculatus_ENSLOCG00000005698	ENSLOCG00000005698	*Lepisosteus oculatus*	Actinopterygii (non-teleost)	ENSLOCP00000006877	ENSLOCT00000006885	ENSLOCG00000005698	Ensembl	175
150	Lepisosteus_oculatus_ENSLOCG00000006813	ENSLOCG00000006813	*Lepisosteus oculatus*	Actinopterygii (non-teleost)	ENSLOCP00000008228	ENSLOCT00000008238	ENSLOCG00000006813	Ensembl	641
151	Lepisosteus_oculatus_TMPRSS13	*TMPRSS13*	*Lepisosteus oculatus*	Actinopterygii (non-teleost)	ENSLOCP00000008107	ENSLOCT00000008117	ENSLOCG00000006710	Ensembl	484
152	Lepisosteus_oculatus_tmprss2	*tmprss2*	*Lepisosteus oculatus*	Actinopterygii (non-teleost)	ENSLOCP00000001585	ENSLOCT00000001590	ENSLOCG00000001392	Ensembl	483
153	Lepisosteus_oculatus_tmprss3b	*tmprss3b*	*Lepisosteus oculatus*	Actinopterygii (non-teleost)	ENSLOCP00000004583	ENSLOCT00000004591	ENSLOCG00000003832	Ensembl	446
154	Lepisosteus_oculatus_TMPRSS5	*TMPRSS5*	*Lepisosteus oculatus*	Actinopterygii (non-teleost)	ENSLOCP00000005335	ENSLOCT00000005343	ENSLOCG00000004438	Ensembl	471
155	Lepisosteus_oculatus_tmprss9	*tmprss9*	*Lepisosteus oculatus*	Actinopterygii (non-teleost)	ENSLOCP00000005159	ENSLOCT00000005167	ENSLOCG00000004321	Ensembl	730
156	Polyodon_spathula_LOC121318214	LOC121318214	*Polyodon spathula*	Actinopterygii (non-teleost)	XP_041110585.1	XM_041254651.1	LOC121318214	NCBI	311
157	Polyodon_spathula_tmprss3a	*tmprss3a*	*Polyodon spathula*	Actinopterygii (non-teleost)	XP_041115893.1	XM_041259959.1	tmprss3a	NCBI	519
158	Latimeria_chalumnae_ENSLACG00000000360	ENSLACG00000000360	*Latimeria chalumnae*	Sarcopterygii	ENSLACP00000000400	ENSLACT00000000402	ENSLACG00000000360	Ensembl	190
159	Latimeria_chalumnae_ENSLACG00000000541	ENSLACG00000000541	*Latimeria chalumnae*	Sarcopterygii	ENSLACP00000000609	ENSLACT00000000612	ENSLACG00000000541	Ensembl	855
160	Latimeria_chalumnae_ENSLACG00000002107	ENSLACG00000002107	*Latimeria chalumnae*	Sarcopterygii	ENSLACP00000002357	ENSLACT00000002377	ENSLACG00000002107	Ensembl	177
161	Latimeria_chalumnae_ENSLACG00000002335	ENSLACG00000002335	*Latimeria chalumnae*	Sarcopterygii	ENSLACP00000002609	ENSLACT00000002630	ENSLACG00000002335	Ensembl	274

162	Latimeria_chalumnae_ENSLACG00000003815	ENSLACG00000003815	*Latimeria chalumnae*	Sarcopterygii	ENSLACP00000004287	ENSLACT00000004325	ENSLACG00000003815	Ensembl	265
163	Latimeria_chalumnae_ENSLACG00000005347	ENSLACG00000005347	*Latimeria chalumnae*	Sarcopterygii	ENSLACP00000006023	ENSLACT00000006075	ENSLACG00000005347	Ensembl	888
164	Latimeria_chalumnae_ENSLACG00000005797	ENSLACG00000005797	*Latimeria chalumnae*	Sarcopterygii	ENSLACP00000006540	ENSLACT00000006593	ENSLACG00000005797	Ensembl	327
165	Latimeria_chalumnae_ENSLACG00000007412	ENSLACG00000007412	*Latimeria chalumnae*	Sarcopterygii	ENSLACP00000008371	ENSLACT00000008439	ENSLACG00000007412	Ensembl	277
166	Latimeria_chalumnae_ENSLACG00000008243	ENSLACG00000008243	*Latimeria chalumnae*	Sarcopterygii	ENSLACP00000009343	ENSLACT00000009414	ENSLACG00000008243	Ensembl	278
167	Latimeria_chalumnae_ENSLACG00000008622	ENSLACG00000008622	*Latimeria chalumnae*	Sarcopterygii	ENSLACP00000009779	ENSLACT00000009854	ENSLACG00000008622	Ensembl	483
168	Latimeria_chalumnae_ENSLACG00000009167	ENSLACG00000009167	*Latimeria chalumnae*	Sarcopterygii	ENSLACP00000010405	ENSLACT00000010484	ENSLACG00000009167	Ensembl	836
169	Latimeria_chalumnae_ENSLACG00000009581	ENSLACG00000009581	*Latimeria chalumnae*	Sarcopterygii	ENSLACP00000010887	ENSLACT00000010967	ENSLACG00000009581	Ensembl	511
170	Latimeria_chalumnae_ENSLACG00000013204	ENSLACG00000013204	*Latimeria chalumnae*	Sarcopterygii	ENSLACP00000015003	ENSLACT00000015108	ENSLACG00000013204	Ensembl	311
171	Latimeria_chalumnae_TMPRSS13B	*TMPRSS13B*	*Latimeria chalumnae*	Sarcopterygii	ENSLACP00000021080	ENSLACT00000021220	ENSLACG00000018519	Ensembl	264
172	Latimeria_chalumnae_TMPRSS2	*TMPRSS2*	*Latimeria chalumnae*	Sarcopterygii	ENSLACP00000017641	ENSLACT00000017771	ENSLACG00000015538	Ensembl	487
173	Latimeria_chalumnae_TMPRSS5	*TMPRSS5*	*Latimeria chalumnae*	Sarcopterygii	ENSLACP00000021099	ENSLACT00000021239	ENSLACG00000018536	Ensembl	399
174	Latimeria_chalumnae_TMPRSS9	*TMPRSS9*	*Latimeria chalumnae*	Sarcopterygii	ENSLACP00000017488	ENSLACT00000017617	ENSLACG00000015402	Ensembl	707
175	Protopterus_annectens_LOC122787486	LOC122787486	*Protopterus annectens*	Sarcopterygii	XP_043910161.1	XM_044054226.1	LOC122787486	NCBI	217
176	Protopterus_annectens_LOC122793158	LOC122793158	*Protopterus annectens*	Sarcopterygii	XP_043916888.1	XM_044060953.1	LOC122793158	NCBI	580
177	Protopterus_annectens_LOC122798578	LOC122798578	*Protopterus annectens*	Sarcopterygii	XP_043923925.1	XM_044067990.1	LOC122798578	NCBI	275
178	Protopterus_annectens_LOC122801279	LOC122801279	*Protopterus*	Sarcopterygii	XP_043926765.1	XM_044070830.1	LOC122801279	NCBI	278

			annectens						
179	Protopterus_annectens_LOC122802743	LOC122802743	*Protopterus annectens*	Sarcopterygii	XP_043928446.1	XM_044072511.1	LOC122802743	NCBI	843
180	Protopterus_annectens_LOC122803970	LOC122803970	*Protopterus annectens*	Sarcopterygii	XP_043929583.1	XM_044073648.1	LOC122803970	NCBI	369
181	Protopterus_annectens_LOC122804094	LOC122804094	*Protopterus annectens*	Sarcopterygii	XP_043929688.1	XM_044073753.1	LOC122804094	NCBI	361
182	Protopterus_annectens_LOC122805304	LOC122805304	*Protopterus annectens*	Sarcopterygii	XP_043931327.1	XM_044075392.1	LOC122805304	NCBI	427
183	Protopterus_annectens_LOC122808402	LOC122808402	*Protopterus annectens*	Sarcopterygii	XP_043935302.1	XM_044079367.1	LOC122808402	NCBI	363
184	Protopterus_annectens_LOC122809435	LOC122809435	*Protopterus annectens*	Sarcopterygii	XP_043936880.1	XM_044080945.1	LOC122809435	NCBI	482
185	Protopterus_annectens_LOC122813740	LOC122813740	*Protopterus annectens*	Sarcopterygii	XP_043942148.1	XM_044086213.1	LOC122813740	NCBI	331
186	Protopterus_annectens_LOC122813743	LOC122813743	*Protopterus annectens*	Sarcopterygii	XP_043942151.1	XM_044086216.1	LOC122813743	NCBI	356
187	Protopterus_annectens_LOC122813744	LOC122813744	*Protopterus annectens*	Sarcopterygii	XP_043942152.1	XM_044086217.1	LOC122813744	NCBI	336
188	Protopterus_annectens_LOC122813829	LOC122813829	*Protopterus annectens*	Sarcopterygii	XP_043942292.1	XM_044086357.1	LOC122813829	NCBI	402
189	Protopterus_annectens_LOC122813830	LOC122813830	*Protopterus annectens*	Sarcopterygii	XP_043942293.1	XM_044086358.1	LOC122813830	NCBI	405
190	Protopterus_annectens_LOC122814339	LOC122814339	*Protopterus annectens*	Sarcopterygii	XP_043942860.1	XM_044086925.1	LOC122814339	NCBI	516
191	Protopterus_annectens_LOC122814355	LOC122814355	*Protopterus*	Sarcopterygii	XP_043942876.1	XM_044086941.1	LOC122814355	NCBI	680

			annectens						
192	Protopterus_annectens_LOC122814356	LOC122814356	*Protopterus annectens*	Sarcopterygii	XP_043942877.1	XM_044086942.1	LOC122814356	NCBI	878
193	Protopterus_annectens_LOC122814357	LOC122814357	*Protopterus annectens*	Sarcopterygii	XP_043942878.1	XM_044086943.1	LOC122814357	NCBI	372
194	Protopterus_annectens_LOC122816486	LOC122816486	*Protopterus annectens*	Sarcopterygii	XP_043945246.1	XM_044089311.1	LOC122816486	NCBI	349
195	Protopterus_annectens_TMPRSS2	*TMPRSS2*	*Protopterus annectens*	Sarcopterygii	XP_043927886.1	XM_044071951.1	TMPRSS2	NCBI	397
196	Protopterus_annectens_TMPRSS5	*TMPRSS5*	*Protopterus annectens*	Sarcopterygii	XP_043937001.1	XM_044081066.1	TMPRSS5	NCBI	464
197	Protopterus_annectens_TMPRSS9	*TMPRSS9*	*Protopterus annectens*	Sarcopterygii	XP_043946556.1	XM_044090621.1	TMPRSS9	NCBI	1010
198	Anolis_carolinensis_ENSACAG00000000041	ENSACAG00000000041	*Anolis carolinensis*	Tetrapoda	ENSACAP00000000040	ENSACAT00000000041	ENSACAG00000000041	Ensembl	1194
199	Anolis_carolinensis_ENSACAG00000006964	ENSACAG00000006964	*Anolis carolinensis*	Tetrapoda	ENSACAP00000006817	ENSACAT00000006967	ENSACAG00000006964	Ensembl	293
200	Anolis_carolinensis_ENSACAG00000006983	ENSACAG00000006983	*Anolis carolinensis*	Tetrapoda	ENSACAP00000006819	ENSACAT00000006969	ENSACAG00000006983	Ensembl	478
201	Anolis_carolinensis_ENSACAG00000006985	ENSACAG00000006985	*Anolis carolinensis*	Tetrapoda	ENSACAP00000006859	ENSACAT00000007009	ENSACAG00000006985	Ensembl	334
202	Anolis_carolinensis_ENSACAG00000007471	ENSACAG00000007471	*Anolis carolinensis*	Tetrapoda	ENSACAP00000030618	ENSACAT00000041812	ENSACAG00000007471	Ensembl	499
203	Anolis_carolinensis_ENSACAG00000007928	ENSACAG00000007928	*Anolis carolinensis*	Tetrapoda	ENSACAP00000007788	ENSACAT00000007954	ENSACAG00000007928	Ensembl	302
204	Anolis_carolinensis_ENSACAG00000008840	ENSACAG00000008840	*Anolis carolinensi*	Tetrapoda	ENSACAP00000008757	ENSACAT00000008945	ENSACAG00000008840	Ensembl	348

			s						
205	Anolis_carolinensis_ENSACAG00000013144	ENSACAG00000013144	*Anolis carolinensis*	Tetrapoda	ENSACAP00000012964	ENSACAT00000013223	ENSACAG00000013144	Ensembl	472
206	Anolis_carolinensis_ENSACAG00000016107	ENSACAG00000016107	*Anolis carolinensis*	Tetrapoda	ENSACAP00000026542	ENSACAT00000039023	ENSACAG00000016107	Ensembl	773
207	Anolis_carolinensis_ENSACAG00000017225	ENSACAG00000017225	*Anolis carolinensis*	Tetrapoda	ENSACAP00000016949	ENSACAT00000017284	ENSACAG00000017225	Ensembl	318
208	Anolis_carolinensis_ENSACAG00000017231	ENSACAG00000017231	*Anolis carolinensis*	Tetrapoda	ENSACAP00000016959	ENSACAT00000017294	ENSACAG00000017231	Ensembl	328
209	Anolis_carolinensis_ENSACAG00000025507	ENSACAG00000025507	*Anolis carolinensis*	Tetrapoda	ENSACAP00000031834	ENSACAT00000051747	ENSACAG00000025507	Ensembl	363
210	Anolis_carolinensis_ENSACAG00000027821	ENSACAG00000027821	*Anolis carolinensis*	Tetrapoda	ENSACAP00000019512	ENSACAT00000024986	ENSACAG00000027821	Ensembl	415
211	Anolis_carolinensis_ENSACAG00000028847	ENSACAG00000028847	*Anolis carolinensis*	Tetrapoda	ENSACAP00000022938	ENSACAT00000030357	ENSACAG00000028847	Ensembl	200
212	Anolis_carolinensis_ENSACAG00000035160	ENSACAG00000035160	*Anolis carolinensis*	Tetrapoda	ENSACAP00000040599	ENSACAT00000056751	ENSACAG00000035160	Ensembl	296
213	Anolis_carolinensis_ENSACAG00000035904	ENSACAG00000035904	*Anolis carolinensis*	Tetrapoda	ENSACAP00000025476	ENSACAT00000053262	ENSACAG00000035904	Ensembl	336
214	Anolis_carolinensis_ENSACAG00000036109	ENSACAG00000036109	*Anolis carolinensis*	Tetrapoda	ENSACAP00000041302	ENSACAT00000055754	ENSACAG00000036109	Ensembl	289
215	Anolis_carolinensis_ENSACAG00000038717	ENSACAG00000038717	*Anolis carolinensis*	Tetrapoda	ENSACAP00000025434	ENSACAT00000056338	ENSACAG00000038717	Ensembl	311
216	Anolis_carolinensis_ENSACAG00000039731	ENSACAG00000039731	*Anolis carolinensis*	Tetrapoda	ENSACAP00000027663	ENSACAT00000056915	ENSACAG00000039731	Ensembl	307
217	Anolis_carolinensis_ENSACAG00000039851	ENSACAG00000039851	*Anolis carolinensi*	Tetrapoda	ENSACAP00000031692	ENSACAT00000042004	ENSACAG00000039851	Ensembl	308

			s						
218	Anolis_carolinensis_ENSACAG00000041218	ENSACAG00000041218	*Anolis carolinensis*	Tetrapoda	ENSACAP00000025804	ENSACAT00000039982	ENSACAG00000041218	Ensembl	206
219	Anolis_carolinensis_ENSACAG00000041333	ENSACAG00000041333	*Anolis carolinensis*	Tetrapoda	ENSACAP00000024165	ENSACAT00000052494	ENSACAG00000041333	Ensembl	305
220	Anolis_carolinensis_ENSACAG00000042194	ENSACAG00000042194	*Anolis carolinensis*	Tetrapoda	ENSACAP00000030468	ENSACAT00000054785	ENSACAG00000042194	Ensembl	350
221	Anolis_carolinensis_ENSACAG00000042661	ENSACAG00000042661	*Anolis carolinensis*	Tetrapoda	ENSACAP00000032328	ENSACAT00000044351	ENSACAG00000042661	Ensembl	330
222	Anolis_carolinensis_ENSACAG00000042903	ENSACAG00000042903	*Anolis carolinensis*	Tetrapoda	ENSACAP00000033993	ENSACAT00000058716	ENSACAG00000042903	Ensembl	230
223	Anolis_carolinensis_ENSACAG00000043452	ENSACAG00000043452	*Anolis carolinensis*	Tetrapoda	ENSACAP00000027824	ENSACAT00000043709	ENSACAG00000043452	Ensembl	344
224	Anolis_carolinensis_OVCH2	*OVCH2*	*Anolis carolinensis*	Tetrapoda	ENSACAP00000037587	ENSACAT00000051790	ENSACAG00000004288	Ensembl	843
225	Anolis_carolinensis_prss8	*prss8*	*Anolis carolinensis*	Tetrapoda	ENSACAP00000006611	ENSACAT00000006757	ENSACAG00000006748	Ensembl	368
226	Anolis_carolinensis_TMPRSS2	*TMPRSS2*	*Anolis carolinensis*	Tetrapoda	ENSACAP00000005307	ENSACAT00000005424	ENSACAG00000005446	Ensembl	486
227	Anolis_carolinensis_TMPRSS3	*TMPRSS3*	*Anolis carolinensis*	Tetrapoda	ENSACAP00000032708	ENSACAT00000037614	ENSACAG00000003022	Ensembl	417
228	Gallus_gallus_ACR	*ACR*	*Gallus gallus*	Tetrapoda	ENSGALP00010009779	ENSGALT00010017687	ENSGALG00010007422	Ensembl	354
229	Gallus_gallus_ACRL	*ACRL*	*Gallus gallus*	Tetrapoda	ENSGALP00010009783	ENSGALT00010017699	ENSGALG00010007427	Ensembl	354
230	Gallus_gallus_ENSGALG00010004460	ENSGALG00010004460	*Gallus gallus*	Tetrapoda	ENSGALP00010006075	ENSGALT00010010411	ENSGALG00010004460	Ensembl	782
231	Gallus_gallus_OVCH1	*OVCH1*	*Gallus*	Tetrapoda	ENSGALP0001003	ENSGALT000100	ENSGALG000100	Ensem	863

			gallus		1311	52393	21584	bl	
232	Gallus_gallus_PRSS55	*PRSS55*	*Gallus gallus*	Tetrapoda	ENSGALP00010015200	ENSGALT00010026649	ENSGALG00010011145	Ensembl	376
233	Gallus_gallus_TMPRSS11D	*TMPRSS11D*	*Gallus gallus*	Tetrapoda	ENSGALP00010006443	ENSGALT00010011471	ENSGALG00010004903	Ensembl	478
234	Gallus_gallus_TMPRSS11F	*TMPRSS11F*	*Gallus gallus*	Tetrapoda	ENSGALP00010006458	ENSGALT00010011526	ENSGALG00010004924	Ensembl	451
235	Gallus_gallus_TMPRSS12	*TMPRSS12*	*Gallus gallus*	Tetrapoda	ENSGALP00010032734	ENSGALT00010054225	ENSGALG00010022287	Ensembl	318
236	Gallus_gallus_TMPRSS13	*TMPRSS13*	*Gallus gallus*	Tetrapoda	ENSGALP00010033540	ENSGALT00010055389	ENSGALG00010022782	Ensembl	502
237	Gallus_gallus_TMPRSS2	*TMPRSS2*	*Gallus gallus*	Tetrapoda	ENSGALP00010019622	ENSGALT00010033210	ENSGALG00010013833	Ensembl	499
238	Gallus_gallus_TMPRSS3	*TMPRSS3*	*Gallus gallus*	Tetrapoda	ENSGALP00010015173	ENSGALT00010026616	ENSGALG00010011125	Ensembl	476
239	Gallus_gallus_TMPRSS5	*TMPRSS5*	*Gallus gallus*	Tetrapoda	ENSGALP00010035168	ENSGALT00010057977	ENSGALG00010023782	Ensembl	425
240	Gallus_gallus_TMPRSS9	*TMPRSS9*	*Gallus gallus*	Tetrapoda	ENSGALP00010042222	ENSGALT00010068710	ENSGALG00010028362	Ensembl	1127
241	Homo_sapiens_ACR	*ACR*	*Homo sapiens*	Tetrapoda	ENSP00000216139	ENST00000216139	ENSG00000100312	Ensembl	421
242	Homo_sapiens_OVCH1	*OVCH1*	*Homo sapiens*	Tetrapoda	ENSP00000326708	ENST00000318184	ENSG00000187950	Ensembl	1134
243	Homo_sapiens_OVCH2	*OVCH2*	*Homo sapiens*	Tetrapoda	ENSP00000484497	ENST00000533663	ENSG00000183378	Ensembl	565
244	Homo_sapiens_PRSS21	*PRSS21*	*Homo sapiens*	Tetrapoda	ENSP00000613092	ENST00000943033	ENSG00000007038	Ensembl	319
245	Homo_sapiens_PRSS22	*PRSS22*	*Homo sapiens*	Tetrapoda	ENSP00000544783	ENST00000874724	ENSG00000005001	Ensembl	511
246	Homo_sapiens_PRSS27	*PRSS27*	*Homo sapiens*	Tetrapoda	ENSP00000306390	ENST00000302641	ENSG00000172382	Ensembl	290
247	Homo_sapiens_PRSS33	*PRSS33*	*Homo sapiens*	Tetrapoda	ENSP00000625898	ENST00000955839	ENSG00000103355	Ensembl	280
248	Homo_sapiens_PRSS41	*PRSS41*	*Homo sapiens*	Tetrapoda	ENSP00000501492	ENST00000674256	ENSG00000215148	Ensembl	334
249	Homo_sapiens_PRSS55	*PRSS55*	*Homo*	Tetrapoda	ENSP00000333003	ENST00000328655	ENSG0000018464	Ensem	352

			sapiens				7	bl	
250	Homo_sapiens_PRSS8	*PRSS8*	*Homo sapiens*	Tetrapoda	ENSP00000319730	ENST00000317508	ENSG0000005234 4	Ensem bl	343
251	Homo_sapiens_TMPRSS11A	*TMPRSS11A*	*Homo sapiens*	Tetrapoda	ENSP00000334611	ENST00000334830	ENSG0000018705 4	Ensem bl	421
252	Homo_sapiens_TMPRSS11B	*TMPRSS11B*	*Homo sapiens*	Tetrapoda	ENSP00000330475	ENST00000332644	ENSG0000018587 3	Ensem bl	416
253	Homo_sapiens_TMPRSS11D	*TMPRSS11D*	*Homo sapiens*	Tetrapoda	ENSP00000283916	ENST00000283916	ENSG0000015380 2	Ensem bl	418
254	Homo_sapiens_TMPRSS11E	*TMPRSS11E*	*Homo sapiens*	Tetrapoda	ENSP00000307519	ENST00000305363	ENSG0000008712 8	Ensem bl	423
255	Homo_sapiens_TMPRSS11F	*TMPRSS11F*	*Homo sapiens*	Tetrapoda	ENSP00000348639	ENST00000356291	ENSG0000019809 2	Ensem bl	438
256	Homo_sapiens_TMPRSS12	*TMPRSS12*	*Homo sapiens*	Tetrapoda	ENSP00000381476	ENST00000398458	ENSG0000018645 2	Ensem bl	348
257	Homo_sapiens_TMPRSS13	*TMPRSS13*	*Homo sapiens*	Tetrapoda	ENSP00000394114	ENST00000445164	ENSG0000013774 7	Ensem bl	586
258	Homo_sapiens_TMPRSS2	*TMPRSS2*	*Homo sapiens*	Tetrapoda	ENSP00000504602	ENST00000679263	ENSG0000018401 2	Ensem bl	545
259	Homo_sapiens_TMPRSS3	*TMPRSS3*	*Homo sapiens*	Tetrapoda	ENSP00000566690	ENST00000896631	ENSG0000016018 3	Ensem bl	455
260	Homo_sapiens_TMPRSS5	*TMPRSS5*	*Homo sapiens*	Tetrapoda	ENSP00000299882	ENST00000299882	ENSG0000016668 2	Ensem bl	457
261	Homo_sapiens_TMPRSS9	*TMPRSS9*	*Homo sapiens*	Tetrapoda	ENSP00000498031	ENST00000648592	ENSG0000017829 7	Ensem bl	1093
262	Homo_sapiens_TPSAB1	*TPSAB1*	*Homo sapiens*	Tetrapoda	ENSP00000418247	ENST00000461509	ENSG0000017223 6	Ensem bl	282
263	Homo_sapiens_TPSB2	*TPSB2*	*Homo sapiens*	Tetrapoda	ENSP00000484461	ENST00000611196	ENSG0000019725 3	Ensem bl	292
264	Homo_sapiens_TPSD1	*TPSD1*	*Homo sapiens*	Tetrapoda	ENSP00000211076	ENST00000211076	ENSG0000009591 7	Ensem bl	242
265	Homo_sapiens_TPSG1	*TPSG1*	*Homo sapiens*	Tetrapoda	ENSP00000234798	ENST00000234798	ENSG0000011617 6	Ensem bl	321
266	Mus_musculus_Acr	*Acr*	*Mus musculus*	Tetrapoda	ENSMUSP000000 23295	ENSMUST000000 23295	ENSMUSG000000 22622	Ensem bl	436
267	Mus_musculus_Ovch2	*Ovch2*	*Mus*	Tetrapoda	ENSMUSP000001	ENSMUST000001	ENSMUSG000000	Ensem	609

			musculus		02366	06755	48236	bl	
268	Mus_musculus_Prss21	*Prss21*	*Mus musculus*	Tetrapoda	ENSMUSP00000024928	ENSMUST00000024928	ENSMUSG00000024116	Ensembl	324
269	Mus_musculus_Prss22	*Prss22*	*Mus musculus*	Tetrapoda	ENSMUSP00000039808	ENSMUST00000041649	ENSMUSG00000045027	Ensembl	307
270	Mus_musculus_Prss27	*Prss27*	*Mus musculus*	Tetrapoda	ENSMUSP00000056483	ENSMUST00000059482	ENSMUSG00000050762	Ensembl	328
271	Mus_musculus_Prss28	*Prss28*	*Mus musculus*	Tetrapoda	ENSMUSP00000015267	ENSMUST00000015267	ENSMUSG00000024171	Ensembl	274
272	Mus_musculus_Prss29	*Prss29*	*Mus musculus*	Tetrapoda	ENSMUSP00000024993	ENSMUST00000024993	ENSMUSG00000034039	Ensembl	279
273	Mus_musculus_Prss30	*Prss30*	*Mus musculus*	Tetrapoda	ENSMUSP00000157077	ENSMUST00000234765	ENSMUSG00000024124	Ensembl	310
274	Mus_musculus_Prss32	*Prss32*	*Mus musculus*	Tetrapoda	ENSMUSP00000050389	ENSMUST00000061725	ENSMUSG00000048992	Ensembl	334
275	Mus_musculus_Prss33	*Prss33*	*Mus musculus*	Tetrapoda	ENSMUSP00000059491	ENSMUST00000059906	ENSMUSG00000049620	Ensembl	277
276	Mus_musculus_Prss34	*Prss34*	*Mus musculus*	Tetrapoda	ENSMUSP00000045672	ENSMUST00000037453	ENSMUSG00000056399	Ensembl	318
277	Mus_musculus_Prss41	*Prss41*	*Mus musculus*	Tetrapoda	ENSMUSP00000024926	ENSMUST00000024926	ENSMUSG00000024114	Ensembl	322
278	Mus_musculus_Prss47	*Prss47*	*Mus musculus*	Tetrapoda	ENSMUSP00000145196	ENSMUST00000203968	ENSMUSG00000090658	Ensembl	395
279	Mus_musculus_Prss52	*Prss52*	*Mus musculus*	Tetrapoda	ENSMUSP00000022537	ENSMUST00000022537	ENSMUSG00000021966	Ensembl	321
280	Mus_musculus_Prss55	*Prss55*	*Mus musculus*	Tetrapoda	ENSMUSP00000086752	ENSMUST00000089338	ENSMUSG00000034623	Ensembl	321
281	Mus_musculus_Prss8	*Prss8*	*Mus musculus*	Tetrapoda	ENSMUSP00000032988	ENSMUST00000032988	ENSMUSG00000030800	Ensembl	339
282	Mus_musculus_Tmprss11a	*Tmprss11a*	*Mus musculus*	Tetrapoda	ENSMUSP00000098634	ENSMUST00000101073	ENSMUSG00000072845	Ensembl	389
283	Mus_musculus_Tmprss11b	*Tmprss11b*	*Mus musculus*	Tetrapoda	ENSMUSP00000042406	ENSMUST00000038448	ENSMUSG00000035861	Ensembl	416
284	Mus_musculus_Tmprss11c	*Tmprss11c*	*Mus musculus*	Tetrapoda	ENSMUSP00000062915	ENSMUST00000059424	ENSMUSG00000061184	Ensembl	431
285	Mus_musculus_Tmprss11d	*Tmprss11d*	*Mus*	Tetrapoda	ENSMUSP000000	ENSMUST000000	ENSMUSG000000	Ensem	417

			musculus		31175	31175	61259	bl	
286	Mus_musculus_Tmprss11e	*Tmprss11e*	*Mus musculus*	Tetrapoda	ENSMUSP00000124534	ENSMUST00000161306	ENSMUSG00000054537	Ensembl	423
287	Mus_musculus_Tmprss11f	*Tmprss11f*	*Mus musculus*	Tetrapoda	ENSMUSP00000112252	ENSMUST00000116553	ENSMUSG00000048764	Ensembl	439
288	Mus_musculus_Tmprss11g	*Tmprss11g*	*Mus musculus*	Tetrapoda	ENSMUSP00000122709	ENSMUST00000134179	ENSMUSG00000079451	Ensembl	417
289	Mus_musculus_Tmprss12	*Tmprss12*	*Mus musculus*	Tetrapoda	ENSMUSP00000093914	ENSMUST00000096200	ENSMUSG00000045631	Ensembl	336
290	Mus_musculus_Tmprss13	*Tmprss13*	*Mus musculus*	Tetrapoda	ENSMUSP00000034597	ENSMUST00000034597	ENSMUSG00000037129	Ensembl	548
291	Mus_musculus_Tmprss2	*Tmprss2*	*Mus musculus*	Tetrapoda	ENSMUSP00000000395	ENSMUST00000000395	ENSMUSG00000000385	Ensembl	490
292	Mus_musculus_Tmprss3	*Tmprss3*	*Mus musculus*	Tetrapoda	ENSMUSP00000110196	ENSMUST00000114549	ENSMUSG00000024034	Ensembl	475
293	Mus_musculus_Tmprss5	*Tmprss5*	*Mus musculus*	Tetrapoda	ENSMUSP00000132181	ENSMUST00000165088	ENSMUSG00000032268	Ensembl	455
294	Mus_musculus_Tmprss9	*Tmprss9*	*Mus musculus*	Tetrapoda	ENSMUSP00000100970	ENSMUST00000105333	ENSMUSG00000059406	Ensembl	1099
295	Mus_musculus_Tpsab1	*Tpsab1*	*Mus musculus*	Tetrapoda	ENSMUSP00000120741	ENSMUST00000153118	ENSMUSG00000024173	Ensembl	310
296	Mus_musculus_Tpsb2	*Tpsb2*	*Mus musculus*	Tetrapoda	ENSMUSP00000159899	ENSMUST00000249732	ENSMUSG00000033825	Ensembl	276
297	Mus_musculus_Tpsg1	*Tpsg1*	*Mus musculus*	Tetrapoda	ENSMUSP00000024999	ENSMUST00000024999	ENSMUSG00000033200	Ensembl	311
298	Xenopus_tropicalis_acr	*acr*	*Xenopus tropicalis*	Tetrapoda	ENSXETP00000066553	ENSXETT00000076375	ENSXETG00000017056	Ensembl	411
299	Xenopus_tropicalis_ENSXETG00000001676	ENSXETG00000001676	*Xenopus tropicalis*	Tetrapoda	ENSXETP00000003529	ENSXETT00000003529	ENSXETG00000001676	Ensembl	388
300	Xenopus_tropicalis_ENSXETG00000006281	ENSXETG00000006281	*Xenopus tropicalis*	Tetrapoda	ENSXETP00000013755	ENSXETT00000013755	ENSXETG00000006281	Ensembl	321
301	Xenopus_tropicalis_ENSXETG00000008526	ENSXETG00000008526	*Xenopus tropicalis*	Tetrapoda	ENSXETP00000096967	ENSXETT00000080290	ENSXETG00000008526	Ensembl	302
302	Xenopus_tropicalis_ENSXETG00000023578	ENSXETG00000023578	*Xenopus tropicalis*	Tetrapoda	ENSXETP00000050888	ENSXETT00000050888	ENSXETG00000023578	Ensembl	329
303	Xenopus_tropicalis_ENSXETG00	ENSXETG00000	*Xenopus*	Tetrapoda	ENSXETP0000010	ENSXETT0000010	ENSXETG000000	Ensem	382

	000025963	025963	*tropicalis*		4775	8525	25963	bl	
304	Xenopus_tropicalis_ENSXETG00 000025971	ENSXETG00000 025971	*Xenopus tropicalis*	Tetrapoda	ENSXETP0000008 3175	ENSXETT0000007 1915	ENSXETG000000 25971	Ensem bl	294
305	Xenopus_tropicalis_ENSXETG00 000032140	ENSXETG00000 032140	*Xenopus tropicalis*	Tetrapoda	ENSXETP0000010 1668	ENSXETT0000007 3744	ENSXETG000000 32140	Ensem bl	403
306	Xenopus_tropicalis_ENSXETG00 000032868	ENSXETG00000 032868	*Xenopus tropicalis*	Tetrapoda	ENSXETP0000006 0032	ENSXETT0000006 5053	ENSXETG000000 32868	Ensem bl	319
307	Xenopus_tropicalis_ENSXETG00 000033327	ENSXETG00000 033327	*Xenopus tropicalis*	Tetrapoda	ENSXETP0000001 8654	ENSXETT0000001 8654	ENSXETG000000 33327	Ensem bl	313
308	Xenopus_tropicalis_ENSXETG00 000034333	ENSXETG00000 034333	*Xenopus tropicalis*	Tetrapoda	ENSXETP0000011 9101	ENSXETT0000011 5924	ENSXETG000000 34333	Ensem bl	365
309	Xenopus_tropicalis_ENSXETG00 000034543	ENSXETG00000 034543	*Xenopus tropicalis*	Tetrapoda	ENSXETP0000006 8057	ENSXETT0000010 1292	ENSXETG000000 34543	Ensem bl	338
310	Xenopus_tropicalis_ENSXETG00 000035063	ENSXETG00000 035063	*Xenopus tropicalis*	Tetrapoda	ENSXETP0000009 1832	ENSXETT0000006 7577	ENSXETG000000 35063	Ensem bl	180
311	Xenopus_tropicalis_ENSXETG00 000035324	ENSXETG00000 035324	*Xenopus tropicalis*	Tetrapoda	ENSXETP0000007 2097	ENSXETT0000010 3055	ENSXETG000000 35324	Ensem bl	187
312	Xenopus_tropicalis_ENSXETG00 000036229	ENSXETG00000 036229	*Xenopus tropicalis*	Tetrapoda	ENSXETP0000008 8999	ENSXETT0000008 8659	ENSXETG000000 36229	Ensem bl	355
313	Xenopus_tropicalis_ENSXETG00 000037718	ENSXETG00000 037718	*Xenopus tropicalis*	Tetrapoda	ENSXETP0000007 7765	ENSXETT0000009 2764	ENSXETG000000 37718	Ensem bl	362
314	Xenopus_tropicalis_ENSXETG00 000037722	ENSXETG00000 037722	*Xenopus tropicalis*	Tetrapoda	ENSXETP0000008 5079	ENSXETT0000007 5395	ENSXETG000000 37722	Ensem bl	380
315	Xenopus_tropicalis_ENSXETG00 000040653	ENSXETG00000 040653	*Xenopus tropicalis*	Tetrapoda	ENSXETP0000008 1871	ENSXETT0000009 7789	ENSXETG000000 40653	Ensem bl	323
316	Xenopus_tropicalis_ENSXETG00 000042799	ENSXETG00000 042799	*Xenopus tropicalis*	Tetrapoda	ENSXETP0000011 3569	ENSXETT0000012 3654	ENSXETG000000 42799	Ensem bl	247
317	Xenopus_tropicalis_ENSXETG00 000043280	ENSXETG00000 043280	*Xenopus tropicalis*	Tetrapoda	ENSXETP0000007 7802	ENSXETT0000008 9415	ENSXETG000000 43280	Ensem bl	242
318	Xenopus_tropicalis_ENSXETG00 000045030	ENSXETG00000 045030	*Xenopus tropicalis*	Tetrapoda	ENSXETP0000010 5460	ENSXETT0000012 4174	ENSXETG000000 45030	Ensem bl	384
319	Xenopus_tropicalis_ENSXETG00 000047235	ENSXETG00000 047235	*Xenopus tropicalis*	Tetrapoda	ENSXETP0000000 3527	ENSXETT0000000 3527	ENSXETG000000 47235	Ensem bl	396
320	Xenopus_tropicalis_ENSXETG00 000048337	ENSXETG00000 048337	*Xenopus tropicalis*	Tetrapoda	ENSXETP0000011 1537	ENSXETT0000011 2802	ENSXETG000000 48337	Ensem bl	314
321	Xenopus_tropicalis_ENSXETG00	ENSXETG00000	*Xenopus*	Tetrapoda	ENSXETP0000011	ENSXETT0000012	ENSXETG000000	Ensem	341

	000049110	049110	*tropicalis*		7967	1654	49110	bl	
322	Xenopus_tropicalis_ovch1	*ovch1*	*Xenopus tropicalis*	Tetrapoda	ENSXETP00000084616	ENSXETT00000092963	ENSXETG00000000325	Ensembl	1514
323	Xenopus_tropicalis_ovch2	*ovch2*	*Xenopus tropicalis*	Tetrapoda	ENSXETP00000019838	ENSXETT00000019838	ENSXETG00000009052	Ensembl	944
324	Xenopus_tropicalis_prss27	*prss27*	*Xenopus tropicalis*	Tetrapoda	ENSXETP00000096330	ENSXETT00000083667	ENSXETG00000006296	Ensembl	336
325	Xenopus_tropicalis_prss29	*prss29*	*Xenopus tropicalis*	Tetrapoda	ENSXETP00000013813	ENSXETT00000013813	ENSXETG00000006302	Ensembl	330
326	Xenopus_tropicalis_prss36	*prss36*	*Xenopus tropicalis*	Tetrapoda	ENSXETP00000068975	ENSXETT00000088388	ENSXETG00000006294	Ensembl	719
327	Xenopus_tropicalis_prss36_2	*prss36*	*Xenopus tropicalis*	Tetrapoda	ENSXETP00000099001	ENSXETT00000094371	ENSXETG00000006297	Ensembl	333
328	Xenopus_tropicalis_prss8	*prss8*	*Xenopus tropicalis*	Tetrapoda	ENSXETP00000058126	ENSXETT00000058126	ENSXETG00000027998	Ensembl	329
329	Xenopus_tropicalis_prss8l.5	*prss8l.5*	*Xenopus tropicalis*	Tetrapoda	ENSXETP00000013782	ENSXETT00000013782	ENSXETG00000006287	Ensembl	353
330	Xenopus_tropicalis_tmprss11f	*tmprss11f*	*Xenopus tropicalis*	Tetrapoda	ENSXETP00000038951	ENSXETT00000038951	ENSXETG00000017962	Ensembl	427
331	Xenopus_tropicalis_tmprss12	*tmprss12*	*Xenopus tropicalis*	Tetrapoda	ENSXETP00000015877	ENSXETT00000015877	ENSXETG00000007300	Ensembl	324
332	Xenopus_tropicalis_tmprss13	*tmprss13*	*Xenopus tropicalis*	Tetrapoda	ENSXETP00000022322	ENSXETT00000022322	ENSXETG00000010125	Ensembl	462
333	Xenopus_tropicalis_tmprss2	*tmprss2*	*Xenopus tropicalis*	Tetrapoda	ENSXETP00000082514	ENSXETT00000085598	ENSXETG00000040648	Ensembl	482
334	Xenopus_tropicalis_tmprss2.12	*tmprss2.12*	*Xenopus tropicalis*	Tetrapoda	ENSXETP00000000109	ENSXETT00000000109	ENSXETG00000000063	Ensembl	497
335	Xenopus_tropicalis_tmprss2.13	*tmprss2.13*	*Xenopus tropicalis*	Tetrapoda	ENSXETP00000107898	ENSXETT00000115329	ENSXETG00000046938	Ensembl	461
336	Xenopus_tropicalis_tmprss2.15	*tmprss2.15*	*Xenopus tropicalis*	Tetrapoda	ENSXETP00000093035	ENSXETT00000103545	ENSXETG00000031788	Ensembl	504
337	Xenopus_tropicalis_TMPRSS3	*TMPRSS3*	*Xenopus tropicalis*	Tetrapoda	ENSXETP00000060870	ENSXETT00000065222	ENSXETG00000030492	Ensembl	487
338	Xenopus_tropicalis_tmprss5	*tmprss5*	*Xenopus tropicalis*	Tetrapoda	ENSXETP00000033129	ENSXETT00000033129	ENSXETG00000015131	Ensembl	464
339	Xenopus_tropicalis_tmprss9	*tmprss9*	*Xenopus*	Tetrapoda	ENSXETP0000000	ENSXETT0000000	ENSXETG000000	Ensem	1151

			tropicalis		3741	3741	01793	bl	
340	Xenopus_tropicalis_tpsb2	*tpsb2*	*Xenopus tropicalis*	Tetrapoda	ENSXETP00000119450	ENSXETT00000109805	ENSXETG00000042697	Ensembl	307
341	Xenopus_tropicalis_XB5723326	XB5723326	*Xenopus tropicalis*	Tetrapoda	ENSXETP00000051257	ENSXETT00000051257	ENSXETG00000023763	Ensembl	379
342	Xenopus_tropicalis_XB5892359	XB5892359	*Xenopus tropicalis*	Tetrapoda	ENSXETP00000013059	ENSXETT00000013059	ENSXETG00000005939	Ensembl	426
343	Cyprinodon_variegatus_ENSCVAG00000000936	ENSCVAG00000000936	*Cyprinodon variegatus*	Teleostei	ENSCVAP00000028020	ENSCVAT00000019627	ENSCVAG00000000936	Ensembl	257
344	Cyprinodon_variegatus_ENSCVAG00000004810	ENSCVAG00000004810	*Cyprinodon variegatus*	Teleostei	ENSCVAP00000022194	ENSCVAT00000009887	ENSCVAG00000004810	Ensembl	301
345	Cyprinodon_variegatus_ENSCVAG00000004826	ENSCVAG00000004826	*Cyprinodon variegatus*	Teleostei	ENSCVAP00000022270	ENSCVAT00000009714	ENSCVAG00000004826	Ensembl	352
346	Cyprinodon_variegatus_ENSCVAG00000004837	ENSCVAG00000004837	*Cyprinodon variegatus*	Teleostei	ENSCVAP00000003636	ENSCVAT00000009702	ENSCVAG00000004837	Ensembl	330
347	Cyprinodon_variegatus_ENSCVAG00000005363	ENSCVAG00000005363	*Cyprinodon variegatus*	Teleostei	ENSCVAP00000004078	ENSCVAT00000008965	ENSCVAG00000005363	Ensembl	252
348	Cyprinodon_variegatus_ENSCVAG00000005580	ENSCVAG00000005580	*Cyprinodon variegatus*	Teleostei	ENSCVAP00000022710	ENSCVAT00000008616	ENSCVAG00000005580	Ensembl	278
349	Cyprinodon_variegatus_ENSCVAG00000005732	ENSCVAG00000005732	*Cyprinodon variegatus*	Teleostei	ENSCVAP00000004175	ENSCVAT00000008793	ENSCVAG00000005732	Ensembl	361
350	Cyprinodon_variegatus_ENSCVAG00000005812	ENSCVAG00000005812	*Cyprinodon variegatus*	Teleostei	ENSCVAP00000022843	ENSCVAT00000008299	ENSCVAG00000005812	Ensembl	306
351	Cyprinodon_variegatus_ENSCVAG00000005944	ENSCVAG00000005944	*Cyprinodon variegatus*	Teleostei	ENSCVAP00000004610	ENSCVAT00000008065	ENSCVAG00000005944	Ensembl	305
352	Cyprinodon_variegatus_ENSCVAG00000008621	ENSCVAG00000008621	*Cyprinodon variegatus*	Teleostei	ENSCVAP00000024506	ENSCVAT00000004014	ENSCVAG00000008621	Ensembl	585
353	Cyprinodon_variegatus_ENSCVA	ENSCVAG00000	*Cyprinodon*	Teleostei	ENSCVAP000000	ENSCVAT000000	ENSCVAG000000	Ensem	301

	G00000014774	014774	*variegatus*		12257	19466	14774	bl	
354	Cyprinodon_variegatus_ENSCVA G00000014911	ENSCVAG00000 014911	*Cyprinodo n variegatus*	Teleostei	ENSCVAP000000 12488	ENSCVAT000000 30351	ENSCVAG000000 14911	Ensem bl	652
355	Cyprinodon_variegatus_ENSCVA G00000014930	ENSCVAG00000 014930	*Cyprinodo n variegatus*	Teleostei	ENSCVAP000000 28092	ENSCVAT000000 19759	ENSCVAG000000 14930	Ensem bl	309
356	Cyprinodon_variegatus_ENSCVA G00000015446	ENSCVAG00000 015446	*Cyprinodo n variegatus*	Teleostei	ENSCVAP000000 12978	ENSCVAT000000 20374	ENSCVAG000000 15446	Ensem bl	197
357	Cyprinodon_variegatus_ENSCVA G00000015833	ENSCVAG00000 015833	*Cyprinodo n variegatus*	Teleostei	ENSCVAP000000 13347	ENSCVAT000000 20854	ENSCVAG000000 15833	Ensem bl	256
358	Cyprinodon_variegatus_ENSCVA G00000018249	ENSCVAG00000 018249	*Cyprinodo n variegatus*	Teleostei	ENSCVAP000000 15431	ENSCVAT000000 23530	ENSCVAG000000 18249	Ensem bl	288
359	Cyprinodon_variegatus_ENSCVA G00000018359	ENSCVAG00000 018359	*Cyprinodo n variegatus*	Teleostei	ENSCVAP000000 15525	ENSCVAT000000 23638	ENSCVAG000000 18359	Ensem bl	298
360	Cyprinodon_variegatus_ENSCVA G00000018374	ENSCVAG00000 018374	*Cyprinodo n variegatus*	Teleostei	ENSCVAP000000 15554	ENSCVAT000000 23658	ENSCVAG000000 18374	Ensem bl	309
361	Cyprinodon_variegatus_ENSCVA G00000019973	ENSCVAG00000 019973	*Cyprinodo n variegatus*	Teleostei	ENSCVAP000000 31016	ENSCVAT000000 25432	ENSCVAG000000 19973	Ensem bl	454
362	Cyprinodon_variegatus_ENSCVA G00000020701	ENSCVAG00000 020701	*Cyprinodo n variegatus*	Teleostei	ENSCVAP000000 31154	ENSCVAT000000 25877	ENSCVAG000000 20701	Ensem bl	328
363	Cyprinodon_variegatus_ENSCVA G00000020750	ENSCVAG00000 020750	*Cyprinodo n variegatus*	Teleostei	ENSCVAP000000 17626	ENSCVAT000000 26346	ENSCVAG000000 20750	Ensem bl	288
364	Cyprinodon_variegatus_ENSCVA G00000021461	ENSCVAG00000 021461	*Cyprinodo n variegatus*	Teleostei	ENSCVAP000000 18041	ENSCVAT000000 26874	ENSCVAG000000 21461	Ensem bl	535
365	Cyprinodon_variegatus_ovch2	*ovch2*	*Cyprinodo n variegatus*	Teleostei	ENSCVAP000000 32779	ENSCVAT000000 33257	ENSCVAG000000 23100	Ensem bl	345
366	Cyprinodon_variegatus_si:dkey-32n7.7	si:dkey-32n7.7	*Cyprinodo n*	Teleostei	ENSCVAP000000 22686	ENSCVAT000000 08671	ENSCVAG000000 05545	Ensem bl	281

			variegatus						
367	Cyprinodon_variegatus_TMPRSS 13	*TMPRSS13*	*Cyprinodo n variegatus*	Teleostei	ENSCVAP000000 29583	ENSCVAT000000 22693	ENSCVAG000000 17450	Ensem bl	488
368	Cyprinodon_variegatus_tmprss2	*tmprss2*	*Cyprinodo n variegatus*	Teleostei	ENSCVAP000000 30356	ENSCVAT000000 24189	ENSCVAG000000 00492	Ensem bl	427
369	Cyprinodon_variegatus_tmprss3b	*tmprss3b*	*Cyprinodo n variegatus*	Teleostei	ENSCVAP000000 08466	ENSCVAT000000 01576	ENSCVAG000000 10342	Ensem bl	382
370	Cyprinodon_variegatus_tmprss5	*tmprss5*	*Cyprinodo n variegatus*	Teleostei	ENSCVAP000000 16913	ENSCVAT000000 25440	ENSCVAG000000 20202	Ensem bl	401
371	Cyprinodon_variegatus_zgc:12321 7	zgc:123217	*Cyprinodo n variegatus*	Teleostei	ENSCVAP000000 22624	ENSCVAT000000 08837	ENSCVAG000000 05424	Ensem bl	319
372	Cyprinodon_variegatus_zgc:92313	zgc:92313	*Cyprinodo n variegatus*	Teleostei	ENSCVAP000000 04329	ENSCVAT000000 08527	ENSCVAG000000 05616	Ensem bl	309
373	Danio_rerio_ENSDARG00000062 758	ENSDARG00000 062758	*Danio rerio*	Teleostei	ENSDARP000000 85529	ENSDART000000 91096	ENSDARG000000 62758	Ensem bl	296
374	Danio_rerio_ovch2	*ovch2*	*Danio rerio*	Teleostei	ENSDARP000001 14446	ENSDART000001 40780	ENSDARG000000 94793	Ensem bl	386
375	Danio_rerio_prss60.1	*prss60.1*	*Danio rerio*	Teleostei	ENSDARP000000 16272	ENSDART000000 04240	ENSDARG000000 70713	Ensem bl	387
376	Danio_rerio_prss60.2	*prss60.2*	*Danio rerio*	Teleostei	ENSDARP000000 19814	ENSDART000000 03716	ENSDARG000000 55644	Ensem bl	328
377	Danio_rerio_prss60.3	*prss60.3*	*Danio rerio*	Teleostei	ENSDARP000001 27971	ENSDART000001 54232	ENSDARG000000 70710	Ensem bl	330
378	Danio_rerio_si:ch73-182e20.3	si:ch73-182e20.3	*Danio rerio*	Teleostei	ENSDARP000000 95227	ENSDART000001 04453	ENSDARG000001 00953	Ensem bl	308
379	Danio_rerio_si:ch73-182e20.4	si:ch73-182e20.4	*Danio rerio*	Teleostei	ENSDARP000001 35587	ENSDART000001 71627	ENSDARG000001 02727	Ensem bl	275
380	Danio_rerio_si:dkey-16l2.17	si:dkey-16l2.17	*Danio rerio*	Teleostei	ENSDARP000000 35814	ENSDART000000 29262	ENSDARG000000 27196	Ensem bl	305
381	Danio_rerio_si:dkey-32n7.9	si:dkey-32n7.9	*Danio rerio*	Teleostei	ENSDARP000000 97350	ENSDART000001 06572	ENSDARG000000 71841	Ensem bl	282

382	Danio_rerio_si:dkeyp-93a5.2	si:dkeyp-93a5.2	*Danio rerio*	Teleostei	ENSDARP00000127837	ENSDART00000156328	ENSDARG00000052064	Ensembl	284
383	Danio_rerio_si:dkeyp-93a5.3	si:dkeyp-93a5.3	*Danio rerio*	Teleostei	ENSDARP00000128709	ENSDART00000155354	ENSDARG00000096975	Ensembl	306
384	Danio_rerio_tmprss12	*tmprss12*	*Danio rerio*	Teleostei	ENSDARP00000118661	ENSDART00000132635	ENSDARG00000093100	Ensembl	285
385	Danio_rerio_tmprss13a	*tmprss13a*	*Danio rerio*	Teleostei	ENSDARP00000133552	ENSDART00000164596	ENSDARG00000100969	Ensembl	506
386	Danio_rerio_tmprss13b	*tmprss13b*	*Danio rerio*	Teleostei	ENSDARP00000079274	ENSDART00000084839	ENSDARG00000077573	Ensembl	475
387	Danio_rerio_tmprss2	*tmprss2*	*Danio rerio*	Teleostei	ENSDARP00000134497	ENSDART00000172306	ENSDARG00000098686	Ensembl	487
388	Danio_rerio_tmprss3a	*tmprss3a*	*Danio rerio*	Teleostei	ENSDARP00000053217	ENSDART00000053218	ENSDARG00000053315	Ensembl	543
389	Danio_rerio_tmprss3b	*tmprss3b*	*Danio rerio*	Teleostei	ENSDARP00000121687	ENSDART00000132843	ENSDARG00000036545	Ensembl	326
390	Danio_rerio_tmprss5	*tmprss5*	*Danio rerio*	Teleostei	ENSDARP00000149114	ENSDART00000189840	ENSDARG00000087717	Ensembl	582
391	Danio_rerio_tmprss9	*tmprss9*	*Danio rerio*	Teleostei	ENSDARP00000122978	ENSDART00000145551	ENSDARG00000029841	Ensembl	800
392	Danio_rerio_zgc:100868	zgc:100868	*Danio rerio*	Teleostei	ENSDARP00000138127	ENSDART00000161644	ENSDARG00000004748	Ensembl	654
393	Danio_rerio_zgc:112038	zgc:112038	*Danio rerio*	Teleostei	ENSDARP00000067840	ENSDART00000067841	ENSDARG00000046140	Ensembl	311
394	Danio_rerio_zgc:123217	zgc:123217	*Danio rerio*	Teleostei	ENSDARP00000097428	ENSDART00000106650	ENSDARG00000059026	Ensembl	326
395	Danio_rerio_zgc:123295	zgc:123295	*Danio rerio*	Teleostei	ENSDARP00000073267	ENSDART00000078807	ENSDARG00000056324	Ensembl	310
396	Danio_rerio_zgc:153968	zgc:153968	*Danio rerio*	Teleostei	ENSDARP00000115045	ENSDART00000139213	ENSDARG00000061858	Ensembl	303
397	Danio_rerio_zgc:165423	zgc:165423	*Danio rerio*	Teleostei	ENSDARP00000069334	ENSDART00000074848	ENSDARG00000052905	Ensembl	538
398	Danio_rerio_zgc:92313	zgc:92313	*Danio rerio*	Teleostei	ENSDARP00000059297	ENSDART00000059298	ENSDARG00000040513	Ensembl	309
399	Fundulus_heteroclitus_ENSFHEG00000000549	ENSFHEG00000000549	*Fundulus heteroclitus*	Teleostei	ENSFHEP00000020311	ENSFHET00000035147	ENSFHEG00000000549	Ensembl	309

400	Fundulus_heteroclitus_ENSFHEG00000000649	ENSFHEG00000000649	*Fundulus heteroclitus*	Teleostei	ENSFHEP00000029386	ENSFHET00000019893	ENSFHEG00000000649	Ensembl	290
401	Fundulus_heteroclitus_ENSFHEG00000001809	ENSFHEG00000001809	*Fundulus heteroclitus*	Teleostei	ENSFHEP00000001048	ENSFHET00000014522	ENSFHEG00000001809	Ensembl	277
402	Fundulus_heteroclitus_ENSFHEG00000003317	ENSFHEG00000003317	*Fundulus heteroclitus*	Teleostei	ENSFHEP00000002310	ENSFHET00000012444	ENSFHEG00000003317	Ensembl	415
403	Fundulus_heteroclitus_ENSFHEG00000004211	ENSFHEG00000004211	*Fundulus heteroclitus*	Teleostei	ENSFHEP00000003350	ENSFHET00000010687	ENSFHEG00000004211	Ensembl	304
404	Fundulus_heteroclitus_ENSFHEG00000004230	ENSFHEG00000004230	*Fundulus heteroclitus*	Teleostei	ENSFHEP00000003310	ENSFHET00000010758	ENSFHEG00000004230	Ensembl	304
405	Fundulus_heteroclitus_ENSFHEG00000006276	ENSFHEG00000006276	*Fundulus heteroclitus*	Teleostei	ENSFHEP00000024600	ENSFHET00000007378	ENSFHEG00000006276	Ensembl	138
406	Fundulus_heteroclitus_ENSFHEG00000007952	ENSFHEG00000007952	*Fundulus heteroclitus*	Teleostei	ENSFHEP00000025612	ENSFHET00000004780	ENSFHEG00000007952	Ensembl	233
407	Fundulus_heteroclitus_ENSFHEG00000009586	ENSFHEG00000009586	*Fundulus heteroclitus*	Teleostei	ENSFHEP00000008394	ENSFHET00000002211	ENSFHEG00000009586	Ensembl	228
408	Fundulus_heteroclitus_ENSFHEG00000009592	ENSFHEG00000009592	*Fundulus heteroclitus*	Teleostei	ENSFHEP00000008263	ENSFHET00000002431	ENSFHEG00000009592	Ensembl	432
409	Fundulus_heteroclitus_ENSFHEG00000009828	ENSFHEG00000009828	*Fundulus heteroclitus*	Teleostei	ENSFHEP00000008531	ENSFHET00000001986	ENSFHEG00000009828	Ensembl	350
410	Fundulus_heteroclitus_ENSFHEG00000010792	ENSFHEG00000010792	*Fundulus heteroclitus*	Teleostei	ENSFHEP00000027352	ENSFHET00000000365	ENSFHEG00000010792	Ensembl	300
411	Fundulus_heteroclitus_ENSFHEG00000016495	ENSFHEG00000016495	*Fundulus heteroclitus*	Teleostei	ENSFHEP00000014898	ENSFHET00000032976	ENSFHEG00000016495	Ensembl	296
412	Fundulus_heteroclitus_ENSFHEG00000016743	ENSFHEG00000016743	*Fundulus heteroclitus*	Teleostei	ENSFHEP00000030717	ENSFHET00000022474	ENSFHEG00000016743	Ensembl	311

413	Fundulus_heteroclitus_ENSFHEG00000016844	ENSFHEG00000016844	*Fundulus heteroclitus*	Teleostei	ENSFHEP00000030816	ENSFHET00000022686	ENSFHEG00000016844	Ensembl	438
414	Fundulus_heteroclitus_ENSFHEG00000017052	ENSFHEG00000017052	*Fundulus heteroclitus*	Teleostei	ENSFHEP00000015201	ENSFHET00000023256	ENSFHEG00000017052	Ensembl	291
415	Fundulus_heteroclitus_ENSFHEG00000017075	ENSFHEG00000017075	*Fundulus heteroclitus*	Teleostei	ENSFHEP00000015454	ENSFHET00000033211	ENSFHEG00000017075	Ensembl	102
416	Fundulus_heteroclitus_ENSFHEG00000019174	ENSFHEG00000019174	*Fundulus heteroclitus*	Teleostei	ENSFHEP00000032637	ENSFHET00000026147	ENSFHEG00000019174	Ensembl	332
417	Fundulus_heteroclitus_ENSFHEG00000019237	ENSFHEG00000019237	*Fundulus heteroclitus*	Teleostei	ENSFHEP00000032641	ENSFHET00000026186	ENSFHEG00000019237	Ensembl	337
418	Fundulus_heteroclitus_ENSFHEG00000020566	ENSFHEG00000020566	*Fundulus heteroclitus*	Teleostei	ENSFHEP00000018658	ENSFHET00000027722	ENSFHEG00000020566	Ensembl	350
419	Fundulus_heteroclitus_ENSFHEG00000020607	ENSFHEG00000020607	*Fundulus heteroclitus*	Teleostei	ENSFHEP00000033444	ENSFHET00000027738	ENSFHEG00000020607	Ensembl	301
420	Fundulus_heteroclitus_ENSFHEG00000020776	ENSFHEG00000020776	*Fundulus heteroclitus*	Teleostei	ENSFHEP00000018725	ENSFHET00000027792	ENSFHEG00000020776	Ensembl	272
421	Fundulus_heteroclitus_ENSFHEG00000021537	ENSFHEG00000021537	*Fundulus heteroclitus*	Teleostei	ENSFHEP00000034032	ENSFHET00000034848	ENSFHEG00000021537	Ensembl	243
422	Fundulus_heteroclitus_ENSFHEG00000022217	ENSFHEG00000022217	*Fundulus heteroclitus*	Teleostei	ENSFHEP00000020283	ENSFHET00000035132	ENSFHEG00000022217	Ensembl	238
423	Fundulus_heteroclitus_ENSFHEG00000022862	ENSFHEG00000022862	*Fundulus heteroclitus*	Teleostei	ENSFHEP00000034740	ENSFHET00000030241	ENSFHEG00000022862	Ensembl	307
424	Fundulus_heteroclitus_ENSFHEG00000022914	ENSFHEG00000022914	*Fundulus heteroclitus*	Teleostei	ENSFHEP00000020950	ENSFHET00000030580	ENSFHEG00000022914	Ensembl	311
425	Fundulus_heteroclitus_ovch2	*ovch2*	*Fundulus heteroclitus*	Teleostei	ENSFHEP00000020020	ENSFHET00000035025	ENSFHEG00000021965	Ensembl	324

426	Fundulus_heteroclitus_tmprss13b	*tmprss13b*	*Fundulus heteroclitus*	Teleostei	ENSFHEP00000030574	ENSFHET00000032812	ENSFHEG00000015942	Ensembl	492
427	Fundulus_heteroclitus_tmprss2	*tmprss2*	*Fundulus heteroclitus*	Teleostei	ENSFHEP00000012323	ENSFHET00000019551	ENSFHEG00000013754	Ensembl	469
428	Fundulus_heteroclitus_TMPRSS5	*TMPRSS5*	*Fundulus heteroclitus*	Teleostei	ENSFHEP00000022776	ENSFHET00000012043	ENSFHEG00000003368	Ensembl	375
429	Fundulus_heteroclitus_tmprss9	*tmprss9*	*Fundulus heteroclitus*	Teleostei	ENSFHEP00000024612	ENSFHET00000007328	ENSFHEG00000006334	Ensembl	338
430	Fundulus_heteroclitus_zgc:123217	zgc:123217	*Fundulus heteroclitus*	Teleostei	ENSFHEP00000029358	ENSFHET00000019861	ENSFHEG00000014035	Ensembl	321
431	Fundulus_heteroclitus_zgc:92313	zgc:92313	*Fundulus heteroclitus*	Teleostei	ENSFHEP00000022523	ENSFHET00000012652	ENSFHEG00000002969	Ensembl	307
432	Gasterosteus_aculeatus_ENSGACG00000001457	ENSGACG00000001457	*Gasterosteus aculeatus*	Teleostei	ENSGACP00000001885	ENSGACT00000001888	ENSGACG00000001457	Ensembl	543
433	Gasterosteus_aculeatus_ENSGACG00000003060	ENSGACG00000003060	*Gasterosteus aculeatus*	Teleostei	ENSGACP00000004011	ENSGACT00000004025	ENSGACG00000003060	Ensembl	711
434	Gasterosteus_aculeatus_ENSGACG00000007675	ENSGACG00000007675	*Gasterosteus aculeatus*	Teleostei	ENSGACP00000010242	ENSGACT00000010264	ENSGACG00000007675	Ensembl	322
435	Gasterosteus_aculeatus_ENSGACG00000007789	ENSGACG00000007789	*Gasterosteus aculeatus*	Teleostei	ENSGACP00000010324	ENSGACT00000010346	ENSGACG00000007789	Ensembl	1804
436	Gasterosteus_aculeatus_ENSGACG00000008441	ENSGACG00000008441	*Gasterosteus aculeatus*	Teleostei	ENSGACP00000011155	ENSGACT00000011178	ENSGACG00000008441	Ensembl	349
437	Gasterosteus_aculeatus_ENSGACG00000014865	ENSGACG00000014865	*Gasterosteus aculeatus*	Teleostei	ENSGACP00000019625	ENSGACT00000019663	ENSGACG00000014865	Ensembl	353
438	Gasterosteus_aculeatus_ENSGACG00000014891	ENSGACG00000014891	*Gasterosteus aculeatus*	Teleostei	ENSGACP00000019656	ENSGACT00000019694	ENSGACG00000014891	Ensembl	576

439	Gasterosteus_aculeatus_ENSGACG00000018767	ENSGACG00000018767	*Gasterosteus aculeatus*	Teleostei	ENSGACP00000024811	ENSGACT00000024860	ENSGACG00000018767	Ensembl	360
440	Gasterosteus_aculeatus_ENSGACG00000023163	ENSGACG00000023163	*Gasterosteus aculeatus*	Teleostei	ENSGACP00000040838	ENSGACT00000074959	ENSGACG00000023163	Ensembl	320
441	Gasterosteus_aculeatus_ovch2	*ovch2*	*Gasterosteus aculeatus*	Teleostei	ENSGACP00000055240	ENSGACT00000039592	ENSGACG00000027088	Ensembl	301
442	Gasterosteus_aculeatus_TMPRSS13	*TMPRSS13*	*Gasterosteus aculeatus*	Teleostei	ENSGACP00000012355	ENSGACT00000012379	ENSGACG00000009369	Ensembl	493
443	Gasterosteus_aculeatus_tmprss2	*tmprss2*	*Gasterosteus aculeatus*	Teleostei	ENSGACP00000065410	ENSGACT00000040344	ENSGACG00000020187	Ensembl	522
444	Gasterosteus_aculeatus_tmprss5	*tmprss5*	*Gasterosteus aculeatus*	Teleostei	ENSGACP00000014203	ENSGACT00000014228	ENSGACG00000010730	Ensembl	460
445	Gasterosteus_aculeatus_tmprss9	*tmprss9*	*Gasterosteus aculeatus*	Teleostei	ENSGACP00000013734	ENSGACT00000013759	ENSGACG00000010394	Ensembl	522
446	Gasterosteus_aculeatus_zgc:92313	zgc:92313	*Gasterosteus aculeatus*	Teleostei	ENSGACP00000010377	ENSGACT00000010399	ENSGACG00000007820	Ensembl	302
447	Kryptolebias_marmoratus_ENSKMAG00000002096	ENSKMAG00000002096	*Kryptolebias marmoratus*	Teleostei	ENSKMAP00000003161	ENSKMAT00000003224	ENSKMAG00000002096	Ensembl	517
448	Kryptolebias_marmoratus_ENSKMAG00000002788	ENSKMAG00000002788	*Kryptolebias marmoratus*	Teleostei	ENSKMAP00000004098	ENSKMAT00000004176	ENSKMAG00000002788	Ensembl	484
449	Kryptolebias_marmoratus_ENSKMAG00000003675	ENSKMAG00000003675	*Kryptolebias marmoratus*	Teleostei	ENSKMAP00000005274	ENSKMAT00000005366	ENSKMAG00000003675	Ensembl	526
450	Kryptolebias_marmoratus_ENSKMAG00000006458	ENSKMAG00000006458	*Kryptolebias marmorat*	Teleostei	ENSKMAP00000008740	ENSKMAT00000008873	ENSKMAG00000006458	Ensembl	304

			us						
451	Kryptolebias_marmoratus_ENSKMAG00000008697	ENSKMAG00000008697	*Kryptolebias marmoratus*	Teleostei	ENSKMAP00000011566	ENSKMAT00000011744	ENSKMAG00000008697	Ensembl	286
452	Kryptolebias_marmoratus_ENSKMAG00000011424	ENSKMAG00000011424	*Kryptolebias marmoratus*	Teleostei	ENSKMAP00000015292	ENSKMAT00000015512	ENSKMAG00000011424	Ensembl	292
453	Kryptolebias_marmoratus_ENSKMAG00000019245	ENSKMAG00000019245	*Kryptolebias marmoratus*	Teleostei	ENSKMAP00000026154	ENSKMAT00000026484	ENSKMAG00000019245	Ensembl	307
454	Kryptolebias_marmoratus_ENSKMAG00000019431	ENSKMAG00000019431	*Kryptolebias marmoratus*	Teleostei	ENSKMAP00000026202	ENSKMAT00000026533	ENSKMAG00000019431	Ensembl	310
455	Kryptolebias_marmoratus_ENSKMAG00000021679	ENSKMAG00000021679	*Kryptolebias marmoratus*	Teleostei	ENSKMAP00000029255	ENSKMAT00000029620	ENSKMAG00000021679	Ensembl	319
456	Kryptolebias_marmoratus_ENSKMAG00000022153	ENSKMAG00000022153	*Kryptolebias marmoratus*	Teleostei	ENSKMAP00000029973	ENSKMAT00000030343	ENSKMAG00000022153	Ensembl	260
457	Kryptolebias_marmoratus_ovch2	*ovch2*	*Kryptolebias marmoratus*	Teleostei	ENSKMAP00000009235	ENSKMAT00000009370	ENSKMAG00000006909	Ensembl	493
458	Kryptolebias_marmoratus_tmprss13b	*tmprss13b*	*Kryptolebias marmoratus*	Teleostei	ENSKMAP00000011919	ENSKMAT00000012099	ENSKMAG00000008972	Ensembl	502
459	Kryptolebias_marmoratus_tmprss2	*tmprss2*	*Kryptolebias marmoratus*	Teleostei	ENSKMAP00000002775	ENSKMAT00000002829	ENSKMAG00000002110	Ensembl	271
460	Kryptolebias_marmoratus_TMPRSS3	*TMPRSS3*	*Kryptolebias marmorat*	Teleostei	ENSKMAP00000004033	ENSKMAT00000004111	ENSKMAG00000003086	Ensembl	543

			us						
461	Kryptolebias_marmoratus_tmprss5	*tmprss5*	*Kryptolebias marmoratus*	Teleostei	ENSKMAP00000008226	ENSKMAT00000008353	ENSKMAG00000006191	Ensembl	445
462	Kryptolebias_marmoratus_tmprss9	*tmprss9*	*Kryptolebias marmoratus*	Teleostei	ENSKMAP00000029969	ENSKMAT00000030339	ENSKMAG00000022152	Ensembl	472
463	Kryptolebias_marmoratus_zgc:92313	zgc:92313	*Kryptolebias marmoratus*	Teleostei	ENSKMAP00000021558	ENSKMAT00000021837	ENSKMAG00000016040	Ensembl	310
464	Larimichthys_crocea_ENSLCRG00005004641	ENSLCRG00005004641	*Larimichthys crocea*	Teleostei	ENSLCRP00005010079	ENSLCRT00005010367	ENSLCRG00005004641	Ensembl	321
465	Larimichthys_crocea_ENSLCRG00005004645	ENSLCRG00005004645	*Larimichthys crocea*	Teleostei	ENSLCRP00005010089	ENSLCRT00005010377	ENSLCRG00005004645	Ensembl	400
466	Larimichthys_crocea_ENSLCRG00005007037	ENSLCRG00005007037	*Larimichthys crocea*	Teleostei	ENSLCRP00005016960	ENSLCRT00005017320	ENSLCRG00005007037	Ensembl	356
467	Larimichthys_crocea_ENSLCRG00005008155	ENSLCRG00005008155	*Larimichthys crocea*	Teleostei	ENSLCRP00005019963	ENSLCRT00005020364	ENSLCRG00005008155	Ensembl	301
468	Larimichthys_crocea_ENSLCRG00005010748	ENSLCRG00005010748	*Larimichthys crocea*	Teleostei	ENSLCRP00005026943	ENSLCRT00005027424	ENSLCRG00005010748	Ensembl	338
469	Larimichthys_crocea_ENSLCRG00005021042	ENSLCRG00005021042	*Larimichthys crocea*	Teleostei	ENSLCRP00005056555	ENSLCRT00005057447	ENSLCRG00005021042	Ensembl	272
470	Larimichthys_crocea_ENSLCRG00005022362	ENSLCRG00005022362	*Larimichthys crocea*	Teleostei	ENSLCRP00005060473	ENSLCRT00005061446	ENSLCRG00005022362	Ensembl	416
471	Larimichthys_crocea_ENSLCRG00005023249	ENSLCRG00005023249	*Larimichthys crocea*	Teleostei	ENSLCRP00005063393	ENSLCRT00005064406	ENSLCRG00005023249	Ensembl	238
472	Larimichthys_crocea_ovch2	*ovch2*	*Larimichthys crocea*	Teleostei	ENSLCRP00005049578	ENSLCRT00005050389	ENSLCRG00005018710	Ensembl	396
473	Larimichthys_crocea_si:ch73-182e20.3	si:ch73-182e20.3	*Larimichthys crocea*	Teleostei	ENSLCRP00005015659	ENSLCRT00005016002	ENSLCRG00005006567	Ensembl	466
474	Larimichthys_crocea_tmprss13b	*tmprss13b*	*Larimichthys crocea*	Teleostei	ENSLCRP00005033037	ENSLCRT00005033681	ENSLCRG00005013021	Ensembl	517
475	Larimichthys_crocea_tmprss2	*tmprss2*	*Larimichthys crocea*	Teleostei	ENSLCRP00005015362	ENSLCRT00005015701	ENSLCRG00005006519	Ensembl	481

476	Larimichthys_crocea_TMPRSS3	*TMPRSS3*	*Larimichthys crocea*	Teleostei	ENSLCRP00005029422	ENSLCRT00005029967	ENSLCRG00005011667	Ensembl	382
477	Larimichthys_crocea_tmprss5	*tmprss5*	*Larimichthys crocea*	Teleostei	ENSLCRP00005016409	ENSLCRT00005016761	ENSLCRG00005006844	Ensembl	201
478	Larimichthys_crocea_tmprss9	*tmprss9*	*Larimichthys crocea*	Teleostei	ENSLCRP00005056563	ENSLCRT00005057455	ENSLCRG00005021045	Ensembl	342
479	Larimichthys_crocea_zgc:123217	zgc:123217	*Larimichthys crocea*	Teleostei	ENSLCRP00005015372	ENSLCRT00005015711	ENSLCRG00005006522	Ensembl	327
480	Larimichthys_crocea_zgc:92313	zgc:92313	*Larimichthys crocea*	Teleostei	ENSLCRP00005016087	ENSLCRT00005016435	ENSLCRG00005006740	Ensembl	369
481	Nothobranchius_furzeri_ENSNFUG00015006725	ENSNFUG00015006725	*Nothobranchius furzeri*	Teleostei	ENSNFUP00015013766	ENSNFUT00015014455	ENSNFUG00015006725	Ensembl	353
482	Nothobranchius_furzeri_ENSNFUG00015006749	ENSNFUG00015006749	*Nothobranchius furzeri*	Teleostei	ENSNFUP00015013808	ENSNFUT00015014499	ENSNFUG00015006749	Ensembl	408
483	Nothobranchius_furzeri_ENSNFUG00015006799	ENSNFUG00015006799	*Nothobranchius furzeri*	Teleostei	ENSNFUP00015013929	ENSNFUT00015014627	ENSNFUG00015006799	Ensembl	236
484	Nothobranchius_furzeri_ENSNFUG00015009143	ENSNFUG00015009143	*Nothobranchius furzeri*	Teleostei	ENSNFUP00015018987	ENSNFUT00015019876	ENSNFUG00015009143	Ensembl	423
485	Nothobranchius_furzeri_ENSNFUG00015010092	ENSNFUG00015010092	*Nothobranchius furzeri*	Teleostei	ENSNFUP00015020862	ENSNFUT00015021852	ENSNFUG00015010092	Ensembl	322
486	Nothobranchius_furzeri_ENSNFUG00015014297	ENSNFUG00015014297	*Nothobranchius furzeri*	Teleostei	ENSNFUP00015029412	ENSNFUT00015030727	ENSNFUG00015014297	Ensembl	493
487	Nothobranchius_furzeri_ENSNFUG00015017110	ENSNFUG00015017110	*Nothobranchius furzeri*	Teleostei	ENSNFUP00015035184	ENSNFUT00015036747	ENSNFUG00015017110	Ensembl	308
488	Nothobranchius_furzeri_ENSNFUG00015020048	ENSNFUG00015020048	*Nothobranchius furzeri*	Teleostei	ENSNFUP00015041822	ENSNFUT00015043660	ENSNFUG00015020048	Ensembl	548
489	Nothobranchius_furzeri_ENSNFUG00015021485	ENSNFUG00015021485	*Nothobranchius furzeri*	Teleostei	ENSNFUP00015045761	ENSNFUT00015047758	ENSNFUG00015021485	Ensembl	611
490	Nothobranchius_furzeri_ENSNFU	ENSNFUG00015	*Nothobranchius*	Teleostei	ENSNFUP0001504	ENSNFUT0001504	ENSNFUG000150	Ensem	702

	G00015021948	021948	*furzeri*		6283	8304	21948	bl	
491	Nothobranchius_furzeri_ENSNFUG00015024028	ENSNFUG00015024028	*Nothobranchius furzeri*	Teleostei	ENSNFUP00015051243	ENSNFUT00015053447	ENSNFUG00015024028	Ensembl	305
492	Nothobranchius_furzeri_ENSNFUG00015025445	ENSNFUG00015025445	*Nothobranchius furzeri*	Teleostei	ENSNFUP00015054884	ENSNFUT00015057197	ENSNFUG00015025445	Ensembl	284
493	Nothobranchius_furzeri_si:ch73-182e20.4	si:ch73-182e20.4	*Nothobranchius furzeri*	Teleostei	ENSNFUP00015022448	ENSNFUT00015023481	ENSNFUG00015010653	Ensembl	482
494	Nothobranchius_furzeri_tmprss3b	*tmprss3b*	*Nothobranchius furzeri*	Teleostei	ENSNFUP00015032929	ENSNFUT00015034420	ENSNFUG00015016043	Ensembl	366
495	Nothobranchius_furzeri_tmprss5	*tmprss5*	*Nothobranchius furzeri*	Teleostei	ENSNFUP00015003682	ENSNFUT00015003895	ENSNFUG00015001849	Ensembl	466
496	Nothobranchius_furzeri_tmprss9	*tmprss9*	*Nothobranchius furzeri*	Teleostei	ENSNFUP00015054883	ENSNFUT00015057196	ENSNFUG00015025444	Ensembl	525
497	Nothobranchius_furzeri_zgc:92313	zgc:92313	*Nothobranchius furzeri*	Teleostei	ENSNFUP00015023344	ENSNFUT00015024419	ENSNFUG00015011301	Ensembl	303
498	Oryzias_latipes_ENSORLG00000000238	ENSORLG00000000238	*Oryzias latipes*	Teleostei	ENSORLP00000000292	ENSORLT00000000292	ENSORLG00000000238	Ensembl	292
499	Oryzias_latipes_ENSORLG00000000242	ENSORLG00000000242	*Oryzias latipes*	Teleostei	ENSORLP00000029522	ENSORLT00000028346	ENSORLG00000000242	Ensembl	308
500	Oryzias_latipes_ENSORLG00000000252	ENSORLG00000000252	*Oryzias latipes*	Teleostei	ENSORLP00000042189	ENSORLT00000031445	ENSORLG00000000252	Ensembl	320
501	Oryzias_latipes_ENSORLG00000000256	ENSORLG00000000256	*Oryzias latipes*	Teleostei	ENSORLP00000034271	ENSORLT00000046271	ENSORLG00000000256	Ensembl	294
502	Oryzias_latipes_ENSORLG00000000282	ENSORLG00000000282	*Oryzias latipes*	Teleostei	ENSORLP00000000344	ENSORLT00000000344	ENSORLG00000000282	Ensembl	283
503	Oryzias_latipes_ENSORLG00000001175	ENSORLG00000001175	*Oryzias latipes*	Teleostei	ENSORLP00000001443	ENSORLT00000001444	ENSORLG00000001175	Ensembl	298
504	Oryzias_latipes_ENSORLG00000001206	ENSORLG00000001206	*Oryzias latipes*	Teleostei	ENSORLP00000039202	ENSORLT00000038850	ENSORLG00000001206	Ensembl	459
505	Oryzias_latipes_ENSORLG00000001209	ENSORLG00000001209	*Oryzias latipes*	Teleostei	ENSORLP00000001488	ENSORLT00000001489	ENSORLG00000001209	Ensembl	247

506	Oryzias_latipes_ENSORLG00000001213	ENSORLG00000001213	*Oryzias latipes*	Teleostei	ENSORLP00000001494	ENSORLT00000001495	ENSORLG00000001213	Ensembl	388
507	Oryzias_latipes_ENSORLG00000005158	ENSORLG00000005158	*Oryzias latipes*	Teleostei	ENSORLP00000006500	ENSORLT00000006501	ENSORLG00000005158	Ensembl	230
508	Oryzias_latipes_ENSORLG00000006898	ENSORLG00000006898	*Oryzias latipes*	Teleostei	ENSORLP00000008667	ENSORLT00000008668	ENSORLG00000006898	Ensembl	308
509	Oryzias_latipes_ENSORLG00000008474	ENSORLG00000008474	*Oryzias latipes*	Teleostei	ENSORLP00000010641	ENSORLT00000010642	ENSORLG00000008474	Ensembl	333
510	Oryzias_latipes_ENSORLG00000008481	ENSORLG00000008481	*Oryzias latipes*	Teleostei	ENSORLP00000010649	ENSORLT00000010650	ENSORLG00000008481	Ensembl	333
511	Oryzias_latipes_ENSORLG00000008718	ENSORLG00000008718	*Oryzias latipes*	Teleostei	ENSORLP00000010944	ENSORLT00000010945	ENSORLG00000008718	Ensembl	293
512	Oryzias_latipes_ENSORLG00000021926	ENSORLG00000021926	*Oryzias latipes*	Teleostei	ENSORLP00000029323	ENSORLT00000033534	ENSORLG00000021926	Ensembl	304
513	Oryzias_latipes_ENSORLG00000022022	ENSORLG00000022022	*Oryzias latipes*	Teleostei	ENSORLP00000032033	ENSORLT00000035854	ENSORLG00000022022	Ensembl	269
514	Oryzias_latipes_ENSORLG00000022076	ENSORLG00000022076	*Oryzias latipes*	Teleostei	ENSORLP00000033635	ENSORLT00000046708	ENSORLG00000022076	Ensembl	143
515	Oryzias_latipes_ENSORLG00000023312	ENSORLG00000023312	*Oryzias latipes*	Teleostei	ENSORLP00000028385	ENSORLT00000037097	ENSORLG00000023312	Ensembl	282
516	Oryzias_latipes_ENSORLG00000023553	ENSORLG00000023553	*Oryzias latipes*	Teleostei	ENSORLP00000029062	ENSORLT00000033328	ENSORLG00000023553	Ensembl	503
517	Oryzias_latipes_ENSORLG00000024648	ENSORLG00000024648	*Oryzias latipes*	Teleostei	ENSORLP00000040695	ENSORLT00000037002	ENSORLG00000024648	Ensembl	487
518	Oryzias_latipes_ENSORLG00000024823	ENSORLG00000024823	*Oryzias latipes*	Teleostei	ENSORLP00000028290	ENSORLT00000045443	ENSORLG00000024823	Ensembl	302
519	Oryzias_latipes_ENSORLG00000025103	ENSORLG00000025103	*Oryzias latipes*	Teleostei	ENSORLP00000040897	ENSORLT00000040429	ENSORLG00000025103	Ensembl	353
520	Oryzias_latipes_ENSORLG00000025385	ENSORLG00000025385	*Oryzias latipes*	Teleostei	ENSORLP00000041093	ENSORLT00000029727	ENSORLG00000025385	Ensembl	302
521	Oryzias_latipes_ENSORLG00000025659	ENSORLG00000025659	*Oryzias latipes*	Teleostei	ENSORLP00000045819	ENSORLT00000029619	ENSORLG00000025659	Ensembl	326
522	Oryzias_latipes_ENSORLG00000026241	ENSORLG00000026241	*Oryzias latipes*	Teleostei	ENSORLP00000040588	ENSORLT00000027341	ENSORLG00000026241	Ensembl	314
523	Oryzias_latipes_ENSORLG00000026595	ENSORLG00000026595	*Oryzias latipes*	Teleostei	ENSORLP00000037108	ENSORLT00000031012	ENSORLG00000026595	Ensembl	1113

524	Oryzias_latipes_ENSORLG00000028700	ENSORLG00000028700	*Oryzias latipes*	Teleostei	ENSORLP00000042837	ENSORLT00000028845	ENSORLG00000028700	Ensembl	456
525	Oryzias_latipes_ENSORLG00000029191	ENSORLG00000029191	*Oryzias latipes*	Teleostei	ENSORLP00000045238	ENSORLT00000032628	ENSORLG00000029191	Ensembl	368
526	Oryzias_latipes_ENSORLG00000030280	ENSORLG00000030280	*Oryzias latipes*	Teleostei	ENSORLP00000036651	ENSORLT00000035179	ENSORLG00000030280	Ensembl	293
527	Oryzias_latipes_ENSORLG00000030405	ENSORLG00000030405	*Oryzias latipes*	Teleostei	ENSORLP00000026393	ENSORLT00000037730	ENSORLG00000030405	Ensembl	469
528	Oryzias_latipes_si:dkey-32n7.7	si:dkey-32n7.7	*Oryzias latipes*	Teleostei	ENSORLP00000008225	ENSORLT00000008226	ENSORLG00000006549	Ensembl	299
529	Oryzias_latipes_tmprss13b	*tmprss13b*	*Oryzias latipes*	Teleostei	ENSORLP00000009045	ENSORLT00000009046	ENSORLG00000007210	Ensembl	453
530	Oryzias_latipes_TMPRSS3	*TMPRSS3*	*Oryzias latipes*	Teleostei	ENSORLP00000031768	ENSORLT00000030309	ENSORLG00000022129	Ensembl	464
531	Oryzias_latipes_tmprss5	*tmprss5*	*Oryzias latipes*	Teleostei	ENSORLP00000044976	ENSORLT00000033620	ENSORLG00000005115	Ensembl	382
532	Oryzias_latipes_zgc:123217	zgc:123217	*Oryzias latipes*	Teleostei	ENSORLP00000007980	ENSORLT00000007981	ENSORLG00000006351	Ensembl	326
533	Oryzias_latipes_zgc:92313	zgc:92313	*Oryzias latipes*	Teleostei	ENSORLP00000008266	ENSORLT00000008267	ENSORLG00000006587	Ensembl	308
534	Poecilia_formosa_ENSPFOG00000000163	ENSPFOG00000000163	*Poecilia formosa*	Teleostei	ENSPFOP00000000155	ENSPFOT00000000155	ENSPFOG00000000163	Ensembl	307
535	Poecilia_formosa_ENSPFOG00000000179	ENSPFOG00000000179	*Poecilia formosa*	Teleostei	ENSPFOP00000000177	ENSPFOT00000000178	ENSPFOG00000000179	Ensembl	285
536	Poecilia_formosa_ENSPFOG00000003011	*tmprss13b*	*Poecilia formosa*	Teleostei	ENSPFOP00000003034	ENSPFOT00000003038	ENSPFOG00000003011	Ensembl	491
537	Poecilia_formosa_ENSPFOG00000004258	zgc:123217	*Poecilia formosa*	Teleostei	ENSPFOP00000004156	ENSPFOT00000004164	ENSPFOG00000004258	Ensembl	316
538	Poecilia_formosa_ENSPFOG00000004506	si:dkey-32n7.7	*Poecilia formosa*	Teleostei	ENSPFOP00000025241	ENSPFOT00000027759	ENSPFOG00000004506	Ensembl	301
539	Poecilia_formosa_ENSPFOG00000004510	ENSPFOG00000004510	*Poecilia formosa*	Teleostei	ENSPFOP00000004366	ENSPFOT00000004374	ENSPFOG00000004510	Ensembl	249
540	Poecilia_formosa_ENSPFOG00000004738	si:ch73-182e20.3	*Poecilia formosa*	Teleostei	ENSPFOP00000004714	ENSPFOT00000004723	ENSPFOG00000004738	Ensembl	302
541	Poecilia_formosa_ENSPFOG00000004928	ENSPFOG00000004928	*Poecilia formosa*	Teleostei	ENSPFOP00000004885	ENSPFOT00000004894	ENSPFOG00000004928	Ensembl	487

542	Poecilia_formosa_ENSPFOG00000005402	*tmprss5*	*Poecilia formosa*	Teleostei	ENSPFOP00000005366	ENSPFOT00000005375	ENSPFOG00000005402	Ensembl	435
543	Poecilia_formosa_ENSPFOG00000005735	ENSPFOG00000005735	*Poecilia formosa*	Teleostei	ENSPFOP00000005722	ENSPFOT00000005731	ENSPFOG00000005735	Ensembl	354
544	Poecilia_formosa_ENSPFOG00000006625	ENSPFOG00000006625	*Poecilia formosa*	Teleostei	ENSPFOP00000021393	ENSPFOT00000029123	ENSPFOG00000006625	Ensembl	658
545	Poecilia_formosa_ENSPFOG00000008651	*OVCH2*	*Poecilia formosa*	Teleostei	ENSPFOP00000026590	ENSPFOT00000028046	ENSPFOG00000008651	Ensembl	541
546	Poecilia_formosa_ENSPFOG00000012424	*tmprss9*	*Poecilia formosa*	Teleostei	ENSPFOP00000012445	ENSPFOT00000012462	ENSPFOG00000012424	Ensembl	800
547	Poecilia_formosa_ENSPFOG00000017381	*TMPRSS3*	*Poecilia formosa*	Teleostei	ENSPFOP00000017467	ENSPFOT00000017489	ENSPFOG00000017381	Ensembl	497
548	Poecilia_formosa_ENSPFOG00000019614	ENSPFOG00000019614	*Poecilia formosa*	Teleostei	ENSPFOP00000019738	ENSPFOT00000019761	ENSPFOG00000019614	Ensembl	575
549	Poecilia_formosa_ENSPFOG00000021734	ENSPFOG00000021734	*Poecilia formosa*	Teleostei	ENSPFOP00000024051	ENSPFOT00000023933	ENSPFOG00000021734	Ensembl	446
550	Poecilia_formosa_ENSPFOG00000022442	ENSPFOG00000022442	*Poecilia formosa*	Teleostei	ENSPFOP00000030007	ENSPFOT00000029830	ENSPFOG00000022442	Ensembl	330
551	Poecilia_formosa_ENSPFOG00000022545	*tmprss2*	*Poecilia formosa*	Teleostei	ENSPFOP00000030800	ENSPFOT00000029004	ENSPFOG00000022545	Ensembl	390
552	Poecilia_formosa_ENSPFOG00000023441	ENSPFOG00000023441	*Poecilia formosa*	Teleostei	ENSPFOP00000027187	ENSPFOT00000022856	ENSPFOG00000023441	Ensembl	182
553	Poecilia_formosa_ENSPFOG00000023554	zgc:92313	*Poecilia formosa*	Teleostei	ENSPFOP00000024497	ENSPFOT00000025637	ENSPFOG00000023554	Ensembl	306
554	Gambusia_affinis_ENSPFOT00000003038_R0	*tmprss13b*	*Gambusia affinis*	Teleostei	ENSPFOT00000003038_R0	ENSPFOT00000003038_R0	ENSPFOT00000003038_R0	Ensembl	501
555	Gambusia_affinis_ENSPFOT00000004164_R0	zgc:123217	*Gambusia affinis*	Teleostei	ENSPFOT00000004164_R0	ENSPFOT00000004164_R0	ENSPFOT00000004164_R0	Ensembl	311
556	Gambusia_affinis_ENSPFOT00000004723_R3	si:ch73-182e20.3	*Gambusia affinis*	Teleostei	ENSPFOT00000004723_R3	ENSPFOT00000004723_R3	ENSPFOT00000004723_R3	Ensembl	356
557	Gambusia_affinis_ENSPFOT00000005375_R0	*tmprss5*	*Gambusia affinis*	Teleostei	ENSPFOT00000005375_R0	ENSPFOT00000005375_R0	ENSPFOT00000005375_R0	Ensembl	418
558	Gambusia_affinis_ENSPFOT00000005731_R0	ENSPFOG00000005735	*Gambusia affinis*	Teleostei	ENSPFOT00000005731_R0	ENSPFOT00000005731_R0	ENSPFOT00000005731_R0	Ensembl	392
559	Gambusia_affinis_ENSPFOT00000012462_R0	*tmprss9*	*Gambusia affinis*	Teleostei	ENSPFOT00000012462_R0	ENSPFOT00000012462_R0	ENSPFOT00000012462_R0	Ensembl	796

560	Gambusia_affinis_ENSPFOT00000017489_R0	*TMPRSS3*	*Gambusia affinis*	Teleostei	ENSPFOT00000017489_R0	ENSPFOT00000017489_R0	ENSPFOT00000017489_R0	Ensembl	490
561	Gambusia_affinis_ENSPFOT00000019761_R0	ENSPFOG00000019614	*Gambusia affinis*	Teleostei	ENSPFOT00000019761_R0	ENSPFOT00000019761_R0	ENSPFOT00000019761_R0	Ensembl	462
562	Gambusia_affinis_ENSPFOT00000019761_R1	ENSPFOG00000019614	*Gambusia affinis*	Teleostei	ENSPFOT00000019761_R1	ENSPFOT00000019761_R1	ENSPFOT00000019761_R1	Ensembl	570
563	Gambusia_affinis_ENSPFOT00000022856_R0	ENSPFOG00000023441	*Gambusia affinis*	Teleostei	ENSPFOT00000022856_R0	ENSPFOT00000022856_R0	ENSPFOT00000022856_R0	Ensembl	182
564	Gambusia_affinis_ENSPFOT00000022856_R1	ENSPFOG00000023441	*Gambusia affinis*	Teleostei	ENSPFOT00000022856_R1	ENSPFOT00000022856_R1	ENSPFOT00000022856_R1	Ensembl	173
565	Gambusia_affinis_ENSPFOT00000022856_R8	ENSPFOG00000023441	*Gambusia affinis*	Teleostei	ENSPFOT00000022856_R8	ENSPFOT00000022856_R8	ENSPFOT00000022856_R8	Ensembl	235
566	Gambusia_affinis_ENSPFOT00000023933_R0	ENSPFOG00000021734	*Gambusia affinis*	Teleostei	ENSPFOT00000023933_R0	ENSPFOT00000023933_R0	ENSPFOT00000023933_R0	Ensembl	452
567	Gambusia_affinis_ENSPFOT00000023933_R1	ENSPFOG00000021734	*Gambusia affinis*	Teleostei	ENSPFOT00000023933_R1	ENSPFOT00000023933_R1	ENSPFOT00000023933_R1	Ensembl	503
568	Gambusia_affinis_ENSPFOT00000025637_R0	zgc:92313	*Gambusia affinis*	Teleostei	ENSPFOT00000025637_R0	ENSPFOT00000025637_R0	ENSPFOT00000025637_R0	Ensembl	306
569	Gambusia_affinis_ENSPFOT00000027759_R2	si:dkey-32n7.7	*Gambusia affinis*	Teleostei	ENSPFOT00000027759_R2	ENSPFOT00000027759_R2	ENSPFOT00000027759_R2	Ensembl	290
570	Gambusia_affinis_ENSPFOT00000027759_R7	si:dkey-32n7.7	*Gambusia affinis*	Teleostei	ENSPFOT00000027759_R7	ENSPFOT00000027759_R7	ENSPFOT00000027759_R7	Ensembl	319
571	Gambusia_affinis_ENSPFOT00000028046_R0	*OVCH2*	*Gambusia affinis*	Teleostei	ENSPFOT00000028046_R0	ENSPFOT00000028046_R0	ENSPFOT00000028046_R0	Ensembl	521
572	Gambusia_affinis_ENSPFOT00000029004_R0	*tmprss2*	*Gambusia affinis*	Teleostei	ENSPFOT00000029004_R0	ENSPFOT00000029004_R0	ENSPFOT00000029004_R0	Ensembl	388
573	Gambusia_affinis_ENSPFOT00000029123_R0	ENSPFOG00000006625	*Gambusia affinis*	Teleostei	ENSPFOT00000029123_R0	ENSPFOT00000029123_R0	ENSPFOT00000029123_R0	Ensembl	640
574	Poecilia_reticulata_ENSPREG00000005250	ENSPREG00000005250	*Poecilia reticulata*	Teleostei	ENSPREP00000007638	ENSPRET00000007730	ENSPREG00000005250	Ensembl	304
575	Poecilia_reticulata_ENSPREG00000009935	ENSPREG00000009935	*Poecilia reticulata*	Teleostei	ENSPREP00000014698	ENSPRET00000014849	ENSPREG00000009935	Ensembl	334
576	Poecilia_reticulata_ENSPREG00000009952	ENSPREG00000009952	*Poecilia reticulata*	Teleostei	ENSPREP00000014731	ENSPRET00000014882	ENSPREG00000009952	Ensembl	265
577	Poecilia_reticulata_ENSPREG00000009980	ENSPREG00000009980	*Poecilia reticulata*	Teleostei	ENSPREP00000014980	ENSPRET00000015135	ENSPREG00000009980	Ensembl	323

578	Poecilia_reticulata_ENSPREG00000011091	ENSPREG00000011091	*Poecilia reticulata*	Teleostei	ENSPREP00000016393	ENSPRET00000016571	ENSPREG00000011091	Ensembl	493
579	Poecilia_reticulata_ENSPREG00000011114	ENSPREG00000011114	*Poecilia reticulata*	Teleostei	ENSPREP00000016447	ENSPRET00000016626	ENSPREG00000011114	Ensembl	425
580	Poecilia_reticulata_ENSPREG00000011255	ENSPREG00000011255	*Poecilia reticulata*	Teleostei	ENSPREP00000016636	ENSPRET00000016816	ENSPREG00000011255	Ensembl	306
581	Poecilia_reticulata_ENSPREG00000011502	ENSPREG00000011502	*Poecilia reticulata*	Teleostei	ENSPREP00000017107	ENSPRET00000017289	ENSPREG00000011502	Ensembl	320
582	Poecilia_reticulata_ENSPREG00000016734	ENSPREG00000016734	*Poecilia reticulata*	Teleostei	ENSPREP00000024777	ENSPRET00000025026	ENSPREG00000016734	Ensembl	170
583	Poecilia_reticulata_ENSPREG00000019676	ENSPREG00000019676	*Poecilia reticulata*	Teleostei	ENSPREP00000029077	ENSPRET00000029398	ENSPREG00000019676	Ensembl	288
584	Poecilia_reticulata_ENSPREG00000022495	ENSPREG00000022495	*Poecilia reticulata*	Teleostei	ENSPREP00000033252	ENSPRET00000033630	ENSPREG00000022495	Ensembl	327
585	Poecilia_reticulata_ENSPREG00000023170	ENSPREG00000023170	*Poecilia reticulata*	Teleostei	ENSPREP00000034204	ENSPRET00000034585	ENSPREG00000023170	Ensembl	246
586	Poecilia_reticulata_ENSPREG00000023176	ENSPREG00000023176	*Poecilia reticulata*	Teleostei	ENSPREP00000034227	ENSPRET00000034608	ENSPREG00000023176	Ensembl	302
587	Poecilia_reticulata_ovch2	*ovch2*	*Poecilia reticulata*	Teleostei	ENSPREP00000002597	ENSPRET00000002645	ENSPREG00000001879	Ensembl	340
588	Poecilia_reticulata_tmprss13b	*tmprss13b*	*Poecilia reticulata*	Teleostei	ENSPREP00000033538	ENSPRET00000033917	ENSPREG00000022725	Ensembl	497
589	Poecilia_reticulata_tmprss2	*tmprss2*	*Poecilia reticulata*	Teleostei	ENSPREP00000015448	ENSPRET00000015605	ENSPREG00000010432	Ensembl	422
590	Poecilia_reticulata_TMPRSS3	*TMPRSS3*	*Poecilia reticulata*	Teleostei	ENSPREP00000022908	ENSPRET00000023146	ENSPREG00000015460	Ensembl	469
591	Poecilia_reticulata_TMPRSS5	*TMPRSS5*	*Poecilia reticulata*	Teleostei	ENSPREP00000015772	ENSPRET00000015937	ENSPREG00000010638	Ensembl	460
592	Poecilia_reticulata_zgc:123217	zgc:123217	*Poecilia reticulata*	Teleostei	ENSPREP00000034179	ENSPRET00000034560	ENSPREG00000023154	Ensembl	314
593	Poecilia_reticulata_zgc:92313	zgc:92313	*Poecilia reticulata*	Teleostei	ENSPREP00000034240	ENSPRET00000034621	ENSPREG00000023183	Ensembl	306
594	Poeciliopsis_prolifica_LOC129352355	LOC129352355	*Poeciliopsis prolifica*	Teleostei	XP_054877284.1	XM_055021309.1	LOC129352355	NCBI	683
595	Poeciliopsis_prolifica_LOC129352365	LOC129352365	*Poeciliopsis prolifica*	Teleostei	XP_054877304.1	XM_055021329.1	LOC129352365	NCBI	370

596	Poeciliopsis_prolifica_LOC129356601	LOC129356601	*Poeciliopsis prolifica*	Teleostei	XP_054882150.1	XM_055026175.1	LOC129356601	NCBI	690
597	Poeciliopsis_prolifica_LOC129361696	LOC129361696	*Poeciliopsis prolifica*	Teleostei	XP_054888700.1	XM_055032725.1	LOC129361696	NCBI	572
598	Poeciliopsis_prolifica_LOC129364928	LOC129364928	*Poeciliopsis prolifica*	Teleostei	XP_054893854.1	XM_055037879.1	LOC129364928	NCBI	889
599	Poeciliopsis_prolifica_LOC129370822	LOC129370822	*Poeciliopsis prolifica*	Teleostei	XP_054903003.1	XM_055047028.1	LOC129370822	NCBI	481
600	Poeciliopsis_prolifica_LOC129372289	LOC129372289	*Poeciliopsis prolifica*	Teleostei	XP_054905442.1	XM_055049467.1	LOC129372289	NCBI	301
601	Poeciliopsis_prolifica_LOC129373695	LOC129373695	*Poeciliopsis prolifica*	Teleostei	XP_054907716.1	XM_055051741.1	LOC129373695	NCBI	600
602	Poeciliopsis_prolifica_LOC129373794	LOC129373794	*Poeciliopsis prolifica*	Teleostei	XP_054907855.1	XM_055051880.1	LOC129373794	NCBI	1166
603	Poeciliopsis_prolifica_LOC129374016	LOC129374016	*Poeciliopsis prolifica*	Teleostei	XP_054908173.1	XM_055052198.1	LOC129374016	NCBI	333
604	Poeciliopsis_prolifica_LOC129377661	LOC129377661	*Poeciliopsis prolifica*	Teleostei	XP_054913741.1	XM_055057766.1	LOC129377661	NCBI	298
605	Poeciliopsis_prolifica_tmprss13a	*tmprss13a*	*Poeciliopsis prolifica*	Teleostei	XP_054885073.1	XM_055029098.1	tmprss13a	NCBI	493
606	Poeciliopsis_prolifica_tmprss2	*tmprss2*	*Poeciliopsis prolifica*	Teleostei	XP_054902670.1	XM_055046695.1	tmprss2	NCBI	450
607	Poeciliopsis_prolifica_tmprss3a	*tmprss3a*	*Poeciliopsis prolifica*	Teleostei	XP_054878361.1	XM_055022386.1	tmprss3a	NCBI	538
608	Poeciliopsis_prolifica_tmprss5	*tmprss5*	*Poeciliopsis prolifica*	Teleostei	XP_054901390.1	XM_055045415.1	tmprss5	NCBI	460
609	Poeciliopsis_prolifica_tmprss9	*tmprss9*	*Poeciliopsis prolifica*	Teleostei	XP_054899983.1	XM_055044008.1	tmprss9	NCBI	785
610	Poeciliopsis_prolifica_zgc_123217	zgc_123217	*Poeciliopsis prolifica*	Teleostei	XP_054913816.1	XM_055057841.1	zgc:123217	NCBI	316
611	Poeciliopsis_prolifica_zgc_92313	zgc_92313	*Poeciliopsis prolifica*	Teleostei	XP_054913740.1	XM_055057765.1	zgc:92313	NCBI	305
612	Xiphophorus_hellerii_LOC116708002	LOC116708002	*Xiphophorus hellerii*	Teleostei	XP_032401641.1	XM_032545750.1	LOC116708002	NCBI	517
613	Xiphophorus_hellerii_LOC116717289	LOC116717289	*Xiphophorus hellerii*	Teleostei	XP_032414439.1	XM_032558548.1	LOC116717289	NCBI	481

614	Xiphophorus_hellerii_LOC116726694	LOC116726694	*Xiphophorus hellerii*	Teleostei	XP_032429416.1	XM_032573525.1	LOC116726694	NCBI	303
615	Xiphophorus_hellerii_LOC116726771	LOC116726771	*Xiphophorus hellerii*	Teleostei	XP_032429549.1	XM_032573658.1	LOC116726771	NCBI	302
616	Xiphophorus_hellerii_LOC116727134	LOC116727134	*Xiphophorus hellerii*	Teleostei	XP_032430218.1	XM_032574327.1	LOC116727134	NCBI	306
617	Xiphophorus_hellerii_LOC116727135	LOC116727135	*Xiphophorus hellerii*	Teleostei	XP_032430220.1	XM_032574329.1	LOC116727135	NCBI	301
618	Xiphophorus_hellerii_LOC116727408	LOC116727408	*Xiphophorus hellerii*	Teleostei	XP_032430713.1	XM_032574822.1	LOC116727408	NCBI	316
619	Xiphophorus_hellerii_LOC116732476	LOC116732476	*Xiphophorus hellerii*	Teleostei	XP_032438579.1	XM_032582688.1	LOC116732476	NCBI	343
620	Xiphophorus_hellerii_LOC116732477	LOC116732477	*Xiphophorus hellerii*	Teleostei	XP_032438580.1	XM_032582689.1	LOC116732477	NCBI	331
621	Xiphophorus_hellerii_LOC116733191	LOC116733191	*Xiphophorus hellerii*	Teleostei	XP_032439863.1	XM_032583972.1	LOC116733191	NCBI	344
622	Xiphophorus_hellerii_LOC116734956	LOC116734956	*Xiphophorus hellerii*	Teleostei	XP_032442392.1	XM_032586501.1	LOC116734956	NCBI	306
623	Xiphophorus_hellerii_LOC116734957	LOC116734957	*Xiphophorus hellerii*	Teleostei	XP_032442394.1	XM_032586503.1	LOC116734957	NCBI	305
624	Xiphophorus_hellerii_LOC116735111	LOC116735111	*Xiphophorus hellerii*	Teleostei	XP_032442630.1	XM_032586739.1	LOC116735111	NCBI	577
625	Xiphophorus_hellerii_LOC116735396	LOC116735396	*Xiphophorus hellerii*	Teleostei	XP_032443162.1	XM_032587271.1	LOC116735396	NCBI	670
626	Xiphophorus_hellerii_LOC116735750	LOC116735750	*Xiphophorus hellerii*	Teleostei	XP_032443712.1	XM_032587821.1	LOC116735750	NCBI	1176
627	Xiphophorus_hellerii_LOC116735768	LOC116735768	*Xiphophorus hellerii*	Teleostei	XP_032443739.1	XM_032587848.1	LOC116735768	NCBI	349
628	Xiphophorus_hellerii_tmprss13a	*tmprss13a*	*Xiphophorus hellerii*	Teleostei	XP_032401157.1	XM_032545266.1	tmprss13a	NCBI	491
629	Xiphophorus_hellerii_tmprss2	*tmprss2*	*Xiphophorus hellerii*	Teleostei	XP_032433042.1	XM_032577151.1	tmprss2	NCBI	419
630	Xiphophorus_hellerii_tmprss3a	*tmprss3a*	*Xiphophorus hellerii*	Teleostei	XP_032422976.1	XM_032567085.1	tmprss3a	NCBI	511
631	Xiphophorus_hellerii_tmprss9	*tmprss9*	*Xiphophorus hellerii*	Teleostei	XP_032428184.1	XM_032572293.1	tmprss9	NCBI	698

632	Xiphophorus_maculatus_ENSXMAG00000010605	ENSXMAG00000010605	*Xiphophorus maculatus*	Teleostei	ENSXMAP00000010629	ENSXMAT00000010642	ENSXMAG00000010605	Ensembl	380
633	Xiphophorus_maculatus_ENSXMAG00000012153	ENSXMAG00000012153	*Xiphophorus maculatus*	Teleostei	ENSXMAP00000012171	ENSXMAT00000012187	ENSXMAG00000012153	Ensembl	342
634	Xiphophorus_maculatus_ENSXMAG00000013783	ENSXMAG00000013783	*Xiphophorus maculatus*	Teleostei	ENSXMAP00000013814	ENSXMAT00000013831	ENSXMAG00000013783	Ensembl	303
635	Xiphophorus_maculatus_ENSXMAG00000018631	ENSXMAG00000018631	*Xiphophorus maculatus*	Teleostei	ENSXMAP00000023001	ENSXMAT00000034283	ENSXMAG00000018631	Ensembl	725
636	Xiphophorus_maculatus_ENSXMAG00000023686	ENSXMAG00000023686	*Xiphophorus maculatus*	Teleostei	ENSXMAP00000013658	ENSXMAT00000013674	ENSXMAG00000023686	Ensembl	634
637	Xiphophorus_maculatus_ENSXMAG00000024988	ENSXMAG00000024988	*Xiphophorus maculatus*	Teleostei	ENSXMAP00000038313	ENSXMAT00000042208	ENSXMAG00000024988	Ensembl	309
638	Xiphophorus_maculatus_ENSXMAG00000025882	ENSXMAG00000025882	*Xiphophorus maculatus*	Teleostei	ENSXMAP00000031565	ENSXMAT00000024400	ENSXMAG00000025882	Ensembl	333
639	Xiphophorus_maculatus_ENSXMAG00000027365	ENSXMAG00000027365	*Xiphophorus maculatus*	Teleostei	ENSXMAP00000037723	ENSXMAT00000035036	ENSXMAG00000027365	Ensembl	618
640	Xiphophorus_maculatus_ENSXMAG00000027927	ENSXMAG00000027927	*Xiphophorus maculatus*	Teleostei	ENSXMAP00000041402	ENSXMAT00000028912	ENSXMAG00000027927	Ensembl	361
641	Xiphophorus_maculatus_ENSXMAG00000029033	ENSXMAG00000029033	*Xiphophorus maculatus*	Teleostei	ENSXMAP00000036273	ENSXMAT00000021829	ENSXMAG00000029033	Ensembl	340
642	Xiphophorus_maculatus_OVCH2	*OVCH2*	*Xiphophorus maculatus*	Teleostei	ENSXMAP00000034946	ENSXMAT00000040750	ENSXMAG00000026877	Ensembl	499
643	Xiphophorus_maculatus_prss60.3	*prss60.3*	*Xiphophorus maculatus*	Teleostei	ENSXMAP00000024964	ENSXMAT00000031244	ENSXMAG00000023785	Ensembl	309
644	Xiphophorus_maculatus_si:dkey-32n7.7	si:dkey-32n7.7	*Xiphophorus maculatus*	Teleostei	ENSXMAP00000024581	ENSXMAT00000037422	ENSXMAG00000025167	Ensembl	310

645	Xiphophorus_maculatus_tmprss13b	*tmprss13b*	*Xiphophorus maculatus*	Teleostei	ENSXMAP00000034308	ENSXMAT00000027735	ENSXMAG00000004024	Ensembl	510
646	Xiphophorus_maculatus_TMPRSS3	*TMPRSS3*	*Xiphophorus maculatus*	Teleostei	ENSXMAP00000034842	ENSXMAT00000039200	ENSXMAG00000021103	Ensembl	375
647	Xiphophorus_maculatus_TMPRSS5	*TMPRSS5*	*Xiphophorus maculatus*	Teleostei	ENSXMAP00000009499	ENSXMAT00000009513	ENSXMAG00000009477	Ensembl	460
648	Xiphophorus_maculatus_zgc:153968	zgc:153968	*Xiphophorus maculatus*	Teleostei	ENSXMAP00000030735	ENSXMAT00000041977	ENSXMAG00000030046	Ensembl	305
649	Xiphophorus_maculatus_zgc:92313	zgc:92313	*Xiphophorus maculatus*	Teleostei	ENSXMAP00000039467	ENSXMAT00000033864	ENSXMAG00000026359	Ensembl	360

Note: Protein accession is the primary sequence identifier; nucleotide (CDS) accessions are provided where available.

Table S3A. Assembly integrity of the anchor-defined intervals used for gene presence/absence assessment.

Species	Target gene	Anchor 1 (scaffold:start–end)	Id%	Anchor 2 (scaffold:start–end)	Id%	Same scaffold?	Interval gaps (N)	Reliably assessable?
Branchiostoma floridae	*TMPRSS12*	NC_049996.1:10304068-10369937	63	NC_049997.1:1706809-1745280	47	No	N/A	No
Branchiostoma floridae	*ACR*	NC_049997.1:15090410-15252845	35	NC_049997.1:15328087-15336553	63	Yes	21555	Yes
Ciona intestinalis	*TMPRSS12*	9:6026221-6057973	53	9:4023217-4044671	52	Yes	34080	Yes
Ciona intestinalis	*ACR*	HT001267.1:1178-3193	42	2:7552573-7554608	61	No	N/A	No
Petromyzon marinus	*TMPRSS12*	GL477282:11654-105297	59	GL476358:329878-417404	61	No	N/A	No
Petromyzon marinus	*ACR*	GL478013:1684-51320	52	GL482214:800-8830	69	No	N/A	No
Callorhinchus milii	*TMPRSS12*	KI636116.1:411438-460724	83	KI635868.1:1190843-1233678	74	No	N/A	No
Callorhinchus milii	*ACR*	KI635862.1:10220214-10359173	45	AAVX02057816.1:177-1164	69	No	N/A	No

Scyliorhinus canicula	*TMPRSS12*	NC_052147.1:149773738-149977114	74	NC_052173.1:15258399-15478230	83	No	N/A	No
Scyliorhinus canicula	*ACR*	NC_052156.1:146877671-147764137	61	NC_052156.1:146384062-146393445	75	Yes	100	Yes
Polypterus senegalus	*TMPRSS12*	NC_053156.1:19987287-20116482	85	NC_053156.1:13330226-13610723	86	Yes	2500	Yes
Polypterus senegalus	*ACR*	NC_053161.1:102529457-103853089	63	NC_053161.1:103882027-103894086	70	Yes	0	Yes
Acipenser ruthenus	*TMPRSS12*	NC_081233.1:8956157-9003015	84	NC_081233.1:8885344-8928050	85	Yes	0	Yes
Acipenser ruthenus	*ACR*	NC_081202.1:14230459-14452873	63	NC_081202.1:14460676-14467590	71	Yes	0	Yes
Lepisosteus oculatus	*TMPRSS12*	LG4:16803164-16866623	83	LG4:16671215-16752667	87	Yes	2264	Yes
Lepisosteus oculatus	*ACR*	LG8:43249027-43713048	64	LG8:43723977-43730565	77	Yes	0	Yes
Oreochromis niloticus	*TMPRSS12*	LG20:20577359-20617244	80	LG20:20522506-20563795	82	Yes	0	Yes
Oreochromis niloticus	*ACR*	LG7:55608194-55756852	60	LG7:55597278-55599706	76	Yes	0	Yes
Danio rerio	*TMPRSS12*	23:27360106-27442393	79	23:27252305-27336327	81	Yes	0	Yes
Danio rerio	*ACR*	18:7663802-8029915	61	18:7639863-7648324	63	Yes	0	Yes
Oryzias latipes	*TMPRSS12*	7:11327645-11371742	80	7:11383565-11422104	80	Yes	0	Yes
Oryzias latipes	*ACR*	6:20291195-20485993	59	6:20283606-20285498	70	Yes	0	Yes
Sebastes schlegelii	*TMPRSS12*	CM025831.1:22037358-22082291	80	CM025831.1:22092701-22143209	81	Yes	0	Yes
Sebastes schlegelii	*ACR*	CM025829.1:21477506-21649779	60	CM025829.1:21466506-21470899	70	Yes	0	Yes
Takifugu rubripes	*TMPRSS12*	3:9338705-9362120	79	3:9369528-9399280	82	Yes	0	Yes
Takifugu rubripes	*ACR*	9:3029796-3109931	59	9:3023721-3025425	70	Yes	0	Yes
Kryptolebias marmoratus	*TMPRSS12*	LWHD01000145.1:737799-773363	79	LWHD01000145.1:678246-722862	82	Yes	1	Yes
Kryptolebias marmoratus	*ACR*	LWHD01000007.1:521736-654852	60	LWHD01000007.1:661721-663380	72	Yes	0	Yes

Nothobranchius furzeri	*TMPRSS12*	sgr15:19006675-19043112	79	sgr15:19054807-19095692	81	Yes	1	Yes
Nothobranchius furzeri	*ACR*	sgr13:27659820-27882547	58	sgr13:27647513-27649502	65	Yes	1	Yes
Cyprinodon variegatus	*TMPRSS12*	KL652547.1:1363946-1425920	79	KL653026.1:216227-269248	81	No	N/A	No
Cyprinodon variegatus	*ACR*	KL652744.1:322310-533479	60	KL652744.1:541328-542782	72	Yes	0	Yes
Fundulus heteroclitus	*TMPRSS12*	KN811376.1:1712662-1773920	80	KN811376.1:1792148-1846154	82	Yes	0	Yes
Fundulus heteroclitus	*ACR*	KN806074.1:14239-139424	58	KN806074.1:149694-153161	71	Yes	100	Yes
Poeciliopsis prolifica	*TMPRSS12*	NW_026562404.1:4298047-4343162	80	NW_026562404.1:4357016-4400063	81	Yes	0	Yes
Poeciliopsis prolifica	*ACR*	NW_026562376.1:11356473-11488860	59	NW_026562376.1:11347308-11349768	71	Yes	0	Yes
Gambusia affinis	*TMPRSS12*	LG01:19560039-19605185	80	LG01:19618837-19664476	81	Yes	0	Yes
Gambusia affinis	*ACR*	LG02:11991248-12122799	58	LG02:11981873-11984053	69	Yes	0	Yes
Poecilia reticulata	*TMPRSS12*	LG7:28160395-28206127	79	LG7:28092544-28146411	81	Yes	205	Yes
Poecilia reticulata	*ACR*	LG6:13565915-13705614	60	LG6:13556647-13558660	71	Yes	330	Yes
Xiphophorus maculatus	*TMPRSS12*	1:14302225-14350168	80	1:14363155-14419658	81	Yes	0	Yes
Xiphophorus maculatus	*ACR*	2:12527604-12675429	60	2:12517985-12520428	70	Yes	0	Yes
Latimeria chalumnae	*TMPRSS12*	JH126563.1:7296196-7378398	86	JH126563.1:5142374-5241275	87	Yes	185306	Yes
Latimeria chalumnae	*ACR*	JH128886.1:70872-203484	61	JH129115.1:94115-114480	74	No	N/A	No
Protopterus annectens	*TMPRSS12*	NC_056743.1:814090380-815568051	87	NC_056743.1:792362338-792454851	91	Yes	425837	Yes
Protopterus annectens	*ACR*	NC_056745.1:1224987320-1225253742	59	NC_056745.1:1258552192-1258778949	66	Yes	258199	Yes

Xenopus tropicalis	*TMPRSS12*	2:143298424-143408485	88	2:143075552-143146718	87	Yes	0	Yes
Xenopus tropicalis	*ACR*	3:89588856-89729257	65	3:89506445-89514700	71	Yes	0	Yes
Anolis carolinensis	*TMPRSS12*	2:91143152-91338481	88	2:90865054-90950977	92	Yes	756	Yes
Anolis carolinensis	*ACR*	GL343837.1:74615-207993	69	GL343765.1:58763-70015	87	No	N/A	No
Gallus gallus	*TMPRSS12*	34:319203-377518	93	34:240587-279759	93	Yes	0	Yes
Gallus gallus	*ACR*	1:571886-841084	70	1:524205-527753	75	Yes	0	Yes
Mus musculus	*TMPRSS12*	15:99936691-100113311	98	15:100834206-100938569	98	Yes	0	Yes
Mus musculus	*ACR*	15:89383835-89442526	95	15:89467562-89474582	80	Yes	0	Yes
Homo sapiens	*TMPRSS12*	12:50505141-50744839	100	12:51662818-51807429	99	Yes	0	Yes
Homo sapiens	*ACR*	22:50674415-50731312	99	22:50768776-50782294	100	Yes	0	Yes

Note: Anchor positions are the miniprot best-hit coordinates for each anchor gene (*dip2ba*/*scn8a* for *TMPRSS12*; *shank3*/*rabl2b* for *ACR*). Id% is the miniprot identity of the anchor alignment. Interval gaps are the number of N bases between the two anchor genes. N/A indicates that the two anchor genes fall on different scaffolds, so the interval cannot be defined and presence/absence within that species was assessed from whole-genome searches rather than from the interval alone. Species with larger interval gaps (e.g., *L. oculatus*, *N. furzeri*) were still scored as Lost because genome-wide tBLASTn recovered no *ACR*-coding hit, and all out-of-interval hits were verified by BLASTp best-hit as other S1 serine protease family members (Table S3D).

Table S3B. ***TMPRSS12*** **presence/absence validation by miniprot and tBLASTn genomic search with flanking-gene anchors (*dip2ba*–*scn8a*).**

**Species**	***dip2ba***			***scn8a***			***tmprss12***				**tblastn**			**Status**	**Final accession**
	**locus**	**CDS**	**Id%**	**locus**	**CDS**	**Id%**	**locus**	**CDS**	**in-int**	**Id%**	**hits**	**in-int**	**qcovs%**		
Branchiostoma floridae	NC_049996.1	39	63%	NC_049997.1	56	47%	*	*	—	*	52	—	81%	n/a	—
Ciona intestinalis	9	34	53%	9	34	52%	1	6	0	36%	22	0	68%	n/a	—
Petromyzon marinus	GL477282	37	59%	GL476358	24	61%	*	*	—	*	27	—	73%	n/a	—
Callorhinchu	KI636116.1	38	83%	KI635868.1	26	74	KI636116.1	4	—	45%	43	—	74%	Present	XP_04220009

s milii						%									1.1
Scyliorhinus canicula	NC_052147.1	37	74%	NC_052173.1	25	83%	NC_052161.1	8	—	35%	41	—	73%	Present	XP_038670763.1
Polypterus senegalus	NC_053156.1	37	85%	NC_053156.1	26	86%	NC_053157.1	4	0	47%	88	0	92%	Present †	XP_039620590.1
Acipenser ruthenus	NC_081233.1, NW_026708206.1	75	85%	NC_081233.1	25	85%	NC_081233.1, NC_081230.1	10	0	44%	132	0	93%	Present †	XP_033857193.3
Lepisosteus oculatus	LG4	38	83%	LG4	26	87%	*	*	0	*	129	6	93%	Present †	XP_015199639.1
Oreochromis niloticus	LG20	38	80%	LG5, LG20	53	82%	LG20	4	4	55%	162	4	90%	Present	XP_019204871.1
Danio rerio	6, ALT_CTG6_1_40…	113	79%	6, 23	51	83%	23	5	5	100%	160	6	100%	Present	XP_068073014.2
Oryzias latipes	7	38	80%	5, 7	52	81%	7	4	4	52%	153	4	91%	Present	XP_004070473.3
Sebastes schlegelii	CM025831.1	38	80%	CM025825.1, CM025831.1	53	81%	CM025844.1	7	0	36%	177	0	90%	Lost	—
Takifugu rubripes	3	37	79%	3	26	82%	7	6	0	38%	173	0	97%	Lost	—
Kryptolebias marmoratus	LWHD01000035.1, LWHD01000145.1	75	79%	LWHD01000035.1, LWHD01000145.1	53	82%	LWHD01000145.1	4	4	51%	111	4	90%	Present	XP_024865063.1
Nothobranchius furzeri	sgr09, sgr15	74	79%	sgr09, sgr15	53	82%	sgr15	4	4	55%	104	2	88%	Present	XP_015823280.3
Cyprinodon variegatus	KL652547.1	38	79%	KL653026.1	25	81%	KL653026.1	4	—	53%	159	—	90%	Present	XP_015246617.1
Fundulus heteroclitus	KN811376.1	38	80%	KN811496.1, KN811376.1	53	82%	KN811376.1	4	4	54%	163	3	85%	Present	XP_035992733.1
Poeciliopsis prolifica	NW_026562404.1	38	80%	NW_026562378.1, NW_026562404.1	52	82%	NW_026562404.1	4	4	49%	119	3	89%	Present	XP_054905442.1
Gambusia affinis	LG01	38	80%	LG20, LG01	52	82%	LG01	4	4	49%	112	4	93%	Present	XP_043983774.1
Poecilia	LG5, LG7	75	79%	LG5, LG7	52	82	LG7	4	4	50%	11	3	86%	Present	XP_00841178

reticulata						%					6				4.1
Xiphophorus maculatus	1	38	80%	20, 1	52	82%	1	4	4	50%	118	4	93%	Present	XP_005801464.3
Latimeria chalumnae	JH126563.1	38	86%	JH126563.1	26	87%	JH127265.1	4	0	40%	53	0	74%	Present †	XP_064408588.1
Protopterus annectens	NC_056728.1, NC_056743.1, NC_056731.1, NC_056735.1	98	87%	NC_056747.1, NC_056737.1, NC_056730.1, NC_056732.1, NC_056743.1	57	91%	NC_056729.1, NC_056745.1	4	0	34%	44	0	74%	Present †	XP_043945246.1 (a); XP_043926765.1 (b); XP_043923925.1 (c)
Xenopus tropicalis	2	38	88%	2	26	87%	9	4	0	36%	108	3	88%	Present †	XP_031751504.1
Anolis carolinensis	2	36	88%	2	26	92%	2	4	4	48%	67	18	79%	Present	XP_062827044.1
Gallus gallus	34	38	93%	34	26	93%	*	*	0	*	50	3	78%	Present †	XP_040549880.1
Mus musculus	15	38	98%	15	26	98%	15	4	4	66%	54	4	82%	Present	NP_898932.2
Homo sapiens	12	38	100%	12	26	99%	12	5	5	99%	56	5	100%	Present	NP_872365.2

Table S3C. *ACR* presence/absence validation by miniprot and tBLASTn genomic search with flanking-gene anchors (*shank3*–*rabl2b*).

**Species**	***shank3***			***rabl2b***			***acr***				**tblastn**			**Status**	**Final accession**
	**locus**	**CDS**	**Id%**	**locus**	**CDS**	**Id%**	**locus**	**CDS**	**in-int**	**Id%**	**hits**	**in-int**	**qcovs%**		
Branchiostoma floridae	NC_049997.1	19	35%	NC_049997.1	7	63%	NC_049979.1	2	0	35%	80	0	66%	n/a	—
Ciona intestinalis	HT001267.1	7	42%	2	6	61%	1	5	—	37%	32	—	62%	n/a	—
Petromyzon marinus	GL478013	5	52%	GL482214	8	69%	GL477748	4	—	43%	52	—	60%	n/a	—
Callorhinchu	KI635862.1	22	45	AAVX02057816.	3	69%	KI636116.1	7	—	34%	63	—	64%	Presen	XP_042191409.1

s milii			%	1									t	
Scyliorhinus canicula	NC_052156.1	24	61%	NC_052156.1	7	75%	NC_052164.1	7	0	42%	58	0	65%	Present†	XP_038668153.1
Polypterus senegalus	NC_053161.1	22	63%	NC_053161.1	7	70%	NW_024381105.1	4	0	39%	103	0	90%	Lost	—
Acipenser ruthenus	NC_081195.1, NC_081202.1	44	63%	NC_081202.1	8	71%	NC_081202.1	4	0	100%	176	0	99%	Present†	XP_033888599.1
Lepisosteus oculatus	LG8	22	64%	LG8	7	77%	JH591439.1	8	0	42%	139	0	89%	Lost	—
Oreochromis niloticus	LG7	23	60%	LG7	7	76%	*	*	0	*	211	0	83%	Lost	—
Danio rerio	18	22	61%	ALT_CTG18_1_3, ALT_CTG18_2_1…	21	63%	23, 22	8	0	38%	174	0	86%	Lost	—
Oryzias latipes	6	23	59%	6	8	70%	18	1	0	50%	168	0	88%	Lost	—
Sebastes schlegelii	CM025829.1	25	60%	CM025829.1	8	70%	CM025844.1	4	0	31%	185	0	89%	Lost	—
Takifugu rubripes	9	25	59%	9	8	70%	8	1	0	43%	179	0	93%	Lost	—
Kryptolebias marmoratus	LWHD01000007.1	25	60%	LWHD01000007.1	8	72%	LWHD01000500.1	5	0	33%	124	0	80%	Lost	—
Nothobranchius furzeri	sgr13	25	58%	sgr13	8	65%	sgr14	5	0	40%	116	0	92%	Lost	—
Cyprinodon variegatus	KL652744.1	23	60%	KL652744.1	7	72%	KL653196.1	2	0	33%	156	0	89%	Lost	—
Fundulus heteroclitus	KN806074.1	20	58%	KN806074.1	8	71%	KN811585.1	5	0	41%	154	0	89%	Lost	—
Poeciliopsis prolifica	NW_026562376.1	24	59%	NW_026562376.1	8	71%	*	*	0	*	131	0	94%	Lost	—
Gambusia affinis	LG02	23	58%	LG02	8	69%	LG14	1	0	52%	107	0	93%	Lost	—
Poecilia reticulata	LG6	24	60%	LG6	8	71%	LG18	1	0	53%	137	0	92%	Lost	—
Xiphophorus maculatus	2	25	60%	2	8	70%	*	*	0	*	119	0	92%	Lost	—

Latimeria chalumnae	JH128886.1	10	61%	JH129115.1	8	74%	JH128886.1	5	—	44%	57	—	62%	Present	XP_014352803.1
Protopterus annectens	NC_056751.1, NC_056745.1	11	67%	NC_056745.1, NC_056739.1, NC_056737.1, NC_056747.1	8	82%	NC_056748.1, NC_056741.1, NC_056728.1, NC_056726.1, NC_056744.1	14	0	33%	56	0	67%	Present†	XP_043929688.1 (a); XP_043929583.1 (b)
Xenopus tropicalis	3	23	65%	3	8	71%	1	5	0	42%	160	0	71%	Present†	XP_017946452.1
Anolis carolinensis	GL343837.1	10	69%	GL343765.1	6	87%	GL343765.1	6	—	43%	94	—	67%	Present	ENSACAP00000040759
Gallus gallus	1	22	70%	1	8	75%	1	14	0	42%	60	6	65%	Present†	XP_040515801.1
Mus musculus	15	22	95%	15	8	80%	15	5	5	70%	83	4	89%	Present	NP_038483.1
Homo sapiens	22	22	99%	22, 2	16	100%	22	5	5	100%	73	4	91%	Present	NP_001088.2

Note: Anchor-gene columns (*dip2ba*/*scn8a* in S3B; *shank3*/*rabl2b* in S3C) show locus (chromosome), miniprot CDS-block count, and Identity%. Target-gene columns (*tmprss12*/*acr*) show locus, CDS-block count, in-interval CDS (blocks within the anchor interval), and Identity%. tBLASTn columns show genome-wide hits, in-interval hits, and query coverage (qcovs%). * = no miniprot CDS hit (locus, CDS, and Id% columns); — = anchors on separate chromosomes, interval undefined (in-interval columns); 0 = interval defined but no hit within it (in-interval columns). Query rules: *TMPRSS12* — zebrafish (actinopterygians) or human (others); *ACR* —acipenseriform (actinopterygians) or human (others). Status: Present / Lost; n/a = outgroup (not scored); blank = unresolved. Presence was assigned using a tiered decision rule. A qualifying miniprot hit spanning at least three CDS blocks within the anchor-defined interval was scored as Present directly. When this criterion was not met, the miniprot best hit was distinguished from the final accepted candidate. Cases in which miniprot placed the target outside the anchor interval or on a different scaffold, or failed to recover a complete model while tBLASTn detected coding evidence within the interval, were scored as Present only when BLASTp best-hit and phylogenetic placement supported the candidate as the bona fide *tmprss12*/*acr* ortholog. Exception species are marked with a dagger (†).

Table S3D. BLASTp best-hit identification of out-of-interval miniprot hits for the *ACR* and *TMPRSS12* queries in gene-lost species.

**Target gene**	**Species**	**Query**	**Hit locus (scaffold:start–end)**	**Length (aa)**	**Best human BLASTp hit**	**Identity (%)**	**E-value**
ACR	*Polypterus senegalus*	human ACR	NW_024381105.1:8429–15817	256	*PRSS33*	46.3	2.5×10^{-78}
ACR	*Polypterus senegalus*	sturgeon ACR	NW_024381105.1:11808–15829	251	*PRSS8*	43.1	1.5×10^{-66}
ACR	*Lepisosteus oculatus*	human ACR	LG26:11224837–11333621	335	*TMPRSS13*	42.9	5.1×10^{-68}

ACR	*Lepisosteus oculatus*	sturgeon ACR	JH591439.1:149282–154800	247	*PRSS27*	47.5	2.7×10^{-74}
ACR	*Lepisosteus oculatus*	sturgeon ACR	JH591439.1:105302–154800	247	*PRSS27*	48.3	1.6×10^{-75}
ACR	*Oreochromis niloticus*	human ACR	LG17:23085459–23089068	221	*HPN*	40.2	2.5×10^{-52}
ACR	*Oreochromis niloticus*	sturgeon ACR	No hit	—	—	—	—
ACR	*Danio rerio*	human ACR	chr25:18744872–18745965	276	*TMPRSS15*	42.7	2.3×10^{-52}
ACR	*Danio rerio*	sturgeon ACR	chr23:27346204–27350851	252	*TMPRSS2*	42.2	4.6×10^{-56}
ACR	*Danio rerio*	sturgeon ACR	chr22:10761624–10768805	242	*TMPRSS9*	52.3	1.5×10^{-81}
ACR	*Oryzias latipes*	human ACR	chr8:2508159–2663392	337	*PRSS8*	43.8	3.7×10^{-68}
ACR	*Oryzias latipes*	human ACR	chr13:15147566–15193794	297	*HPN*	43.4	8.4×10^{-61}
ACR	*Oryzias latipes*	sturgeon ACR	chr18:779092–779263	58	*CFI*	62.3	1.4×10^{-18}
ACR	*Sebastes schlegelii*	human ACR	CM025834.1:20425016–20499982	335	*TMPRSS2*	51.7	1.1×10^{-78}
ACR	*Sebastes schlegelii*	sturgeon ACR	CM025844.1:22529578–22545971	211	*CFI*	50.0	3.0×10^{-44}
TMPRSS12	*Sebastes schlegelii*	zebrafish TMPRSS12	CM025844.1:22533659–22603051	250	*CFI*	44.7	3.0×10^{-56}
TMPRSS12	*Sebastes schlegelii*	human TMPRSS12	CM025847.1:549826–550797	240	*F7*	47.8	3.7×10^{-75}
ACR	*Takifugu rubripes*	human ACR	chr1:873180–921049	381	*CELA2A*	39.8	1.1×10^{-57}
ACR	*Takifugu rubripes*	human ACR	chr11:8489984–8586531	351	*TMPRSS13*	40.8	8.5×10^{-67}
ACR	*Takifugu rubripes*	sturgeon ACR	chr8:19467626–19467836	71	*CFI*	63.2	5.1×10^{-20}
TMPRSS12	*Takifugu rubripes*	zebrafish TMPRSS12	chr7:9220665–9224996	292	*HPN*	56.3	1.1×10^{-112}

TMPRSS12	*Takifugu rubripes*	human TMPRSS12	chr20:9618498–9621614	244	*TMPRSS9*	51.2	3.1×10^{-82}
ACR	*Kryptolebias marmoratus*	human ACR	LWHD01000343.1:23939–70509	338	*TMPRSS2*	42.0	1.8×10^{-55}
ACR	*Kryptolebias marmoratus*	sturgeon ACR	LWHD01000500.1:12721–14657	225	*CFI*	42.2	5.2×10^{-54}
ACR	*Nothobranchius furzeri*	human ACR	sgr01:96848010–96853062	314	*HPN*	41.0	1.4×10^{-52}
ACR	*Nothobranchius furzeri*	sturgeon ACR	sgr14:10470895–10485952	235	*TMPRSS3*	56.8	8.4×10^{-86}
ACR	*Cyprinodon variegatus*	human ACR	KL653507.1:60538–159405	328	*TMPRSS2*	41.8	3.2×10^{-54}
ACR	*Cyprinodon variegatus*	sturgeon ACR	KL653196.1:341406–345864	147	*CFI*	45.2	5.4×10^{-34}
ACR	*Fundulus heteroclitus*	human ACR	KN811472.1:324296–413464	315	*HPN*	42.6	1.6×10^{-55}
ACR	*Fundulus heteroclitus*	sturgeon ACR	KN811585.1:211397–214580	236	*TMPRSS5*	57.9	1.5×10^{-98}
ACR	*Poeciliopsis prolifica*	human ACR	NW_026562555.1:168064–187211	317	*TMPRSS3*	53.2	8.3×10^{-86}
ACR	*Poeciliopsis prolifica*	sturgeon ACR	No hit	—	—	—	—
ACR	*Gambusia affinis*	human ACR	LG07:438794–512510	329	*TMPRSS3*	57.6	9.8×10^{-94}
ACR	*Gambusia affinis*	sturgeon ACR	LG14:24437873–24438044	58	*CFI*	67.9	3.4×10^{-20}
ACR	*Poecilia reticulata*	human ACR	LG14:19046658–19115982	295	*TMPRSS2*	48.5	9.4×10^{-76}
ACR	*Poecilia reticulata*	sturgeon ACR	LG18:21720413–21720584	58	*CFI*	64.2	6.5×10^{-19}
ACR	*Xiphophorus maculatus*	human ACR	chr7:513566–610062	333	*TMPRSS3*	56.0	2.5×10^{-92}
ACR	*Xiphophorus maculatus*	sturgeon ACR	No hit	—	—	—	—

Note: Out-of-interval hits are miniprot alignments for the target-gene queries (*ACR*: human *ACR* and sturgeon *Acipenser ruthenus ACR*; *TMPRSS12*: zebrafish and human *TMPRSS12*) that fall outside the respective anchor interval (*shank3–rabl2b* for *ACR*; *dip2ba–scn8a* for *TMPRSS12*). Each hit was translated and searched against the human proteome (BLASTp, E-value 1e-5); the best human match is shown. Table S3D contains 38 search records, comprising 35 out-of-interval miniprot hits and three no-hit records. All

35 recovered hits were assigned to other S1 serine protease family members; none was identified as *ACR* or *TMPRSS12*. tBLASTn (E-value 1e-5) additionally recovered fragmentary hits per species outside the interval (78–211 for *ACR*; 88–177 for *TMPRSS12*), all representing cross-matches of the conserved S1 catalytic domain without spliced gene structure. Both search engines returned zero hits inside the anchor interval. Three species (*Oreochromis niloticus*, *Poeciliopsis prolifica*, *Xiphophorus maculatus*) had no miniprot hit for the sturgeon *ACR* query; four species (*Gambusia affinis*, *Oryzias latipes*, *Poecilia reticulata*, *Takifugu rubripes*) had only a 57–71 aa fragment covering the conserved GDSGGP substrate pocket.

Table S4. Trait matrix for ancestral state reconstruction.

Species	Clade	Subclade	*ACR*	*TMPRSS12*	Acrosome	Micropyle	Fertilization	Key Reference(s)
Branchiostoma floridae	Outgroup	Cephalochordata	N/A	N/A	Present	N/A	External	[2]
Ciona intestinalis	Outgroup	Urochordata	N/A	N/A	Present	N/A	External	[3]
Petromyzon marinus	Outgroup	Cyclostomata	N/A	N/A	Present	N/A	External	[4]
Callorhinchus milii	Chondrichthyes	Holocephali	Present	Present	Present	Absent	Internal	[5,6]
Scyliorhinus canicula	Chondrichthyes	Elasmobranchii	Present	Present	Present	Absent	Internal	[7–9]
Polypterus senegalus	Actinopterygii	Polypteriformes	Lost	Present	Present	Single	External	[10,11,12]
Acipenser ruthenus	Actinopterygii	Chondrostei	Present	Present	Present	Multiple	External	[13,14]
Lepisosteus oculatus	Actinopterygii	Holostei	Lost	Present	Absent	Single	External	[15,16]
Oreochromis niloticus	Actinopterygii	Teleostei	Lost	Present	Absent	Single	External	[17,18]
Danio rerio	Actinopterygii	Teleostei	Lost	Present	Absent	Single	External	[19,20]
Oryzias latipes	Actinopterygii	Teleostei	Lost	Present	Absent	Single	External	[21,22]
Sebastes schlegelii	Actinopterygii	Teleostei	Lost	Lost	Absent	Single	Internal	[23,24]
Takifugu rubripes	Actinopterygii	Teleostei	Lost	Lost	Absent	Single	External	[25,26]
Kryptolebias marmoratus	Actinopterygii	Teleostei (Cyprinodontiformes)	Lost	Present	Absent	Single	Selfing	[27,28]
Nothobranchius	Actinopterygii	Teleostei	Lost	Present	Absent	Single	External	[11,29]

furzeri		(Cyprinodontiformes)						
Cyprinodon variegatus	Actinopterygii	Teleostei (Cyprinodontiformes)	Lost	Present	Absent	Single	External	[30,31]
Fundulus heteroclitus	Actinopterygii	Teleostei (Cyprinodontiformes)	Lost	Present	Absent	Single	External	[32,33]
Poeciliopsis prolifica	Actinopterygii	Teleostei (Poeciliidae)	Lost	Present*	Absent	Absent	Internal	[34]
Gambusia affinis	Actinopterygii	Teleostei (Poeciliidae)	Lost	Present*	Absent	Absent	Internal	[34,35]
Poecilia reticulata	Actinopterygii	Teleostei (Poeciliidae)	Lost	Present*	Absent	Absent	Internal	[34,36]
Xiphophorus maculatus	Actinopterygii	Teleostei (Poeciliidae)	Lost	Present*	Absent	Absent	Internal	[34]
Protopterus annectens	Sarcopterygii	Dipnoi	Present	Present	Present	Absent*	External	[37]
Latimeria chalumnae	Sarcopterygii	Coelacanthiformes	Present	Present	Present	Absent*	Internal	[38]
Xenopus tropicalis	Tetrapoda	Amphibia	Present	Present	Present	Absent*	External	[39]
Anolis carolinensis	Tetrapoda	Reptilia	Present	Present	Present	Absent*	Internal	[40]
Gallus gallus	Tetrapoda	Aves	Present	Present	Present	Absent*	Internal	[41]
Mus musculus	Tetrapoda	Mammalia	Present	Present	Present	Absent*	Internal	[42]
Homo sapiens	Tetrapoda	Mammalia	Present	Present	Present	Absent*	Internal	[43]

Note: N/A: not applicable (outgroup), excluded from reconstruction. Coding: *ACR* (0 = present, 1 = lost); Acrosome (0 = present, 1 = absent); Micropyle (0 = absent, 1 = single, 2 = multiple); Fertilization (0 = external, 1 = internal, 2 = selfing; for *K. marmoratus*, "selfing" denotes self-fertilization within the gonadal lumen, a form of internal fertilization, with occasional male-mediated outcrossing through external fertilization). Present*: *TMPRSS12* present but under relaxed selection in Poeciliidae. Absent*: chondrichthyans, sarcopterygians, and tetrapods, which lack the actinopterygian-type chorion, were scored as micropyle-absent (0), and this state was included in the ancestral-state reconstruction. By contrast, N/A (not applicable) applies only to the three outgroup taxa, which were excluded from the reconstruction and therefore not coded for this trait. For selected species, trait states were informed by ultrastructural data from closely related taxa and are designated as indirect evidence: *Lepisosteus oculatus* from *L. osseus* [16]; *Nothobranchius furzeri* from *N. guentheri* and *N. patrizii* [29]; the four poeciliid species (*Poeciliopsis prolifica*, *Gambusia affinis*, *Poecilia reticulata*, and *Xiphophorus maculatus*) from *Heterandria formosa* [34]; *Protopterus annectens* from *Neoceratodus forsteri* [37]; and *Xenopus tropicalis* from *X. laevis* [39].

Table S5. Model selection and leave-one-out sensitivity analysis for ancestral state reconstruction.

Table S5A. Model selection

Trait	States	Model	lnL	K	AIC	ΔAIC	Starting points (hits)	Transition rates
ACR	2	ER	−7.416	1	16.832	0	1 (1)	0.000463
ACR	2	SYM	−7.416	1	16.832	0	1 (1)	0.000463
ACR	2	ARD	−7.274	2	18.548	1.715	51 (39)	0.000597; 0.000131
Acrosome	2	ER	−5.936	1	13.872	0	1 (1)	0.000254
Acrosome	2	SYM	−5.936	1	13.872	0	1 (1)	0.000254
Acrosome	2	ARD	−5.686	2	15.372	1.501	51 (35)	0.00031; 0
Micropyle	3	ER	−14.508	1	31.017	0	1 (1)	0.00034
Micropyle	3	SYM	−13.457	3	32.915	1.898	61 (45)	0; 0.000488; 0.000491
Micropyle	3	ARD	−12.948	6	37.896	6.879	201 (60)	0.007171; 0.000663; 0; 0.00076; 0; 0.00378
Fertilization	3	ER	−21.569	1	45.139	1.475	1 (1)	0.000927
Fertilization	3	SYM	−18.832	3	43.664	0	61 (33)	0.000377; 0.002228; 0
Fertilization	3	ARD	−16.900	6	45.800	2.137	201 (68)	0.161994; 0; 0.014799; 0; 0; 0.022869

Table S5B. Model sensitivity: ancestral-state probabilities under ER, ARD, and SYM.

Trait	Node	ER (state, p)	ARD (state, p)	SYM (state, p)
ACR	*Gnathostomata*	present (0.988)	present (1.000)	present (0.988)

ACR	*Osteichthyes*	present (0.986)	present (1.000)	present (0.986)
ACR	*Actinopterygii*	present (0.633)	present (0.865)	present (0.633)
ACR	*Neopterygii*	lost (0.804)	lost (0.638)	lost (0.804)
ACR	*Teleostei*	lost (0.979)	lost (0.921)	lost (0.979)
ACR	*Cyprinodontiformes*	lost (1.000)	lost (1.000)	lost (1.000)
Acrosome	*Gnathostomata*	present (1.000)	present (1.000)	present (1.000)
Acrosome	*Osteichthyes*	present (1.000)	present (1.000)	present (1.000)
Acrosome	*Actinopterygii*	present (0.993)	present (1.000)	present (0.993)
Acrosome	*Neopterygii*	absent (0.796)	absent (0.745)	absent (0.796)
Acrosome	*Teleostei*	absent (0.987)	absent (0.942)	absent (0.987)
Acrosome	*Cyprinodontiformes*	absent (1.000)	absent (1.000)	absent (1.000)
Micropyle	*Gnathostomata*	absent (0.985)	multiple (0.875)	absent (0.978)
Micropyle	*Osteichthyes*	absent (0.982)	multiple (0.854)	absent (0.973)
Micropyle	*Actinopterygii*	single (0.709)	single (0.657)	single (0.760)
Micropyle	*Neopterygii*	single (0.954)	single (0.862)	single (0.990)
Micropyle	*Teleostei*	single (0.996)	single (0.952)	single (0.998)
Micropyle	*Cyprinodontiformes*	single (1.000)	single (0.999)	single (1.000)
Fertilization	*Gnathostomata*	internal (0.535)	external or selfing (0.526)	internal (0.574)
Fertilization	*Osteichthyes*	external (0.511)	external (0.939)	internal (0.539)
Fertilization	*Actinopterygii*	external (0.764)	external (0.940)	external (0.585)
Fertilization	*Neopterygii*	external (0.945)	external (0.939)	external (0.765)
Fertilization	*Teleostei*	external (0.982)	external (0.941)	external (0.882)
Fertilization	*Cyprinodontiformes*	external (0.999)	external (0.903)	external (0.999)

Table S5C. Leave-one-out node sensitivity

**Trait**	**Node**	**ML state**	***p* (full)**	**Mean ± SD**	**Range**
ACR	Gnathostomata	present	0.988	0.985 ± 0.010	[0.95, 1.00]
ACR	Osteichthyes	present	0.986	0.983 ± 0.010	[0.95, 1.00]
ACR	Actinopterygii	present	0.633	0.639 ± 0.115	[0.20, 0.93]
ACR	Neopterygii	lost	0.804	0.784 ± 0.097	[0.38, 0.98]
ACR	Teleostei	lost	0.979	0.971 ± 0.033	[0.82, 1.00]
ACR	Cyprinodontiformes	lost	1.000	1.000 ± 0.000	[1.00, 1.00]
Acrosome	Gnathostomata	present	1.000	1.000 ± 0.000	[1.00, 1.00]
Acrosome	Osteichthyes	present	1.000	1.000 ± 0.000	[1.00, 1.00]
Acrosome	Actinopterygii	present	0.993	0.989 ± 0.014	[0.93, 1.00]
Acrosome	Neopterygii	absent	0.796	0.766 ± 0.117	[0.24, 0.91]
Acrosome	Teleostei	absent	0.987	0.978 ± 0.037	[0.80, 1.00]
Acrosome	Cyprinodontiformes	absent	1.000	1.000 ± 0.000	[1.00, 1.00]
Micropyle	Gnathostomata	absent	0.985	0.980 ± 0.015	[0.93, 0.99]
Micropyle	Osteichthyes	absent	0.982	0.977 ± 0.015	[0.93, 0.99]
Micropyle	Actinopterygii	single	0.709	0.676 ± 0.100	[0.26, 0.81]
Micropyle	Neopterygii	single	0.954	0.936 ± 0.057	[0.68, 0.98]
Micropyle	Teleostei	single	0.996	0.992 ± 0.012	[0.94, 1.00]
Micropyle	Cyprinodontiformes	single	1.000	1.000 ± 0.000	[1.00, 1.00]

Trait	Node	ML state	*p* (full)	Mean ± SD	Range
Fertilization	Gnathostomata	internal	0.574	0.571 ± 0.047	[0.47, 0.66]
Fertilization	Osteichthyes	internal	0.539	0.539 ± 0.050	[0.43, 0.62]
Fertilization	Actinopterygii	external	0.585	0.576 ± 0.047	[0.50, 0.67]
Fertilization	Neopterygii	external	0.765	0.747 ± 0.049	[0.65, 0.88]
Fertilization	Teleostei	external	0.882	0.864 ± 0.039	[0.74, 0.96]
Fertilization	Cyprinodontiformes	external	0.999	0.999 ± 0.001	[0.99, 1.00]

Note: Model comparison was based on AIC. ER = equal-rates model; SYM = symmetrical model; ARD = all-rates-different model. For binary traits (*ACR*, Acrosome), SYM is mathematically equivalent to ER and returns an identical likelihood. lnL = log-likelihood; K = number of estimated transition-rate parameters (ER: 1; SYM: k(k−1)/2; ARD: k(k−1)); ΔAIC = AIC − AIC(best model). Table S5B reports the maximum-likelihood ancestral state and its probability at six monitored nodes under each model; for the binary traits, the maximum-likelihood state at each monitored node was identical across ER, SYM, and ARD; for the three-state traits, most nodes were consistent across models, with minor differences at a few deep nodes. Fertilization was coded as three analytical states (external, internal, and selfing). The selfing state, recorded only for *Kryptolebias marmoratus*, represents self-fertilization within the gonadal lumen, a specialized form of internal fertilization, and was coded separately to capture this distinctive reproductive condition. Occasional male-mediated outcrossing occurs through external fertilization. In Table S5B, "external or selfing" denotes comparable probabilities of the external and selfing states under the ARD model, reflecting uncertainty between these two states rather than a separate character state. For each trait, leave-one-out statistics were computed by treating each species' character state as unknown (NA) in turn while retaining the complete 25-species phylogeny (25 replicates). p (full) = maximum-likelihood state probability from the complete dataset. Mean ± SD = average and standard deviation of the focal state probability across leave-one-out replicates. Range = [minimum, maximum] probability across all replicates. Nodes with SD < 0.02 are highly robust, SD 0.02–0.05 robust, and SD > 0.05 sensitive to taxonomic sampling and interpreted qualitatively. AIC and ΔAIC were calculated from the unrounded log-likelihood values; values shown in the table are rounded to three decimal places. All leave-one-out replicates were analyzed using the transition model selected from the full dataset. When *Kryptolebias marmoratus*, the only species scored as selfing, was treated as unknown, the Fertilization SYM model reached a boundary solution. Ancestral-state probabilities for this replicate were evaluated from the stable epsilon-limit of the SYM model as the boundary transition rates approached zero. For the SYM and ARD models, fitting was initialized from the ER-estimated transition rate plus 50–200 random starting points spanning ±3 orders of magnitude around it, and the fit with the highest log-likelihood was retained. Starting points (hits) = total number of starting points (number converging to the best log-likelihood within 0.001). Transition rates are listed in the order of the model's rate-matrix parameters.

Table S6. Eight gene family sequences used in Figure 3D.

No.	Tip label	Gene	Species	Clade	Protein accession	Nucleotide accession (CDS)	GeneID	Database	Protein length (aa)
1	Acipenser_ruthenus_acr	*ACR*	*Acipenser ruthenus*	Actinopterygii (non-teleost)	XP_033888599.1	XM_034032708.1	117419697	NCBI RefSeq	311
2	Callorhinchus_milii_acr	*ACR*	*Callorhinchus milii*	Chondrichthyes	XP_042191409.1	XM_042335475.1	103190823	NCBI RefSeq	303
3	Gallus_gallus_ACR	*ACR*	*Gallus gallus*	Tetrapoda	ENSGALP00010009779	ENSGALT00010017687.1	ENSGALG00010007422	Ensembl	354
4	Homo_sapiens_ACR	*ACR*	*Homo sapiens*	Tetrapoda	NP_001088.2	NM_001097.3	49	NCBI RefSeq	421
5	Latimeria_chalumnae_acr	*ACR*	*Latimeria chalumnae*	Sarcopterygii	XP_014352803.1	XM_014497317.2	102354559	NCBI RefSeq	277
6	Mus_musculus_ACR	*ACR*	*Mus musculus*	Tetrapoda	NP_038483.1	NM_013455.4	11434	NCBI RefSeq	436

7	Protopterus_annectens_acra_like	*ACR*	*Protopterus annectens*	Sarcopterygii	XP_043929688.1	XM_044073753.1	122804094	NCBI RefSeq	361
8	Protopterus_annectens_acrb_like	*ACR*	*Protopterus annectens*	Sarcopterygii	XP_043929583.1	XM_044073648.1	122803970	NCBI RefSeq	369
9	Xenopus_tropicalis_ACR	*ACR*	*Xenopus tropicalis*	Tetrapoda	ENSXETP00000066553	ENSXETT00000076375.2	ENSXETG00000017056	Ensembl	411
10	Acipenser_ruthenus_tmprss12	*TMPRSS12*	*Acipenser ruthenus*	Actinopterygii (non-teleost)	XP_033857193.3	XM_034001302.3	117400941	NCBI RefSeq	328
11	Callorhinchus_milii_tmprss12	*TMPRSS12*	*Callorhinchus milii*	Chondrichthyes	KI636116.1:384249-386894(+)	ENSCMIT00000012723	103189266	genome (miniprot)	303
12	Danio_rerio_tmprss12	*TMPRSS12*	*Danio rerio*	Teleostei	XP_068073014.2	XM_068216913.3	110438336	NCBI RefSeq	325
13	Erpetoichthys_calabaricus_tmprss12	*TMPRSS12*	*Erpetoichthys calabaricus*	Actinopterygii (non-teleost)	XP_028671351.1	XM_028815518.1	114662162	NCBI RefSeq	287
14	Gallus_gallus_TMPRSS12	*TMPRSS12*	*Gallus gallus*	Tetrapoda	XP_040549880.1	XM_040693946.2	426872	NCBI RefSeq	318
15	Gambusia_affinis_tmprss12	*TMPRSS12*	*Gambusia affinis*	Teleostei	XP_043983774.1	XM_044127839.1	122837479	NCBI RefSeq	302
16	Homo_sapiens_TMPRSS12	*TMPRSS12*	*Homo sapiens*	Tetrapoda	NP_872365.2	NM_182559.3	283471	NCBI RefSeq	348
17	Latimeria_chalumnae_tmprss12	*TMPRSS12*	*Latimeria chalumnae*	Sarcopterygii	XP_064408588.1	XM_064552518.1	102351894	NCBI RefSeq	306
18	Lepisosteus_oculatus_tmprss12	*TMPRSS12*	*Lepisosteus oculatus*	Actinopterygii (non-teleost)	XP_015199639.1	XM_015344153.2	102689718	NCBI RefSeq	311
19	Mus_musculus_TMPRSS12	*TMPRSS12*	*Mus musculus*	Tetrapoda	NP_898932.2	NM_183109.4	75002	NCBI RefSeq	336
20	Oryzias_latipes_tmprss12	*TMPRSS12*	*Oryzias latipes*	Teleostei	XP_004070473.3	XM_004070425.5	101163815	NCBI RefSeq	308
21	Poecilia_reticulata_tmprss12	*TMPRSS12*	*Poecilia reticulata*	Teleostei	XP_008411784.1	XM_008413562.2	103467305	NCBI RefSeq	301
22	Protopterus_annectens_tmprss12a_like	*TMPRSS12*	*Protopterus annectens*	Sarcopterygii	XP_043945246.1	XM_044089311.1	122816486	NCBI RefSeq	349

23	Protopterus_annectens_tmprss12 b_like	*TMPRSS12*	*Protopteru s annectens*	Sarcopteryg ii	XP_043926765.1	XM_044070830.1	122801279	NCBI RefSeq	278
24	Protopterus_annectens_tmprss12 c_like	*TMPRSS12*	*Protopteru s annectens*	Sarcopteryg ii	XP_043923925.1	XM_044067990.1	122798578	NCBI RefSeq	275
25	Xenopus_tropicalis_TMPRSS12	*TMPRSS12*	*Xenopus tropicalis*	Tetrapoda	XP_031751504.1	XM_031895644.1	100127698	NCBI RefSeq	324
26	Acipenser_ruthenus_TMPRSS6	*TMPRSS6*	*Acipenser ruthenus*	Actinoptery gii (non-teleost)	NW_026708340.1:5 1324-68668(+)	XM_034910745.1	117432912	genom e (minipr ot)	668
27	Callorhinchus_milii_TMPRSS6	*TMPRSS6*	*Callorhinc hus milii*	Chondricht hyes	ENSCMIP00000014 235	ENSCMIT00000014 540.1	ENSCMIG000000070 76	Ensem bl	800
28	Danio_rerio_TMPRSS6	*TMPRSS6*	*Danio rerio*	Teleostei	XP_087112432.1	XM_087256306.1	100005420	NCBI RefSeq	798
29	Erpetoichthys_calabaricus_TMP RSS6	*TMPRSS6*	*Erpetoicht hys calabaricu s*	Actinoptery gii (non-teleost)	ENSECRP00000023 965	ENSECRT00000024 490	ENSECRG000000162 30	Ensem bl	809
30	Gallus_gallus_TMPRSS6	*TMPRSS6*	*Gallus gallus*	Tetrapoda	ENSGALP00010020 649	ENSGALT00010035 537.1	ENSGALG00010014 778	Ensem bl	809
31	Gambusia_affinis_TMPRSS6	*TMPRSS6*	*Gambusia affinis*	Teleostei	LG05:26583569-26599694(+)	ENSPFOT00000004 728_R0	ENSPFOT000000047 28_R0-gene	genom e (minipr ot)	805
32	Homo_sapiens_TMPRSS6	*TMPRSS6*	*Homo sapiens*	Tetrapoda	ENSP00000501573	ENST00000676104.1	ENSG00000187045	Ensem bl	802
33	Latimeria_chalumnae_TMPRSS 6	*TMPRSS6*	*Latimeria chalumnae*	Sarcopteryg ii	ENSLACP00000007 263	ENSLACT00000007 323.1	ENSLACG00000006 442	Ensem bl	781
34	Lepisosteus_oculatus_TMPRSS6	*TMPRSS6*	*Lepisosteus oculatus*	Actinoptery gii (non-teleost)	ENSLOCP00000013 730	ENSLOCT00000013 759.1	ENSLOCG00000011 175	Ensem bl	837
35	Mus_musculus_TMPRSS6	*TMPRSS6*	*Mus musculus*	Tetrapoda	ENSMUSP00000155 401	ENSMUST00000230 226.2	ENSMUSG00000016 942	Ensem bl	799
36	Oryzias_latipes_TMPRSS6	*TMPRSS6*	*Oryzias latipes*	Teleostei	ENSORLP00000013 412	ENSORLT00000013 413.2	ENSORLG00000010 701	Ensem bl	803
37	Poecilia_reticulata_TMPRSS6	*TMPRSS6*	*Poecilia reticulata*	Teleostei	ENSPREP00000000 219	ENSPRET00000000 243.1	ENSPREG000000001 81	Ensem bl	827
38	Protopterus_annectens_TMPRSS	*TMPRSS6*	*Protopteru*	Sarcopteryg	NC_056725.1:15460	XM_044068021.1	122798955	genom	524

	6		*s annectens*	ii	71483-1546561223(-)			e (miniprot)	
39	Xenopus_tropicalis_TMPRSS6	*TMPRSS6*	*Xenopus tropicalis*	Tetrapoda	ENSXETP00000035000	ENSXETT00000035000.5	ENSXETG00000016040	Ensembl	806
40	Acipenser_ruthenus_ST14	*ST14*	*Acipenser ruthenus*	Actinopterygii (non-teleost)	NC_081229.1:645439-653367(-)	XM_033996290.2	117397427	genome (miniprot)	826
41	Callorhinchus_milii_ST14	*ST14*	*Callorhinchus milii*	Chondrichthyes	ENSCMIP00000009259	ENSCMIT00000009515.1	ENSCMIG00000004897	Ensembl	835
42	Danio_rerio_ST14	*ST14*	*Danio rerio*	Teleostei	ENSDARP00000131984	ENSDART00000165867.2	ENSDARG00000100088	Ensembl	841
43	Erpetoichthys_calabaricus_ST14	*ST14*	*Erpetoichthys calabaricus*	Actinopterygii (non-teleost)	ENSECRP00000026280	ENSECRT00000026826	ENSECRG00000017758	Ensembl	848
44	Gallus_gallus_ST14	*ST14*	*Gallus gallus*	Tetrapoda	ENSGALP00010034661	ENSGALT00010056997.1	ENSGALG00010023375	Ensembl	897
45	Gambusia_affinis_ST14	*ST14*	*Gambusia affinis*	Teleostei	LG03:17956265-17972060(-)	ENSPFOT00000005996_R0	ENSPFOT00000005996_R0-gene	genome (miniprot)	835
46	Homo_sapiens_ST14	*ST14*	*Homo sapiens*	Tetrapoda	ENSP00000278742	ENST00000278742.6	ENSG00000149418	Ensembl	855
47	Latimeria_chalumnae_ST14	*ST14*	*Latimeria chalumnae*	Sarcopterygii	ENSLACP00000020830	ENSLACT00000020970.1	ENSLACG00000018299	Ensembl	845
48	Lepisosteus_oculatus_ST14	*ST14*	*Lepisosteus oculatus*	Actinopterygii (non-teleost)	ENSLOCP00000004725	ENSLOCT00000004733.1	ENSLOCG00000003953	Ensembl	566
49	Mus_musculus_ST14	*ST14*	*Mus musculus*	Tetrapoda	ENSMUSP00000034478	ENSMUST00000034478.3	ENSMUSG00000031995	Ensembl	855
50	Oryzias_latipes_ST14	*ST14*	*Oryzias latipes*	Teleostei	ENSORLP00000008313	ENSORLT00000008314.2	ENSORLG00000006623	Ensembl	831
51	Poecilia_reticulata_ST14	*ST14*	*Poecilia reticulata*	Teleostei	ENSPREP00000004940	ENSPRET00000005008.1	ENSPREG00000003475	Ensembl	841
52	Xenopus_tropicalis_ST14	*ST14*	*Xenopus tropicalis*	Tetrapoda	ENSXETP00000082172	ENSXETT00000093563.2	ENSXETG00000017920	Ensembl	861

53	Acipenser_ruthenus_CORIN	*CORIN*	*Acipenser ruthenus*	Actinopterygii (non-teleost)	NC_081190.1:109398626-109437871(+)	XM_034035260.2	117421188	genome (miniprot)	841
54	Callorhinchus_milii_CORIN	*CORIN*	*Callorhinchus milii*	Chondrichthyes	ENSCMIP00000024603	ENSCMIT00000025012.1	ENSCMIG00000010880	Ensembl	976
55	Danio_rerio_CORIN	*CORIN*	*Danio rerio*	Teleostei	ENSDARP00000130128	ENSDART00000164080.2	ENSDARG00000101281	Ensembl	833
56	Erpetoichthys_calabaricus_CORIN	*CORIN*	*Erpetoichthys calabaricus*	Actinopterygii (non-teleost)	ENSECRP00000003570	ENSECRT00000003631	ENSECRG00000002380	Ensembl	1029
57	Gallus_gallus_CORIN	*CORIN*	*Gallus gallus*	Tetrapoda	ENSGALP00010019123	ENSGALT00010032345.1	ENSGALG00010013434	Ensembl	1130
58	Gambusia_affinis_CORIN	*CORIN*	*Gambusia affinis*	Teleostei	LG14:25561012-25572402(+)	ENSPFOT00000007208_R0	ENSPFOT00000007208_R0-gene	genome (miniprot)	844
59	Homo_sapiens_CORIN	*CORIN*	*Homo sapiens*	Tetrapoda	ENSP00000273857	ENST00000273857.9	ENSG00000145244	Ensembl	1042
60	Latimeria_chalumnae_CORIN	*CORIN*	*Latimeria chalumnae*	Sarcopterygii	ENSLACP00000000689	ENSLACT00000000695.1	ENSLACG00000000616	Ensembl	753
61	Lepisosteus_oculatus_CORIN	*CORIN*	*Lepisosteus oculatus*	Actinopterygii (non-teleost)	ENSLOCP00000014778	ENSLOCT00000014807.1	ENSLOCG00000012013	Ensembl	917
62	Mus_musculus_CORIN	*CORIN*	*Mus musculus*	Tetrapoda	ENSMUSP00000005352	ENSMUST00000005352.10	ENSMUSG00000005220	Ensembl	1113
63	Oryzias_latipes_CORIN	*CORIN*	*Oryzias latipes*	Teleostei	ENSORLP00000023715	ENSORLT00000023716.2	ENSORLG00000019007	Ensembl	721
64	Poecilia_formosa_CORIN	*CORIN*	*Poecilia formosa*	Teleostei	ENSPFOP00000007196	ENSPFOT00000007208.2	ENSPFOG00000006951	Ensembl	818
65	Protopterus_annectens_CORIN	*CORIN*	*Protopterus annectens*	Sarcopterygii	NC_056728.1:1235304995-1235909719(-)	XM_044061410.1	122793421	genome (miniprot)	753
66	Xenopus_tropicalis_CORIN	*CORIN*	*Xenopus tropicalis*	Tetrapoda	ENSXETP00000093076	ENSXETT00000070456.2	ENSXETG00000009837	Ensembl	1123
67	Acipenser_ruthenus_PRSS56	*PRSS56*	*Acipenser*	Actinopterygii (non-	NC_081200.1:26293			genome	870

			ruthenus	teleost)	9-271436(-)			(miniprot)	
68	Callorhinchus_milii_PRSS56	*PRSS56*	*Callorhinchus milii*	Chondrichthyes	ENSCMIP00000021112	ENSCMIT00000021496.1	ENSCMIG00000009677	Ensembl	415
69	Danio_rerio_PRSS56	*PRSS56*	*Danio rerio*	Teleostei	2:45044764-45070071(+)	ENSDART00000075152	ENSDARG00000053158	genome (miniprot)	858
70	Erpetoichthys_calabaricus_PRSS56	*PRSS56*	*Erpetoichthys calabaricus*	Actinopterygii (non-teleost)	2:183477242-183488442(+)	ENSECRT00000008252	ENSECRG00000005363	genome (miniprot)	828
71	Gallus_gallus_PRSS56	*PRSS56*	*Gallus gallus*	Tetrapoda	ENSGALP00010025646	ENSGALT00010043178.1	ENSGALG00010017861	Ensembl	795
72	Gambusia_affinis_PRSS56	*PRSS56*	*Gambusia affinis*	Teleostei	LG06:12176340-12183188(-)	ENSPFOT00000005496_R0	ENSPFOT00000005496_R0-gene	genome (miniprot)	897
73	Homo_sapiens_PRSS56	*PRSS56*	*Homo sapiens*	Tetrapoda	ENSP00000479745	ENST00000617714.2	ENSG00000237412	Ensembl	603
74	Latimeria_chalumnae_PRSS56	*PRSS56*	*Latimeria chalumnae*	Sarcopterygii	ENSLACP00000017718	ENSLACT00000017848.1	ENSLACG00000015605	Ensembl	477
75	Lepisosteus_oculatus_PRSS56	*PRSS56*	*Lepisosteus oculatus*	Actinopterygii (non-teleost)	ENSLOCP00000007095	ENSLOCT00000007103.1	ENSLOCG00000005875	Ensembl	578
76	Mus_musculus_PRSS56	*PRSS56*	*Mus musculus*	Tetrapoda	ENSMUSP00000138773	ENSMUST00000044533.9	ENSMUSG00000036480	Ensembl	604
77	Oryzias_latipes_PRSS56	*PRSS56*	*Oryzias latipes*	Teleostei	ENSORLP00000019708	ENSORLT00000019709.2	ENSORLG00000015737	Ensembl	879
78	Poecilia_reticulata_PRSS56	*PRSS56*	*Poecilia reticulata*	Teleostei	ENSPREP00000023973	ENSPRET00000024218.1	ENSPREG00000016176	Ensembl	912
79	Xenopus_tropicalis_PRSS56	*PRSS56*	*Xenopus tropicalis*	Tetrapoda	ENSXETP00000082378	ENSXETT00000104370.2	ENSXETG00000041361	Ensembl	786
80	Acipenser_ruthenus_TMPRSS7	*TMPRSS7*	*Acipenser ruthenus*	Actinopterygii (non-teleost)	NC_081196.1:2460646-2492517(-)	XM_034922698.1	117407156	genome (miniprot)	809
81	Callorhinchus_milii_TMPRSS7	*TMPRSS7*	*Callorhinc*	Chondricht	KI635896.1:3085465	ENSCMIT00000048	ENSCMIG000000197	genome	851

			hus milii	hyes	-3134337(-)	855	04	(miniprot)	
82	Danio_rerio_TMPRSS7	*TMPRSS7*	*Danio rerio*	Teleostei	24:21813147-21860412(-)	ENSDART00000189097	ENSDARG00000116575	genome (miniprot)	836
83	Erpetoichthys_calabaricus_TMPRSS7	*TMPRSS7*	*Erpetoichthys calabaricus*	Actinopterygii (non-teleost)	ENSECRP00000011272	ENSECRT00000011458	ENSECRG00000007492	Ensembl	849
84	Gallus_gallus_TMPRSS7	*TMPRSS7*	*Gallus gallus*	Tetrapoda	ENSGALP00010006143	ENSGALT00010010578.1	ENSGALG00010004545	Ensembl	827
85	Gambusia_affinis_TMPRSS7	*TMPRSS7*	*Gambusia affinis*	Teleostei	LG21:10906845-10922539(+)			genome (miniprot)	821
86	Homo_sapiens_TMPRSS7	*TMPRSS7*	*Homo sapiens*	Tetrapoda	ENSP00000398236	ENST00000452346.7	ENSG00000176040	Ensembl	843
87	Latimeria_chalumnae_TMPRSS7	*TMPRSS7*	*Latimeria chalumnae*	Sarcopterygii	ENSLACP00000004806	ENSLACT00000004847.1	ENSLACG00000004274	Ensembl	829
88	Lepisosteus_oculatus_TMPRSS7	*TMPRSS7*	*Lepisosteus oculatus*	Actinopterygii (non-teleost)	ENSLOCP00000005292	ENSLOCT00000005300.1	ENSLOCG00000004426	Ensembl	847
89	Mus_musculus_TMPRSS7	*TMPRSS7*	*Mus musculus*	Tetrapoda	ENSMUSP00000110209	ENSMUST00000114562.3	ENSMUSG00000033177	Ensembl	829
90	Oryzias_latipes_TMPRSS7	*TMPRSS7*	*Oryzias latipes*	Teleostei	20:17358885-17374076(+)	ENSORLT00000010938	ENSORLG00000008713	genome (miniprot)	801
91	Poecilia_reticulata_TMPRSS7	*TMPRSS7*	*Poecilia reticulata*	Teleostei	LG20:14774629-14791595(-)	ENSPRET00000029421	ENSPREG00000019688	genome (miniprot)	821
92	Protopterus_annectens_TMPRSS7	*TMPRSS7*	*Protopterus annectens*	Sarcopterygii	NC_056735.1:662058221-662505749(+)	XM_044072510.1	122802742	genome (miniprot)	816
93	Acipenser_ruthenus_TMPRSS9	*TMPRSS9*	*Acipenser ruthenus*	Actinopterygii (non-	NC_081235.1:6654011-6661832(-)	XM_034047778.2	117428824	genome	777

				teleost)				(miniprot)	
94	Callorhinchus_milii_TMPRSS9	*TMPRSS9*	*Callorhinchus milii*	Chondrichthyes	KI635896.1:3048336-3092008(-)	ENSCMIT00000048831	ENSCMIG00000019693	genome (miniprot)	592
95	Danio_rerio_TMPRSS9	*TMPRSS9*	*Danio rerio*	Teleostei	ENSDARP00000122978	ENSDART00000145551.4	ENSDARG00000029841	Ensembl	800
96	Erpetoichthys_calabaricus_TMPRSS9	*TMPRSS9*	*Erpetoichthys calabaricus*	Actinopterygii (non-teleost)	12:134557210-134574903(-)			genome (miniprot)	801
97	Gallus_gallus_TMPRSS9	*TMPRSS9*	*Gallus gallus*	Tetrapoda	ENSGALP00010042222	ENSGALT00010068710.1	ENSGALG00010028362	Ensembl	1127
98	Gambusia_affinis_TMPRSS9	*TMPRSS9*	*Gambusia affinis*	Teleostei	LG09:14724097-14729898(+)	ENSPFOT00000012462_R0	ENSPFOT00000012462_R0-gene	genome (miniprot)	789
99	Homo_sapiens_TMPRSS9	*TMPRSS9*	*Homo sapiens*	Tetrapoda	ENSP00000512457	ENST00000696167.1	ENSG00000178297	Ensembl	1093
100	Latimeria_chalumnae_TMPRSS9	*TMPRSS9*	*Latimeria chalumnae*	Sarcopterygii	ENSLACP00000017488	ENSLACT00000017617.1	ENSLACG00000015402	Ensembl	707
101	Lepisosteus_oculatus_TMPRSS9	*TMPRSS9*	*Lepisosteus oculatus*	Actinopterygii (non-teleost)	ENSLOCP00000005159	ENSLOCT00000005167.1	ENSLOCG00000004321	Ensembl	730
102	Mus_musculus_TMPRSS9	*TMPRSS9*	*Mus musculus*	Tetrapoda	ENSMUSP00000152034	ENSMUST00000219817.2	ENSMUSG00000059406	Ensembl	1099
103	Oryzias_latipes_TMPRSS9	*TMPRSS9*	*Oryzias latipes*	Teleostei	4:8259954-8269491(-)	ENSORLT00000029704	ENSORLG00000023312	genome (miniprot)	794
104	Poecilia_reticulata_TMPRSS9	*TMPRSS9*	*Poecilia reticulata*	Teleostei	LG4:28254026-28258385(+)			genome (miniprot)	453
105	Protopterus_annectens_TMPRSS9	*TMPRSS9*	*Protopterus annectens*	Sarcopterygii	NC_056747.1:60389111-60586494(+)	XM_044090621.1	122817581	genome (miniprot)	534

106	Xenopus_tropicalis_TMPRSS9	*TMPRSS9*	*Xenopus tropicalis*	Tetrapoda	ENSXETP00000003741	ENSXETT00000003741.5	ENSXETG00000001793	Ensembl	1151
107	Branchiostoma_lanceolatum_BL30580	trypsin-like	*Branchiostoma lanceolatum*	Outgroup	BL30580_evm0	BL30580_evm0	BL30580	Ensembl	781
108	Branchiostoma_lanceolatum_BL07494	trypsin-like	*Branchiostoma lanceolatum*	Outgroup	BL07494_evm0	BL07494_evm0	BL07494	Ensembl	653
109	Branchiostoma_lanceolatum_BL23320	trypsin-like	*Branchiostoma lanceolatum*	Outgroup	BL23320_cuf0	BL23320_cuf0	BL23320	Ensembl	542
110	Branchiostoma_lanceolatum_BL95919	trypsin-like	*Branchiostoma lanceolatum*	Outgroup	BL95919_evm1	BL95919_evm1	BL95919	Ensembl	496
111	Ciona_intestinalis_ENSCING00000001883	uncharacterized LOC100179863	*Ciona intestinalis*	Outgroup	ENSCINP00000003791.1	ENSCINT00000003791.1	ENSCING00000001883.1	Ensembl	519
112	Ciona_intestinalis_ENSCING00000001370	trypsin-like	*Ciona intestinalis*	Outgroup	ENSCINP00000002671.3	ENSCINT00000002671.3	ENSCING00000001370.3	Ensembl	496
113	Ciona_intestinalis_ENSCING00000007451	trypsin-like	*Ciona intestinalis*	Outgroup	ENSCINP00000015303.3	ENSCINT00000015303.3	ENSCING00000007451.3	Ensembl	397
114	Ciona_intestinalis_ENSCING00000002556	plasma kallikrein	*Ciona intestinalis*	Outgroup	ENSCINP00000005200.3	ENSCINT00000005200.3	ENSCING00000002556.3	Ensembl	389

Note: Publicly annotated proteins are identified by their database accessions where available. For loci for which the reference annotation model was incomplete or inconsistent with the inferred locus identity, a miniprot-derived protein model was reconstructed from the corresponding genomic region and used in subsequent phylogenetic analyses. For these entries, genomic coordinates are provided as the sequence identifier, while available CDS accessions and GeneIDs are retained in their respective columns. For loci lacking an annotated protein model, protein sequences were predicted directly from the genomic region using miniprot; these entries therefore have no corresponding CDS accession or GeneID. Identifiers for *Gambusia affinis* derived from the genome annotation of Shao et al. [1] are internal annotation identifiers rather than public database accessions.

Table S7. *ACR* and *TMPRSS12* sequences used in Figure 4B and 5A.

No.	Tip label	Gene	Species	Clade	Protein accession	Nucleotide accession (CDS)	GeneID	Database	Protein lengt

									**h (aa)**
1	Callorhinchus_milii_acr	*ACR*	*Callorhinchus milii*	Chondrichthyes	XP_042191409.1	XM_042335475.1	103190823	NCBI RefSeq	303
2	Hemiscyllium_ocellatum_acr	*ACR*	*Hemiscyllium ocellatum*	Chondrichthyes	XP_060698521.1	XM_060842538.1	132826581	NCBI RefSeq	329
3	Callorhinchus_milii_tmprss12	*TMPRSS12*	*Callorhinchus milii*	Chondrichthyes	XP_042200091.1	XM_042344157.1	103189266	NCBI RefSeq	243
4	Hemiscyllium_ocellatum_tmprss12	*TMPRSS12*	*Hemiscyllium ocellatum*	Chondrichthyes	XP_060679438.1	XM_060823455.1	132814268	NCBI RefSeq	314
5	Latimeria_chalumnae_acr	*ACR*	*Latimeria chalumnae*	Sarcopterygii	XP_014352803.1	XM_014497317.2	102354559	NCBI RefSeq	277
6	Protopterus_annectens_acrb_like	*ACR*	*Protopterus annectens*	Sarcopterygii	XP_043929583.1	XM_044073648.1	122803970	NCBI RefSeq	369
7	Protopterus_annectens_acra_like	*ACR*	*Protopterus annectens*	Sarcopterygii	XP_043929688.1	XM_044073753.1	122804094	NCBI RefSeq	361
8	Latimeria_chalumnae_tmprss12	*TMPRSS12*	*Latimeria chalumnae*	Sarcopterygii	XP_064408588.1	XM_064552518.1	102351894	NCBI RefSeq	306
9	Protopterus_annectens_tmprss12a_like	*TMPRSS12*	*Protopterus annectens*	Sarcopterygii	XP_043945246.1	XM_044089311.1	122816486	NCBI RefSeq	349
10	Protopterus_annectens_tmprss12c_like	*TMPRSS12*	*Protopterus annectens*	Sarcopterygii	XP_043923925.1	XM_044067990.1	122798578	NCBI RefSeq	275
11	Protopterus_annectens_tmprss12b_like	*TMPRSS12*	*Protopterus annectens*	Sarcopterygii	XP_043926765.1	XM_044070830.1	122801279	NCBI RefSeq	278
12	Acipenser_ruthenus_acr	*ACR*	*Acipenser ruthenus*	Actinopterygii (non-teleost)	XP_033888599.1	XM_034032708.1	117419697	NCBI RefSeq	311
13	Polyodon_spathula_acr	*ACR*	*Polyodon*	Actinopter	XP_041110585.1	XM_041254	121318214	NCBI	311

			spathula	ygii (non-teleost)		651.1		RefSeq	
14	Acipenser_ruthenus_tmprss12	*TMPRSS12*	*Acipenser ruthenus*	Actinopterygii (non-teleost)	XP_033857193.3	XM_034001302.3	117400941	NCBI RefSeq	328
15	Amia_ocellicauda_tmprss12	*TMPRSS12*	*Amia ocellicauda*	Actinopterygii (non-teleost)	XP_066564742.1	XM_066708645.1	136752922	NCBI RefSeq	343
16	Erpetoichthys_calabaricus_tmprss12	*TMPRSS12*	*Erpetoichthys calabaricus*	Actinopterygii (non-teleost)	XP_028671351.1	XM_028815518.1	114662162	NCBI RefSeq	287
17	Lepisosteus_oculatus_tmprss12	*TMPRSS12*	*Lepisosteus oculatus*	Actinopterygii (non-teleost)	XP_015199639.1	XM_015344153.2	102689718	NCBI RefSeq	311
18	Polyodon_spathula_tmprss12	*TMPRSS12*	*Polyodon spathula*	Actinopterygii (non-teleost)	XP_041097537.1	XM_041241603.1	121308889	NCBI RefSeq	183
19	Polypterus_senegalus_tmprss12	*TMPRSS12*	*Polypterus senegalus*	Actinopterygii (non-teleost)	XP_039620590.1	XM_039764656.1	120536330	NCBI RefSeq	287
20	Acanthochromis_polyacanthus_tmprss12	*TMPRSS12*	*Acanthochromis polyacanthus*	Teleostei	XP_051805717.1	XM_051949757.1	110958613	NCBI RefSeq	312
21	Acanthopagrus_latus_tmprss12	*TMPRSS12*	*Acanthopagrus latus*	Teleostei	XP_036960484.1	XM_037104589.1	119023012	NCBI RefSeq	302
22	Alosa_alosa_tmprss12	*TMPRSS12*	*Alosa alosa*	Teleostei	XP_048097844.1	XM_048241887.1	125293777	NCBI RefSeq	304
23	Alosa_pseudoharengus_tmprss12	*TMPRSS12*	*Alosa pseudoharengus*	Teleostei	XP_076133380.1	XM_076277265.1	143115294	NCBI RefSeq	210
24	Alosa_sapidissima_tmprss12	*TMPRSS12*	*Alosa sapidissima*	Teleostei	XP_041954116.1	XM_042098182.1	121713520	NCBI RefSeq	310
25	Ammodytes_marinus_tmprss12	*TMPRSS12*	*Ammodytes marinus*	Teleostei	XP_079887856.1	XM_080031730.1	307253617	NCBI RefSeq	281
26	Amphiprion_ocellaris_tmprss12	*TMPRSS12*	*Amphiprion*	Teleostei	XP_023123595.2	XM_023267	111566998	NCBI	303

			ocellaris			827.3		RefSeq	
27	Anabas_testudineus_tmprss12	*TMPRSS12*	*Anabas testudineus*	Teleostei	XP_026224446.1	XM_026368661.1	113167796	NCBI RefSeq	302
28	Anarhichas_minor_tmprss12	*TMPRSS12*	*Anarhichas minor*	Teleostei	XP_075958484.1	XM_076102369.1	142960508	NCBI RefSeq	298
29	Anarrhichthys_ocellatus_tmprss12	*TMPRSS12*	*Anarrhichthys ocellatus*	Teleostei	XP_031701702.1	XM_031845842.1	116382633	NCBI RefSeq	339
30	Anoplopoma_fimbria_tmprss12	*TMPRSS12*	*Anoplopoma fimbria*	Teleostei	XP_054464661.1	XM_054608686.1	129099446	NCBI RefSeq	312
31	Archocentrus_centrarchus_tmprss12	*TMPRSS12*	*Archocentrus centrarchus*	Teleostei	XP_030590474.1	XM_030734614.1	115783676	NCBI RefSeq	303
32	Astatotilapia_calliptera_tmprss12	*TMPRSS12*	*Astatotilapia calliptera*	Teleostei	XP_026008899.2	XM_026153114.2	113012796	NCBI RefSeq	304
33	Astyanax_mexicanus_tmprss12	*TMPRSS12*	*Astyanax mexicanus*	Teleostei	XP_022532596.2	XM_022676875.2	111194057	NCBI RefSeq	310
34	Aulonocara_stuartgranti_tmprss12	*TMPRSS12*	*Aulonocara stuartgranti*	Teleostei	XP_082374548.1	XM_082518422.1	309696901	NCBI RefSeq	283
35	Barbatula_barbatula_tmprss12	*TMPRSS12*	*Barbatula barbatula*	Teleostei	XP_079334395.1	XM_079478269.1	306906670	NCBI RefSeq	286
36	Boleophthalmus_pectinirostris_tmprss12	*TMPRSS12*	*Boleophthalmus pectinirostris*	Teleostei	XP_020785775.2	XM_020930116.2	110164970	NCBI RefSeq	306
37	Brachyhypopomus_gauderio_tmprss12	*TMPRSS12*	*Brachyhypopomus gauderio*	Teleostei	XP_076874693.1	XM_077018578.1	143525078	NCBI RefSeq	299
38	Brachyistius_frenatus_tmprss12	*TMPRSS12*	*Brachyistius frenatus*	Teleostei	XP_069545271.1	XM_069689170.1	138615502	NCBI RefSeq	315
39	Carassius_auratus_tmprss12	*TMPRSS12*	*Carassius*	Teleostei	XP_026099587.1	XM_026243	113070497	NCBI	317

			auratus			802.1		RefSeq	
40	Carassius_carassius_tmprss12	*TMPRSS12*	*Carassius carassius*	Teleostei	XP_059383024.1	XM_059527041.1	132117727	NCBI RefSeq	317
41	Centroberyx_affinis_tmprss12	*TMPRSS12*	*Centroberyx affinis*	Teleostei	XP_071379931.1	XM_071523830.1	139647846	NCBI RefSeq	277
42	Centroberyx_gerrardi_tmprss12	*TMPRSS12*	*Centroberyx gerrardi*	Teleostei	XP_078144212.1	XM_078288086.1	139927654	NCBI RefSeq	277
43	Centropristis_striata_tmprss12	*TMPRSS12*	*Centropristis striata*	Teleostei	XP_059189463.1	XM_059333480.1	131971840	NCBI RefSeq	303
44	Chaetodon_trifascialis_tmprss12	*TMPRSS12*	*Chaetodon trifascialis*	Teleostei	XP_070825140.1	XM_070969039.1	139335437	NCBI RefSeq	272
45	Channa_argus_tmprss12	*TMPRSS12*	*Channa argus*	Teleostei	XP_067382385.1	XM_067526284.1	137139271	NCBI RefSeq	214
46	Chanodichthys_erythropterus_tmprss12	*TMPRSS12*	*Chanodichthys erythropterus*	Teleostei	XP_067226606.1	XM_067370505.1	137008788	NCBI RefSeq	276
47	Chelmon_rostratus_tmprss12	*TMPRSS12*	*Chelmon rostratus*	Teleostei	XP_041800810.1	XM_041944876.1	121612172	NCBI RefSeq	303
48	Clinocottus_analis_tmprss12	*TMPRSS12*	*Clinocottus analis*	Teleostei	XP_068454289.1	XM_068598188.1	137800834	NCBI RefSeq	312
49	Clupea_harengus_tmprss12	*TMPRSS12*	*Clupea harengus*	Teleostei	XP_042563518.1	XM_042707584.1	122132859	NCBI RefSeq	307
50	Cololabis_saira_tmprss12	*TMPRSS12*	*Cololabis saira*	Teleostei	XP_061590840.1	XM_061734856.1	133456377	NCBI RefSeq	288
51	Coregonus_clupeaformis_tmprss12	*TMPRSS12*	*Coregonus clupeaformis*	Teleostei	XP_041701392.1	XM_041845458.1	121537852	NCBI RefSeq	307
52	Cottoperca_gobio_tmprss12	*TMPRSS12*	*Cottoperca*	Teleostei	XP_029291195.1	XM_029435	115010652	NCBI	301

			gobio			335.1		RefSe q	
53	Cyclopterus_lumpus_tmprss12	*TMPRSS12*	*Cyclopterus lumpus*	Teleostei	XP_034393578.1	XM_034537 687.1	117733795	NCBI RefSe q	304
54	Cyprinodon_tularosa_tmprss12	*TMPRSS12*	*Cyprinodon tularosa*	Teleostei	XP_038139067.1	XM_038283 139.1	119782251	NCBI RefSe q	303
55	Cyprinodon_variegatus_tmprss12	*TMPRSS12*	*Cyprinodon variegatus*	Teleostei	XP_015246617.1	XM_015391 131.1	107095153	NCBI RefSe q	274
56	Danio_aesculapii_tmprss12	*TMPRSS12*	*Danio aesculapii*	Teleostei	XP_056305583.1	XM_056449 608.1	130217497	NCBI RefSe q	304
57	Danio_rerio_tmprss12	*TMPRSS12*	*Danio rerio*	Teleostei	XP_068073014.2	XM_068216 913.3	110438336	NCBI RefSe q	325
58	Dicentrarchus_labrax_tmprss12	*TMPRSS12*	*Dicentrarchus labrax*	Teleostei	XP_051280929.1	XM_051424 969.1	127377248	NCBI RefSe q	322
59	Echeneis_naucrates_tmprss12	*TMPRSS12*	*Echeneis naucrates*	Teleostei	XP_029361305.1	XM_029505 445.1	115045659	NCBI RefSe q	303
60	Electrophorus_electricus_tmprss12	*TMPRSS12*	*Electrophorus electricus*	Teleostei	XP_081563375.1	XM_081707 249.1	113584438	NCBI RefSe q	429
61	Eleginops_maclovinus_tmprss12	*TMPRSS12*	*Eleginops maclovinus*	Teleostei	XP_063748004.1	XM_063891 934.1	134869934	NCBI RefSe q	308
62	Embiotoca_jacksoni_tmprss12	*TMPRSS12*	*Embiotoca jacksoni*	Teleostei	XP_068996675.1	XM_069140 574.1	138192894	NCBI RefSe q	285
63	Engraulis_encrasicolus_tmprss12	*TMPRSS12*	*Engraulis encrasicolus*	Teleostei	XP_063063791.1	XM_063207 721.1	134456351	NCBI RefSe q	309
64	Epinephelus_fuscoguttatus_tmprss12	*TMPRSS12*	*Epinephelus fuscoguttatus*	Teleostei	XP_049437088.1	XM_049581 131.1	125891696	NCBI RefSe q	303
65	Epinephelus_lanceolatus_tmprss12	*TMPRSS12*	*Epinephelus*	Teleostei	XP_078026538.1	XM_078170	117257780	NCBI	285

			lanceolatus			412.1		RefSeq	
66	Epinephelus_moara_tmprss12	*TMPRSS12*	*Epinephelus moara*	Teleostei	XP_049920016.1	XM_050064059.1	126402256	NCBI RefSeq	303
67	Esox_lucius_tmprss12	*TMPRSS12*	*Esox lucius*	Teleostei	XP_019907083.1	XM_020051524.2	109616400	NCBI RefSeq	148
68	Etheostoma_cragini_tmprss12	*TMPRSS12*	*Etheostoma cragini*	Teleostei	XP_034732948.1	XM_034877057.1	117947790	NCBI RefSeq	303
69	Etheostoma_spectabile_tmprss12	*TMPRSS12*	*Etheostoma spectabile*	Teleostei	XP_032378082.1	XM_032522191.1	116693303	NCBI RefSeq	303
70	Eucyclogobius_newberryi_tmprss12	*TMPRSS12*	*Eucyclogobius newberryi*	Teleostei	XP_072304720.1	XM_072448619.1	140362533	NCBI RefSeq	339
71	Fundulus_heteroclitus_tmprss12	*TMPRSS12*	*Fundulus heteroclitus*	Teleostei	XP_035992733.1	XM_036136840.1	105931750	NCBI RefSeq	304
72	Gadus_chalcogrammus_tmprss12	*TMPRSS12*	*Gadus chalcogrammus*	Teleostei	XP_056458495.1	XM_056602520.1	130392090	NCBI RefSeq	303
73	Gadus_macrocephalus_tmprss12	*TMPRSS12*	*Gadus macrocephalus*	Teleostei	XP_059906984.1	XM_060051001.1	132456590	NCBI RefSeq	304
74	Gadus_morhua_tmprss12	*TMPRSS12*	*Gadus morhua*	Teleostei	XP_030204094.1	XM_030348234.1	115536564	NCBI RefSeq	304
75	Gambusia_affinis_tmprss12	*TMPRSS12*	*Gambusia affinis*	Teleostei	XP_043983774.1	XM_044127839.1	122837479	NCBI RefSeq	302
76	Garra_rufa_tmprss12	*TMPRSS12*	*Garra rufa*	Teleostei	XP_073678803.1	XM_073822702.1	141290586	NCBI RefSeq	334
77	Gasterosteus_aculeatus_tmprss12	*TMPRSS12*	*Gasterosteus aculeatus*	Teleostei	XP_040048895.1	XM_040192961.2	120829120	NCBI RefSeq	312
78	Girardinichthys_multiradiatus_tmprss12	*TMPRSS12*	*Girardinichthy*	Teleostei	XP_047216337.1	XM_047360	124865361	NCBI	304

			s multiradiatus			381.1		RefSeq	
79	Gobio_gobio_tmprss12	*TMPRSS12*	*Gobio gobio*	Teleostei	XP_079658163.1	XM_079802037.1	307117245	NCBI RefSeq	279
80	Gobius_niger_tmprss12	*TMPRSS12*	*Gobius niger*	Teleostei	XP_079414516.1	XM_079558390.1	306964397	NCBI RefSeq	284
81	Gymnodraco_acuticeps_tmprss12	*TMPRSS12*	*Gymnodraco acuticeps*	Teleostei	XP_034065423.1	XM_034209532.1	117542059	NCBI RefSeq	305
82	Haplochromis_burtoni_tmprss12	*TMPRSS12*	*Haplochromis burtoni*	Teleostei	XP_042083945.1	XM_042228011.1	102310288	NCBI RefSeq	304
83	Hippoglossus_hippoglossus_tmprss12	*TMPRSS12*	*Hippoglossus hippoglossus*	Teleostei	XP_034447049.1	XM_034591158.1	117764972	NCBI RefSeq	235
84	Hoplias_malabaricus_tmprss12	*TMPRSS12*	*Hoplias malabaricus*	Teleostei	XP_066526486.1	XM_066670389.1	136696201	NCBI RefSeq	302
85	Hypomesus_transpacificus_tmprss12	*TMPRSS12*	*Hypomesus transpacificus*	Teleostei	XP_046895350.1	XM_047039394.1	124480265	NCBI RefSeq	282
86	Kryptolebias_marmoratus_tmprss12	*TMPRSS12*	*Kryptolebias marmoratus*	Teleostei	XP_024865063.1	XM_025009295.2	108244580	NCBI RefSeq	306
87	Larimichthys_crocea_tmprss12	*TMPRSS12*	*Larimichthys crocea*	Teleostei	XP_010746631.1	XM_010748329.3	104932980	NCBI RefSeq	301
88	Lates_calcarifer_tmprss12	*TMPRSS12*	*Lates calcarifer*	Teleostei	XP_018521354.1	XM_018665838.2	108876379	NCBI RefSeq	303
89	Leuresthes_tenuis_tmprss12	*TMPRSS12*	*Leuresthes tenuis*	Teleostei	XP_072223932.1	XM_072367831.1	140272399	NCBI RefSeq	303
90	Mastacembelus_armatus_tmprss12	*TMPRSS12*	*Mastacembelus armatus*	Teleostei	XP_026187415.1	XM_026331630.1	113145192	NCBI RefSeq	313
91	Maylandia_zebra_tmprss12	*TMPRSS12*	*Maylandia*	Teleostei	XP_004558758.2	XM_004558	101469494	NCBI	304

			zebra			701.2		RefSeq	
92	Megalobrama_amblycephala_tmprss12	*TMPRSS12*	*Megalobrama amblycephala*	Teleostei	XP_048033305.1	XM_048177348.1	125259604	NCBI RefSeq	302
93	Melanostigma_gelatinosum_tmprss12	*TMPRSS12*	*Melanostigma gelatinosum*	Teleostei	XP_079129246.1	XM_079273120.1	306780684	NCBI RefSeq	287
94	Melanotaenia_boesemani_tmprss12	*TMPRSS12*	*Melanotaenia boesemani*	Teleostei	XP_041859525.1	XM_042003591.1	121651411	NCBI RefSeq	365
95	Micropterus_dolomieu_tmprss12	*TMPRSS12*	*Micropterus dolomieu*	Teleostei	XP_045911558.1	XM_046055602.1	123974735	NCBI RefSeq	312
96	Monopterus_albus_tmprss12	*TMPRSS12*	*Monopterus albus*	Teleostei	XP_020471004.1	XM_020615348.1	109968813	NCBI RefSeq	303
97	Morone_saxatilis_tmprss12	*TMPRSS12*	*Morone saxatilis*	Teleostei	XP_035512861.1	XM_035656968.1	118324554	NCBI RefSeq	300
98	Mugil_cephalus_tmprss12	*TMPRSS12*	*Mugil cephalus*	Teleostei	XP_047437846.1	XM_047581890.1	125006130	NCBI RefSeq	303
99	Myripristis_murdjan_tmprss12	*TMPRSS12*	*Myripristis murdjan*	Teleostei	XP_029912345.1	XM_030056485.1	115362526	NCBI RefSeq	293
100	Myxocyprinus_asiaticus_tmprss12	*TMPRSS12*	*Myxocyprinus asiaticus*	Teleostei	XP_051515062.1	XM_051659102.1	127418512	NCBI RefSeq	287
101	Nansenia_antarctica_tmprss12	*TMPRSS12*	*Nansenia antarctica*	Teleostei	XP_080390707.1	XM_080534581.1	308160637	NCBI RefSeq	282
102	Nematolebias_whitei_tmprss12	*TMPRSS12*	*Nematolebias whitei*	Teleostei	XP_037540418.1	XM_037684490.1	119417282	NCBI RefSeq	362
103	Neolamprologus_brichardi_tmprss12	*TMPRSS12*	*Neolamprologus brichardi*	Teleostei	XP_006785657.1	XM_006785594.1	102793534	NCBI RefSeq	305
104	Nothobranchius_furzeri_tmprss12	*TMPRSS12*	*Nothobranchi*	Teleostei	XP_015823280.3	XM_015967	107390837	NCBI	305

			us furzeri			794.3		RefSe q	
105	Odontesthes_bonariensis_tmprss12	*TMPRSS12*	*Odontesthes bonariensis*	Teleostei	XP_075331614.1	XM_075475 499.1	142390129	NCBI RefSe q	303
106	Oncorhynchus_kisutch_tmprss12	*TMPRSS12*	*Oncorhynchus kisutch*	Teleostei	XP_020360478.1	XM_020504 889.2	109907108	NCBI RefSe q	284
107	Oncorhynchus_mykiss_tmprss12	*TMPRSS12*	*Oncorhynchus mykiss*	Teleostei	XP_021472837.2	XM_021617 162.2	110533035	NCBI RefSe q	284
108	Oncorhynchus_tshawytscha_tmprss12	*TMPRSS12*	*Oncorhynchus tshawytscha*	Teleostei	XP_024232385.1	XM_024376 617.2	112216629	NCBI RefSe q	284
109	Onychostoma_macrolepis_tmprss12	*TMPRSS12*	*Onychostoma macrolepis*	Teleostei	XP_058618406.1	XM_058762 423.1	131531581	NCBI RefSe q	301
110	Oreochromis_aureus_tmprss12	*TMPRSS12*	*Oreochromis aureus*	Teleostei	XP_031602829.1	XM_031746 969.2	116325904	NCBI RefSe q	305
111	Oreochromis_niloticus_tmprss12	*TMPRSS12*	*Oreochromis niloticus*	Teleostei	XP_019204871.1	XM_019349 326.2	100702191	NCBI RefSe q	312
112	Oryzias_latipes_tmprss12	*TMPRSS12*	*Oryzias latipes*	Teleostei	XP_004070473.3	XM_004070 425.5	101163815	NCBI RefSe q	308
113	Oryzias_melastigma_tmprss12	*TMPRSS12*	*Oryzias melastigma*	Teleostei	XP_024148676.1	XM_024292 908.1	112159072	NCBI RefSe q	312
114	Osmerus_eperlanus_tmprss12	*TMPRSS12*	*Osmerus eperlanus*	Teleostei	XP_062314080.1	XM_062458 096.1	134018216	NCBI RefSe q	307
115	Osmerus_mordax_tmprss12	*TMPRSS12*	*Osmerus mordax*	Teleostei	XP_067095085.1	XM_067238 984.1	136945239	NCBI RefSe q	282
116	Pagrus_major_tmprss12	*TMPRSS12*	*Pagrus major*	Teleostei	XP_073327308.1	XM_073471 207.1	141000461	NCBI RefSe q	301
117	Pelmatolapia_mariae_tmprss12	*TMPRSS12*	*Pelmatolapia*	Teleostei	XP_063319126.1	XM_063463	134617961	NCBI	187

			mariae			056.1		RefSeq	
118	Perca_flavescens_tmprss12	*TMPRSS12*	*Perca flavescens*	Teleostei	XP_028438328.1	XM_028582527.1	114558485	NCBI RefSeq	304
119	Perca_fluviatilis_tmprss12	*TMPRSS12*	*Perca fluviatilis*	Teleostei	XP_039655464.1	XM_039799530.1	120558509	NCBI RefSeq	304
120	Periophthalmus_magnuspinnatus_tmprss12	*TMPRSS12*	*Periophthalmus magnuspinnatus*	Teleostei	XP_033824791.1	XM_033968900.1	117373043	NCBI RefSeq	290
121	Pholis_gunnellus_tmprss12	*TMPRSS12*	*Pholis gunnellus*	Teleostei	XP_079032255.1	XM_079176129.1	306708323	NCBI RefSeq	280
122	Phoxinus_phoxinus_tmprss12	*TMPRSS12*	*Phoxinus phoxinus*	Teleostei	XP_079444976.1	XM_079588850.1	306986018	NCBI RefSeq	285
123	Plectropomus_leopardus_tmprss12	*TMPRSS12*	*Plectropomus leopardus*	Teleostei	XP_042347115.1	XM_042491181.1	121946563	NCBI RefSeq	302
124	Pleuronectes_platessa_tmprss12	*TMPRSS12*	*Pleuronectes platessa*	Teleostei	XP_053280129.1	XM_053424154.1	128442004	NCBI RefSeq	266
125	Poecilia_formosa_tmprss12	*TMPRSS12*	*Poecilia formosa*	Teleostei	XP_016529116.1	XM_016673630.1	103141210	NCBI RefSeq	302
126	Poecilia_latipinna_tmprss12	*TMPRSS12*	*Poecilia latipinna*	Teleostei	XP_014893528.1	XM_015038042.1	106950882	NCBI RefSeq	302
127	Poecilia_mexicana_tmprss12	*TMPRSS12*	*Poecilia mexicana*	Teleostei	XP_014837730.1	XM_014982244.1	106914976	NCBI RefSeq	301
128	Poecilia_reticulata_tmprss12	*TMPRSS12*	*Poecilia reticulata*	Teleostei	XP_008411784.1	XM_008413562.2	103467305	NCBI RefSeq	301
129	Poeciliopsis_prolifica_tmprss12	*TMPRSS12*	*Poeciliopsis prolifica*	Teleostei	XP_054905442.1	XM_055049467.1	129372289	NCBI RefSeq	301

130	Pollachius_pollachius_tmprss12	*TMPRSS12*	*Pollachius pollachius*	Teleostei	XP_079598191.1	XM_079742065.1	307079624	NCBI RefSeq	304
131	Pseudochaenichthys_georgianus_tmprss12	*TMPRSS12*	*Pseudochaenichthys georgianus*	Teleostei	XP_033944035.1	XM_034088144.2	117450110	NCBI RefSeq	305
132	Pseudoliparis_swirei_tmprss12	*TMPRSS12*	*Pseudoliparis swirei*	Teleostei	XP_056279444.1	XM_056423469.1	130199739	NCBI RefSeq	307
133	Pseudorasbora_parva_tmprss12	*TMPRSS12*	*Pseudorasbora parva*	Teleostei	XP_067303553.1	XM_067447452.1	137079499	NCBI RefSeq	291
134	Pundamilia_nyererei_tmprss12	*TMPRSS12*	*Pundamilia nyererei*	Teleostei	XP_005732702.1	XM_005732645.1	102192393	NCBI RefSeq	304
135	Pungitius_pungitius_tmprss12	*TMPRSS12*	*Pungitius pungitius*	Teleostei	XP_037334610.1	XM_037478713.2	119222241	NCBI RefSeq	323
136	Rhamphochromis_sp._chilingali_tmprss12	*TMPRSS12*	*Rhamphochromis sp. chilingali*	Teleostei	XP_080753796.1	XM_080897670.1	308406590	NCBI RefSeq	282
137	Salarias_fasciatus_tmprss12	*TMPRSS12*	*Salarias fasciatus*	Teleostei	XP_029973655.1	XM_030117795.1	115407448	NCBI RefSeq	299
138	Salminus_brasiliensis_tmprss12	*TMPRSS12*	*Salminus brasiliensis*	Teleostei	XP_072553532.1	XM_072697431.1	140577446	NCBI RefSeq	398
139	Salmo_trutta_tmprss12	*TMPRSS12*	*Salmo trutta*	Teleostei	XP_029576225.1	XM_029720365.1	115166466	NCBI RefSeq	282
140	Salvelinus_alpinus_tmprss12	*TMPRSS12*	*Salvelinus alpinus*	Teleostei	XP_071229023.1	XM_071372922.1	139557759	NCBI RefSeq	287
141	Salvelinus_sp._IW2-2015_tmprss12	*TMPRSS12*	*Salvelinus sp. IW2-2015*	Teleostei	XP_023846297.1	XM_023990529.2	111966107	NCBI RefSeq	286
142	Sander_lucioperca_tmprss12	*TMPRSS12*	*Sander lucioperca*	Teleostei	XP_031140199.1	XM_031284339.2	116039453	NCBI RefSeq	303

143	Sander_vitreus_tmprss12	*TMPRSS12*	*Sander vitreus*	Teleostei	XP_078110078.1	XM_078253952.1	144520285	NCBI RefSeq	303
144	Sardina_pilchardus_tmprss12	*TMPRSS12*	*Sardina pilchardus*	Teleostei	XP_062397262.1	XM_062541278.1	134087654	NCBI RefSeq	303
145	Scatophagus_argus_tmprss12	*TMPRSS12*	*Scatophagus argus*	Teleostei	XP_046253279.1	XM_046397323.1	124063551	NCBI RefSeq	303
146	Scomber_japonicus_tmprss12	*TMPRSS12*	*Scomber japonicus*	Teleostei	XP_053173926.1	XM_053317951.1	128357589	NCBI RefSeq	297
147	Scomber_scombrus_tmprss12	*TMPRSS12*	*Scomber scombrus*	Teleostei	XP_062283073.1	XM_062427089.1	133987645	NCBI RefSeq	323
148	Scophthalmus_maximus_tmprss12	*TMPRSS12*	*Scophthalmus maximus*	Teleostei	XP_035500582.1	XM_035644689.2	118316655	NCBI RefSeq	345
149	Seriola_aureovittata_tmprss12	*TMPRSS12*	*Seriola aureovittata*	Teleostei	XP_056240454.1	XM_056384479.1	130174554	NCBI RefSeq	344
150	Seriola_dumerili_tmprss12	*TMPRSS12*	*Seriola dumerili*	Teleostei	XP_022597547.1	XM_022741826.1	111219213	NCBI RefSeq	259
151	Simochromis_diagramma_tmprss12	*TMPRSS12*	*Simochromis diagramma*	Teleostei	XP_039903566.1	XM_040047632.1	120743782	NCBI RefSeq	304
152	Siniperca_chuatsi_tmprss12	*TMPRSS12*	*Siniperca chuatsi*	Teleostei	XP_044068319.1	XM_044212384.1	122883548	NCBI RefSeq	303
153	Sinocyclocheilus_anshuiensis_tmprss12	*TMPRSS12*	*Sinocyclocheilus anshuiensis*	Teleostei	XP_016351272.1	XM_016495786.1	107695755	NCBI RefSeq	153
154	Sinocyclocheilus_grahami_tmprss12	*TMPRSS12*	*Sinocyclocheilus grahami*	Teleostei	XP_016104048.1	XM_016248562.1	107563642	NCBI RefSeq	156
155	Sinocyclocheilus_rhinocerous_tmprss12	*TMPRSS12*	*Sinocyclocheilus rhinocerous*	Teleostei	XP_016377879.1	XM_016522393.1	107716074	NCBI RefSeq	157

156	Siphateles_boraxobius_tmprss12	*TMPRSS12*	*Siphateles boraxobius*	Teleostei	XP_077075180.1	XM_077219065.1	143726077	NCBI RefSeq	291
157	Sparus_aurata_tmprss12	*TMPRSS12*	*Sparus aurata*	Teleostei	XP_030279604.1	XM_030423744.1	115585404	NCBI RefSeq	308
158	Sphaeramia_orbicularis_tmprss12	*TMPRSS12*	*Sphaeramia orbicularis*	Teleostei	XP_029994680.1	XM_030138820.1	115422467	NCBI RefSeq	306
159	Sprattus_sprattus_tmprss12	*TMPRSS12*	*Sprattus sprattus*	Teleostei	XP_080688521.1	XM_080832395.1	308360464	NCBI RefSeq	307
160	Stegastes_partitus_tmprss12	*TMPRSS12*	*Stegastes partitus*	Teleostei	XP_008294832.1	XM_008296610.1	103368282	NCBI RefSeq	304
161	Taurulus_bubalis_tmprss12	*TMPRSS12*	*Taurulus bubalis*	Teleostei	XP_079081936.1	XM_079225810.1	306743306	NCBI RefSeq	282
162	Telmatherina_bonti_tmprss12	*TMPRSS12*	*Telmatherina bonti*	Teleostei	XP_079094621.1	XM_079238495.1	306754476	NCBI RefSeq	285
163	Thalassophryne_amazonica_tmprss12	*TMPRSS12*	*Thalassophryne amazonica*	Teleostei	XP_034028215.1	XM_034172324.1	117512305	NCBI RefSeq	300
164	Thunnus_albacares_tmprss12	*TMPRSS12*	*Thunnus albacares*	Teleostei	XP_044207393.1	XM_044351458.1	122982271	NCBI RefSeq	245
165	Thunnus_maccoyii_tmprss12	*TMPRSS12*	*Thunnus maccoyii*	Teleostei	XP_042265064.1	XM_042409130.1	121895709	NCBI RefSeq	287
166	Thunnus_thynnus_tmprss12	*TMPRSS12*	*Thunnus thynnus*	Teleostei	XP_067449710.1	XM_067593609.1	137185262	NCBI RefSeq	263
167	Toxotes_jaculatrix_tmprss12	*TMPRSS12*	*Toxotes jaculatrix*	Teleostei	XP_040908358.1	XM_041052424.1	121191254	NCBI RefSeq	313
168	Trachinotus_anak_tmprss12	*TMPRSS12*	*Trachinotus anak*	Teleostei	XP_071332200.1	XM_071476099.1	139620494	NCBI RefSeq	327

169	Trematomus_bernacchii_tmprss12	*TMPRSS12*	*Trematomus bernacchii*	Teleostei	XP_034001016.1	XM_034145<br>125.1	117494286	NCBI<br>RefSe<br>q	308
170	Trichomycterus_rosablanca_tmprss12	*TMPRSS12*	*Trichomycteru s rosablanca*	Teleostei	XP_062872080.1	XM_063016<br>010.1	134333843	NCBI<br>RefSe<br>q	289
171	Triplophysa_dalaica_tmprss12	*TMPRSS12*	*Triplophysa dalaica*	Teleostei	XP_056589352.1	XM_056733<br>374.1	130409409	NCBI<br>RefSe<br>q	302
172	Triplophysa_rosa_tmprss12	*TMPRSS12*	*Triplophysa rosa*	Teleostei	XP_057175748.1	XM_057319<br>765.1	130545326	NCBI<br>RefSe<br>q	237
173	Xiphias_gladius_tmprss12	*TMPRSS12*	*Xiphias gladius*	Teleostei	XP_040007518.1	XM_040151<br>584.1	120803268	NCBI<br>RefSe<br>q	341
174	Xiphophorus_couchianus_tmprss12	*TMPRSS12*	*Xiphophorus couchianus*	Teleostei	XP_027876438.1	XM_028020<br>637.1	114146514	NCBI<br>RefSe<br>q	302
175	Xiphophorus_hellerii_tmprss12	*TMPRSS12*	*Xiphophorus hellerii*	Teleostei	XP_032429549.1	XM_032573<br>658.1	116726771	NCBI<br>RefSe<br>q	302
176	Xiphophorus_maculatus_tmprss12	*TMPRSS12*	*Xiphophorus maculatus*	Teleostei	XP_005801464.3	XM_005801<br>407.3	102234008	NCBI<br>RefSe<br>q	329
177	Xyrauchen_texanus_tmprss12	*TMPRSS12*	*Xyrauchen texanus*	Teleostei	XP_051961306.1	XM_052105<br>346.1	127628568	NCBI<br>RefSe<br>q	240
178	Acinonyx_jubatus_ACR	*ACR*	*Acinonyx jubatus*	Tetrapoda	XP_014941408.2	XM_015085<br>922.3	106987633	NCBI<br>RefSe<br>q	423
179	Acomys_russatus_ACR	*ACR*	*Acomys russatus*	Tetrapoda	XP_051016177.1	XM_051160<br>220.1	127201561	NCBI<br>RefSe<br>q	444
180	Ailuropoda_melanoleuca_ACR	*ACR*	*Ailuropoda melanoleuca*	Tetrapoda	XP_019653068.2	XM_019797<br>509.2	100482745	NCBI<br>RefSe<br>q	350
181	Anas_acuta_ACR	*ACR*	*Anas acuta*	Tetrapoda	XP_068526453.1	XM_068670<br>352.1	137850220	NCBI<br>RefSe<br>q	923

182	Anser_cygnoides_ACR	*ACR*	*Anser cygnoides*	Tetrapoda	XP_066846655.1	XM_066990554.1	106029620	NCBI RefSeq	713
183	Aotus_nancymaae_ACR	*ACR*	*Aotus nancymaae*	Tetrapoda	XP_012292656.1	XM_012437233.2	105706695	NCBI RefSeq	413
184	Apodemus_sylvaticus_ACR	*ACR*	*Apodemus sylvaticus*	Tetrapoda	XP_052016875.1	XM_052160915.1	127667705	NCBI RefSeq	435
185	Aptenodytes_forsteri_ACR	*ACR*	*Aptenodytes forsteri*	Tetrapoda	XP_009273116.2	XM_009274841.2	103895289	NCBI RefSeq	345
186	Aptenodytes_patagonicus_ACR	*ACR*	*Aptenodytes patagonicus*	Tetrapoda	XP_076184683.1	XM_076328568.1	143155598	NCBI RefSeq	430
187	Aquila_chrysaetos_chrysaetos_ACR	*ACR*	*Aquila chrysaetos chrysaetos*	Tetrapoda	XP_029872086.1	XM_030016226.1	115342214	NCBI RefSeq	347
188	Artibeus_jamaicensis_ACR	*ACR*	*Artibeus jamaicensis*	Tetrapoda	XP_037016332.2	XM_037160437.2	119060332	NCBI RefSeq	440
189	Arvicanthis_niloticus_ACR	*ACR*	*Arvicanthis niloticus*	Tetrapoda	XP_034372902.1	XM_034517011.2	117718981	NCBI RefSeq	438
190	Arvicola_amphibius_ACR	*ACR*	*Arvicola amphibius*	Tetrapoda	XP_038199117.1	XM_038343189.1	119823273	NCBI RefSeq	436
191	Astur_gentilis_ACR	*ACR*	*Astur gentilis*	Tetrapoda	XP_049669539.1	XM_049813582.1	126044256	NCBI RefSeq	347
192	Athene_noctua_ACR	*ACR*	*Athene noctua*	Tetrapoda	XP_074757793.1	XM_074901692.1	141958804	NCBI RefSeq	478
193	Balaenoptera_acutorostrata_ACR	*ACR*	*Balaenoptera acutorostrata*	Tetrapoda	XP_057413322.1	XM_057557339.1	103017313	NCBI RefSeq	418
194	Balaenoptera_borealis_ACR	*ACR*	*Balaenoptera borealis*	Tetrapoda	XP_082206442.1	XM_082350316.1	309609176	NCBI RefSeq	414

195	Balaenoptera_musculus_ACR	*ACR*	*Balaenoptera musculus*	Tetrapoda	XP_036723300.1	XM_036867405.1	118902739	NCBI RefSeq	420
196	Balaenoptera_physalus_ACR	*ACR*	*Balaenoptera physalus*	Tetrapoda	XP_082277282.1	XM_082421156.1	309641303	NCBI RefSeq	429
197	Balaenoptera_ricei_ACR	*ACR*	*Balaenoptera ricei*	Tetrapoda	XP_059790897.1	XM_059934914.1	132372774	NCBI RefSeq	414
198	Bison_bison_bison_ACR	*ACR*	*Bison bison bison*	Tetrapoda	XP_010826864.1	XM_010828562.1	104980012	NCBI RefSeq	414
199	Bos_indicus_ACR	*ACR*	*Bos indicus*	Tetrapoda	XP_070646813.1	XM_070790712.1	139183317	NCBI RefSeq	414
200	Bos_indicus_x_Bos_taurus_ACR	*ACR*	*Bos indicus x Bos taurus*	Tetrapoda	XP_027396179.1	XM_027540378.1	113892047	NCBI RefSeq	414
201	Bos_javanicus_ACR	*ACR*	*Bos javanicus*	Tetrapoda	XP_061274980.1	XM_061418996.1	133248604	NCBI RefSeq	414
202	Bos_mutus_ACR	*ACR*	*Bos mutus*	Tetrapoda	XP_070227469.1	XM_070371368.1	102270129	NCBI RefSeq	414
203	Bos_taurus_ACR	*ACR*	*Bos taurus*	Tetrapoda	NP_776311.2	NM_173886.2	280711	NCBI RefSeq	414
204	Branta_canadensis_ACR	*ACR*	*Branta canadensis*	Tetrapoda	XP_081891949.1	XM_082035823.1	309430033	NCBI RefSeq	332
205	Bubalus_bubalis_ACR	*ACR*	*Bubalus bubalis*	Tetrapoda	XP_025138352.2	XM_025282567.3	102391015	NCBI RefSeq	414
206	Bubalus_kerabau_ACR	*ACR*	*Bubalus kerabau*	Tetrapoda	XP_055421967.1	XM_055565992.1	129641264	NCBI RefSeq	414
207	Budorcas_taxicolor_ACR	*ACR*	*Budorcas taxicolor*	Tetrapoda	XP_052497467.1	XM_052641507.1	128049003	NCBI RefSeq	413

208	Calidris_pugnax_ACR	*ACR*	*Calidris pugnax*	Tetrapoda	XP_014817446.1	XM_014961960.1	106900005	NCBI RefSeq	359
209	Callithrix_jacchus_ACR	*ACR*	*Callithrix jacchus*	Tetrapoda	XP_035130765.2	XM_035274874.3	100406660	NCBI RefSeq	402
210	Callorhinus_ursinus_ACR	*ACR*	*Callorhinus ursinus*	Tetrapoda	XP_025714454.1	XM_025858669.2	112813629	NCBI RefSeq	412
211	Callospermophilus_lateralis_ACR	*ACR*	*Callospermophilus lateralis*	Tetrapoda	XP_076711691.1	XM_076855576.2	143398648	NCBI RefSeq	482
212	Camelus_bactrianus_ACR	*ACR*	*Camelus bactrianus*	Tetrapoda	XP_010955632.3	XM_010957330.3	105070825	NCBI RefSeq	409
213	Camelus_dromedarius_ACR	*ACR*	*Camelus dromedarius*	Tetrapoda	XP_010998282.3	XM_010999980.3	105106149	NCBI RefSeq	424
214	Camelus_ferus_ACR	*ACR*	*Camelus ferus*	Tetrapoda	XP_032348538.1	XM_032492647.1	102504353	NCBI RefSeq	409
215	Canis_aureus_ACR	*ACR*	*Canis aureus*	Tetrapoda	XP_077770036.1	XM_077913910.1	144323457	NCBI RefSeq	414
216	Canis_lupus_baileyi_ACR	*ACR*	*Canis lupus baileyi*	Tetrapoda	XP_072699450.1	XM_072843349.1	140642580	NCBI RefSeq	414
217	Canis_lupus_dingo_ACR	*ACR*	*Canis lupus dingo*	Tetrapoda	XP_025310707.1	XM_025454922.3	112665305	NCBI RefSeq	414
218	Canis_lupus_familiaris_ACR	*ACR*	*Canis lupus familiaris*	Tetrapoda	XP_038469988.1	XM_038614060.1	606765	NCBI RefSeq	415
219	Capra_hircus_ACR	*ACR*	*Capra hircus*	Tetrapoda	XP_017904493.1	XM_018049004.1	102191416	NCBI RefSeq	413
220	Capricornis_sumatraensis_ACR	*ACR*	*Capricornis sumatraensis*	Tetrapoda	XP_068828324.1	XM_068972223.1	138079329	NCBI RefSeq	413

221	Caprimulgus_europaeus_ACR	*ACR*	*Caprimulgus europaeus*	Tetrapoda	XP_079047355.1	XM_079191229.1	306718769	NCBI RefSeq	347
222	Caretta_caretta_ACR	*ACR*	*Caretta caretta*	Tetrapoda	XP_074980740.1	XM_075124639.1	125625502	NCBI RefSeq	355
223	Carettochelys_insculpta_ACR	*ACR*	*Carettochelys insculpta*	Tetrapoda	XP_074840359.1	XM_074984258.1	142007570	NCBI RefSeq	362
224	Carlito_syrichta_ACR	*ACR*	*Carlito syrichta*	Tetrapoda	XP_008067165.1	XM_008068974.2	103271503	NCBI RefSeq	417
225	Castor_canadensis_ACR	*ACR*	*Castor canadensis*	Tetrapoda	XP_020029715.2	XM_020174126.2	109693070	NCBI RefSeq	442
226	Cavia_porcellus_ACR	*ACR*	*Cavia porcellus*	Tetrapoda	XP_013009008.3	XM_013153554.3	100717414	NCBI RefSeq	447
227	Cebus_imitator_ACR	*ACR*	*Cebus imitator*	Tetrapoda	XP_017360461.1	XM_017504972.2	108286851	NCBI RefSeq	417
228	Ceratotherium_simum_simum_ACR	*ACR*	*Ceratotherium simum simum*	Tetrapoda	XP_004438114.1	XM_004438057.3	101403171	NCBI RefSeq	404
229	Cervus_canadensis_ACR	*ACR*	*Cervus canadensis*	Tetrapoda	XP_043295719.1	XM_043439784.1	122423039	NCBI RefSeq	414
230	Cervus_elaphus_ACR	*ACR*	*Cervus elaphus*	Tetrapoda	XP_043736510.1	XM_043880575.1	122679818	NCBI RefSeq	414
231	Chaetura_pelagica_ACR	*ACR*	*Chaetura pelagica*	Tetrapoda	XP_009998622.1	XM_010000320.1	104391094	NCBI RefSeq	345
232	Chelonia_mydas_ACR	*ACR*	*Chelonia mydas*	Tetrapoda	XP_027684823.3	XM_027829022.3	102938344	NCBI RefSeq	355
233	Chinchilla_lanigera_ACR	*ACR*	*Chinchilla lanigera*	Tetrapoda	XP_005379655.2	XM_005379598.2	102009368	NCBI RefSeq	452

234	Chionomys_nivalis_ACR	*ACR*	*Chionomys nivalis*	Tetrapoda	XP_057648351.1	XM_057792368.1	130888961	NCBI RefSeq	440
235	Chlorocebus_sabaeus_ACR	*ACR*	*Chlorocebus sabaeus*	Tetrapoda	XP_037863150.1	XM_038007222.2	103223579	NCBI RefSeq	419
236	Choloepus_didactylus_ACR	*ACR*	*Choloepus didactylus*	Tetrapoda	XP_037704217.1	XM_037848289.1	119543455	NCBI RefSeq	486
237	Chroicocephalus_ridibundus_ACR	*ACR*	*Chroicocephalus ridibundus*	Tetrapoda	XP_063179890.1	XM_063323820.1	134510472	NCBI RefSeq	343
238	Chrysemys_picta_bellii_ACR	*ACR*	*Chrysemys picta bellii*	Tetrapoda	XP_042715942.2	XM_042860008.2	101943992	NCBI RefSeq	505
239	Chrysochloris_asiatica_ACR	*ACR*	*Chrysochloris asiatica*	Tetrapoda	XP_006876466.1	XM_006876404.1	102830826	NCBI RefSeq	410
240	Cnephaeus_nilssonii_ACR	*ACR*	*Cnephaeus nilssonii*	Tetrapoda	XP_079631603.1	XM_079775477.1	307102008	NCBI RefSeq	423
241	Colobus_angolensis_palliatus_ACR	*ACR*	*Colobus angolensis palliatus*	Tetrapoda	XP_011797647.1	XM_011942257.1	105512002	NCBI RefSeq	326
242	Condylura_cristata_ACR	*ACR*	*Condylura cristata*	Tetrapoda	XP_012590522.1	XM_012735068.1	101631790	NCBI RefSeq	466
243	Coturnix_japonica_ACR	*ACR*	*Coturnix japonica*	Tetrapoda	XP_015734463.1	XM_015878977.2	107319775	NCBI RefSeq	354
244	Cricetulus_griseus_ACR	*ACR*	*Cricetulus griseus*	Tetrapoda	XP_035293549.1	XM_035437658.1	100754856	NCBI RefSeq	438
245	Crocuta_crocuta_ACR	*ACR*	*Crocuta crocuta*	Tetrapoda	XP_077614005.1	XM_077757879.1	144226478	NCBI RefSeq	361
246	Cynocephalus_volans_ACR	*ACR*	*Cynocephalus volans*	Tetrapoda	XP_062932251.1	XM_063076181.1	134361196	NCBI RefSeq	385

247	Dama_dama_ACR	*ACR*	*Dama dama*	Tetrapoda	XP_060980139.1	XM_061124156.1	133043338	NCBI RefSeq	414
248	Dasypus_novemcinctus_ACR	*ACR*	*Dasypus novemcinctus*	Tetrapoda	XP_004470406.1	XM_004470349.2	101443016	NCBI RefSeq	412
249	Delphinapterus_leucas_ACR	*ACR*	*Delphinapterus leucas*	Tetrapoda	XP_022452635.1	XM_022596927.2	111186384	NCBI RefSeq	410
250	Delphinus_delphis_ACR	*ACR*	*Delphinus delphis*	Tetrapoda	XP_059881995.1	XM_060026012.1	132434361	NCBI RefSeq	410
251	Desmodus_rotundus_ACR	*ACR*	*Desmodus rotundus*	Tetrapoda	XP_053775790.1	XM_053919815.1	112321661	NCBI RefSeq	556
252	Diceros_bicornis_minor_ACR	*ACR*	*Diceros bicornis minor*	Tetrapoda	XP_058424786.1	XM_058568803.1	131421907	NCBI RefSeq	404
253	Dipodomys_merriami_ACR	*ACR*	*Dipodomys merriami*	Tetrapoda	XP_069855999.1	XM_069999898.1	138811929	NCBI RefSeq	402
254	Dipodomys_ordii_ACR	*ACR*	*Dipodomys ordii*	Tetrapoda	XP_012881187.1	XM_013025733.1	105992722	NCBI RefSeq	402
255	Echinops_telfairi_ACR	*ACR*	*Echinops telfairi*	Tetrapoda	XP_004700657.1	XM_004700600.1	101649792	NCBI RefSeq	426
256	Elephas_maximus_indicus_ACR	*ACR*	*Elephas maximus indicus*	Tetrapoda	XP_049740956.1	XM_049884999.1	126076508	NCBI RefSeq	411
257	Eleutherodactylus_coqui_ACR	*ACR*	*Eleutherodactylus coqui*	Tetrapoda	XP_066445422.1	XM_066589325.1	136610058	NCBI RefSeq	552
258	Empidonax_traillii_ACR	*ACR*	*Empidonax traillii*	Tetrapoda	XP_027746443.1	XM_027890642.1	114060836	NCBI RefSeq	438
259	Enhydra_lutris_kenyoni_ACR	*ACR*	*Enhydra lutris kenyoni*	Tetrapoda	XP_022375016.1	XM_022519308.1	111157685	NCBI RefSeq	408

260	Eptesicus_fuscus_ACR	*ACR*	*Eptesicus fuscus*	Tetrapoda	XP_027986867.2	XM_028131066.2	103298716	NCBI RefSeq	369
261	Equus_caballus_ACR	*ACR*	*Equus caballus*	Tetrapoda	XP_005606872.1	XM_005606815.4	100057028	NCBI RefSeq	399
262	Equus_przewalskii_ACR	*ACR*	*Equus przewalskii*	Tetrapoda	XP_070456480.1	XM_070600379.1	103543987	NCBI RefSeq	399
263	Equus_quagga_ACR	*ACR*	*Equus quagga*	Tetrapoda	XP_046502002.1	XM_046646046.1	124230229	NCBI RefSeq	402
264	Eretmochelys_imbricata_ACR	*ACR*	*Eretmochelys imbricata*	Tetrapoda	XP_077663671.1	XM_077807545.1	144259333	NCBI RefSeq	318
265	Erinaceus_europaeus_ACR	*ACR*	*Erinaceus europaeus*	Tetrapoda	XP_060044862.1	XM_060188879.1	103110241	NCBI RefSeq	481
266	Erithacus_rubecula_ACR	*ACR*	*Erithacus rubecula*	Tetrapoda	XP_079159502.1	XM_079303376.1	306800905	NCBI RefSeq	365
267	Eschrichtius_robustus_ACR	*ACR*	*Eschrichtius robustus*	Tetrapoda	XP_068417725.1	XM_068561624.1	137775609	NCBI RefSeq	424
268	Eubalaena_glacialis_ACR	*ACR*	*Eubalaena glacialis*	Tetrapoda	XP_061061751.1	XM_061205768.1	133101127	NCBI RefSeq	418
269	Eumetopias_jubatus_ACR	*ACR*	*Eumetopias jubatus*	Tetrapoda	XP_027971109.1	XM_028115308.1	114218308	NCBI RefSeq	412
270	Excalfactoria_chinensis_ACR	*ACR*	*Excalfactoria chinensis*	Tetrapoda	XP_072217637.1	XM_072361536.1	140265528	NCBI RefSeq	346
271	Felis_catus_ACR	*ACR*	*Felis catus*	Tetrapoda	XP_003989479.5	XM_003989430.6	101083949	NCBI RefSeq	423
272	Fukomys_damarensis_ACR	*ACR*	*Fukomys damarensis*	Tetrapoda	XP_033622045.1	XM_033766154.1	104869149	NCBI RefSeq	415

273	Galeopterus_variegatus_ACR	*ACR*	*Galeopterus variegatus*	Tetrapoda	XP_008571980.1	XM_008573758.1	103591321	NCBI RefSeq	353
274	Gavialis_gangeticus_ACR	*ACR*	*Gavialis gangeticus*	Tetrapoda	XP_019364892.1	XM_019509347.1	109292091	NCBI RefSeq	277
275	Gekko_japonicus_ACR	*ACR*	*Gekko japonicus*	Tetrapoda	XP_015276412.1	XM_015420926.1	107118562	NCBI RefSeq	351
276	Giraffa_tippelskirchi_ACR	*ACR*	*Giraffa tippelskirchi*	Tetrapoda	XP_081447266.1	XM_081591140.1	145545879	NCBI RefSeq	411
277	Globicephala_melas_ACR	*ACR*	*Globicephala melas*	Tetrapoda	XP_030711273.2	XM_030855413.2	115852559	NCBI RefSeq	404
278	Gorilla_gorilla_gorilla_ACR	*ACR*	*Gorilla gorilla gorilla*	Tetrapoda	XP_004063760.3	XM_004063712.5	101144239	NCBI RefSeq	419
279	Gracilinanus_agilis_ACR	*ACR*	*Gracilinanus agilis*	Tetrapoda	XP_044535439.1	XM_044679504.1	123250441	NCBI RefSeq	461
280	Grammomys_surdaster_ACR	*ACR*	*Grammomys surdaster*	Tetrapoda	XP_028609528.1	XM_028753695.1	114609348	NCBI RefSeq	436
281	Grampus_griseus_ACR	*ACR*	*Grampus griseus*	Tetrapoda	XP_082522335.1	XM_082666209.1	309829959	NCBI RefSeq	404
282	Haliaeetus_leucocephalus_ACR	*ACR*	*Haliaeetus leucocephalus*	Tetrapoda	XP_010568401.1	XM_010570099.1	104833452	NCBI RefSeq	343
283	Halichoerus_grypus_ACR	*ACR*	*Halichoerus grypus*	Tetrapoda	XP_035949443.1	XM_036093550.2	118536613	NCBI RefSeq	414
284	Herpailurus_yagouaroundi_ACR	*ACR*	*Herpailurus yagouaroundi*	Tetrapoda	XP_040346373.1	XM_040490439.1	121040095	NCBI RefSeq	423
285	Heterocephalus_glaber_ACR	*ACR*	*Heterocephalus glaber*	Tetrapoda	XP_004845950.1	XM_004845893.2	101712354	NCBI RefSeq	1743

286	Hippopotamus_amphibius_kiboko_ACR	*ACR*	*Hippopotamus amphibius kiboko*	Tetrapoda	XP_057599009.1	XM_057743 026.1	130857388	NCBI RefSe q	409
287	Hipposideros_armiger_ACR	*ACR*	*Hipposideros armiger*	Tetrapoda	XP_019524094.1	XM_019668 549.1	109396613	NCBI RefSe q	428
288	Homo_sapiens_ACR	*ACR*	*Homo sapiens*	Tetrapoda	NP_001088.2	NM_001097. 3	49	NCBI RefSe q	421
289	Hyaena_hyaena_ACR	*ACR*	*Hyaena hyaena*	Tetrapoda	XP_039090808.1	XM_039234 877.1	120233554	NCBI RefSe q	426
290	Hyla_sarda_ACR	*ACR*	*Hyla sarda*	Tetrapoda	XP_056428996.1	XM_056573 021.1	130368795	NCBI RefSe q	347
291	Hylobates_moloch_ACR	*ACR*	*Hylobates moloch*	Tetrapoda	XP_031995126.1	XM_032139 235.2	116458999	NCBI RefSe q	439
292	Hyperoodon_ampullatus_ACR	*ACR*	*Hyperoodon ampullatus*	Tetrapoda	XP_079571049.1	XM_079714 923.1	307060404	NCBI RefSe q	412
293	Ictidomys_tridecemlineatus_ACR	*ACR*	*Ictidomys tridecemlineat us*	Tetrapoda	XP_077908606.1	XM_078052 480.1	101961747	NCBI RefSe q	482
294	Jaculus_jaculus_ACR	*ACR*	*Jaculus jaculus*	Tetrapoda	XP_045009198.1	XM_045153 263.1	101607295	NCBI RefSe q	247
295	Kogia_breviceps_ACR	*ACR*	*Kogia breviceps*	Tetrapoda	XP_058936542.1	XM_059080 559.2	131766363	NCBI RefSe q	413
296	Lagenorhynchus_albirostris_ACR	*ACR*	*Lagenorhynch us albirostris*	Tetrapoda	XP_060021511.1	XM_060165 528.1	132529224	NCBI RefSe q	410
297	Lemur_catta_ACR	*ACR*	*Lemur catta*	Tetrapoda	XP_045410832.1	XM_045554 876.1	123640348	NCBI RefSe q	407
298	Leopardus_geoffroyi_ACR	*ACR*	*Leopardus geoffroyi*	Tetrapoda	XP_045320482.1	XM_045464 526.1	123590898	NCBI RefSe q	423

299	Leptonychotes_weddellii_ACR	*ACR*	*Leptonychotes weddellii*	Tetrapoda	XP_030886154.2	XM_031030294.2	102727815	NCBI RefSeq	414
300	Lepus_europaeus_ACR	*ACR*	*Lepus europaeus*	Tetrapoda	XP_062057872.1	XM_062201888.1	133767488	NCBI RefSeq	416
301	Leucopleurus_acutus_ACR	*ACR*	*Leucopleurus acutus*	Tetrapoda	XP_082706611.1	XM_082850485.1	309968989	NCBI RefSeq	410
302	Lipotes_vexillifer_ACR	*ACR*	*Lipotes vexillifer*	Tetrapoda	XP_007453931.1	XM_007453869.1	103072664	NCBI RefSeq	404
303	Lissotriton_helveticus_ACR	*ACR*	*Lissotriton helveticus*	Tetrapoda	XP_078505062.1	XM_078648936.1	144763371	NCBI RefSeq	370
304	Lontra_canadensis_ACR	*ACR*	*Lontra canadensis*	Tetrapoda	XP_032738884.1	XM_032882993.1	116882521	NCBI RefSeq	410
305	Loxodonta_africana_ACR	*ACR*	*Loxodonta africana*	Tetrapoda	XP_003423413.2	XM_003423365.3	100674496	NCBI RefSeq	411
306	Lutra_lutra_ACR	*ACR*	*Lutra lutra*	Tetrapoda	XP_053303837.1	XM_053447862.1	125105991	NCBI RefSeq	410
307	Lynx_canadensis_ACR	*ACR*	*Lynx canadensis*	Tetrapoda	XP_030176885.1	XM_030321025.1	115517921	NCBI RefSeq	425
308	Lynx_rufus_ACR	*ACR*	*Lynx rufus*	Tetrapoda	XP_046923169.1	XM_047067213.1	124502535	NCBI RefSeq	426
309	Macaca_fascicularis_ACR	*ACR*	*Macaca fascicularis*	Tetrapoda	XP_005566910.3	XM_005566853.4	102115325	NCBI RefSeq	419
310	Macaca_mulatta_ACR	*ACR*	*Macaca mulatta*	Tetrapoda	XP_001113191.4	XM_001113191.5	716579	NCBI RefSeq	419
311	Macaca_nemestrina_ACR	*ACR*	*Macaca nemestrina*	Tetrapoda	XP_011753039.2	XM_011754737.2	105489733	NCBI RefSeq	427

312	Macaca_thibetana_thibetana_ACR	*ACR*	*Macaca thibetana thibetana*	Tetrapoda	XP_050662840.1	XM_050806883.1	126964017	NCBI RefSeq	419
313	Mandrillus_leucophaeus_ACR	*ACR*	*Mandrillus leucophaeus*	Tetrapoda	XP_011840955.1	XM_011985565.1	105543589	NCBI RefSeq	417
314	Manis_javanica_ACR	*ACR*	*Manis javanica*	Tetrapoda	XP_017532986.2	XM_017677497.3	108407873	NCBI RefSeq	397
315	Manis_pentadactyla_ACR	*ACR*	*Manis pentadactyla*	Tetrapoda	XP_036762694.2	XM_036906799.2	118923223	NCBI RefSeq	411
316	Marmota_flaviventris_ACR	*ACR*	*Marmota flaviventris*	Tetrapoda	XP_027781183.2	XM_027925382.2	114084234	NCBI RefSeq	427
317	Marmota_marmota_marmota_ACR	*ACR*	*Marmota marmota marmota*	Tetrapoda	XP_048659105.1	XM_048803148.1	107160221	NCBI RefSeq	420
318	Marmota_monax_ACR	*ACR*	*Marmota monax*	Tetrapoda	XP_046299442.1	XM_046443486.1	124092131	NCBI RefSeq	431
319	Martes_martes_ACR	*ACR*	*Martes martes*	Tetrapoda	XP_080667947.1	XM_080811821.1	308347977	NCBI RefSeq	410
320	Mastomys_coucha_ACR	*ACR*	*Mastomys coucha*	Tetrapoda	XP_031208909.1	XM_031353049.1	116078085	NCBI RefSeq	436
321	Meleagris_gallopavo_ACR	*ACR*	*Meleagris gallopavo*	Tetrapoda	XP_010709824.2	XM_010711522.3	692129	NCBI RefSeq	402
322	Meles_meles_ACR	*ACR*	*Meles meles*	Tetrapoda	XP_045869356.1	XM_046013400.1	123947286	NCBI RefSeq	410
323	Meriones_unguiculatus_ACR	*ACR*	*Meriones unguiculatus*	Tetrapoda	XP_060246528.1	XM_060390545.1	110566471	NCBI RefSeq	441
324	Mesocricetus_auratus_ACR	*ACR*	*Mesocricetus auratus*	Tetrapoda	XP_005067166.1	XM_005067109.4	101839533	NCBI RefSeq	433

325	Mesoplodon_bidens_ACR	*ACR*	*Mesoplodon bidens*	Tetrapoda	XP_081105646.1	XM_081249520.1	308625402	NCBI RefSeq	392
326	Mesoplodon_densirostris_ACR	*ACR*	*Mesoplodon densirostris*	Tetrapoda	XP_059969649.1	XM_060113666.1	132499225	NCBI RefSeq	392
327	Mesoplodon_mirus_ACR	*ACR*	*Mesoplodon mirus*	Tetrapoda	XP_082491431.1	XM_082635305.1	309811932	NCBI RefSeq	412
328	Microcaecilia_unicolor_ACR	*ACR*	*Microcaecilia unicolor*	Tetrapoda	XP_030072594.1	XM_030216734.1	115479025	NCBI RefSeq	352
329	Microcebus_murinus_ACR	*ACR*	*Microcebus murinus*	Tetrapoda	XP_012644946.1	XM_012789492.3	105884990	NCBI RefSeq	404
330	Micromys_minutus_ACR	*ACR*	*Micromys minutus*	Tetrapoda	XP_080795050.1	XM_080938924.1	308437770	NCBI RefSeq	430
331	Microtus_ochrogaster_ACR	*ACR*	*Microtus ochrogaster*	Tetrapoda	XP_005354386.3	XM_005354329.3	101988062	NCBI RefSeq	439
332	Microtus_oregoni_ACR	*ACR*	*Microtus oregoni*	Tetrapoda	XP_041528431.1	XM_041672497.1	121461410	NCBI RefSeq	267
333	Microtus_pennsylvanicus_ACR	*ACR*	*Microtus pennsylvanicus*	Tetrapoda	XP_075816471.1	XM_075960356.1	142843262	NCBI RefSeq	440
334	Miniopterus_natalensis_ACR	*ACR*	*Miniopterus natalensis*	Tetrapoda	XP_016067129.1	XM_016211643.1	107536030	NCBI RefSeq	1743
335	Miniopterus_schreibersii_ACR	*ACR*	*Miniopterus schreibersii*	Tetrapoda	XP_080983973.1	XM_081127847.1	308552646	NCBI RefSeq	467
336	Mirounga_angustirostris_ACR	*ACR*	*Mirounga angustirostris*	Tetrapoda	XP_045722511.1	XM_045866555.2	123837557	NCBI RefSeq	414
337	Mirounga_leonina_ACR	*ACR*	*Mirounga leonina*	Tetrapoda	XP_034841831.1	XM_034985940.1	117997712	NCBI RefSeq	414

338	Molossus_molossus_ACR	*ACR*	*Molossus molossus*	Tetrapoda	XP_036134057.1	XM_036278164.1	118641119	NCBI RefSeq	397
339	Monodon_monoceros_ACR	*ACR*	*Monodon monoceros*	Tetrapoda	XP_029061641.1	XM_029205808.1	114885661	NCBI RefSeq	410
340	Moschus_berezovskii_ACR	*ACR*	*Moschus berezovskii*	Tetrapoda	XP_055252714.1	XM_055396739.2	129537237	NCBI RefSeq	414
341	Muntiacus_reevesi_ACR	*ACR*	*Muntiacus reevesi*	Tetrapoda	XP_065780734.1	XM_065924662.1	136160680	NCBI RefSeq	414
342	Mus_caroli_ACR	*ACR*	*Mus caroli*	Tetrapoda	XP_021039061.1	XM_021183402.3	110310440	NCBI RefSeq	436
343	Mus_musculus_ACR	*ACR*	*Mus musculus*	Tetrapoda	NP_038483.1	NM_013455.4	11434	NCBI RefSeq	436
344	Mus_pahari_ACR	*ACR*	*Mus pahari*	Tetrapoda	XP_021072710.1	XM_021217051.1	110334980	NCBI RefSeq	437
345	Muscardinus_avellanarius_ACR	*ACR*	*Muscardinus avellanarius*	Tetrapoda	XP_080561889.1	XM_080705763.1	308270068	NCBI RefSeq	267
346	Mustela_erminea_ACR	*ACR*	*Mustela erminea*	Tetrapoda	XP_032201626.1	XM_032345735.1	116592564	NCBI RefSeq	410
347	Mustela_lutreola_ACR	*ACR*	*Mustela lutreola*	Tetrapoda	XP_059043407.1	XM_059187424.1	131839677	NCBI RefSeq	410
348	Mustela_nigripes_ACR	*ACR*	*Mustela nigripes*	Tetrapoda	XP_059260548.1	XM_059404565.1	132020422	NCBI RefSeq	410
349	Mustela_nivalis_ACR	*ACR*	*Mustela nivalis*	Tetrapoda	XP_082606851.1	XM_082750725.1	309858029	NCBI RefSeq	410
350	Mustela_putorius_furo_ACR	*ACR*	*Mustela putorius furo*	Tetrapoda	XP_004775396.1	XM_004775339.3	101682369	NCBI RefSeq	410

351	Mycteria_americana_ACR	*ACR*	*Mycteria americana*	Tetrapoda	XP_075361954.1	XM_075505839.1	142411620	NCBI RefSeq	338
352	Myodes_glareolus_ACR	*ACR*	*Myodes glareolus*	Tetrapoda	XP_048300774.1	XM_048444817.3	125407331	NCBI RefSeq	436
353	Myotis_brandtii_ACR	*ACR*	*Myotis brandtii*	Tetrapoda	XP_005868307.1	XM_005868245.1	102256900	NCBI RefSeq	409
354	Myotis_daubentonii_ACR	*ACR*	*Myotis daubentonii*	Tetrapoda	XP_059536870.1	XM_059680887.1	132225977	NCBI RefSeq	440
355	Myotis_davidii_ACR	*ACR*	*Myotis davidii*	Tetrapoda	XP_006771183.1	XM_006771120.1	102774021	NCBI RefSeq	436
356	Myotis_lucifugus_ACR	*ACR*	*Myotis lucifugus*	Tetrapoda	XP_023602983.1	XM_023747215.1	102426257	NCBI RefSeq	347
357	Myotis_myotis_ACR	*ACR*	*Myotis myotis*	Tetrapoda	XP_036167156.1	XM_036311263.1	118657372	NCBI RefSeq	286
358	Myotis_nattereri_ACR	*ACR*	*Myotis nattereri*	Tetrapoda	XP_080940197.1	XM_081084071.1	308514043	NCBI RefSeq	426
359	Myotis_yumanensis_ACR	*ACR*	*Myotis yumanensis*	Tetrapoda	XP_070264966.1	XM_070408865.1	139006552	NCBI RefSeq	440
360	Nannospalax_galili_ACR	*ACR*	*Nannospalax galili*	Tetrapoda	XP_008833012.1	XM_008834790.3	103735496	NCBI RefSeq	431
361	Natator_depressus_ACR	*ACR*	*Natator depressus*	Tetrapoda	XP_074819104.1	XM_074963003.1	141993430	NCBI RefSeq	324
362	Natrix_helvetica_ACR	*ACR*	*Natrix helvetica*	Tetrapoda	XP_081814788.1	XM_081958662.1	309394944	NCBI RefSeq	447
363	Neofelis_nebulosa_ACR	*ACR*	*Neofelis nebulosa*	Tetrapoda	XP_058596842.1	XM_058740859.1	131518627	NCBI RefSeq	425

364	Neogale_vison_ACR	*ACR*	*Neogale vison*	Tetrapoda	XP_044083486.1	XM_044227551.1	122891694	NCBI RefSeq	410
365	Neomonachus_schauinslandi_ACR	*ACR*	*Neomonachus schauinslandi*	Tetrapoda	XP_021543525.1	XM_021687850.1	110578315	NCBI RefSeq	412
366	Neophocaena_asiaeorientalis_asiaeorientalis_ACR	*ACR*	*Neophocaena asiaeorientalis asiaeorientalis*	Tetrapoda	XP_024593155.1	XM_024737387.1	112394593	NCBI RefSeq	359
367	Nesoenas_mayeri_ACR	*ACR*	*Nesoenas mayeri*	Tetrapoda	XP_080214242.1	XM_080358116.1	308048806	NCBI RefSeq	415
368	Nomascus_leucogenys_ACR	*ACR*	*Nomascus leucogenys*	Tetrapoda	XP_003281526.1	XM_003281478.3	100586140	NCBI RefSeq	439
369	Numida_meleagris_ACR	*ACR*	*Numida meleagris*	Tetrapoda	XP_021239872.1	XM_021384197.1	110392172	NCBI RefSeq	356
370	Nyctereutes_procyonoides_ACR	*ACR*	*Nyctereutes procyonoides*	Tetrapoda	XP_055185339.1	XM_055329364.1	129512087	NCBI RefSeq	414
371	Nycticebus_coucang_ACR	*ACR*	*Nycticebus coucang*	Tetrapoda	XP_053441406.1	XM_053585431.1	128581992	NCBI RefSeq	409
372	Ochotona_curzoniae_ACR	*ACR*	*Ochotona curzoniae*	Tetrapoda	XP_040847795.1	XM_040991861.1	121164017	NCBI RefSeq	425
373	Ochotona_princeps_ACR	*ACR*	*Ochotona princeps*	Tetrapoda	XP_058529438.1	XM_058673455.1	101522965	NCBI RefSeq	467
374	Octodon_degus_ACR	*ACR*	*Octodon degus*	Tetrapoda	XP_004642496.1	XM_004642439.2	101581357	NCBI RefSeq	267
375	Odobenus_rosmarus_divergens_ACR	*ACR*	*Odobenus rosmarus divergens*	Tetrapoda	XP_012423484.1	XM_012568030.1	101379789	NCBI RefSeq	238
376	Odocoileus_virginianus_ACR	*ACR*	*Odocoileus virginianus*	Tetrapoda	XP_020729550.1	XM_020873891.2	110125051	NCBI RefSeq	414

377	Onychomys_torridus_ACR	*ACR*	*Onychomys torridus*	Tetrapoda	XP_036063895.1	XM_036208002.1	118596922	NCBI RefSeq	429
378	Orcinus_orca_ACR	*ACR*	*Orcinus orca*	Tetrapoda	XP_033260858.2	XM_033404967.2	101282422	NCBI RefSeq	410
379	Orycteropus_afer_afer_ACR	*ACR*	*Orycteropus afer afer*	Tetrapoda	XP_007952968.1	XM_007954777.1	103209014	NCBI RefSeq	425
380	Oryctolagus_cuniculus_ACR	*ACR*	*Oryctolagus cuniculus*	Tetrapoda	NP_001075805.1	NM_001082336.1	100009184	NCBI RefSeq	431
381	Oryx_dammah_ACR	*ACR*	*Oryx dammah*	Tetrapoda	XP_040089463.1	XM_040233529.1	120857786	NCBI RefSeq	413
382	Otolemur_garnettii_ACR	*ACR*	*Otolemur garnettii*	Tetrapoda	XP_003783457.1	XM_003783409.2	100963151	NCBI RefSeq	393
383	Ovis_aries_ACR	*ACR*	*Ovis aries*	Tetrapoda	XP_004007107.2	XM_004007058.6	443195	NCBI RefSeq	413
384	Ovis_canadensis_ACR	*ACR*	*Ovis canadensis*	Tetrapoda	XP_069438001.1	XM_069581900.1	138436213	NCBI RefSeq	413
385	Pan_paniscus_ACR	*ACR*	*Pan paniscus*	Tetrapoda	XP_003804640.5	XM_003804592.5	100979160	NCBI RefSeq	419
386	Pan_troglodytes_ACR	*ACR*	*Pan troglodytes*	Tetrapoda	XP_016795004.2	XM_016939515.4	458950	NCBI RefSeq	419
387	Panthera_leo_ACR	*ACR*	*Panthera leo*	Tetrapoda	XP_042804188.1	XM_042948254.1	122225308	NCBI RefSeq	423
388	Panthera_onca_ACR	*ACR*	*Panthera onca*	Tetrapoda	XP_060511979.1	XM_060655996.3	132693540	NCBI RefSeq	423
389	Panthera_pardus_ACR	*ACR*	*Panthera pardus*	Tetrapoda	XP_053751418.1	XM_053895443.1	109265792	NCBI RefSeq	423

390	Panthera_tigris_ACR	*ACR*	*Panthera tigris*	Tetrapoda	XP_007094083.2	XM_007094021.3	102954045	NCBI RefSeq	423
391	Panthera_uncia_ACR	*ACR*	*Panthera uncia*	Tetrapoda	XP_049482586.1	XM_049626629.1	125919756	NCBI RefSeq	423
392	Pelobates_fuscus_ACR	*ACR*	*Pelobates fuscus*	Tetrapoda	XP_063301792.1	XM_063445722.1	134601255	NCBI RefSeq	288
393	Peromyscus_californicus_insignis_ACR	*ACR*	*Peromyscus californicus insignis*	Tetrapoda	XP_052604631.1	XM_052748671.1	128116962	NCBI RefSeq	430
394	Peromyscus_eremicus_ACR	*ACR*	*Peromyscus eremicus*	Tetrapoda	XP_059103502.1	XM_059247519.1	131896751	NCBI RefSeq	431
395	Peromyscus_leucopus_ACR	*ACR*	*Peromyscus leucopus*	Tetrapoda	XP_028725055.1	XM_028869222.2	114693039	NCBI RefSeq	430
396	Peromyscus_maniculatus_bairdii_ACR	*ACR*	*Peromyscus maniculatus bairdii*	Tetrapoda	NP_001277291.1	NM_001290362.1	102925156	NCBI RefSeq	427
397	Phacochoerus_africanus_ACR	*ACR*	*Phacochoerus africanus*	Tetrapoda	XP_047644372.1	XM_047788416.1	125131633	NCBI RefSeq	412
398	Phascolarctos_cinereus_ACR	*ACR*	*Phascolarctos cinereus*	Tetrapoda	XP_078003979.1	XM_078147853.1	110205953	NCBI RefSeq	355
399	Phoca_vitulina_ACR	*ACR*	*Phoca vitulina*	Tetrapoda	XP_032272830.1	XM_032416939.1	116640129	NCBI RefSeq	414
400	Phocoena_phocoena_ACR	*ACR*	*Phocoena phocoena*	Tetrapoda	XP_065743371.1	XM_065887299.1	136130748	NCBI RefSeq	416
401	Phocoena_sinus_ACR	*ACR*	*Phocoena sinus*	Tetrapoda	XP_032501274.1	XM_032645383.1	116760485	NCBI RefSeq	416
402	Phodopus_roborovskii_ACR	*ACR*	*Phodopus roborovskii*	Tetrapoda	XP_051045621.1	XM_051189664.1	127225965	NCBI RefSeq	437

403	Phyllobates_terribilis_ACR	*ACR*	*Phyllobates terribilis*	Tetrapoda	XP_073510799.1	XM_073654698.1	141167264	NCBI RefSeq	293
404	Phyllostomus_discolor_ACR	*ACR*	*Phyllostomus discolor*	Tetrapoda	XP_028389092.1	XM_028533291.2	114513967	NCBI RefSeq	436
405	Phyllostomus_hastatus_ACR	*ACR*	*Phyllostomus hastatus*	Tetrapoda	XP_045690705.1	XM_045834749.1	123816028	NCBI RefSeq	439
406	Physeter_macrocephalus_ACR	*ACR*	*Physeter macrocephalus*	Tetrapoda	XP_028346647.1	XM_028490846.1	102982720	NCBI RefSeq	413
407	Piliocolobus_tephrosceles_ACR	*ACR*	*Piliocolobus tephrosceles*	Tetrapoda	XP_023078061.1	XM_023222293.2	111549166	NCBI RefSeq	419
408	Pipistrellus_kuhlii_ACR	*ACR*	*Pipistrellus kuhlii*	Tetrapoda	XP_036286051.1	XM_036430158.2	118714759	NCBI RefSeq	437
409	Plecotus_auritus_ACR	*ACR*	*Plecotus auritus*	Tetrapoda	XP_080516404.1	XM_080660278.1	308244351	NCBI RefSeq	440
410	Pogoniulus_pusillus_ACR	*ACR*	*Pogoniulus pusillus*	Tetrapoda	XP_063998387.1	XM_064142317.1	135174780	NCBI RefSeq	316
411	Pongo_abelii_ACR	*ACR*	*Pongo abelii*	Tetrapoda	XP_002831364.5	XM_002831318.5	100448292	NCBI RefSeq	409
412	Pongo_pygmaeus_ACR	*ACR*	*Pongo pygmaeus*	Tetrapoda	XP_054325621.2	XM_054469646.2	129023123	NCBI RefSeq	409
413	Prionailurus_bengalensis_ACR	*ACR*	*Prionailurus bengalensis*	Tetrapoda	XP_043419059.1	XM_043563124.1	122473012	NCBI RefSeq	423
414	Prionailurus_viverrinus_ACR	*ACR*	*Prionailurus viverrinus*	Tetrapoda	XP_047722816.1	XM_047866860.1	125170322	NCBI RefSeq	423
415	Propithecus_coquereli_ACR	*ACR*	*Propithecus coquereli*	Tetrapoda	XP_012504206.1	XM_012648752.1	105813926	NCBI RefSeq	407

416	Psammomys_obesus_ACR	*ACR*	*Psammomys obesus*	Tetrapoda	XP_055482954.1	XM_055626979.1	129691048	NCBI RefSeq	260
417	Pseudorca_crassidens_ACR	*ACR*	*Pseudorca crassidens*	Tetrapoda	XP_067554833.1	XM_067698732.1	137202963	NCBI RefSeq	410
418	Psittacula_echo_ACR	*ACR*	*Psittacula echo*	Tetrapoda	XP_080466794.1	XM_080610668.1	308217527	NCBI RefSeq	378
419	Pteronotus_mesoamericanus_ACR	*ACR*	*Pteronotus mesoamericanus*	Tetrapoda	XP_054426457.1	XM_054570482.1	129068320	NCBI RefSeq	437
420	Pteropus_alecto_ACR	*ACR*	*Pteropus alecto*	Tetrapoda	XP_024906786.1	XM_025051018.1	102886339	NCBI RefSeq	328
421	Pteropus_medius_ACR	*ACR*	*Pteropus medius*	Tetrapoda	XP_039718963.1	XM_039863029.1	120602806	NCBI RefSeq	416
422	Pteropus_vampyrus_ACR	*ACR*	*Pteropus vampyrus*	Tetrapoda	XP_023381210.1	XM_023525442.1	105308288	NCBI RefSeq	465
423	Puma_concolor_ACR	*ACR*	*Puma concolor*	Tetrapoda	XP_025782269.1	XM_025926484.1	112863376	NCBI RefSeq	239
424	Pygoscelis_adeliae_ACR	*ACR*	*Pygoscelis adeliae*	Tetrapoda	XP_009330840.1	XM_009332565.1	103924103	NCBI RefSeq	321
425	Rattus_norvegicus_ACR	*ACR*	*Rattus norvegicus*	Tetrapoda	NP_036622.2	NM_012490.2	24163	NCBI RefSeq	437
426	Rattus_rattus_ACR	*ACR*	*Rattus rattus*	Tetrapoda	XP_032746801.1	XM_032890910.1	116890176	NCBI RefSeq	403
427	Rhea_pennata_ACR	*ACR*	*Rhea pennata*	Tetrapoda	XP_062434832.1	XM_062578848.1	134142089	NCBI RefSeq	325
428	Rhinolophus_ferrumequinum_ACR	*ACR*	*Rhinolophus ferrumequinum*	Tetrapoda	XP_032972492.1	XM_033116601.1	117028267	NCBI RefSeq	458

429	Rhinolophus_hipposideros_ACR	*ACR*	*Rhinolophus hipposideros*	Tetrapoda	XP_080916624.1	XM_081060498.1	308502575	NCBI RefSeq	373
430	Rhinolophus_sinicus_ACR	*ACR*	*Rhinolophus sinicus*	Tetrapoda	XP_074181049.1	XM_074324948.1	141570034	NCBI RefSeq	448
431	Rhinopithecus_bieti_ACR	*ACR*	*Rhinopithecus bieti*	Tetrapoda	XP_017751911.1	XM_017896422.1	108545017	NCBI RefSeq	392
432	Rhinopithecus_roxellana_ACR	*ACR*	*Rhinopithecus roxellana*	Tetrapoda	XP_030770281.1	XM_030914421.1	104655563	NCBI RefSeq	419
433	Rousettus_aegyptiacus_ACR	*ACR*	*Rousettus aegyptiacus*	Tetrapoda	XP_036091793.1	XM_036235900.1	107500248	NCBI RefSeq	492
434	Saccopteryx_bilineata_ACR	*ACR*	*Saccopteryx bilineata*	Tetrapoda	XP_066108683.1	XM_066252586.1	136319245	NCBI RefSeq	358
435	Saccopteryx_leptura_ACR	*ACR*	*Saccopteryx leptura*	Tetrapoda	XP_066216378.1	XM_066360281.1	136388410	NCBI RefSeq	358
436	Sagmatias_obliquidens_ACR	*ACR*	*Sagmatias obliquidens*	Tetrapoda	XP_026964068.1	XM_027108267.1	113621343	NCBI RefSeq	365
437	Saimiri_boliviensis_ACR	*ACR*	*Saimiri boliviensis*	Tetrapoda	XP_003932847.1	XM_003932798.4	101047167	NCBI RefSeq	409
438	Sapajus_apella_ACR	*ACR*	*Sapajus apella*	Tetrapoda	XP_032122136.1	XM_032266245.1	116541779	NCBI RefSeq	417
439	Sarcophilus_harrisii_ACR	*ACR*	*Sarcophilus harrisii*	Tetrapoda	XP_031794500.1	XM_031938640.1	100932714	NCBI RefSeq	359
440	Sceloporus_undulatus_ACR	*ACR*	*Sceloporus undulatus*	Tetrapoda	XP_042326815.1	XM_042470881.1	121932299	NCBI RefSeq	442
441	Sciurus_carolinensis_ACR	*ACR*	*Sciurus carolinensis*	Tetrapoda	XP_047405789.1	XM_047549833.1	124982996	NCBI RefSeq	414

442	Sciurus_vulgaris_ACR	*ACR*	*Sciurus vulgaris*	Tetrapoda	XP_078997397.1	XM_079141 271.1	306661930	NCBI RefSe q	497
443	Sminthopsis_crassicaudata_ACR	*ACR*	*Sminthopsis crassicaudata*	Tetrapoda	XP_074128037.1	XM_074271 936.1	141545047	NCBI RefSe q	287
444	Sorex_araneus_ACR	*ACR*	*Sorex araneus*	Tetrapoda	XP_054999901.1	XM_055143 926.1	129406088	NCBI RefSe q	419
445	Sorex_fumeus_ACR	*ACR*	*Sorex fumeus*	Tetrapoda	XP_055963088.1	XM_056107 113.1	130016409	NCBI RefSe q	436
446	Stenella_coeruleoalba_ACR	*ACR*	*Stenella coeruleoalba*	Tetrapoda	XP_079792656.1	XM_079936 530.1	307193918	NCBI RefSe q	410
447	Strigops_habroptila_ACR	*ACR*	*Strigops habroptila*	Tetrapoda	XP_030327996.1	XM_030472 136.1	115601755	NCBI RefSe q	423
448	Strix_aluco_ACR	*ACR*	*Strix aluco*	Tetrapoda	XP_074682033.1	XM_074825 932.1	141924085	NCBI RefSe q	351
449	Sturnira_hondurensis_ACR	*ACR*	*Sturnira hondurensis*	Tetrapoda	XP_036905850.1	XM_037049 955.1	118989694	NCBI RefSe q	436
450	Suncus_etruscus_ACR	*ACR*	*Suncus etruscus*	Tetrapoda	XP_049627453.1	XM_049771 496.1	126006164	NCBI RefSe q	412
451	Suricata_suricatta_ACR	*ACR*	*Suricata suricatta*	Tetrapoda	XP_029810348.1	XM_029954 488.1	115304337	NCBI RefSe q	422
452	Sus_scrofa_ACR	*ACR*	*Sus scrofa*	Tetrapoda	NP_999198.2	NM_214033. 2	397098	NCBI RefSe q	415
453	Symphalangus_syndactylus_ACR	*ACR*	*Symphalangus syndactylus*	Tetrapoda	XP_055108609.1	XM_055252 634.2	129467815	NCBI RefSe q	443
454	Talpa_occidentalis_ACR	*ACR*	*Talpa occidentalis*	Tetrapoda	XP_037371086.1	XM_037515 189.2	119248216	NCBI RefSe q	416

455	Tamandua_tetradactyla_ACR	*ACR*	*Tamandua tetradactyla*	Tetrapoda	XP_077025429.1	XM_077169314.1	143691190	NCBI RefSeq	410
456	Tenrec_ecaudatus_ACR	*ACR*	*Tenrec ecaudatus*	Tetrapoda	XP_075408708.1	XM_075552593.1	142450604	NCBI RefSeq	426
457	Tetrao_urogallus_ACR	*ACR*	*Tetrao urogallus*	Tetrapoda	XP_079397346.1	XM_079541220.1	306950835	NCBI RefSeq	352
458	Theropithecus_gelada_ACR	*ACR*	*Theropithecus gelada*	Tetrapoda	XP_025255585.1	XM_025399800.1	112633283	NCBI RefSeq	419
459	Trachypithecus_francoisi_ACR	*ACR*	*Trachypithecus francoisi*	Tetrapoda	XP_033089262.1	XM_033233371.1	117096242	NCBI RefSeq	419
460	Trichechus_manatus_latirostris_ACR	*ACR*	*Trichechus manatus latirostris*	Tetrapoda	XP_023590410.1	XM_023734642.1	101350147	NCBI RefSeq	434
461	Tupaia_chinensis_ACR	*ACR*	*Tupaia chinensis*	Tetrapoda	XP_006157617.1	XM_006157555.3	102477933	NCBI RefSeq	399
462	Tursiops_truncatus_ACR	*ACR*	*Tursiops truncatus*	Tetrapoda	XP_033721502.1	XM_033865611.2	117314091	NCBI RefSeq	410
463	Urocitellus_parryii_ACR	*ACR*	*Urocitellus parryii*	Tetrapoda	XP_026237528.1	XM_026381743.1	113177199	NCBI RefSeq	267
464	Ursus_americanus_ACR	*ACR*	*Ursus americanus*	Tetrapoda	XP_045671478.1	XM_045815522.1	123803812	NCBI RefSeq	416
465	Ursus_arctos_ACR	*ACR*	*Ursus arctos*	Tetrapoda	XP_026335059.2	XM_026479274.3	113241583	NCBI RefSeq	413
466	Ursus_maritimus_ACR	*ACR*	*Ursus maritimus*	Tetrapoda	XP_008701190.2	XM_008702968.2	103674103	NCBI RefSeq	413
467	Varanus_komodoensis_ACR	*ACR*	*Varanus komodoensis*	Tetrapoda	XP_044286602.1	XM_044430667.1	123023716	NCBI RefSeq	502

468	Vicugna_pacos_ACR	*ACR*	*Vicugna pacos*	Tetrapoda	XP_006212615.2	XM_006212553.3	102543078	NCBI RefSeq	408
469	Vombatus_ursinus_ACR	*ACR*	*Vombatus ursinus*	Tetrapoda	XP_027710171.1	XM_027854370.1	114037470	NCBI RefSeq	311
470	Vulpes_lagopus_ACR	*ACR*	*Vulpes lagopus*	Tetrapoda	XP_041613427.1	XM_041757493.1	121492162	NCBI RefSeq	416
471	Vulpes_vulpes_ACR	*ACR*	*Vulpes vulpes*	Tetrapoda	XP_025875614.2	XM_026019829.2	112935879	NCBI RefSeq	416
472	Acinonyx_jubatus_TMPRSS12	*TMPRSS12*	*Acinonyx jubatus*	Tetrapoda	XP_053055600.1	XM_053199625.1	106969251	NCBI RefSeq	400
473	Acomys_russatus_TMPRSS12	*TMPRSS12*	*Acomys russatus*	Tetrapoda	XP_051015349.1	XM_051159392.1	127201038	NCBI RefSeq	340
474	Agelaius_phoeniceus_TMPRSS12	*TMPRSS12*	*Agelaius phoeniceus*	Tetrapoda	XP_054505232.2	XM_054649257.2	129130682	NCBI RefSeq	316
475	Agelaius_tricolor_TMPRSS12	*TMPRSS12*	*Agelaius tricolor*	Tetrapoda	XP_071274982.1	XM_071418881.1	139585872	NCBI RefSeq	316
476	Alligator_mississippiensis_TMPRSS12	*TMPRSS12*	*Alligator mississippiensis*	Tetrapoda	XP_059575028.1	XM_059719045.1	102576602	NCBI RefSeq	369
477	Alligator_sinensis_TMPRSS12	*TMPRSS12*	*Alligator sinensis*	Tetrapoda	XP_006030793.2	XM_006030731.3	102385536	NCBI RefSeq	211
478	Ammospiza_caudacuta_TMPRSS12	*TMPRSS12*	*Ammospiza caudacuta*	Tetrapoda	XP_058678218.1	XM_058822235.1	131569916	NCBI RefSeq	316
479	Ammospiza_nelsoni_TMPRSS12	*TMPRSS12*	*Ammospiza nelsoni*	Tetrapoda	XP_059347329.1	XM_059491346.1	132085867	NCBI RefSeq	316
480	Anas_acuta_TMPRSS12	*TMPRSS12*	*Anas acuta*	Tetrapoda	XP_068519727.1	XM_068663626.1	137846035	NCBI RefSeq	315

481	Anas_platyrhynchos_TMPRSS12	*TMPRSS12*	*Anas platyrhynchos*	Tetrapoda	XP_021130631.4	XM_021274956.4	101799751	NCBI RefSeq	315
482	Anomaloglossus_baeobatrachus_TMPRSS12	*TMPRSS12*	*Anomaloglossus baeobatrachus*	Tetrapoda	XP_075190622.1	XM_075334507.1	142290543	NCBI RefSeq	314
483	Anomalospiza_imberbis_TMPRSS12	*TMPRSS12*	*Anomalospiza imberbis*	Tetrapoda	XP_068068500.1	XM_068212399.1	137486859	NCBI RefSeq	318
484	Anser_brachyrhynchus_TMPRSS12	*TMPRSS12*	*Anser brachyrhynchus*	Tetrapoda	XP_080426908.1	XM_080570782.1	308184069	NCBI RefSeq	315
485	Anser_cygnoides_TMPRSS12	*TMPRSS12*	*Anser cygnoides*	Tetrapoda	XP_013054810.3	XM_013199356.3	106047728	NCBI RefSeq	315
486	Aotus_nancymaae_TMPRSS12	*TMPRSS12*	*Aotus nancymaae*	Tetrapoda	XP_064233158.1	XM_064377088.1	110567205	NCBI RefSeq	348
487	Aphelocoma_coerulescens_TMPRSS12	*TMPRSS12*	*Aphelocoma coerulescens*	Tetrapoda	XP_068853724.1	XM_068997623.1	138099976	NCBI RefSeq	318
488	Apodemus_sylvaticus_TMPRSS12	*TMPRSS12*	*Apodemus sylvaticus*	Tetrapoda	XP_052017067.1	XM_052161107.1	127667783	NCBI RefSeq	338
489	Aptenodytes_forsteri_TMPRSS12	*TMPRSS12*	*Aptenodytes forsteri*	Tetrapoda	XP_009272689.1	XM_009274414.1	103894933	NCBI RefSeq	306
490	Aptenodytes_patagonicus_TMPRSS12	*TMPRSS12*	*Aptenodytes patagonicus*	Tetrapoda	XP_076216542.1	XM_076360427.1	143171448	NCBI RefSeq	315
491	Apteryx_mantelli_TMPRSS12	*TMPRSS12*	*Apteryx mantelli*	Tetrapoda	XP_067170354.1	XM_067314253.1	106499418	NCBI RefSeq	313
492	Apteryx_rowi_TMPRSS12	*TMPRSS12*	*Apteryx rowi*	Tetrapoda	XP_025940770.1	XM_026084985.1	112975220	NCBI RefSeq	305
493	Apus_apus_TMPRSS12	*TMPRSS12*	*Apus apus*	Tetrapoda	XP_051497347.1	XM_051641387.1	127395012	NCBI RefSeq	329

494	Aquila_chrysaetos_chrysaetos_TMPRSS12	*TMPRSS12*	*Aquila chrysaetos chrysaetos*	Tetrapoda	XP_029893096.1	XM_030037236.2	115350997	NCBI RefSeq	326
495	Artibeus_jamaicensis_TMPRSS12	*TMPRSS12*	*Artibeus jamaicensis*	Tetrapoda	XP_053517410.1	XM_053661435.1	119042945	NCBI RefSeq	392
496	Arvicanthis_niloticus_TMPRSS12	*TMPRSS12*	*Arvicanthis niloticus*	Tetrapoda	XP_034371859.1	XM_034515968.2	117718430	NCBI RefSeq	334
497	Arvicola_amphibius_TMPRSS12	*TMPRSS12*	*Arvicola amphibius*	Tetrapoda	XP_038197090.1	XM_038341162.1	119822188	NCBI RefSeq	341
498	Ascaphus_truei_TMPRSS12	*TMPRSS12*	*Ascaphus truei*	Tetrapoda	XP_075447769.1	XM_075591654.1	142489968	NCBI RefSeq	322
499	Astur_gentilis_TMPRSS12	*TMPRSS12*	*Astur gentilis*	Tetrapoda	XP_049674491.1	XM_049818534.1	126046307	NCBI RefSeq	326
500	Athene_cunicularia_TMPRSS12	*TMPRSS12*	*Athene cunicularia*	Tetrapoda	XP_026723466.1	XM_026867665.1	113491041	NCBI RefSeq	220
501	Athene_noctua_TMPRSS12	*TMPRSS12*	*Athene noctua*	Tetrapoda	XP_074785523.1	XM_074929422.1	141971828	NCBI RefSeq	304
502	Aythya_fuligula_TMPRSS12	*TMPRSS12*	*Aythya fuligula*	Tetrapoda	XP_032060588.1	XM_032204697.1	116499846	NCBI RefSeq	312
503	Aythya_marila_TMPRSS12	*TMPRSS12*	*Aythya marila*	Tetrapoda	XP_082637583.1	XM_082781457.1	309888849	NCBI RefSeq	312
504	Balaenoptera_acutorostrata_TMPRSS12	*TMPRSS12*	*Balaenoptera acutorostrata*	Tetrapoda	XP_007179856.1	XM_007179794.2	103006389	NCBI RefSeq	356
505	Balaenoptera_borealis_TMPRSS12	*TMPRSS12*	*Balaenoptera borealis*	Tetrapoda	XP_082208695.1	XM_082352569.1	309610108	NCBI RefSeq	354
506	Balaenoptera_physalus_TMPRSS12	*TMPRSS12*	*Balaenoptera physalus*	Tetrapoda	XP_082277642.1	XM_082421516.1	309641472	NCBI RefSeq	354

507	Balaenoptera_ricei_TMPRSS12	*TMPRSS12*	*Balaenoptera ricei*	Tetrapoda	XP_059790571.1	XM_059934588.1	132372523	NCBI RefSeq	365
508	Balearica_regulorum_gibbericeps_TMPRSS12	*TMPRSS12*	*Balearica regulorum gibbericeps*	Tetrapoda	XP_075593933.1	XM_075737818.1	104633696	NCBI RefSeq	321
509	Bison_bison_bison_TMPRSS12	*TMPRSS12*	*Bison bison bison*	Tetrapoda	XP_010856376.1	XM_010858074.1	105001698	NCBI RefSeq	343
510	Bos_indicus_TMPRSS12	*TMPRSS12*	*Bos indicus*	Tetrapoda	XP_070644848.1	XM_070788747.1	109558354	NCBI RefSeq	469
511	Bos_indicus_x_Bos_taurus_TMPRSS12	*TMPRSS12*	*Bos indicus x Bos taurus*	Tetrapoda	XP_027399295.1	XM_027543494.1	113893575	NCBI RefSeq	356
512	Bos_javanicus_TMPRSS12	*TMPRSS12*	*Bos javanicus*	Tetrapoda	XP_061275012.1	XM_061419028.1	133248657	NCBI RefSeq	467
513	Bos_mutus_TMPRSS12	*TMPRSS12*	*Bos mutus*	Tetrapoda	XP_005892095.1	XM_005892033.2	102272262	NCBI RefSeq	356
514	Bos_taurus_TMPRSS12	*TMPRSS12*	*Bos taurus*	Tetrapoda	XP_059743229.1	XM_059887246.1	100137906	NCBI RefSeq	467
515	Branta_canadensis_TMPRSS12	*TMPRSS12*	*Branta canadensis*	Tetrapoda	XP_081894694.1	XM_082038568.1	309431807	NCBI RefSeq	315
516	Bubalus_bubalis_TMPRSS12	*TMPRSS12*	*Bubalus bubalis*	Tetrapoda	XP_044797922.1	XM_044941987.1	112584409	NCBI RefSeq	368
517	Bubalus_kerabau_TMPRSS12	*TMPRSS12*	*Bubalus kerabau*	Tetrapoda	XP_055436098.1	XM_055580123.1	129651347	NCBI RefSeq	368
518	Bucephala_clangula_TMPRSS12	*TMPRSS12*	*Bucephala clangula*	Tetrapoda	XP_080822209.1	XM_080966083.1	308453844	NCBI RefSeq	296
519	Budorcas_taxicolor_TMPRSS12	*TMPRSS12*	*Budorcas taxicolor*	Tetrapoda	XP_052496506.1	XM_052640546.1	128048100	NCBI RefSeq	349

520	Buteo_buteo_TMPRSS12	*TMPRSS12*	*Buteo buteo*	Tetrapoda	XP_074905192.1	XM_075049091.1	142040820	NCBI RefSeq	326
521	Calidris_pugnax_TMPRSS12	*TMPRSS12*	*Calidris pugnax*	Tetrapoda	XP_014802933.1	XM_014947447.1	106891133	NCBI RefSeq	320
522	Callithrix_jacchus_TMPRSS12	*TMPRSS12*	*Callithrix jacchus*	Tetrapoda	XP_002752463.4	XM_002752417.7	100387148	NCBI RefSeq	348
523	Callorhinus_ursinus_TMPRSS12	*TMPRSS12*	*Callorhinus ursinus*	Tetrapoda	XP_025739212.2	XM_025883427.2	112832670	NCBI RefSeq	351
524	Caloenas_nicobarica_TMPRSS12	*TMPRSS12*	*Caloenas nicobarica*	Tetrapoda	XP_065510595.1	XM_065654523.1	136000601	NCBI RefSeq	343
525	Calonectris_borealis_TMPRSS12	*TMPRSS12*	*Calonectris borealis*	Tetrapoda	XP_074989778.1	XM_075133677.1	142073659	NCBI RefSeq	315
526	Calypte_anna_TMPRSS12	*TMPRSS12*	*Calypte anna*	Tetrapoda	XP_030323946.1	XM_030468086.1	103527121	NCBI RefSeq	317
527	Camarhynchus_parvulus_TMPRSS12	*TMPRSS12*	*Camarhynchus parvulus*	Tetrapoda	XP_030822845.1	XM_030966985.1	115914459	NCBI RefSeq	362
528	Camelus_bactrianus_TMPRSS12	*TMPRSS12*	*Camelus bactrianus*	Tetrapoda	XP_074230977.1	XM_074374876.1	105076357	NCBI RefSeq	258
529	Camelus_dromedarius_TMPRSS12	*TMPRSS12*	*Camelus dromedarius*	Tetrapoda	XP_064347309.1	XM_064491239.1	105097205	NCBI RefSeq	353
530	Camelus_ferus_TMPRSS12	*TMPRSS12*	*Camelus ferus*	Tetrapoda	XP_032349762.1	XM_032493871.1	102518241	NCBI RefSeq	420
531	Canis_aureus_TMPRSS12	*TMPRSS12*	*Canis aureus*	Tetrapoda	XP_077726195.1	XM_077870069.1	144296823	NCBI RefSeq	351
532	Canis_lupus_baileyi_TMPRSS12	*TMPRSS12*	*Canis lupus baileyi*	Tetrapoda	XP_072654038.1	XM_072797937.1	140617098	NCBI RefSeq	351

533	Canis_lupus_dingo_TMPRSS12	*TMPRSS12*	*Canis lupus dingo*	Tetrapoda	XP_025333410.1	XM_025477625.3	112678325	NCBI RefSeq	351
534	Canis_lupus_familiaris_TMPRSS12	*TMPRSS12*	*Canis lupus familiaris*	Tetrapoda	XP_038294123.1	XM_038438195.1	608553	NCBI RefSeq	352
535	Capra_hircus_TMPRSS12	*TMPRSS12*	*Capra hircus*	Tetrapoda	XP_017904512.1	XM_018049023.1	102188196	NCBI RefSeq	349
536	Capricornis_sumatraensis_TMPRSS12	*TMPRSS12*	*Capricornis sumatraensis*	Tetrapoda	XP_068827525.1	XM_068971424.1	138078601	NCBI RefSeq	349
537	Carettochelys_insculpta_TMPRSS12	*TMPRSS12*	*Carettochelys insculpta*	Tetrapoda	XP_074836416.1	XM_074980315.1	142003395	NCBI RefSeq	334
538	Cariama_cristata_TMPRSS12	*TMPRSS12*	*Cariama cristata*	Tetrapoda	XP_009693365.1	XM_009695063.1	104157013	NCBI RefSeq	168
539	Carlito_syrichta_TMPRSS12	*TMPRSS12*	*Carlito syrichta*	Tetrapoda	XP_008072284.1	XM_008074093.1	103276686	NCBI RefSeq	297
540	Castor_canadensis_TMPRSS12	*TMPRSS12*	*Castor canadensis*	Tetrapoda	XP_020035499.2	XM_020179910.2	109696742	NCBI RefSeq	379
541	Catharus_ustulatus_TMPRSS12	*TMPRSS12*	*Catharus ustulatus*	Tetrapoda	XP_032938985.1	XM_033083094.1	117008953	NCBI RefSeq	318
542	Cavia_porcellus_TMPRSS12	*TMPRSS12*	*Cavia porcellus*	Tetrapoda	XP_013014493.2	XM_013159039.3	100735680	NCBI RefSeq	367
543	Cebus_imitator_TMPRSS12	*TMPRSS12*	*Cebus imitator*	Tetrapoda	XP_017401336.1	XM_017545847.2	108316096	NCBI RefSeq	348
544	Centrocercus_urophasianus_TMPRSS12	*TMPRSS12*	*Centrocercus urophasianus*	Tetrapoda	XP_042694233.1	XM_042838299.1	122170991	NCBI RefSeq	318
545	Ceratotherium_simum_simum_TMPRSS12	*TMPRSS12*	*Ceratotherium simum simum*	Tetrapoda	XP_014642257.1	XM_014786771.1	101389703	NCBI RefSeq	354

546	Cercocebus_atys_TMPRSS12	*TMPRSS12*	*Cercocebus atys*	Tetrapoda	XP_011903680.1	XM_012048290.1	105580350	NCBI RefSeq	348
547	Cervus_canadensis_TMPRSS12	*TMPRSS12*	*Cervus canadensis*	Tetrapoda	XP_043302402.1	XM_043446467.1	122427168	NCBI RefSeq	356
548	Cervus_elaphus_TMPRSS12	*TMPRSS12*	*Cervus elaphus*	Tetrapoda	XP_043747187.1	XM_043891252.1	122686136	NCBI RefSeq	356
549	Chaetura_pelagica_TMPRSS12	*TMPRSS12*	*Chaetura pelagica*	Tetrapoda	XP_009992390.1	XM_009994088.1	104385610	NCBI RefSeq	304
550	Chamaea_fasciata_TMPRSS12	*TMPRSS12*	*Chamaea fasciata*	Tetrapoda	XP_066063389.1	XM_066207292.1	136303712	NCBI RefSeq	354
551	Charadrius_vociferus_TMPRSS12	*TMPRSS12*	*Charadrius vociferus*	Tetrapoda	XP_009881505.1	XM_009883203.1	104284831	NCBI RefSeq	292
552	Chelonia_mydas_TMPRSS12	*TMPRSS12*	*Chelonia mydas*	Tetrapoda	XP_037739686.2	XM_037883758.2	102939954	NCBI RefSeq	377
553	Chelonoidis_abingdonii_TMPRSS12	*TMPRSS12*	*Chelonoidis abingdonii*	Tetrapoda	XP_032642384.1	XM_032786493.2	116828347	NCBI RefSeq	329
554	Chinchilla_lanigera_TMPRSS12	*TMPRSS12*	*Chinchilla lanigera*	Tetrapoda	XP_013363897.1	XM_013508443.1	102018227	NCBI RefSeq	367
555	Chionomys_nivalis_TMPRSS12	*TMPRSS12*	*Chionomys nivalis*	Tetrapoda	XP_057648666.1	XM_057792683.1	130889130	NCBI RefSeq	341
556	Chiroxiphia_lanceolata_TMPRSS12	*TMPRSS12*	*Chiroxiphia lanceolata*	Tetrapoda	XP_032569310.1	XM_032713419.1	116799950	NCBI RefSeq	319
557	Chlorocebus_sabaeus_TMPRSS12	*TMPRSS12*	*Chlorocebus sabaeus*	Tetrapoda	XP_008001427.2	XM_008003236.3	103238309	NCBI RefSeq	348
558	Choloepus_didactylus_TMPRSS12	*TMPRSS12*	*Choloepus didactylus*	Tetrapoda	XP_037703366.1	XM_037847438.1	119542784	NCBI RefSeq	326

559	Chroicocephalus_ridibundus_TMPRSS12	*TMPRSS12*	*Chroicocephalus ridibundus*	Tetrapoda	XP_063215368.1	XM_063359298.1	134526947	NCBI RefSeq	308
560	Chrysemys_picta_bellii_TMPRSS12	*TMPRSS12*	*Chrysemys picta bellii*	Tetrapoda	XP_042713333.2	XM_042857399.2	101942973	NCBI RefSeq	326
561	Chrysochloris_asiatica_TMPRSS12	*TMPRSS12*	*Chrysochloris asiatica*	Tetrapoda	XP_006870199.1	XM_006870137.1	102831774	NCBI RefSeq	395
562	Ciconia_boyciana_TMPRSS12	*TMPRSS12*	*Ciconia boyciana*	Tetrapoda	XP_072703532.1	XM_072847431.1	140644515	NCBI RefSeq	316
563	Cinclus_cinclus_TMPRSS12	*TMPRSS12*	*Cinclus cinclus*	Tetrapoda	XP_062367011.1	XM_062511027.1	134054948	NCBI RefSeq	318
564	Cnephaeus_nilssonii_TMPRSS12	*TMPRSS12*	*Cnephaeus nilssonii*	Tetrapoda	XP_079630192.1	XM_079774066.1	307101266	NCBI RefSeq	293
565	Colius_striatus_TMPRSS12	*TMPRSS12*	*Colius striatus*	Tetrapoda	XP_061871311.1	XM_062015327.1	104553668	NCBI RefSeq	373
566	Colobus_angolensis_palliatus_TMPRSS12	*TMPRSS12*	*Colobus angolensis palliatus*	Tetrapoda	XP_011791674.1	XM_011936284.1	105507681	NCBI RefSeq	217
567	Coloeus_monedula_TMPRSS12	*TMPRSS12*	*Coloeus monedula*	Tetrapoda	XP_082155188.1	XM_082299062.1	309583402	NCBI RefSeq	320
568	Columba_livia_TMPRSS12	*TMPRSS12*	*Columba livia*	Tetrapoda	XP_064899746.1	XM_065043674.1	102094369	NCBI RefSeq	369
569	Condylura_cristata_TMPRSS12	*TMPRSS12*	*Condylura cristata*	Tetrapoda	XP_004692573.1	XM_004692516.1	101625431	NCBI RefSeq	350
570	Corapipo_altera_TMPRSS12	*TMPRSS12*	*Corapipo altera*	Tetrapoda	XP_027524317.1	XM_027668516.1	113959461	NCBI RefSeq	319
571	Corvus_brachyrhynchos_TMPRSS12	*TMPRSS12*	*Corvus brachyrhynchos*	Tetrapoda	XP_017586229.1	XM_017730740.1	103613221	NCBI RefSeq	302

572	Corvus_hawaiiensis_TMPRSS12	*TMPRSS12*	*Corvus hawaiiensis*	Tetrapoda	XP_048184266.1	XM_048328309.1	125337995	NCBI RefSeq	356
573	Corvus_kubaryi_TMPRSS12	*TMPRSS12*	*Corvus kubaryi*	Tetrapoda	XP_041879604.1	XM_042023670.1	121662478	NCBI RefSeq	318
574	Corvus_moneduloides_TMPRSS12	*TMPRSS12*	*Corvus moneduloides*	Tetrapoda	XP_031949708.1	XM_032093817.1	116436592	NCBI RefSeq	318
575	Coturnix_japonica_TMPRSS12	*TMPRSS12*	*Coturnix japonica*	Tetrapoda	XP_015742319.1	XM_015886833.2	107325709	NCBI RefSeq	318
576	Cricetulus_griseus_TMPRSS12	*TMPRSS12*	*Cricetulus griseus*	Tetrapoda	XP_003513589.1	XM_003513541.3	100763916	NCBI RefSeq	342
577	Crocodylus_porosus_TMPRSS12	*TMPRSS12*	*Crocodylus porosus*	Tetrapoda	XP_019411939.1	XM_019556394.1	109324399	NCBI RefSeq	402
578	Crocuta_crocuta_TMPRSS12	*TMPRSS12*	*Crocuta crocuta*	Tetrapoda	XP_077609800.1	XM_077753674.1	144222969	NCBI RefSeq	414
579	Cuculus_canorus_TMPRSS12	*TMPRSS12*	*Cuculus canorus*	Tetrapoda	XP_053907380.1	XM_054051405.1	104055655	NCBI RefSeq	445
580	Cyanistes_caeruleus_TMPRSS12	*TMPRSS12*	*Cyanistes caeruleus*	Tetrapoda	XP_023799006.1	XM_023943238.1	111940516	NCBI RefSeq	304
581	Cygnus_atratus_TMPRSS12	*TMPRSS12*	*Cygnus atratus*	Tetrapoda	XP_035426255.1	XM_035570362.1	118260240	NCBI RefSeq	312
582	Cygnus_columbianus_TMPRSS12	*TMPRSS12*	*Cygnus columbianus*	Tetrapoda	XP_082645094.1	XM_082788968.1	309893321	NCBI RefSeq	315
583	Cygnus_olor_TMPRSS12	*TMPRSS12*	*Cygnus olor*	Tetrapoda	XP_040395724.1	XM_040539790.1	121061228	NCBI RefSeq	315
584	Cynocephalus_volans_TMPRSS12	*TMPRSS12*	*Cynocephalus volans*	Tetrapoda	XP_062971636.1	XM_063115566.1	134391572	NCBI RefSeq	354

585	Dama_dama_TMPRSS12	*TMPRSS12*	*Dama dama*	Tetrapoda	XP_060979876.1	XM_061123893.1	133042872	NCBI RefSeq	356
586	Dasypus_novemcinctus_TMPRSS12	*TMPRSS12*	*Dasypus novemcinctus*	Tetrapoda	XP_004453750.1	XM_004453693.2	101416790	NCBI RefSeq	310
587	Delphinapterus_leucas_TMPRSS12	*TMPRSS12*	*Delphinapterus leucas*	Tetrapoda	XP_022427202.1	XM_022571494.1	111173722	NCBI RefSeq	356
588	Delphinus_delphis_TMPRSS12	*TMPRSS12*	*Delphinus delphis*	Tetrapoda	XP_059882199.1	XM_060026216.1	132434532	NCBI RefSeq	366
589	Dendrobates_tinctorius_TMPRSS12	*TMPRSS12*	*Dendrobates tinctorius*	Tetrapoda	XP_073433027.1	XM_073576926.1	141091370	NCBI RefSeq	232
590	Dendropsophus_ebraccatus_TMPRSS12	*TMPRSS12*	*Dendropsophus ebraccatus*	Tetrapoda	XP_069826380.1	XM_069970279.1	138792633	NCBI RefSeq	316
591	Dermochelys_coriacea_TMPRSS12	*TMPRSS12*	*Dermochelys coriacea*	Tetrapoda	XP_043362352.1	XM_043506417.1	119845644	NCBI RefSeq	347
592	Desmodus_rotundus_TMPRSS12	*TMPRSS12*	*Desmodus rotundus*	Tetrapoda	XP_053775740.1	XM_053919765.1	112318598	NCBI RefSeq	445
593	Diceros_bicornis_minor_TMPRSS12	*TMPRSS12*	*Diceros bicornis minor*	Tetrapoda	XP_058415229.1	XM_058559246.1	131416578	NCBI RefSeq	354
594	Dipodomys_merriami_TMPRSS12	*TMPRSS12*	*Dipodomys merriami*	Tetrapoda	XP_069897121.1	XM_070041020.1	138840719	NCBI RefSeq	337
595	Dipodomys_ordii_TMPRSS12	*TMPRSS12*	*Dipodomys ordii*	Tetrapoda	XP_012874142.1	XM_013018688.1	105987423	NCBI RefSeq	338
596	Dipodomys_spectabilis_TMPRSS12	*TMPRSS12*	*Dipodomys spectabilis*	Tetrapoda	XP_042524736.1	XM_042668802.1	122098399	NCBI RefSeq	379
597	Dromaius_novaehollandiae_TMPRSS12	*TMPRSS12*	*Dromaius novaehollandiae*	Tetrapoda	XP_025970111.1	XM_026114326.2	112991670	NCBI RefSeq	317

598	Dryobates_pubescens_TMPRSS12	*TMPRSS12*	*Dryobates pubescens*	Tetrapoda	XP_054033311.1	XM_054177336.1	104305504	NCBI RefSeq	459
599	Echinops_telfairi_TMPRSS12	*TMPRSS12*	*Echinops telfairi*	Tetrapoda	XP_012862370.1	XM_013006916.2	101643530	NCBI RefSeq	345
600	Egretta_garzetta_TMPRSS12	*TMPRSS12*	*Egretta garzetta*	Tetrapoda	XP_009644411.1	XM_009646116.1	104132782	NCBI RefSeq	308
601	Elephantulus_edwardii_TMPRSS12	*TMPRSS12*	*Elephantulus edwardii*	Tetrapoda	XP_006900325.1	XM_006900263.1	102858857	NCBI RefSeq	288
602	Elephas_maximus_indicus_TMPRSS12	*TMPRSS12*	*Elephas maximus indicus*	Tetrapoda	XP_049740838.1	XM_049884881.1	126076328	NCBI RefSeq	365
603	Eleutherodactylus_coqui_TMPRSS12	*TMPRSS12*	*Eleutherodactylus coqui*	Tetrapoda	XP_066445096.1	XM_066588999.1	136606209	NCBI RefSeq	270
604	Empidonax_traillii_TMPRSS12	*TMPRSS12*	*Empidonax traillii*	Tetrapoda	XP_027762902.1	XM_027907101.1	114069952	NCBI RefSeq	319
605	Emydura_macquarii_macquarii_TMPRSS12	*TMPRSS12*	*Emydura macquarii macquarii*	Tetrapoda	XP_067385928.1	XM_067529827.1	137141028	NCBI RefSeq	289
606	Emys_orbicularis_TMPRSS12	*TMPRSS12*	*Emys orbicularis*	Tetrapoda	XP_065275134.1	XM_065419062.1	135891515	NCBI RefSeq	330
607	Engystomops_pustulosus_TMPRSS12	*TMPRSS12*	*Engystomops pustulosus*	Tetrapoda	XP_071989124.1	XM_072133023.1	140116507	NCBI RefSeq	327
608	Enhydra_lutris_kenyoni_TMPRSS12	*TMPRSS12*	*Enhydra lutris kenyoni*	Tetrapoda	XP_022375502.1	XM_022519794.1	111158018	NCBI RefSeq	363
609	Eptesicus_fuscus_TMPRSS12	*TMPRSS12*	*Eptesicus fuscus*	Tetrapoda	XP_054575055.1	XM_054719080.1	103285528	NCBI RefSeq	345
610	Equus_asinus_TMPRSS12	*TMPRSS12*	*Equus asinus*	Tetrapoda	XP_070350296.1	XM_070494195.1	106845030	NCBI RefSeq	356

611	Equus_caballus_TMPRSS12	*TMPRSS12*	*Equus caballus*	Tetrapoda	XP_023499321.1	XM_023643553.2	100060327	NCBI RefSeq	355
612	Equus_przewalskii_TMPRSS12	*TMPRSS12*	*Equus przewalskii*	Tetrapoda	XP_008510658.1	XM_008512436.2	103545926	NCBI RefSeq	355
613	Equus_quagga_TMPRSS12	*TMPRSS12*	*Equus quagga*	Tetrapoda	XP_046495786.1	XM_046639830.1	124226491	NCBI RefSeq	356
614	Erinaceus_europaeus_TMPRSS12	*TMPRSS12*	*Erinaceus europaeus*	Tetrapoda	XP_007528593.2	XM_007528531.2	103118316	NCBI RefSeq	344
615	Erythrolamprus_reginae_TMPRSS12	*TMPRSS12*	*Erythrolamprus reginae*	Tetrapoda	XP_070596614.1	XM_070740513.1	139161416	NCBI RefSeq	313
616	Eschrichtius_robustus_TMPRSS12	*TMPRSS12*	*Eschrichtius robustus*	Tetrapoda	XP_068418019.1	XM_068561918.1	137775743	NCBI RefSeq	365
617	Eubalaena_glacialis_TMPRSS12	*TMPRSS12*	*Eubalaena glacialis*	Tetrapoda	XP_061062116.1	XM_061206133.1	133101381	NCBI RefSeq	365
618	Eublepharis_macularius_TMPRSS12	*TMPRSS12*	*Eublepharis macularius*	Tetrapoda	XP_054831686.1	XM_054975711.1	129327225	NCBI RefSeq	299
619	Eulemur_rufifrons_TMPRSS12	*TMPRSS12*	*Eulemur rufifrons*	Tetrapoda	XP_069347557.1	XM_069491456.1	138397435	NCBI RefSeq	351
620	Euleptes_europaea_TMPRSS12	*TMPRSS12*	*Euleptes europaea*	Tetrapoda	XP_056704941.1	XM_056848963.1	130476951	NCBI RefSeq	319
621	Eumetopias_jubatus_TMPRSS12	*TMPRSS12*	*Eumetopias jubatus*	Tetrapoda	XP_027981238.1	XM_028125437.1	114225715	NCBI RefSeq	352
622	Eurypyga_helias_TMPRSS12	*TMPRSS12*	*Eurypyga helias*	Tetrapoda	XP_010146883.1	XM_010148581.1	104507336	NCBI RefSeq	275
623	Excalfactoria_chinensis_TMPRSS12	*TMPRSS12*	*Excalfactoria chinensis*	Tetrapoda	XP_072214241.1	XM_072358140.1	140263300	NCBI RefSeq	318

624	Falco_biarmicus_TMPRSS12	*TMPRSS12*	*Falco biarmicus*	Tetrapoda	XP_056178555.1	XM_056322580.1	130141557	NCBI RefSeq	326
625	Falco_naumanni_TMPRSS12	*TMPRSS12*	*Falco naumanni*	Tetrapoda	XP_040437139.1	XM_040581205.1	121081896	NCBI RefSeq	287
626	Falco_punctatus_TMPRSS12	*TMPRSS12*	*Falco punctatus*	Tetrapoda	XP_080224953.1	XM_080368827.1	308055155	NCBI RefSeq	321
627	Falco_rusticolus_TMPRSS12	*TMPRSS12*	*Falco rusticolus*	Tetrapoda	XP_037227181.1	XM_037371284.1	119140198	NCBI RefSeq	287
628	Ficedula_albicollis_TMPRSS12	*TMPRSS12*	*Ficedula albicollis*	Tetrapoda	XP_005062119.1	XM_005062062.1	101810780	NCBI RefSeq	286
629	Fukomys_damarensis_TMPRSS12	*TMPRSS12*	*Fukomys damarensis*	Tetrapoda	XP_033619779.1	XM_033763888.1	117286164	NCBI RefSeq	411
630	Galeopterus_variegatus_TMPRSS12	*TMPRSS12*	*Galeopterus variegatus*	Tetrapoda	XP_008567200.1	XM_008568978.1	103587502	NCBI RefSeq	354
631	Gallus_gallus_TMPRSS12	*TMPRSS12*	*Gallus gallus*	Tetrapoda	XP_040549880.1	XM_040693946.2	426872	NCBI RefSeq	318
632	Gavia_stellata_TMPRSS12	*TMPRSS12*	*Gavia stellata*	Tetrapoda	XP_059688733.1	XM_059832750.1	104252137	NCBI RefSeq	316
633	Gavialis_gangeticus_TMPRSS12	*TMPRSS12*	*Gavialis gangeticus*	Tetrapoda	XP_019366546.1	XM_019511001.1	109293188	NCBI RefSeq	334
634	Gekko_japonicus_TMPRSS12	*TMPRSS12*	*Gekko japonicus*	Tetrapoda	XP_015277158.1	XM_015421672.1	107119211	NCBI RefSeq	297
635	Geospiza_fortis_TMPRSS12	*TMPRSS12*	*Geospiza fortis*	Tetrapoda	XP_005430156.2	XM_005430099.3	102038026	NCBI RefSeq	321
636	Geotrypetes_seraphini_TMPRSS12	*TMPRSS12*	*Geotrypetes seraphini*	Tetrapoda	XP_033791765.1	XM_033935874.1	117356539	NCBI RefSeq	317

637	Giraffa_tippelskirchi_TMPRSS12	*TMPRSS12*	*Giraffa tippelskirchi*	Tetrapoda	XP_081448596.1	XM_081592470.1	145546224	NCBI RefSeq	356
638	Globicephala_melas_TMPRSS12	*TMPRSS12*	*Globicephala melas*	Tetrapoda	XP_060163102.1	XM_060307119.1	115841270	NCBI RefSeq	366
639	Gopherus_evgoodei_TMPRSS12	*TMPRSS12*	*Gopherus evgoodei*	Tetrapoda	XP_030410764.1	XM_030554904.1	115647945	NCBI RefSeq	331
640	Gopherus_flavomarginatus_TMPRSS12	*TMPRSS12*	*Gopherus flavomarginatus*	Tetrapoda	XP_050781333.1	XM_050925376.1	127035468	NCBI RefSeq	209
641	Gorilla_gorilla_gorilla_TMPRSS12	*TMPRSS12*	*Gorilla gorilla gorilla*	Tetrapoda	XP_018893581.1	XM_019038036.3	101132625	NCBI RefSeq	348
642	Grammomys_surdaster_TMPRSS12	*TMPRSS12*	*Grammomys surdaster*	Tetrapoda	XP_028644185.1	XM_028788352.1	114639575	NCBI RefSeq	334
643	Grampus_griseus_TMPRSS12	*TMPRSS12*	*Grampus griseus*	Tetrapoda	XP_082521992.1	XM_082665866.1	309829733	NCBI RefSeq	299
644	Grus_americana_TMPRSS12	*TMPRSS12*	*Grus americana*	Tetrapoda	XP_054662843.1	XM_054806868.1	129197986	NCBI RefSeq	317
645	Grus_grus_TMPRSS12	*TMPRSS12*	*Grus grus*	Tetrapoda	XP_081258604.1	XM_081402478.1	308711339	NCBI RefSeq	244
646	Gymnogyps_californianus_TMPRSS12	*TMPRSS12*	*Gymnogyps californianus*	Tetrapoda	XP_050770021.1	XM_050914064.1	127028359	NCBI RefSeq	305
647	Haemorhous_mexicanus_TMPRSS12	*TMPRSS12*	*Haemorhous mexicanus*	Tetrapoda	XP_059728008.1	XM_059872025.1	132340851	NCBI RefSeq	316
648	Haliaeetus_albicilla_TMPRSS12	*TMPRSS12*	*Haliaeetus albicilla*	Tetrapoda	XP_069662304.1	XM_069806203.1	104317512	NCBI RefSeq	326
649	Haliaeetus_leucocephalus_TMPRSS12	*TMPRSS12*	*Haliaeetus leucocephalus*	Tetrapoda	XP_010564865.1	XM_010566563.1	104831166	NCBI RefSeq	311

650	Halichoerus_grypus_TMPRSS12	*TMPRSS12*	*Halichoerus grypus*	Tetrapoda	XP_077930298.1	XM_078074172.1	118539954	NCBI RefSeq	460
651	Harpia_harpyja_TMPRSS12	*TMPRSS12*	*Harpia harpyja*	Tetrapoda	XP_052665819.1	XM_052809859.1	128151966	NCBI RefSeq	326
652	Heliangelus_exortis_TMPRSS12	*TMPRSS12*	*Heliangelus exortis*	Tetrapoda	XP_071585461.1	XM_071729360.1	139788963	NCBI RefSeq	317
653	Hemicordylus_capensis_TMPRSS12	*TMPRSS12*	*Hemicordylus capensis*	Tetrapoda	XP_053156423.1	XM_053300448.1	128346775	NCBI RefSeq	338
654	Herpailurus_yagouaroundi_TMPRSS12	*TMPRSS12*	*Herpailurus yagouaroundi*	Tetrapoda	XP_040344079.1	XM_040488145.1	121038578	NCBI RefSeq	363
655	Heterocephalus_glaber_TMPRSS12	*TMPRSS12*	*Heterocephalus glaber*	Tetrapoda	XP_021098729.1	XM_021243070.1	101705858	NCBI RefSeq	222
656	Heteronotia_binoei_TMPRSS12	*TMPRSS12*	*Heteronotia binoei*	Tetrapoda	XP_060108454.1	XM_060252471.1	132581289	NCBI RefSeq	319
657	Hippopotamus_amphibius_kiboko_TMPRSS12	*TMPRSS12*	*Hippopotamus amphibius kiboko*	Tetrapoda	XP_057556954.1	XM_057700971.1	130832565	NCBI RefSeq	359
658	Hipposideros_armiger_TMPRSS12	*TMPRSS12*	*Hipposideros armiger*	Tetrapoda	XP_019517834.1	XM_019662289.1	109393257	NCBI RefSeq	355
659	Hirundo_rustica_TMPRSS12	*TMPRSS12*	*Hirundo rustica*	Tetrapoda	XP_039944261.1	XM_040088327.1	120764375	NCBI RefSeq	315
660	Homo_sapiens_TMPRSS12	*TMPRSS12*	*Homo sapiens*	Tetrapoda	NP_872365.2	NM_182559.3	283471	NCBI RefSeq	348
661	Hyaena_hyaena_TMPRSS12	*TMPRSS12*	*Hyaena hyaena*	Tetrapoda	XP_039097270.1	XM_039241339.1	120237876	NCBI RefSeq	531
662	Hyla_sarda_TMPRSS12	*TMPRSS12*	*Hyla sarda*	Tetrapoda	XP_056418677.1	XM_056562702.1	130358837	NCBI RefSeq	317

663	Hylobates_moloch_TMPRSS12	*TMPRSS12*	*Hylobates moloch*	Tetrapoda	XP_058289412.1	XM_058433429.1	116461790	NCBI RefSeq	330
664	Hyperolius_riggenbachi_TMPRSS12	*TMPRSS12*	*Hyperolius riggenbachi*	Tetrapoda	XP_068123455.1	XM_068267354.1	137545761	NCBI RefSeq	314
665	Hyperoodon_ampullatus_TMPRSS12	*TMPRSS12*	*Hyperoodon ampullatus*	Tetrapoda	XP_079571805.1	XM_079715679.1	307060855	NCBI RefSeq	355
666	Ictidomys_tridecemlineatus_TMPRSS12	*TMPRSS12*	*Ictidomys tridecemlineatus*	Tetrapoda	XP_077871973.1	XM_078015847.1	101954911	NCBI RefSeq	400
667	Indicator_indicator_TMPRSS12	*TMPRSS12*	*Indicator indicator*	Tetrapoda	XP_054253050.1	XM_054397075.1	128979009	NCBI RefSeq	451
668	Jaculus_jaculus_TMPRSS12	*TMPRSS12*	*Jaculus jaculus*	Tetrapoda	XP_045008163.1	XM_045152228.1	101606264	NCBI RefSeq	512
669	Kogia_breviceps_TMPRSS12	*TMPRSS12*	*Kogia breviceps*	Tetrapoda	XP_058938807.1	XM_059082824.1	131767468	NCBI RefSeq	366
670	Lagenorhynchus_albirostris_TMPRSS12	*TMPRSS12*	*Lagenorhynchus albirostris*	Tetrapoda	XP_060022478.1	XM_060166495.1	132529674	NCBI RefSeq	366
671	Lagopus_muta_TMPRSS12	*TMPRSS12*	*Lagopus muta*	Tetrapoda	XP_048784588.1	XM_048928631.1	125685536	NCBI RefSeq	318
672	Larus_argentatus_TMPRSS12	*TMPRSS12*	*Larus argentatus*	Tetrapoda	XP_081948007.1	XM_082091881.1	309458478	NCBI RefSeq	368
673	Larus_michahellis_TMPRSS12	*TMPRSS12*	*Larus michahellis*	Tetrapoda	XP_074420887.1	XM_074564786.1	141733838	NCBI RefSeq	332
674	Lathamus_discolor_TMPRSS12	*TMPRSS12*	*Lathamus discolor*	Tetrapoda	XP_065515527.1	XM_065659455.1	136003648	NCBI RefSeq	355
675	Lemur_catta_TMPRSS12	*TMPRSS12*	*Lemur catta*	Tetrapoda	XP_045411123.1	XM_045555167.1	123640559	NCBI RefSeq	360

676	Leopardus_geoffroyi_TMPRSS12	*TMPRSS12*	*Leopardus geoffroyi*	Tetrapoda	XP_045321091.1	XM_045465135.1	123591208	NCBI RefSeq	363
677	Lepidochelys_kempii_TMPRSS12	*TMPRSS12*	*Lepidochelys kempii*	Tetrapoda	XP_073175207.1	XM_073319106.1	140900988	NCBI RefSeq	378
678	Lepidothrix_coronata_TMPRSS12	*TMPRSS12*	*Lepidothrix coronata*	Tetrapoda	XP_017694355.1	XM_017838866.1	108509432	NCBI RefSeq	317
679	Leptodactylus_fuscus_TMPRSS12	*TMPRSS12*	*Leptodactylus fuscus*	Tetrapoda	XP_075122523.1	XM_075266422.1	142196205	NCBI RefSeq	363
680	Leptonychotes_weddellii_TMPRSS12	*TMPRSS12*	*Leptonychotes weddellii*	Tetrapoda	XP_081675088.1	XM_081818962.1	102730953	NCBI RefSeq	296
681	Lepus_europaeus_TMPRSS12	*TMPRSS12*	*Lepus europaeus*	Tetrapoda	XP_062058196.1	XM_062202212.1	133767769	NCBI RefSeq	351
682	Leucopleurus_acutus_TMPRSS12	*TMPRSS12*	*Leucopleurus acutus*	Tetrapoda	XP_082706706.1	XM_082850580.1	309969042	NCBI RefSeq	299
683	Lipotes_vexillifer_TMPRSS12	*TMPRSS12*	*Lipotes vexillifer*	Tetrapoda	XP_007452418.1	XM_007452356.1	103084582	NCBI RefSeq	355
684	Lonchura_striata_TMPRSS12	*TMPRSS12*	*Lonchura striata*	Tetrapoda	XP_077644709.1	XM_077788583.1	110478525	NCBI RefSeq	330
685	Lontra_canadensis_TMPRSS12	*TMPRSS12*	*Lontra canadensis*	Tetrapoda	XP_032732434.1	XM_032876543.1	116878804	NCBI RefSeq	363
686	Loxodonta_africana_TMPRSS12	*TMPRSS12*	*Loxodonta africana*	Tetrapoda	XP_064141231.1	XM_064285161.1	100676006	NCBI RefSeq	317
687	Lutra_lutra_TMPRSS12	*TMPRSS12*	*Lutra lutra*	Tetrapoda	XP_047598299.1	XM_047742343.1	125107355	NCBI RefSeq	363
688	Lynx_canadensis_TMPRSS12	*TMPRSS12*	*Lynx canadensis*	Tetrapoda	XP_032449673.1	XM_032593782.1	115518975	NCBI RefSeq	367

689	Macaca_fascicularis_TMPRSS12	*TMPRSS12*	*Macaca fascicularis*	Tetrapoda	XP_065380280.1	XM_065524208.2	102127248	NCBI RefSeq	366
690	Macaca_mulatta_TMPRSS12	*TMPRSS12*	*Macaca mulatta*	Tetrapoda	XP_015007204.1	XM_015151718.3	693778	NCBI RefSeq	348
691	Macaca_nemestrina_TMPRSS12	*TMPRSS12*	*Macaca nemestrina*	Tetrapoda	XP_011727280.2	XM_011728978.3	105474362	NCBI RefSeq	366
692	Macaca_thibetana_thibetana_TMPRSS12	*TMPRSS12*	*Macaca thibetana thibetana*	Tetrapoda	XP_050604766.1	XM_050748809.1	126931068	NCBI RefSeq	348
693	Malaclemys_terrapin_pileata_TMPRSS12	*TMPRSS12*	*Malaclemys terrapin pileata*	Tetrapoda	XP_053868522.1	XM_054012547.1	128828145	NCBI RefSeq	326
694	Manacus_candei_TMPRSS12	*TMPRSS12*	*Manacus candei*	Tetrapoda	XP_051627396.1	XM_051771436.1	127462644	NCBI RefSeq	319
695	Manacus_vitellinus_TMPRSS12	*TMPRSS12*	*Manacus vitellinus*	Tetrapoda	XP_029821846.1	XM_029965986.1	103760587	NCBI RefSeq	219
696	Mandrillus_leucophaeus_TMPRSS12	*TMPRSS12*	*Mandrillus leucophaeus*	Tetrapoda	XP_011819764.1	XM_011964374.1	105527874	NCBI RefSeq	348
697	Manis_javanica_TMPRSS12	*TMPRSS12*	*Manis javanica*	Tetrapoda	XP_017519172.2	XM_017663683.2	108399120	NCBI RefSeq	359
698	Marmota_flaviventris_TMPRSS12	*TMPRSS12*	*Marmota flaviventris*	Tetrapoda	XP_071465381.1	XM_071609280.1	114103863	NCBI RefSeq	337
699	Marmota_marmota_marmota_TMPRSS12	*TMPRSS12*	*Marmota marmota marmota*	Tetrapoda	XP_048658684.1	XM_048802727.1	107148734	NCBI RefSeq	376
700	Marmota_monax_TMPRSS12	*TMPRSS12*	*Marmota monax*	Tetrapoda	XP_058432112.1	XM_058576129.1	124087048	NCBI RefSeq	325
701	Martes_martes_TMPRSS12	*TMPRSS12*	*Martes martes*	Tetrapoda	XP_080668393.1	XM_080812267.1	308348281	NCBI RefSeq	352

702	Mastomys_coucha_TMPRSS12	*TMPRSS12*	*Mastomys coucha*	Tetrapoda	XP_031214853.1	XM_031358993.1	116082155	NCBI RefSeq	332
703	Mauremys_mutica_TMPRSS12	*TMPRSS12*	*Mauremys mutica*	Tetrapoda	XP_044850752.1	XM_044994817.1	123353595	NCBI RefSeq	331
704	Mauremys_reevesii_TMPRSS12	*TMPRSS12*	*Mauremys reevesii*	Tetrapoda	XP_039370054.1	XM_039514120.1	120390982	NCBI RefSeq	359
705	Meleagris_gallopavo_TMPRSS12	*TMPRSS12*	*Meleagris gallopavo*	Tetrapoda	XP_010718831.2	XM_010720529.4	100542819	NCBI RefSeq	318
706	Meles_meles_TMPRSS12	*TMPRSS12*	*Meles meles*	Tetrapoda	XP_045867780.1	XM_046011824.1	123946397	NCBI RefSeq	547
707	Melopsittacus_undulatus_TMPRSS12	*TMPRSS12*	*Melopsittacus undulatus*	Tetrapoda	XP_033927490.1	XM_034071599.1	101874561	NCBI RefSeq	295
708	Melospiza_georgiana_TMPRSS12	*TMPRSS12*	*Melospiza georgiana*	Tetrapoda	XP_057898195.1	XM_058042212.1	131094557	NCBI RefSeq	316
709	Melospiza_melodia_melodia_TMPRSS12	*TMPRSS12*	*Melospiza melodia melodia*	Tetrapoda	XP_063035402.1	XM_063179332.1	134431341	NCBI RefSeq	316
710	Melozone_crissalis_TMPRSS12	*TMPRSS12*	*Melozone crissalis*	Tetrapoda	XP_054149514.1	XM_054293539.1	128948774	NCBI RefSeq	316
711	Meriones_unguiculatus_TMPRSS12	*TMPRSS12*	*Meriones unguiculatus*	Tetrapoda	XP_021513684.1	XM_021658009.2	110561549	NCBI RefSeq	340
712	Mesocricetus_auratus_TMPRSS12	*TMPRSS12*	*Mesocricetus auratus*	Tetrapoda	XP_040609745.1	XM_040753811.1	101836587	NCBI RefSeq	331
713	Mesoplodon_densirostris_TMPRSS12	*TMPRSS12*	*Mesoplodon densirostris*	Tetrapoda	XP_059969955.1	XM_060113972.1	132499409	NCBI RefSeq	365
714	Mesoplodon_mirus_TMPRSS12	*TMPRSS12*	*Mesoplodon mirus*	Tetrapoda	XP_082492100.1	XM_082635974.1	309812292	NCBI RefSeq	355

715	Microcebus_murinus_TMPRSS12	*TMPRSS12*	*Microcebus murinus*	Tetrapoda	XP_012617604.1	XM_012762150.3	105869960	NCBI RefSeq	349
716	Microtus_ochrogaster_TMPRSS12	*TMPRSS12*	*Microtus ochrogaster*	Tetrapoda	XP_005353948.1	XM_005353891.2	101987129	NCBI RefSeq	341
717	Microtus_oregoni_TMPRSS12	*TMPRSS12*	*Microtus oregoni*	Tetrapoda	XP_041498540.1	XM_041642606.1	121441445	NCBI RefSeq	340
718	Microtus_pennsylvanicus_TMPRSS12	*TMPRSS12*	*Microtus pennsylvanicus*	Tetrapoda	XP_075819678.1	XM_075963563.1	142844760	NCBI RefSeq	340
719	Miniopterus_natalensis_TMPRSS12	*TMPRSS12*	*Miniopterus natalensis*	Tetrapoda	XP_016056526.1	XM_016201040.1	107528117	NCBI RefSeq	426
720	Miniopterus_schreibersii_TMPRSS12	*TMPRSS12*	*Miniopterus schreibersii*	Tetrapoda	XP_080983908.1	XM_081127782.1	308552555	NCBI RefSeq	435
721	Mirounga_angustirostris_TMPRSS12	*TMPRSS12*	*Mirounga angustirostris*	Tetrapoda	XP_064447315.1	XM_064591245.1	123838965	NCBI RefSeq	217
722	Mirounga_leonina_TMPRSS12	*TMPRSS12*	*Mirounga leonina*	Tetrapoda	XP_034857923.1	XM_035002032.1	118008488	NCBI RefSeq	236
723	Mixophyes_fleayi_TMPRSS12	*TMPRSS12*	*Mixophyes fleayi*	Tetrapoda	XP_075055392.1	XM_075199291.1	142141136	NCBI RefSeq	318
724	Molossus_molossus_TMPRSS12	*TMPRSS12*	*Molossus molossus*	Tetrapoda	XP_036107416.1	XM_036251523.1	118623709	NCBI RefSeq	358
725	Molothrus_aeneus_TMPRSS12	*TMPRSS12*	*Molothrus aeneus*	Tetrapoda	XP_066423936.1	XM_066567839.1	136567999	NCBI RefSeq	316
726	Molothrus_ater_TMPRSS12	*TMPRSS12*	*Molothrus ater*	Tetrapoda	XP_054373720.1	XM_054517745.1	118697210	NCBI RefSeq	317
727	Monodon_monoceros_TMPRSS12	*TMPRSS12*	*Monodon monoceros*	Tetrapoda	XP_029062540.1	XM_029206707.1	114886153	NCBI RefSeq	356

728	Moschus_berezovskii_TMPRSS12	*TMPRSS12*	*Moschus berezovskii*	Tetrapoda	XP_055250743.1	XM_055394768.1	129535981	NCBI RefSeq	356
729	Motacilla_alba_alba_TMPRSS12	*TMPRSS12*	*Motacilla alba alba*	Tetrapoda	XP_038020111.1	XM_038164183.1	119712668	NCBI RefSeq	316
730	Muntiacus_reevesi_TMPRSS12	*TMPRSS12*	*Muntiacus reevesi*	Tetrapoda	XP_065791134.1	XM_065935062.1	136167533	NCBI RefSeq	356
731	Mus_caroli_TMPRSS12	*TMPRSS12*	*Mus caroli*	Tetrapoda	XP_021038458.1	XM_021182799.1	110310107	NCBI RefSeq	301
732	Mus_musculus_TMPRSS12	*TMPRSS12*	*Mus musculus*	Tetrapoda	NP_898932.2	NM_183109.4	75002	NCBI RefSeq	336
733	Mus_pahari_TMPRSS12	*TMPRSS12*	*Mus pahari*	Tetrapoda	XP_021073077.1	XM_021217418.1	110335335	NCBI RefSeq	339
734	Muscardinus_avellanarius_TMPRSS12	*TMPRSS12*	*Muscardinus avellanarius*	Tetrapoda	XP_080561016.1	XM_080704890.1	308269528	NCBI RefSeq	410
735	Mustela_erminea_TMPRSS12	*TMPRSS12*	*Mustela erminea*	Tetrapoda	XP_032204086.1	XM_032348195.1	116593764	NCBI RefSeq	426
736	Mustela_lutreola_TMPRSS12	*TMPRSS12*	*Mustela lutreola*	Tetrapoda	XP_059041338.1	XM_059185355.1	131838767	NCBI RefSeq	363
737	Mustela_nigripes_TMPRSS12	*TMPRSS12*	*Mustela nigripes*	Tetrapoda	XP_059260496.1	XM_059404513.1	132020334	NCBI RefSeq	351
738	Mustela_nivalis_TMPRSS12	*TMPRSS12*	*Mustela nivalis*	Tetrapoda	XP_082597440.1	XM_082741314.1	309869729	NCBI RefSeq	363
739	Mustela_putorius_furo_TMPRSS12	*TMPRSS12*	*Mustela putorius furo*	Tetrapoda	XP_004782125.1	XM_004782068.3	101690574	NCBI RefSeq	363
740	Mycteria_americana_TMPRSS12	*TMPRSS12*	*Mycteria americana*	Tetrapoda	XP_075381436.1	XM_075525321.1	142420961	NCBI RefSeq	370

741	Myiozetetes_cayanensis_TMPRSS12	*TMPRSS12*	*Myiozetetes cayanensis*	Tetrapoda	XP_050186933.1	XM_050330976.1	126648226	NCBI RefSeq	322
742	Myodes_glareolus_TMPRSS12	*TMPRSS12*	*Myodes glareolus*	Tetrapoda	XP_048300527.1	XM_048444570.2	125407206	NCBI RefSeq	404
743	Myotis_brandtii_TMPRSS12	*TMPRSS12*	*Myotis brandtii*	Tetrapoda	XP_014397509.1	XM_014542023.1	102240839	NCBI RefSeq	307
744	Myotis_daubentonii_TMPRSS12	*TMPRSS12*	*Myotis daubentonii*	Tetrapoda	XP_059537127.1	XM_059681144.1	132226535	NCBI RefSeq	327
745	Myotis_davidii_TMPRSS12	*TMPRSS12*	*Myotis davidii*	Tetrapoda	XP_006757178.1	XM_006757115.2	102759543	NCBI RefSeq	354
746	Myotis_lucifugus_TMPRSS12	*TMPRSS12*	*Myotis lucifugus*	Tetrapoda	XP_006091647.1	XM_006091585.3	102437194	NCBI RefSeq	327
747	Myotis_myotis_TMPRSS12	*TMPRSS12*	*Myotis myotis*	Tetrapoda	XP_036152469.1	XM_036296576.1	118649482	NCBI RefSeq	244
748	Myotis_nattereri_TMPRSS12	*TMPRSS12*	*Myotis nattereri*	Tetrapoda	XP_080938757.1	XM_081082631.1	308512917	NCBI RefSeq	327
749	Myotis_yumanensis_TMPRSS12	*TMPRSS12*	*Myotis yumanensis*	Tetrapoda	XP_070264909.1	XM_070408808.1	139006430	NCBI RefSeq	327
750	Nannospalax_galili_TMPRSS12	*TMPRSS12*	*Nannospalax galili*	Tetrapoda	XP_008827127.2	XM_008828905.2	103730633	NCBI RefSeq	337
751	Nanorana_parkeri_TMPRSS12	*TMPRSS12*	*Nanorana parkeri*	Tetrapoda	XP_018420029.1	XM_018564527.1	108793548	NCBI RefSeq	344
752	Natator_depressus_TMPRSS12	*TMPRSS12*	*Natator depressus*	Tetrapoda	XP_074791650.1	XM_074935549.1	141975253	NCBI RefSeq	343
753	Neofelis_nebulosa_TMPRSS12	*TMPRSS12*	*Neofelis nebulosa*	Tetrapoda	XP_058538214.1	XM_058682231.1	131483848	NCBI RefSeq	400

754	Neogale_vison_TMPRSS12	*TMPRSS12*	*Neogale vison*	Tetrapoda	XP_044083586.1	XM_044227651.1	122891744	NCBI RefSeq	363
755	Neomonachus_schauinslandi_TMPRSS12	*TMPRSS12*	*Neomonachus schauinslandi*	Tetrapoda	XP_021560521.1	XM_021704846.1	110593558	NCBI RefSeq	365
756	Neopelma_chrysocephalum_TMPRSS12	*TMPRSS12*	*Neopelma chrysocephalum*	Tetrapoda	XP_027554869.1	XM_027699068.1	113975245	NCBI RefSeq	319
757	Neophocaena_asiaeorientalis_asiaeorientalis_TMPRSS12	*TMPRSS12*	*Neophocaena asiaeorientalis asiaeorientalis*	Tetrapoda	XP_024592191.1	XM_024736423.1	112393997	NCBI RefSeq	356
758	Neopsephotus_bourkii_TMPRSS12	*TMPRSS12*	*Neopsephotus bourkii*	Tetrapoda	XP_061203236.1	XM_061347252.1	133208965	NCBI RefSeq	320
759	Nesoenas_mayeri_TMPRSS12	*TMPRSS12*	*Nesoenas mayeri*	Tetrapoda	XP_080216701.1	XM_080360575.1	308050458	NCBI RefSeq	315
760	Nipponia_nippon_TMPRSS12	*TMPRSS12*	*Nipponia nippon*	Tetrapoda	XP_009462578.1	XM_009464303.1	104011987	NCBI RefSeq	335
761	Nomascus_leucogenys_TMPRSS12	*TMPRSS12*	*Nomascus leucogenys*	Tetrapoda	XP_003252199.1	XM_003252151.3	100589300	NCBI RefSeq	344
762	Notamacropus_eugenii_TMPRSS12	*TMPRSS12*	*Notamacropus eugenii*	Tetrapoda	XP_072510781.1	XM_072654680.1	140534063	NCBI RefSeq	316
763	Nothoprocta_perdicaria_TMPRSS12	*TMPRSS12*	*Nothoprocta perdicaria*	Tetrapoda	XP_025898592.1	XM_026042807.1	112950305	NCBI RefSeq	313
764	Numenius_arquata_TMPRSS12	*TMPRSS12*	*Numenius arquata*	Tetrapoda	XP_074020945.1	XM_074164844.1	141476229	NCBI RefSeq	317
765	Numida_meleagris_TMPRSS12	*TMPRSS12*	*Numida meleagris*	Tetrapoda	XP_021234786.1	XM_021379111.1	110389161	NCBI RefSeq	386
766	Nyctereutes_procyonoides_TMPRSS12	*TMPRSS12*	*Nyctereutes procyonoides*	Tetrapoda	XP_055193132.1	XM_055337157.1	129517373	NCBI RefSeq	351

767	Nyctibius_grandis_TMPRSS12	*TMPRSS12*	*Nyctibius grandis*	Tetrapoda	XP_068265908.1	XM_068409807.1	137668368	NCBI RefSeq	357
768	Nycticebus_coucang_TMPRSS12	*TMPRSS12*	*Nycticebus coucang*	Tetrapoda	XP_053414008.1	XM_053558033.1	128562743	NCBI RefSeq	348
769	Ochotona_curzoniae_TMPRSS12	*TMPRSS12*	*Ochotona curzoniae*	Tetrapoda	XP_040833668.1	XM_040977734.1	121154733	NCBI RefSeq	337
770	Ochotona_princeps_TMPRSS12	*TMPRSS12*	*Ochotona princeps*	Tetrapoda	XP_058529560.1	XM_058673577.1	101516154	NCBI RefSeq	279
771	Odobenus_rosmarus_divergens_TMPRSS12	*TMPRSS12*	*Odobenus rosmarus divergens*	Tetrapoda	XP_012417993.1	XM_012562539.1	101378840	NCBI RefSeq	364
772	Odocoileus_virginianus_TMPRSS12	*TMPRSS12*	*Odocoileus virginianus*	Tetrapoda	XP_070310946.1	XM_070454845.1	110146982	NCBI RefSeq	395
773	Oenanthe_melanoleuca_TMPRSS12	*TMPRSS12*	*Oenanthe melanoleuca*	Tetrapoda	XP_056370988.1	XM_056515013.1	130265725	NCBI RefSeq	318
774	Onychomys_torridus_TMPRSS12	*TMPRSS12*	*Onychomys torridus*	Tetrapoda	XP_036063525.1	XM_036207632.1	118596752	NCBI RefSeq	293
775	Opisthocomus_hoazin_TMPRSS12	*TMPRSS12*	*Opisthocomus hoazin*	Tetrapoda	XP_075301754.1	XM_075445639.1	104326611	NCBI RefSeq	318
776	Orcinus_orca_TMPRSS12	*TMPRSS12*	*Orcinus orca*	Tetrapoda	XP_049550088.1	XM_049694131.1	101271367	NCBI RefSeq	366
777	Ornithorhynchus_anatinus_TMPRSS12	*TMPRSS12*	*Ornithorhynchus anatinus*	Tetrapoda	XP_028928660.1	XM_029072827.2	100093400	NCBI RefSeq	319
778	Orycteropus_afer_afer_TMPRSS12	*TMPRSS12*	*Orycteropus afer afer*	Tetrapoda	XP_007936133.1	XM_007937942.1	103194519	NCBI RefSeq	342
779	Oryctolagus_cuniculus_TMPRSS12	*TMPRSS12*	*Oryctolagus cuniculus*	Tetrapoda	XP_051702257.1	XM_051846297.2	100342281	NCBI RefSeq	370

780	Oryx_dammah_TMPRSS12	*TMPRSS12*	*Oryx dammah*	Tetrapoda	XP_040089898.1	XM_040233964.1	120858105	NCBI RefSeq	356
781	Otolemur_garnettii_TMPRSS12	*TMPRSS12*	*Otolemur garnettii*	Tetrapoda	XP_003790665.1	XM_003790617.1	100946091	NCBI RefSeq	348
782	Ovis_aries_TMPRSS12	*TMPRSS12*	*Ovis aries*	Tetrapoda	XP_027821739.2	XM_027965938.2	101116644	NCBI RefSeq	356
783	Ovis_canadensis_TMPRSS12	*TMPRSS12*	*Ovis canadensis*	Tetrapoda	XP_069438052.1	XM_069581951.1	138436319	NCBI RefSeq	356
784	Oxyura_jamaicensis_TMPRSS12	*TMPRSS12*	*Oxyura jamaicensis*	Tetrapoda	XP_035164799.1	XM_035308908.1	118155571	NCBI RefSeq	315
785	Pan_paniscus_TMPRSS12	*TMPRSS12*	*Pan paniscus*	Tetrapoda	XP_003825916.1	XM_003825868.3	100984038	NCBI RefSeq	348
786	Pan_troglodytes_TMPRSS12	*TMPRSS12*	*Pan troglodytes*	Tetrapoda	XP_522598.3	XM_522598.7	467200	NCBI RefSeq	348
787	Panthera_leo_TMPRSS12	*TMPRSS12*	*Panthera leo*	Tetrapoda	XP_042802460.1	XM_042946526.1	122224547	NCBI RefSeq	363
788	Panthera_onca_TMPRSS12	*TMPRSS12*	*Panthera onca*	Tetrapoda	XP_078306001.1	XM_078449875.2	144616912	NCBI RefSeq	363
789	Panthera_tigris_TMPRSS12	*TMPRSS12*	*Panthera tigris*	Tetrapoda	XP_015400642.2	XM_015545156.2	102965138	NCBI RefSeq	363
790	Papio_anubis_TMPRSS12	*TMPRSS12*	*Papio anubis*	Tetrapoda	XP_009178998.1	XM_009180734.3	101010270	NCBI RefSeq	366
791	Paroedura_picta_TMPRSS12	*TMPRSS12*	*Paroedura picta*	Tetrapoda	XP_077184774.1	XM_077328659.1	143833168	NCBI RefSeq	319
792	Parus_major_TMPRSS12	*TMPRSS12*	*Parus major*	Tetrapoda	XP_015507486.1	XM_015652000.3	107215651	NCBI RefSeq	310

793	Passer_domesticus_TMPRSS12	*TMPRSS12*	*Passer domesticus*	Tetrapoda	XP_064257081.1	XM_064401011.1	135287798	NCBI RefSeq	316
794	Passer_montanus_TMPRSS12	*TMPRSS12*	*Passer montanus*	Tetrapoda	XP_039561803.1	XM_039705869.1	120500678	NCBI RefSeq	366
795	Patagioenas_fasciata_TMPRSS12	*TMPRSS12*	*Patagioenas fasciata*	Tetrapoda	XP_065714889.2	XM_065858817.2	136113664	NCBI RefSeq	315
796	Pelecanus_crispus_TMPRSS12	*TMPRSS12*	*Pelecanus crispus*	Tetrapoda	XP_075581007.1	XM_075724892.1	104030678	NCBI RefSeq	314
797	Pelodiscus_sinensis_TMPRSS12	*TMPRSS12*	*Pelodiscus sinensis*	Tetrapoda	XP_075758156.1	XM_075902041.1	112547131	NCBI RefSeq	246
798	Perognathus_longimembris_pacificus_TMPRSS12	*TMPRSS12*	*Perognathus longimembris pacificus*	Tetrapoda	XP_048198047.1	XM_048342090.1	125348669	NCBI RefSeq	366
799	Peromyscus_californicus_insignis_TMPRSS12	*TMPRSS12*	*Peromyscus californicus insignis*	Tetrapoda	XP_052604921.1	XM_052748961.1	128117099	NCBI RefSeq	342
800	Peromyscus_eremicus_TMPRSS12	*TMPRSS12*	*Peromyscus eremicus*	Tetrapoda	XP_059103317.1	XM_059247334.1	131896602	NCBI RefSeq	342
801	Peromyscus_leucopus_TMPRSS12	*TMPRSS12*	*Peromyscus leucopus*	Tetrapoda	XP_028730224.1	XM_028874391.2	114696590	NCBI RefSeq	342
802	Peromyscus_maniculatus_bairdii_TMPRSS12	*TMPRSS12*	*Peromyscus maniculatus bairdii*	Tetrapoda	XP_042120487.2	XM_042264553.2	102912681	NCBI RefSeq	342
803	Pezoporus_flaviventris_TMPRSS12	*TMPRSS12*	*Pezoporus flaviventris*	Tetrapoda	XP_061299306.1	XM_061443322.1	133263532	NCBI RefSeq	327
804	Pezoporus_occidentalis_TMPRSS12	*TMPRSS12*	*Pezoporus occidentalis*	Tetrapoda	XP_062490148.1	XM_062634164.1	134171693	NCBI RefSeq	294
805	Pezoporus_wallicus_TMPRSS12	*TMPRSS12*	*Pezoporus wallicus*	Tetrapoda	XP_057269911.1	XM_057413928.1	130601688	NCBI RefSeq	327

806	Phacochoerus_africanus_TMPRSS12	*TMPRSS12*	*Phacochoerus africanus*	Tetrapoda	XP_047641667.1	XM_047785711.1	125130336	NCBI RefSeq	364
807	Phaethon_aethereus_TMPRSS12	*TMPRSS12*	*Phaethon aethereus*	Tetrapoda	XP_081867978.1	XM_082011852.1	309414512	NCBI RefSeq	292
808	Phaethon_lepturus_TMPRSS12	*TMPRSS12*	*Phaethon lepturus*	Tetrapoda	XP_010285441.1	XM_010287139.1	104615277	NCBI RefSeq	282
809	Phalacrocorax_aristotelis_TMPRSS12	*TMPRSS12*	*Phalacrocorax aristotelis*	Tetrapoda	XP_074931728.1	XM_075075627.1	142048581	NCBI RefSeq	315
810	Phalacrocorax_carbo_TMPRSS12	*TMPRSS12*	*Phalacrocorax carbo*	Tetrapoda	XP_009498686.2	XM_009500391.2	104041160	NCBI RefSeq	233
811	Phasianus_colchicus_TMPRSS12	*TMPRSS12*	*Phasianus colchicus*	Tetrapoda	XP_031465688.1	XM_031609828.1	116239673	NCBI RefSeq	318
812	Phoca_vitulina_TMPRSS12	*TMPRSS12*	*Phoca vitulina*	Tetrapoda	XP_032276985.1	XM_032421094.1	116642614	NCBI RefSeq	476
813	Phocoena_phocoena_TMPRSS12	*TMPRSS12*	*Phocoena phocoena*	Tetrapoda	XP_065744245.1	XM_065888173.1	136131389	NCBI RefSeq	356
814	Phocoena_sinus_TMPRSS12	*TMPRSS12*	*Phocoena sinus*	Tetrapoda	XP_032502837.1	XM_032646946.1	116761179	NCBI RefSeq	356
815	Phodopus_roborovskii_TMPRSS12	*TMPRSS12*	*Phodopus roborovskii*	Tetrapoda	XP_051045042.1	XM_051189085.1	127225591	NCBI RefSeq	337
816	Phyllostomus_discolor_TMPRSS12	*TMPRSS12*	*Phyllostomus discolor*	Tetrapoda	XP_035872550.1	XM_036016657.1	114513037	NCBI RefSeq	371
817	Phyllostomus_hastatus_TMPRSS12	*TMPRSS12*	*Phyllostomus hastatus*	Tetrapoda	XP_045685343.1	XM_045829387.1	123812611	NCBI RefSeq	373
818	Physeter_macrocephalus_TMPRSS12	*TMPRSS12*	*Physeter macrocephalus*	Tetrapoda	XP_054941609.1	XM_055085634.1	102992411	NCBI RefSeq	366

819	Piliocolobus_tephrosceles_TMPRSS12	*TMPRSS12*	*Piliocolobus tephrosceles*	Tetrapoda	XP_023066904.1	XM_023211136.2	111542003	NCBI RefSeq	348
820	Pipistrellus_kuhlii_TMPRSS12	*TMPRSS12*	*Pipistrellus kuhlii*	Tetrapoda	XP_036312906.1	XM_036457013.1	118729472	NCBI RefSeq	287
821	Pipra_filicauda_TMPRSS12	*TMPRSS12*	*Pipra filicauda*	Tetrapoda	XP_027587978.1	XM_027732177.2	113993738	NCBI RefSeq	319
822	Platalea_leucorodia_TMPRSS12	*TMPRSS12*	*Platalea leucorodia*	Tetrapoda	XP_082188373.1	XM_082332247.1	309600172	NCBI RefSeq	315
823	Plecotus_auritus_TMPRSS12	*TMPRSS12*	*Plecotus auritus*	Tetrapoda	XP_080518744.1	XM_080662618.1	308245395	NCBI RefSeq	298
824	Poecile_atricapillus_TMPRSS12	*TMPRSS12*	*Poecile atricapillus*	Tetrapoda	XP_058717116.1	XM_058861133.1	131590817	NCBI RefSeq	387
825	Pogoniulus_pusillus_TMPRSS12	*TMPRSS12*	*Pogoniulus pusillus*	Tetrapoda	XP_063997631.1	XM_064141561.1	135174309	NCBI RefSeq	339
826	Pongo_abelii_TMPRSS12	*TMPRSS12*	*Pongo abelii*	Tetrapoda	XP_009246000.3	XM_009247725.3	100436307	NCBI RefSeq	347
827	Pongo_pygmaeus_TMPRSS12	*TMPRSS12*	*Pongo pygmaeus*	Tetrapoda	XP_054298798.1	XM_054442823.1	129009602	NCBI RefSeq	348
828	Prinia_subflava_TMPRSS12	*TMPRSS12*	*Prinia subflava*	Tetrapoda	XP_063277097.1	XM_063421027.1	134563190	NCBI RefSeq	315
829	Prionailurus_bengalensis_TMPRSS12	*TMPRSS12*	*Prionailurus bengalensis*	Tetrapoda	XP_043419841.1	XM_043563906.1	122473392	NCBI RefSeq	363
830	Prionailurus_viverrinus_TMPRSS12	*TMPRSS12*	*Prionailurus viverrinus*	Tetrapoda	XP_047722163.1	XM_047866207.1	125170067	NCBI RefSeq	363
831	Propithecus_coquereli_TMPRSS12	*TMPRSS12*	*Propithecus coquereli*	Tetrapoda	XP_012506911.1	XM_012651457.1	105816129	NCBI RefSeq	348

832	Psammomys_obesus_TMPRSS12	*TMPRSS12*	*Psammomys obesus*	Tetrapoda	XP_055483237.1	XM_055627262.1	129691216	NCBI RefSeq	340
833	Pseudophryne_corroboree_TMPRSS12	*TMPRSS12*	*Pseudophryne corroboree*	Tetrapoda	XP_063808326.1	XM_063952256.1	135009213	NCBI RefSeq	245
834	Pseudopipra_pipra_TMPRSS12	*TMPRSS12*	*Pseudopipra pipra*	Tetrapoda	XP_064495083.1	XM_064639013.1	135404297	NCBI RefSeq	319
835	Pseudopodoces_humilis_TMPRSS12	*TMPRSS12*	*Pseudopodoces humilis*	Tetrapoda	XP_005532901.1	XM_005532844.2	102109382	NCBI RefSeq	372
836	Pseudorca_crassidens_TMPRSS12	*TMPRSS12*	*Pseudorca crassidens*	Tetrapoda	XP_067554758.1	XM_067698657.1	137202833	NCBI RefSeq	366
837	Psittacula_echo_TMPRSS12	*TMPRSS12*	*Psittacula echo*	Tetrapoda	XP_080491542.1	XM_080635416.1	308230618	NCBI RefSeq	364
838	Pteronotus_mesoamericanus_TMPRSS12	*TMPRSS12*	*Pteronotus mesoamericanus*	Tetrapoda	XP_054447483.1	XM_054591508.1	129085098	NCBI RefSeq	356
839	Pteropus_alecto_TMPRSS12	*TMPRSS12*	*Pteropus alecto*	Tetrapoda	XP_006916960.1	XM_006916898.1	102885841	NCBI RefSeq	274
840	Pteropus_medius_TMPRSS12	*TMPRSS12*	*Pteropus medius*	Tetrapoda	XP_039738785.1	XM_039882851.1	120618579	NCBI RefSeq	274
841	Pteropus_vampyrus_TMPRSS12	*TMPRSS12*	*Pteropus vampyrus*	Tetrapoda	XP_011366655.1	XM_011368353.1	105297592	NCBI RefSeq	346
842	Puma_concolor_TMPRSS12	*TMPRSS12*	*Puma concolor*	Tetrapoda	XP_025786259.1	XM_025930474.1	112867480	NCBI RefSeq	239
843	Pygoscelis_adeliae_TMPRSS12	*TMPRSS12*	*Pygoscelis adeliae*	Tetrapoda	XP_009319699.1	XM_009321424.1	103914728	NCBI RefSeq	269
844	Pyrgilauda_ruficollis_TMPRSS12	*TMPRSS12*	*Pyrgilauda ruficollis*	Tetrapoda	XP_041324178.1	XM_041468244.1	121353984	NCBI RefSeq	316

845	Ranitomeya_imitator_TMPRSS12	*TMPRSS12*	*Ranitomeya imitator*	Tetrapoda	XP_069615985.1	XM_069759884.1	138671733	NCBI RefSeq	474
846	Ranitomeya_variabilis_TMPRSS12	*TMPRSS12*	*Ranitomeya variabilis*	Tetrapoda	XP_077153959.1	XM_077297844.1	143816894	NCBI RefSeq	347
847	Rattus_norvegicus_TMPRSS12	*TMPRSS12*	*Rattus norvegicus*	Tetrapoda	XP_038936305.1	XM_039080377.2	100362912	NCBI RefSeq	427
848	Rattus_rattus_TMPRSS12	*TMPRSS12*	*Rattus rattus*	Tetrapoda	XP_032746846.1	XM_032890955.1	116890218	NCBI RefSeq	338
849	Rhea_pennata_TMPRSS12	*TMPRSS12*	*Rhea pennata*	Tetrapoda	XP_062453298.1	XM_062597314.1	134152168	NCBI RefSeq	313
850	Rhinoderma_darwinii_TMPRSS12	*TMPRSS12*	*Rhinoderma darwinii*	Tetrapoda	XP_075708039.1	XM_075851924.1	142742317	NCBI RefSeq	315
851	Rhinolophus_ferrumequinum_TMPRSS12	*TMPRSS12*	*Rhinolophus ferrumequinum*	Tetrapoda	XP_032973563.1	XM_033117672.1	117028806	NCBI RefSeq	325
852	Rhinolophus_hipposideros_TMPRSS12	*TMPRSS12*	*Rhinolophus hipposideros*	Tetrapoda	XP_080915091.1	XM_081058965.1	308501881	NCBI RefSeq	356
853	Rhinolophus_sinicus_TMPRSS12	*TMPRSS12*	*Rhinolophus sinicus*	Tetrapoda	XP_019579912.2	XM_019724353.2	109443672	NCBI RefSeq	354
854	Rhinopithecus_bieti_TMPRSS12	*TMPRSS12*	*Rhinopithecus bieti*	Tetrapoda	XP_017747226.1	XM_017891737.1	108541983	NCBI RefSeq	348
855	Rhinopithecus_roxellana_TMPRSS12	*TMPRSS12*	*Rhinopithecus roxellana*	Tetrapoda	XP_010383640.1	XM_010385338.1	104679683	NCBI RefSeq	348
856	Rissa_tridactyla_TMPRSS12	*TMPRSS12*	*Rissa tridactyla*	Tetrapoda	XP_054039072.1	XM_054183097.1	128901244	NCBI RefSeq	337
857	Rousettus_aegyptiacus_TMPRSS12	*TMPRSS12*	*Rousettus aegyptiacus*	Tetrapoda	XP_036091755.1	XM_036235862.1	107514287	NCBI RefSeq	357

858	Saccopteryx_bilineata_TMPRSS12	*TMPRSS12*	*Saccopteryx bilineata*	Tetrapoda	XP_066110483.1	XM_066254386.1	136322317	NCBI RefSeq	410
859	Saccopteryx_leptura_TMPRSS12	*TMPRSS12*	*Saccopteryx leptura*	Tetrapoda	XP_066219332.1	XM_066363235.1	136391525	NCBI RefSeq	355
860	Sagmatias_obliquidens_TMPRSS12	*TMPRSS12*	*Sagmatias obliquidens*	Tetrapoda	XP_026964000.1	XM_027108199.1	113621242	NCBI RefSeq	355
861	Saimiri_boliviensis_TMPRSS12	*TMPRSS12*	*Saimiri boliviensis*	Tetrapoda	XP_003939213.1	XM_003939164.4	101033744	NCBI RefSeq	348
862	Sapajus_apella_TMPRSS12	*TMPRSS12*	*Sapajus apella*	Tetrapoda	XP_032140934.1	XM_032285043.1	116556070	NCBI RefSeq	348
863	Sarcophilus_harrisii_TMPRSS12	*TMPRSS12*	*Sarcophilus harrisii*	Tetrapoda	XP_023360047.1	XM_023504279.2	100930281	NCBI RefSeq	316
864	Sciurus_vulgaris_TMPRSS12	*TMPRSS12*	*Sciurus vulgaris*	Tetrapoda	XP_078995941.1	XM_079139815.1	306661001	NCBI RefSeq	313
865	Serinus_canaria_TMPRSS12	*TMPRSS12*	*Serinus canaria*	Tetrapoda	XP_030086265.1	XM_030230405.1	103823881	NCBI RefSeq	316
866	Seriola_lalandi_dorsalis_tmprss12	*TMPRSS12*	*Seriola lalandi dorsalis*	Teleostei	XP_023283366.1	XM_023427598.1	111670737	NCBI RefSeq	303
867	Sorex_araneus_TMPRSS12	*TMPRSS12*	*Sorex araneus*	Tetrapoda	XP_054980561.1	XM_055124586.1	101536989	NCBI RefSeq	385
868	Sorex_fumeus_TMPRSS12	*TMPRSS12*	*Sorex fumeus*	Tetrapoda	XP_055975968.1	XM_056119993.1	130029124	NCBI RefSeq	727
869	Spermophilus_citellus_TMPRSS12	*TMPRSS12*	*Spermophilus citellus*	Tetrapoda	XP_081166952.1	XM_081310826.1	308658226	NCBI RefSeq	350
870	Sphaerodactylus_townsendi_TMPRSS12	*TMPRSS12*	*Sphaerodactylus townsendi*	Tetrapoda	XP_048343388.1	XM_048487431.1	125427870	NCBI RefSeq	319

871	Stenella_coeruleoalba_TMPRSS12	*TMPRSS12*	*Stenella coeruleoalba*	Tetrapoda	XP_079793625.1	XM_079937499.1	307194402	NCBI RefSeq	390
872	Streptopelia_decaocto_TMPRSS12	*TMPRSS12*	*Streptopelia decaocto*	Tetrapoda	XP_081929581.1	XM_082073455.1	309450523	NCBI RefSeq	315
873	Strigops_habroptila_TMPRSS12	*TMPRSS12*	*Strigops habroptila*	Tetrapoda	XP_030366168.1	XM_030510308.1	115618601	NCBI RefSeq	326
874	Strix_aluco_TMPRSS12	*TMPRSS12*	*Strix aluco*	Tetrapoda	XP_074663640.1	XM_074807539.1	141915731	NCBI RefSeq	309
875	Strix_uralensis_TMPRSS12	*TMPRSS12*	*Strix uralensis*	Tetrapoda	XP_074710087.1	XM_074853986.1	141936743	NCBI RefSeq	309
876	Struthio_camelus_TMPRSS12	*TMPRSS12*	*Struthio camelus*	Tetrapoda	XP_068777643.1	XM_068921542.1	104144039	NCBI RefSeq	279
877	Sturnira_hondurensis_TMPRSS12	*TMPRSS12*	*Sturnira hondurensis*	Tetrapoda	XP_036886871.1	XM_037030976.1	118976468	NCBI RefSeq	358
878	Sturnus_vulgaris_TMPRSS12	*TMPRSS12*	*Sturnus vulgaris*	Tetrapoda	XP_014747958.1	XM_014892472.1	106863123	NCBI RefSeq	234
879	Suncus_etruscus_TMPRSS12	*TMPRSS12*	*Suncus etruscus*	Tetrapoda	XP_049638924.1	XM_049782967.1	126022116	NCBI RefSeq	420
880	Suricata_suricatta_TMPRSS12	*TMPRSS12*	*Suricata suricatta*	Tetrapoda	XP_029811543.1	XM_029955683.1	115305439	NCBI RefSeq	362
881	Sus_scrofa_TMPRSS12	*TMPRSS12*	*Sus scrofa*	Tetrapoda	XP_020946873.1	XM_021091214.1	110260566	NCBI RefSeq	401
882	Sylvia_atricapilla_TMPRSS12	*TMPRSS12*	*Sylvia atricapilla*	Tetrapoda	XP_066193491.1	XM_066337394.1	136372669	NCBI RefSeq	319
883	Symphalangus_syndactylus_TMPRSS12	*TMPRSS12*	*Symphalangus syndactylus*	Tetrapoda	XP_055150859.1	XM_055294884.2	129491079	NCBI RefSeq	346

884	Tachyglossus_aculeatus_TMPRSS12	*TMPRSS12*	*Tachyglossus aculeatus*	Tetrapoda	XP_038609108.1	XM_038753180.1	119933629	NCBI RefSeq	318
885	Taeniopygia_guttata_TMPRSS12	*TMPRSS12*	*Taeniopygia guttata*	Tetrapoda	XP_032600773.3	XM_032744882.3	100222677	NCBI RefSeq	317
886	Talpa_occidentalis_TMPRSS12	*TMPRSS12*	*Talpa occidentalis*	Tetrapoda	XP_054552764.1	XM_054696789.1	119247915	NCBI RefSeq	411
887	Tamandua_tetradactyla_TMPRSS12	*TMPRSS12*	*Tamandua tetradactyla*	Tetrapoda	XP_077024095.1	XM_077167980.1	143689959	NCBI RefSeq	326
888	Tauraco_erythrolophus_TMPRSS12	*TMPRSS12*	*Tauraco erythrolophus*	Tetrapoda	XP_009979826.1	XM_009981524.1	104375226	NCBI RefSeq	281
889	Tenrec_ecaudatus_TMPRSS12	*TMPRSS12*	*Tenrec ecaudatus*	Tetrapoda	XP_075409407.1	XM_075553292.1	142451507	NCBI RefSeq	336
890	Terrapene_triunguis_TMPRSS12	*TMPRSS12*	*Terrapene triunguis*	Tetrapoda	XP_024070540.1	XM_024214772.1	112116979	NCBI RefSeq	305
891	Tetrao_urogallus_TMPRSS12	*TMPRSS12*	*Tetrao urogallus*	Tetrapoda	XP_079404063.1	XM_079547937.1	306956662	NCBI RefSeq	318
892	Theropithecus_gelada_TMPRSS12	*TMPRSS12*	*Theropithecus gelada*	Tetrapoda	XP_025258616.1	XM_025402831.2	112634925	NCBI RefSeq	348
893	Tinamus_guttatus_TMPRSS12	*TMPRSS12*	*Tinamus guttatus*	Tetrapoda	XP_010220834.1	XM_010222532.1	104575200	NCBI RefSeq	313
894	Trachemys_scripta_elegans_TMPRSS12	*TMPRSS12*	*Trachemys scripta elegans*	Tetrapoda	XP_034648890.1	XM_034792999.1	117889135	NCBI RefSeq	326
895	Trachypithecus_francoisi_TMPRSS12	*TMPRSS12*	*Trachypithecus francoisi*	Tetrapoda	XP_033077974.1	XM_033222083.1	117090128	NCBI RefSeq	348
896	Trichechus_manatus_latirostris_TMPRSS12	*TMPRSS12*	*Trichechus manatus latirostris*	Tetrapoda	XP_023598201.1	XM_023742433.1	101357168	NCBI RefSeq	286

897	Tupaia_chinensis_TMPRSS12	*TMPRSS12*	*Tupaia chinensis*	Tetrapoda	XP_006168217.1	XM_006168155.3	102474251	NCBI RefSeq	351
898	Tursiops_truncatus_TMPRSS12	*TMPRSS12*	*Tursiops truncatus*	Tetrapoda	XP_073645393.1	XM_073789292.1	101325086	NCBI RefSeq	366
899	Tympanuchus_pallidicinctus_TMPRSS12	*TMPRSS12*	*Tympanuchus pallidicinctus*	Tetrapoda	XP_052561214.1	XM_052705254.1	128091858	NCBI RefSeq	318
900	Tyto_alba_TMPRSS12	*TMPRSS12*	*Tyto alba*	Tetrapoda	XP_042652687.1	XM_042796753.1	104360587	NCBI RefSeq	322
901	Urocitellus_parryii_TMPRSS12	*TMPRSS12*	*Urocitellus parryii*	Tetrapoda	XP_077655188.1	XM_077799062.1	113195475	NCBI RefSeq	337
902	Ursus_americanus_TMPRSS12	*TMPRSS12*	*Ursus americanus*	Tetrapoda	XP_045656693.1	XM_045800737.1	123794570	NCBI RefSeq	544
903	Ursus_arctos_TMPRSS12	*TMPRSS12*	*Ursus arctos*	Tetrapoda	XP_026357403.3	XM_026501618.4	113257467	NCBI RefSeq	544
904	Ursus_maritimus_TMPRSS12	*TMPRSS12*	*Ursus maritimus*	Tetrapoda	XP_040497883.1	XM_040641949.1	103675481	NCBI RefSeq	352
905	Vanellus_vanellus_TMPRSS12	*TMPRSS12*	*Vanellus vanellus*	Tetrapoda	XP_082352605.1	XM_082496479.1	309682397	NCBI RefSeq	360
906	Vidua_chalybeata_TMPRSS12	*TMPRSS12*	*Vidua chalybeata*	Tetrapoda	XP_053823610.1	XM_053967635.1	128801612	NCBI RefSeq	318
907	Vidua_macroura_TMPRSS12	*TMPRSS12*	*Vidua macroura*	Tetrapoda	XP_053857979.1	XM_054002004.1	128821108	NCBI RefSeq	318
908	Vulpes_lagopus_TMPRSS12	*TMPRSS12*	*Vulpes lagopus*	Tetrapoda	XP_041592654.1	XM_041736720.1	121480277	NCBI RefSeq	351
909	Vulpes_vulpes_TMPRSS12	*TMPRSS12*	*Vulpes vulpes*	Tetrapoda	XP_072621408.1	XM_072765307.1	112921136	NCBI RefSeq	351

910	Xenopus_tropicalis_TMPRSS12	*TMPRSS12*	*Xenopus tropicalis*	Tetrapoda	XP_031751504.1	XM_031895644.1	100127698	NCBI RefSeq	324
911	Zalophus_californianus_TMPRSS12	*TMPRSS12*	*Zalophus californianus*	Tetrapoda	XP_027451056.2	XM_027595255.2	113922883	NCBI RefSeq	351
912	Zonotrichia_albicollis_TMPRSS12	*TMPRSS12*	*Zonotrichia albicollis*	Tetrapoda	XP_074386887.1	XM_074530786.1	106630010	NCBI RefSeq	316
913	Zonotrichia_leucophrys_gambelii_TMPRSS12	*TMPRSS12*	*Zonotrichia leucophrys gambelii*	Tetrapoda	XP_064591045.1	XM_064734975.1	135459224	NCBI RefSeq	316
914	Branchiostoma_lanceolatum_BL96938	trypsin-like	*Branchiostoma lanceolatum*	Outgroup	BL96938_cuf4	BL96938_cuf4	BL96938	Ensembl	262
915	Branchiostoma_lanceolatum_BL97158	trypsin-like	*Branchiostoma lanceolatum*	Outgroup	BL97158_cuf1	BL97158_cuf1	BL97158	Ensembl	345
916	Branchiostoma_lanceolatum_BL23464	trypsin-like	*Branchiostoma lanceolatum*	Outgroup	BL23464_evm0	BL23464_evm0	BL23464	Ensembl	116
917	Branchiostoma_lanceolatum_BL13011	trypsin-like	*Branchiostoma lanceolatum*	Outgroup	BL13011_evm0	BL13011_evm0	BL13011	Ensembl	416
918	Branchiostoma_lanceolatum_BL95919	trypsin-like	*Branchiostoma lanceolatum*	Outgroup	BL95919_evm1	BL95919_evm1	BL95919	Ensembl	496
919	Branchiostoma_lanceolatum_BL00028	trypsin-like	*Branchiostoma lanceolatum*	Outgroup	BL00028_cuf1	BL00028_cuf1	BL00028	Ensembl	196
920	Branchiostoma_lanceolatum_BL30580	trypsin-like	*Branchiostoma lanceolatum*	Outgroup	BL30580_evm0	BL30580_evm0	BL30580	Ensembl	781
921	Branchiostoma_lanceolatum_BL95920	trypsin-like	*Branchiostoma lanceolatum*	Outgroup	BL95920_evm0	BL95920_evm0	BL95920	Ensembl	475
922	Branchiostoma_lanceolatum_BL00082	trypsin-like	*Branchiostoma lanceolatum*	Outgroup	BL00082_cuf1	BL00082_cuf1	BL00082	Ensembl	181
923	Branchiostoma_lanceolatum_BL09271	trypsin-like	*Branchiostoma lanceolatum*	Outgroup	BL09271_evm0	BL09271_evm0	BL09271	Ensembl	395
924	Branchiostoma_lanceolatum_BL13663	trypsin-like	*Branchiostoma lanceolatum*	Outgroup	BL13663_evm0	BL13663_evm0	BL13663	Ensembl	316
925	Branchiostoma_lanceolatum_BL96022	trypsin-like	*Branchiostoma lanceolatum*	Outgroup	BL96022_evm0	BL96022_evm0	BL96022	Ensembl	425
926	Branchiostoma_lanceolatum_BL07494	trypsin-like	*Branchiostom*	Outgroup	BL07494_evm0	BL07494_ev	BL07494	Ensem	653

			a lanceolatum			m0		bl	
927	Branchiostoma_lanceolatum_BL04692	trypsin-like	*Branchiostoma lanceolatum*	Outgroup	BL04692_cuf2	BL04692_cuf2	BL04692	Ensembl	230
928	Branchiostoma_lanceolatum_BL00022	trypsin-like	*Branchiostoma lanceolatum*	Outgroup	BL00022_evm1	BL00022_evm1	BL00022	Ensembl	400
929	Branchiostoma_lanceolatum_BL23320	trypsin-like	*Branchiostoma lanceolatum*	Outgroup	BL23320_cuf0	BL23320_cuf0	BL23320	Ensembl	542
930	Branchiostoma_lanceolatum_BL16600	trypsin-like	*Branchiostoma lanceolatum*	Outgroup	BL16600_evm0	BL16600_evm0	BL16600	Ensembl	555
931	Branchiostoma_lanceolatum_BL07121	trypsin-like	*Branchiostoma lanceolatum*	Outgroup	BL07121_evm2	BL07121_evm2	BL07121	Ensembl	394
932	Ciona_intestinalis_ENSCING00000022292	chymotrypsinogen B-like	*Ciona intestinalis*	Outgroup	ENSCINP00000036482.1	ENSCINT00000032908.1	ENSCING00000022292.1	Ensembl	253
933	Ciona_intestinalis_ENSCING00000013897	trypsin-like	*Ciona intestinalis*	Outgroup	ENSCINP00000025317.2	ENSCINT00000025563.2	ENSCING00000013897.2	Ensembl	375
934	Ciona_intestinalis_ENSCING00000007761	trypsin-like	*Ciona intestinalis*	Outgroup	ENSCINP00000015917.3	ENSCINT00000015917.3	ENSCING00000007761.3	Ensembl	251
935	Ciona_intestinalis_ENSCING00000007451	trypsin-like	*Ciona intestinalis*	Outgroup	ENSCINP00000015303.3	ENSCINT00000015303.3	ENSCING00000007451.3	Ensembl	397
936	Ciona_intestinalis_ENSCING00000010034	trypsin-like	*Ciona intestinalis*	Outgroup	ENSCINP00000020154.3	ENSCINT00000020154.3	ENSCING00000010034.3	Ensembl	266
937	Ciona_intestinalis_ENSCING00000009644	trypsin-like	*Ciona intestinalis*	Outgroup	ENSCINP00000019607.3	ENSCINT00000019607.3	ENSCING00000009644.3	Ensembl	251
938	Ciona_intestinalis_ENSCING00000006812	plasminogen	*Ciona intestinalis*	Outgroup	ENSCINP00000013976.2	ENSCINT00000013976.2	ENSCING00000006812.3	Ensembl	256
939	Ciona_intestinalis_ENSCING00000002556	plasma kallikrein	*Ciona intestinalis*	Outgroup	ENSCINP00000005200.3	ENSCINT00000005200.3	ENSCING00000002556.3	Ensembl	389
940	Ciona_intestinalis_ENSCING00000020357	anionic trypsin-2-like	*Ciona intestinalis*	Outgroup	ENSCINP00000033597.1	ENSCINT00000031820.1	ENSCING00000020357.1	Ensembl	349
941	Ciona_intestinalis_ENSCING00000018117	trypsin-like	*Ciona intestinalis*	Outgroup	ENSCINP00000035333.1	ENSCINT00000032729.1	ENSCING00000018117.1	Ensembl	283
942	Ciona_intestinalis_ENSCING00000003037	trypsin-like	*Ciona intestinalis*	Outgroup	ENSCINP00000006180.3	ENSCINT00000006180.3	ENSCING00000003037.3	Ensembl	264
943	Ciona_intestinalis_ENSCING00000022780	trypsin-like	*Ciona intestinalis*	Outgroup	ENSCINP00000034632.1	ENSCINT00000034979.1	ENSCING00000022780.1	Ensembl	238
944	Ciona_intestinalis_ENSCING00000001	trypsin-like	*Ciona*	Outgroup	ENSCINP0000000	ENSCINT00	ENSCING0000000	Ensem	496

	370		*intestinalis*		2671.3	000002671.3	1370.3	bl	
945	Ciona_intestinalis_ENSCING00000020687	trypsin-like	*Ciona intestinalis*	Outgroup	ENSCINP00000030350.1	ENSCINT00000032308.1	ENSCING00000020687.1	Ensembl	165
946	Ciona_intestinalis_ENSCING00000019075	trypsin-like	*Ciona intestinalis*	Outgroup	ENSCINP00000031261.1	ENSCINT00000030599.1	ENSCING00000019075.1	Ensembl	297
947	Ciona_intestinalis_ENSCING00000003102	trypsin-like	*Ciona intestinalis*	Outgroup	ENSCINP00000025876.2	ENSCINT00000026122.2	ENSCING00000003102.3	Ensembl	263
948	Ciona_intestinalis_ENSCING00000016432	trypsin-like	*Ciona intestinalis*	Outgroup	ENSCINP00000028475.2	ENSCINT00000028721.2	ENSCING00000016432.2	Ensembl	340
949	Ciona_intestinalis_ENSCING00000008363	transmembrane protease serine 12	*Ciona intestinalis*	Outgroup	ENSCINP00000017059.3	ENSCINT00000017059.3	ENSCING00000008363.3	Ensembl	391
950	Ciona_intestinalis_ENSCING00000001883	uncharacterized LOC100179863	*Ciona intestinalis*	Outgroup	ENSCINP00000003791.1	ENSCINT00000003791.1	ENSCING00000001883.1	Ensembl	519
951	Ciona_intestinalis_ENSCING00000015598	trypsin-like	*Ciona intestinalis*	Outgroup	ENSCINP00000027536.2	ENSCINT00000027782.2	ENSCING00000015598.2	Ensembl	190
952	Ciona_intestinalis_ENSCING00000005353	trypsin-like	*Ciona intestinalis*	Outgroup	ENSCINP00000011002.2	ENSCINT00000011002.2	ENSCING00000005353.2	Ensembl	310
953	Petromyzon_marinus_ENSPMAG00000001180	trypsin-like	*Petromyzon marinus*	Outgroup	ENSPMAP00000001309.1	ENSPMAT00000001315.1	ENSPMAG00000001180.1	Ensembl	328
954	Petromyzon_marinus_ENSPMAG00000002621	trypsin-like	*Petromyzon marinus*	Outgroup	ENSPMAP00000002855.1	ENSPMAT00000002869.1	ENSPMAG00000002621.1	Ensembl	213
955	Petromyzon_marinus_ENSPMAG00000003338	trypsin-like	*Petromyzon marinus*	Outgroup	ENSPMAP00000003633.1	ENSPMAT00000003648.1	ENSPMAG00000003338.1	Ensembl	212
956	Petromyzon_marinus_ENSPMAG00000005045	trypsin-like	*Petromyzon marinus*	Outgroup	ENSPMAP00000005522.1	ENSPMAT00000005542.1	ENSPMAG00000005045.1	Ensembl	128
957	Petromyzon_marinus_ENSPMAG00000005051	trypsin-like	*Petromyzon marinus*	Outgroup	ENSPMAP00000005527.1	ENSPMAT00000005547.1	ENSPMAG00000005051.1	Ensembl	98
958	Petromyzon_marinus_ENSPMAG00000005545	trypsin-like	*Petromyzon marinus*	Outgroup	ENSPMAP00000006099.1	ENSPMAT00000006128.1	ENSPMAG00000005545.1	Ensembl	249

959	Petromyzon_marinus_ENSPMAG00000006000	trypsin-like	*Petromyzon marinus*	Outgroup	ENSPMAP00000006611.1	ENSPMAT00000006641.1	ENSPMAG00000006000.1	Ensembl	235
960	Petromyzon_marinus_ENSPMAG00000006960	trypsin-like	*Petromyzon marinus*	Outgroup	ENSPMAP00000007674.1	ENSPMAT00000007708.1	ENSPMAG00000006960.1	Ensembl	186
961	Petromyzon_marinus_ENSPMAG00000008628	trypsin-like	*Petromyzon marinus*	Outgroup	ENSPMAP00000009500.1	ENSPMAT00000009540.1	ENSPMAG00000008628.1	Ensembl	213
962	Petromyzon_marinus_ENSPMAG00000008757	trypsin-like	*Petromyzon marinus*	Outgroup	ENSPMAP00000009645.1	ENSPMAT00000009686.1	ENSPMAG00000008757.1	Ensembl	215
963	Petromyzon_marinus_ENSPMAG00000009244	trypsin-like	*Petromyzon marinus*	Outgroup	ENSPMAP00000010166.1	ENSPMAT00000010211.1	ENSPMAG00000009244.1	Ensembl	258
964	Petromyzon_marinus_ENSPMAG00000009480	trypsin-like	*Petromyzon marinus*	Outgroup	ENSPMAP00000010421.1	ENSPMAT00000010467.1	ENSPMAG00000009480.1	Ensembl	214
965	Petromyzon_marinus_ENSPMAG00000009758	trypsin-like	*Petromyzon marinus*	Outgroup	ENSPMAP00000010727.1	ENSPMAT00000010773.1	ENSPMAG00000009758.1	Ensembl	220
966	Petromyzon_marinus_ENSPMAG00000009793	transmembrane serine protease 3a	*Petromyzon marinus*	Outgroup	ENSPMAP00000010763.1	ENSPMAT00000010809.1	ENSPMAG00000009793.1	Ensembl	389

Note: Protein accession is the primary sequence identifier; nucleotide (CDS) accessions are provided where available.

Table S8. Sequence filtering and alignment quality control for *TMPRSS12* codon alignment.

A. Filtering workflow

Step	Tool / Criterion	Input	Output
Actinopterygian TMPRSS12 (Table S7)	—	—	165 sequences
Non-teleost taxon reduction	Retain *Amia ocellicauda* (Holostei) as the sole non-teleost outgroup	165	160
Fragment removal	Alignment coverage < 30%	160	146
MAFFT alignment	--auto	146 protein	855 aa
pal2nal back-translation	—	Protein alignment + CDS	855 codons
trimAl trimming	-gt 0.3 (> 30% occupancy)	855 codons	303 codons
IQ-TREE phylogeny	ModelFinder BIC → MG+F3X4+R6, ML	146 x 303 codons	Gene tree

B. Quality control metrics

QC Metric	Before Trimming	After Trimming
Sequences	146	146
Alignment length (codons)	855	303
Columns with > 50% gaps	64.1%	0%
Columns with < 10% gaps	31.8%	91.4%
Fragment sequences removed	—	14

Note: Five distant non-teleost actinopterygians (*Acipenser ruthenus*, *Erpetoichthys calabaricus*, *Lepisosteus oculatus*, *Polyodon spathula*, *Polypterus senegalus*) were excluded to focus the analysis on teleosts, and *Amia ocellicauda* (Holostei, sister to Teleostei) was retained as the sole non-teleost outgroup. Fragment sequences removed during filtering were: *Sinocyclocheilus grahami*, *S. anshuiensis*, *S. rhinocerous*, *Esox lucius*, *Anarrhichthys ocellatus*, *Hippoglossus hippoglossus*, *Chelmon rostratus*, *Thunnus albacares*, *Xyrauchen texanus*, *Pleuronectes platessa*, *Triplophysa rosa*, *Pelmatolapia mariae*, *Channa argus*, *Alosa pseudoharengus*. All 14 sequences had alignment coverage < 30% (range 14.3–28.7%), and the shortest retained sequence had 30.3% coverage. trimAl was run with -gt 0.3, retaining alignment columns with ≥ 30% occupancy.

Table S9. Robustness of RELAX results for poeciliid *TMPRSS12* to alignment trimming, taxon sampling, and foreground definition.

Analysis	Condition	Taxa	Codons	Foreground	K	*p*-value	LRT	Result
A0	Baseline	146	303	Stem + crown	0.543	6.9×10^{-5}	15.83	Relaxation
A1	trimAl -gt 0.1 (relaxed trimming)	146	314	Stem + crown	0.551	8.6×10^{-5}	15.41	Relaxation
A2	trimAl -gt 0.5 (strict trimming)	146	303	Stem + crown	0.541	6.5×10^{-5}	15.95	Relaxation
B1	Without *Gambusia affinis*	145	303	Stem + crown	0.540	1.6×10^{-4}	14.27	Relaxation
B2	Without *Fundulus heteroclitus*	145	303	Stem + crown	0.515	5.7×10^{-5}	16.21	Relaxation
B3	Without *Dicentrarchus*	145	303	Stem +	0.547	8.8×10^{-5}	15.37	Relaxation

	labrax			crown				
B4	Without *Aulonocara stuartgranti*	145	303	Stem + crown	0.536	1.6×10^{-4}	14.26	Relaxation
C1	Foreground redefined	146	303	Crown only	0.666	2.9×10^{-3}	8.89	Relaxation
C2	Foreground redefined	146	303	Stem only	0.167	4.3×10^{-4}	12.40	Relaxation
C3	Foreground redefined	146	303	Terminal branches	0.714	5.7×10^{-2}	3.62	Not significant

Note: All RELAX analyses used the same settings (grid-size 250, five starting points). The poeciliid clade remained monophyletic in every re-estimated tree. A0 (stem-plus-crown) is the main RELAX analysis reported in Table S16. A0–A2 and B1–B4 used the poeciliid stem-plus-crown branch set (the stem branch leading to the poeciliid MRCA plus all descendant branches, 17 branches in total) as the foreground. C1–C3 redefined the foreground to test sensitivity to branch definition: C1 used the crown branches alone (all branches descending from the poeciliid MRCA, excluding the stem, 16 branches), C2 used the stem branch alone, and C3 used the nine terminal branches. For each sensitivity analysis, only the targeted input dimension was altered relative to the baseline analysis. When alignment trimming or taxon sampling was modified, the gene tree was re-estimated from the corresponding modified alignment, while the remaining RELAX settings were held constant. The four species removed in B1–B4 represent different phylogenetic positions relative to Poeciliidae: G. affinis is a focal poeciliid species, F. heteroclitus is a non-poeciliid cyprinodontiform relative, and D. labrax and A. stuartgranti are more distantly related teleosts.

Table S10. Testis RNA-seq data sources for four teleost species used in *TMPRSS12* loss validation.

Species	BioProject Accession	Developmental Stage (replicates)	Reference Genome (version)
Gambusia affinis	PRJNA1345902	30 d, 60 d, 90 d<br>(3 replicates per stage, 9 total)	SWU_Gaff_1.0<br>(GCA_019740435.1)
Nothobranchius furzeri	PRJNA1028576 [44]	5, 8, 10, 11 wph<br>(3 replicates per stage, 12 total)	Nfu_20140520<br>Ensembl Release 115
Sebastes schlegelii	PRJNA573572 [45]	Testis stage III, IV, V<br>(3 replicates per stage, 9 total)	ASM1467356v1<br>(GCA_014673565.1)
Takifugu rubripes	PRJNA591733 [46]	Adult testis<br>(3 replicates)	fTakRub1.2<br>Ensembl Release 115

Note: wph, weeks post-hatch. All samples are testis tissue. RNA-seq data for G. affinis were generated in this study (BioProject PRJNA1345902); data for the other three species were downloaded from the NCBI SRA under the BioProject accessions indicated [44–46].

Table S11. Primer sequences and amplification characteristics.

Gene	Assay	Forward primer (5′–3′)	Reverse primer (5′–3′)	Amplicon (bp)	Tm (°C)	Efficiency (%)
β-actin	RT-qPCR	TGAAATTGCAGTTCTCGTTG	CGACCAACAATGGAGGGGAA	102	82.9 ± 0.9	85.2 ± 3.8
tmprss12	RT-qPCR	GGAGTCGACACTTGTCAGGG	TCCACAATTCTCCCCGAAGC	108	79.2 ± 2.1	91.3 ± 9.3
β-actin	RT-PCR	TGGAGAAGATCTGGCATCACAC	GTCAGGATTTTCATGAGGTAGTCTG	337	—	—
tmprss12	RT-PCR	CTGAGCAACTGCTTCATCACC	GCTGGTTTTCGAATAGACTCCTG	301	—	—

Note: Tm (amplicon melting temperature) values for the RT-qPCR assays are reported as mean ± SD from 24 reactions. Amplification efficiencies were estimated from individual fluorescence amplification curves using LinRegPCR [47], following the recommendations of the MIQE 2.0 guidelines [48]. The RT-PCR primer pairs were used to examine amplification products by agarose gel electrophoresis (Figure S3).

Table S12. Domain sequences used in Figure S4 and S5.

No.	Tip label	Gene	Species	Clade	Protein accession	Nucleotide accession (CDS)	GeneID	Database	Protein length (aa)
1	Callorhinchus_milii_acr	*ACR*	*Callorhinchus milii*	Chondrichthyes	XP_042191409.1	XM_042335475.1	103190823	NCBI RefSeq	303
2	Callorhinchus_milii_tmprss12	*TMPRSS12*	*Callorhinchus milii*	Chondrichthyes	XP_042200091.1	XM_042344157.1	103189266	NCBI RefSeq	243
3	Scyliorhinus_canicula_acr	*ACR*	*Scyliorhinus canicula*	Chondrichthyes	XP_038668153.1	XM_038812225.1	119973800	NCBI RefSeq	332
4	Scyliorhinus_canicula_tmprss12	*TMPRSS12*	*Scyliorhinus canicula*	Chondrichthyes	XP_038670763.1	XM_038814835.1	119975214	NCBI RefSeq	360
5	Polypterus_senegalus_tmprss12	*TMPRSS12*	*Polypterus senegalus*	Actinopterygii (non-teleost)	XP_039620590.1	XM_039764656.1	120536330	NCBI RefSeq	287
6	Acipenser_ruthenus_acr	*ACR*	*Acipenser ruthenus*	Actinopterygii (non-teleost)	XP_033888599.1	XM_034032708.1	117419697	NCBI RefSeq	311
7	Acipenser_ruthenus_tmprss12	*TMPRSS12*	*Acipenser ruthenus*	Actinopterygii (non-	XP_033857193.3	XM_034001302.3	117400941	NCBI RefSeq	328

				teleost)					
8	Lepisosteus_oculatus_tmprss12	*TMPRSS 12*	*Lepisosteus oculatus*	Actinopterygii (non-teleost)	XP_015199639.1	XM_015344153.2	102689718	NCBI RefSeq	311
9	Oreochromis_niloticus_tmprss12	*TMPRSS 12*	*Oreochromis niloticus*	Teleostei	XP_019204871.1	XM_019349326.2	100702191	NCBI RefSeq	312
10	Danio_rerio_tmprss12	*TMPRSS 12*	*Danio rerio*	Teleostei	XP_068073014.2	XM_068216913.3	110438336	NCBI RefSeq	325
11	Oryzias_latipes_tmprss12	*TMPRSS 12*	*Oryzias latipes*	Teleostei	XP_004070473.3	XM_004070425.5	101163815	NCBI RefSeq	308
12	Kryptolebias_marmoratus_tmprss12	*TMPRSS 12*	*Kryptolebias marmoratus*	Teleostei	XP_024865063.1	XM_025009295.2	108244580	NCBI RefSeq	306
13	Nothobranchius_furzeri_tmprss12	*TMPRSS 12*	*Nothobranchius furzeri*	Teleostei	XP_015823280.3	XM_015967794.3	107390837	NCBI RefSeq	305
14	Cyprinodon_variegatus_tmprss12	*TMPRSS 12*	*Cyprinodon variegatus*	Teleostei	XP_015246617.1	XM_015391131.1	107095153	NCBI RefSeq	274
15	Fundulus_heteroclitus_tmprss12	*TMPRSS 12*	*Fundulus heteroclitus*	Teleostei	XP_035992733.1	XM_036136840.1	105931750	NCBI RefSeq	304
16	Poeciliopsis_prolifica_tmprss12	*TMPRSS 12*	*Poeciliopsis prolifica*	Teleostei	XP_054905442.1	XM_055049467.1	129372289	NCBI RefSeq	301
17	Gambusia_affinis_tmprss12	*TMPRSS 12*	*Gambusia affinis*	Teleostei	XP_043983774.1	XM_044127839.1	122837479	NCBI RefSeq	302
18	Poecilia_reticulata_tmprss12	*TMPRSS 12*	*Poecilia reticulata*	Teleostei	XP_008411784.1	XM_008413562.2	103467305	NCBI RefSeq	301
19	Xiphophorus_maculatus_tmprss12	*TMPRSS 12*	*Xiphophorus maculatus*	Teleostei	XP_005801464.3	XM_005801407.3	102234008	NCBI RefSeq	329
20	Protopterus_annectens_acra_like	*ACR*	*Protopterus annectens*	Sarcopterygii	XP_043929688.1	XM_044073753.1	122804094	NCBI RefSeq	361
21	Protopterus_annectens_tmprss12a_like	*TMPRSS 12*	*Protopterus annectens*	Sarcopterygii	XP_043945246.1	XM_044089311.1	122816486	NCBI RefSeq	349
22	Latimeria_chalumnae_acr	*ACR*	*Latimeria chalumnae*	Sarcopterygii	XP_014352803.1	XM_014497317.2	102354559	NCBI RefSeq	277
23	Latimeria_chalumnae_tmprss12	*TMPRSS 12*	*Latimeria chalumnae*	Sarcopterygii	XP_064408588.1	XM_064552518.1	102351894	NCBI RefSeq	306
24	Xenopus_tropicalis_ACR	*ACR*	*Xenopus tropicalis*	Tetrapoda	XP_017946452.1	XM_018090963.2	108645681	NCBI RefSeq	394

25	Xenopus_tropicalis_TMPRSS12	*TMPRSS 12*	*Xenopus tropicalis*	Tetrapoda	XP_031751504.1	XM_031895644.1	100127698	NCBI RefSeq	324
26	Anolis_carolinensis_ACR	*ACR*	*Anolis carolinensis*	Tetrapoda	ENSACAP00000040759	ENSACAT00000056286.1	ENSACAG00000040065	Ensembl	656
27	Anolis_carolinensis_TMPRSS12	*TMPRSS 12*	*Anolis carolinensis*	Tetrapoda	XP_062827044.1	XM_062970974.1	103277763	NCBI RefSeq	342
28	Gallus_gallus_ACR	*ACR*	*Gallus gallus*	Tetrapoda	XP_040515801.1	XM_040659867.2	426864	NCBI RefSeq	354
29	Gallus_gallus_TMPRSS12	*TMPRSS 12*	*Gallus gallus*	Tetrapoda	XP_040549880.1	XM_040693946.2	426872	NCBI RefSeq	318
30	Mus_musculus_ACR	*ACR*	*Mus musculus*	Tetrapoda	NP_038483.1	NM_013455.4	11434	NCBI RefSeq	436
31	Mus_musculus_TMPRSS12	*TMPRSS 12*	*Mus musculus*	Tetrapoda	NP_898932.2	NM_183109.4	75002	NCBI RefSeq	336
32	Homo_sapiens_ACR	*ACR*	*Homo sapiens*	Tetrapoda	NP_001088.2	NM_001097.3	49	NCBI RefSeq	421
33	Homo_sapiens_TMPRSS12	*TMPRSS 12*	*Homo sapiens*	Tetrapoda	NP_872365.2	NM_182559.3	283471	NCBI RefSeq	348

Note: Protein accession is the primary sequence identifier; nucleotide (CDS) accessions are provided where available.

Table S13. Assembly quality and transcriptional validation of the *dip2ba–scn8a* interval in four teleost species.

Species	Chromosome	*dip2ba* end	*scn8a* start	Interval (bp)	N bases (%)	RNA-seq read coverage (mean depth)	Gap transcripts tblastn vs *tmprss12*	Interpretation
Gambusia affinis	LG01	19,605,185	19,618,837	13,653	0 (0%)	74.4–137.0	MSTRG.2: 56.0% id, $e=1.2\times10^{-118}$	*tmprss12* transcript
Nothobranchius furzeri	sgr15	19,043,112	19,054,807	11,696	1 (0.01%)	93.9–233.6	MSTRG.2.1: self-match; 91–100% id, $e\approx10^{-132}$	*tmprss12* present
Sebastes schlegelii	CM025831.1	22,082,291	22,092,701	10,411	0 (0%)	12.8–32.8	MSTRG.4.1: no significant hit (E > 10)	No *tmprss12* similarity
Takifugu rubripes	3	9,362,120	9,369,528	7,409	0 (0%)	39.2–47.3	MSTRG.5.1: no significant hit (E > 10) MSTRG.6.1: 30.0% id (20 aa), e=7.0	*dip2ba* 3′ readthrough

Note: Boundary coordinates are defined as in Table S3A (miniprot best-hit, CDS boundaries). Assembly gaps were assessed by extracting the genomic interval between *dip2ba* end and *scn8a* start and counting N bases. RNA-seq read coverage was computed with samtools depth across the full interval, and mean depth is reported as the range across the biological samples of each species. StringTie transcripts assembled at the *dip2ba–scn8a* interval were extracted from merged.gtf and searched against killifish *tmprss12* (XP_015823280.3) using tBLASTn (e-value cutoff = 10); in *G. affinis* and *N. furzeri*, the recovered transcripts correspond to *tmprss12* itself and served as positive controls. In *T. rubripes* and *S. schlegelii*, the two species in which *tmprss12* was not recovered by genome-level searches, the examined transcripts showed no significant protein-level similarity to *tmprss12* (E-values near or above 1), providing no transcriptional evidence of residual *tmprss12* sequence; together with the genomic evidence, this supports lineage-specific loss.

Table S14. OHNOLOGS v2 candidate pairs between human chr12 and chr22 with MCScanX synteny validation.

Gene family	Gene 1 (chr)	Gene 2 (chr)	OS	MCScanX block	N	e-value
Intermediate filament superfamily	*PRPH* (12)	*NEFH*(22)	2	✓	26	1.9×10^{-24}
	KRT84(12)	*NEFH*(22)	2	✓	7	1.0×10^{-4}
	KRT85(12)	*NEFH*(22)	2	—		
	KRT82(12)	*NEFH*(22)	2	—		
	KRT81(12)	*NEFH*(22)	2	—		
	KRT83(12)	*NEFH*(22)	2	—		
	KRT86(12)	*NEFH*(22)	2	—		
	KRT80(12)	*NEFH*(22)	2	—		
RIM-binding protein family	*RIMBP2*(12)	*RIMBP3B*(22)	3	✓	5	1.3×10^{-5}
	RIMBP2(12)	*RIMBP3*(22)	3	—		
	RIMBP2(12)	*RIMBP3C*(22)	3	—		
Solute carrier family (SLC)	*SLC5A1*(22)	*SLC5A8*(12)	2	✓	14	9.5×10^{-10}
	SLC16A8(22)	*SLC16A7*(12)	3	✓	10	2.7×10^{-9}
	SLC5A4(22)	*SLC5A8*(12)	2	—		
S1 serine protease	*ACR*(22)	*TMPRSS12*(12)	3	—		
	TMPRSS12(12)	*TMPRSS6*(22)	1	—		
Armadillo repeat-containing protein family	*PKP2*(12)	*ARVCF*(22)	2	✓	4	5.8×10^{-5}
Inwardly rectifying potassium channel (KCNJ)	*KCNJ4*(22)	*KCNJ8*(12)	2	✓	7	6.2×10^{-7}
Matrix metalloproteinase family	*MMP11*(22)	*MMP17*(12)	3	✓	5	1.3×10^{-5}
Plexin family	*PLXNB2*(22)	*PLXNC1*(12)	3	—		

Note: OHNOLOGS pairs were retrieved from OHNOLOGS v2 [49] at the relaxed level (q(out) < 0.05). MCScanX blocks were detected with relaxed parameters (−s 3, −m 50, −e 1×10^{-3}). OS = outgroup support (number of outgroup species supporting the ohnolog pair). N = number of gene pairs in the collinear block. e-value = statistical significance of the collinear block (the probability of observing ≥N collinear gene pairs by chance under a genome-wide null model). ✓ indicates the gene pair falls within a detected MCScanX collinear block; — indicates no block detected. The one-to-eight pairing pattern between *NEFH* (chr22) and the *KRT*/*PRPH* cluster (chr12) reflects a single WGD-derived ohnolog relationship followed by tandem expansion on chr12.

Table S15. Node support values for the *ACR–TMPRSS12* duplication and major clades.

Node / Relationship	Support (SH-aLRT / UFboot)	Source
ACR–TMPRSS12 duplication node	100 / 100	IQ-TREE
ACR clade	100 / 100	IQ-TREE
TMPRSS12 clade	84.5 / 99	IQ-TREE
Outgroup clade (53 sequences)	93.0 / 99	IQ-TREE

Note: The 53 outgroup sequences were selected from orthogroup OG0000013, which was identified by OrthoFinder across 30 representative chordate proteomes and comprises 649 sequences spanning multiple S1 serine protease subfamilies, including *TMPRSS, OVCH, PRSS, ACR*, and related family members. The ingroup dataset of 913 sequences, comprising 301 *ACR* and 612 *TMPRSS12* sequences, was assembled independently through the expanded genome searches described in Section 2.1. For phylogenetic rooting, 53 sequences from OG0000013 were selected from three early-diverging chordate lineages, *Ciona intestinalis* (21 sequences), *Branchiostoma lanceolatum* (18), and *Petromyzon marinus* (14), and combined with the 913 ingroup sequences to form the 966-sequence dataset listed in Table S7. These sequences were used collectively as an outgroup set to provide phylogenetic context for rooting the *ACR–TMPRSS12* tree. The two chondrichthyan *ACR* sequences (*Callorhinchus milii* and *Hemiscyllium ocellatum*) are sister to each other and form the basalmost lineage within the *ACR* clade (100/100), whereas the remaining 299 non-chondrichthyan *ACR* sequences form a clade with 98.4/100 support. In the unrooted tree, the two chondrichthyan *ACR* sequences appear as separate arms of the basal trifurcation; when the tree is rooted with the outgroup, they are resolved as the basalmost lineage within the *ACR* clade.

Table S16. Selection pressure analysis summary for poeciliid *TMPRSS12*.

Analysis	Metric	Value
RELAX	K	0.543
	p-value	6.9×10^{-5}
	TEST ω (class 1/2/3)	0.099 / 1.00 / 10.2
	REFERENCE ω (class 1/2/3)	0.014 / 1.00 / 71.8
BUSTED	LRT	248.33
	p-value	< 0.001
MEME	Significant sites (FDR q < 0.05)	20

Note: RELAX used the poeciliid stem-plus-crown branch set (the stem branch leading to the poeciliid MRCA plus all descendant branches, 17 branches in total) as the foreground (test) branch set. BUSTED was applied to all branches in the phylogeny. MEME used Benjamini-Hochberg FDR correction (q < 0.05).

Table S17. RELAX rate class distribution for poeciliid *TMPRSS12*.

Rate class	REFERENCE ω	TEST ω	Proportion
Class 1 (strong purifying)	0.014	0.099	60.4%
Class 2 (neutral)	1.00	1.00	38.5%
Class 3 (positive selection)	71.8	10.2	1.1%

Note: The three rate classes are compressed toward ω = 1 in the TEST (poeciliid stem-plus-crown) branches relative to REFERENCE branches (K = 0.543, $p = 6.9 \times 10^{-5}$), indicating relaxed selection.

Table S18. Branches with significant episodic diversifying selection detected by aBSREL.

Branch	Corrected p	LRT	ω class
Poecilia mexicana	1.71×10^{-9}	49.4	ω > 1
Trematomus bernacchii	< 0.001	112.2	ω > 1

**Branch**	**Corrected** p	**LRT**	**ω class**
Seriola aureovittata	< 0.001	146.0	ω > 1
Dicentrarchus labrax	< 0.001	88.8	ω > 1
Scomber scombrus	< 0.001	54.1	ω > 1
Taurulus bubalis	< 0.001	48.7	ω > 1
Melanostigma gelatinosum	< 0.001	43.0	ω > 1
Maylandia zebra	< 0.001	42.9	ω > 1
Gobio gobio	< 0.001	34.0	ω > 1
Seriola dumerili	< 0.001	35.9	ω > 1
Aulonocara stuartgranti	1.0×10^{-5}	31.9	ω > 1
Phoxinus phoxinus	1.0×10^{-4}	27.1	ω > 1
Brachyistius frenatus	1.7×10^{-4}	26.4	ω > 1
Nematolebias whitei	3.0×10^{-4}	25.4	ω > 1
Oreochromis niloticus	4.0×10^{-4}	24.6	ω > 1
Scomber japonicus	0.0010	22.8	ω > 1
Garra rufa	2.0×10^{-4}	26.2	ω > 1
Acanthochromis polyacanthus	0.0019	21.5	ω > 1
Node81	< 0.001	58.1	ω > 1
Node204	0.0025	21.0	ω > 1
Anarhichas minor	0.0032	20.5	ω > 1
Thunnus maccoyii	0.0064	19.1	ω > 1
Node102	0.0267	16.2	ω > 1
Node113	0.0307	15.9	ω > 1
Leuresthes tenuis	0.0338	15.7	ω > 1
Node266	0.0417	15.3	ω > 1
Node73	0.0466	15.1	ω > 1
Pagrus major	0.0437	15.2	ω > 1

Note: Only branches with Holm-corrected $p < 0.05$ are listed. Branch names follow the IQ-TREE treefile format (terminal branches are named with species_*tmprss12*, internal branches are named NodeXXX). Internal-node labels refer to the following clades: Node81, *Salvelinus*; Node204, Sparidae (*Acanthopagrus latus*, *Pagrus major*, and *Sparus aurata*); Node102, gobioid fishes (*Boleophthalmus pectinirostris*, *Eucyclogobius newberryi*, *Gobius niger*, *Periophthalmus magnuspinnatus*, and *Sphaeramia orbicularis*); Node113, *Thunnus* (*T. maccoyii* and *T. thynnus*); Node266, Poeciliidae (all nine poeciliid species); and Node73, Salmonidae (*Oncorhynchus kisutch*, *O. mykiss*, *O. tshawytscha*, *Salmo trutta*, *Salvelinus alpinus*, and *Salvelinus sp.*). Within Poeciliidae, episodic diversifying selection was restricted to the stem branch (Node266) and one terminal branch (*Poecilia mexicana*); no internal poeciliid (crown) node was significant.

Table S19. MEME significant sites (FDR < 0.05) with codon positions, human residues, and domain assignments.

Codon	Human residue	Domain	Raw p	BH q
5	9	N-term	2.20×10^{-4}	0.00802
8	16		1.40×10^{-3}	0.0282
15	36		1.01×10^{-3}	0.0234
16	37		3.28×10^{-3}	0.0498
19	39		1.89×10^{-3}	0.0336
20	40		1.13×10^{-7}	1.14×10^{-5}
21	41		8.20×10^{-8}	1.14×10^{-5}
23	43		2.22×10^{-15}	6.73×10^{-13}
25	45		7.06×10^{-4}	0.0178
26	46		1.41×10^{-5}	0.000853
57	96	S1 (catalytic)	4.26×10^{-4}	0.0117
73	116		2.38×10^{-4}	0.00802
125	166		3.36×10^{-4}	0.0102
173	213		1.55×10^{-3}	0.0293
202	242		3.13×10^{-3}	0.0498
283	321	C-term	2.08×10^{-3}	0.035
287	326		4.30×10^{-5}	0.00217
293	336		1.31×10^{-3}	0.0282
299	344		1.90×10^{-6}	0.000144
300	345		8.92×10^{-5}	0.00386

Table S20. FUBAR site-specific selection results across all 146 ray-finned fish *TMPRSS12* sequences.

Codon	Human residue	Domain	Prob(α>β) purifying	Prob(α<β) positive	Type
1	1	N-term	0.9943	0.0033	Purifying
4	8	N-term	0.9356	0.0001	Purifying
25	45	N-term	0.9996	0.0001	Purifying
26	46	N-term	0.9895	0.0040	Purifying
27	47	N-term	0.9683	0.0003	Purifying
28	48	N-term	0.9913	0.0017	Purifying
29	49	N-term	0.9705	0.0003	Purifying
31	61	N-term	0.9999	0.0000	Purifying
33	68	N-term	0.9996	0.0000	Purifying
34	73	N-term	1.0000	0.0000	Purifying
35	74	N-term	0.9996	0.0000	Purifying
37	76	N-term	0.9989	0.0002	Purifying
38	77	N-term	0.9999	0.0000	Purifying
39	78	S1	1.0000	0.0000	Purifying
41	80	S1	1.0000	0.0000	Purifying
42	81	S1	1.0000	0.0000	Purifying
45	84	S1	1.0000	0.0000	Purifying
46	85	S1	0.9559	0.0002	Purifying
47	86	S1	0.9230	0.0024	Purifying
48	87	S1	1.0000	0.0000	Purifying
49	88	S1	1.0000	0.0000	Purifying
50	89	S1	0.9810	0.0033	Purifying
51	90	S1	1.0000	0.0000	Purifying
52	91	S1	0.9810	0.0033	Purifying
53	92	S1	0.9906	0.0037	Purifying
54	93	S1	1.0000	0.0000	Purifying
55	94	S1	1.0000	0.0000	Purifying
56	95	S1	0.9989	0.0004	Purifying

61	104	S1	0.9869	0.0002	Purifying
62	105	S1	1.0000	0.0000	Purifying
64	107	S1	1.0000	0.0000	Purifying
65	108	S1	1.0000	0.0000	Purifying
66	109	S1	1.0000	0.0000	Purifying
67	110	S1	1.0000	0.0000	Purifying
68	111	S1	0.9997	0.0001	Purifying
69	112	S1	0.9435	0.0266	Purifying
71	114	S1	0.9382	0.0008	Purifying
74	117	S1	0.9995	0.0002	Purifying
75	118	S1	0.9555	0.0228	Purifying
76	119	S1	0.9999	0.0000	Purifying
77	120	S1	1.0000	0.0000	Purifying
79	122	S1	1.0000	0.0000	Purifying
80	123	S1	1.0000	0.0000	Purifying
81	—	unmapped	0.9990	0.0002	Purifying
92	133	S1	1.0000	0.0000	Purifying
93	134	S1	0.9634	0.0005	Purifying
94	135	S1	0.9998	0.0001	Purifying
96	137	S1	1.0000	0.0000	Purifying
97	138	S1	1.0000	0.0000	Purifying
98	139	S1	0.9687	0.0002	Purifying
99	140	S1	0.9770	0.0002	Purifying
101	142	S1	1.0000	0.0000	Purifying
102	143	S1	1.0000	0.0000	Purifying
105	146	S1	0.9606	0.0194	Purifying
107	148	S1	0.9136	0.0013	Purifying
109	150	S1	0.9996	0.0001	Purifying
111	152	S1	0.9995	0.0002	Purifying
113	154	S1	0.9605	0.0203	Purifying
114	155	S1	0.9636	0.0001	Purifying

118	159	S1	0.9972	0.0000	Purifying
119	160	S1	1.0000	0.0000	Purifying
122	163	S1	1.0000	0.0000	Purifying
123	164	S1	0.9127	0.0002	Purifying
126	167	S1	0.9910	0.0001	Purifying
129	170	S1	1.0000	0.0000	Purifying
130	171	S1	1.0000	0.0000	Purifying
132	173	S1	1.0000	0.0000	Purifying
133	174	S1	1.0000	0.0000	Purifying
136	177	S1	1.0000	0.0000	Purifying
139	180	S1	1.0000	0.0000	Purifying
140	181	S1	0.9991	0.0000	Purifying
141	182	S1	0.0000	0.9914	Positive
142	183	S1	1.0000	0.0000	Purifying
143	184	S1	0.9277	0.0010	Purifying
145	186	S1	0.9950	0.0000	Purifying
146	187	S1	0.9833	0.0074	Purifying
147	188	S1	1.0000	0.0000	Purifying
148	189	S1	1.0000	0.0000	Purifying
150	191	S1	1.0000	0.0000	Purifying
153	—	unmapped	0.9514	0.0010	Purifying
154	194	S1	1.0000	0.0000	Purifying
157	197	S1	1.0000	0.0000	Purifying
158	198	S1	1.0000	0.0000	Purifying
160	200	S1	0.0001	0.9207	Positive
161	201	S1	1.0000	0.0000	Purifying
163	203	S1	0.9910	0.0039	Purifying
164	204	S1	0.9834	0.0075	Purifying
166	206	S1	1.0000	0.0000	Purifying
167	207	S1	0.9999	0.0000	Purifying
169	209	S1	0.9965	0.0000	Purifying

170	210	S1	1.0000	0.0000	Purifying
171	211	S1	0.9974	0.0013	Purifying
172	212	S1	1.0000	0.0000	Purifying
174	214	S1	0.9510	0.0006	Purifying
178	218	S1	1.0000	0.0000	Purifying
180	220	S1	0.0000	0.9477	Positive
181	221	S1	0.9983	0.0000	Purifying
184	224	S1	1.0000	0.0000	Purifying
185	225	S1	0.9963	0.0017	Purifying
186	226	S1	1.0000	0.0000	Purifying
187	227	S1	1.0000	0.0000	Purifying
188	228	S1	0.9942	0.0020	Purifying
189	229	S1	0.9999	0.0000	Purifying
191	231	S1	1.0000	0.0000	Purifying
192	232	S1	1.0000	0.0000	Purifying
193	233	S1	0.9248	0.0447	Purifying
194	234	S1	0.9895	0.0001	Purifying
196	236	S1	0.9993	0.0000	Purifying
197	237	S1	1.0000	0.0000	Purifying
198	238	S1	0.9997	0.0000	Purifying
200	240	S1	0.0001	0.9736	Positive
202	242	S1	0.9561	0.0271	Purifying
203	243	S1	1.0000	0.0000	Purifying
205	245	S1	0.9969	0.0013	Purifying
208	248	S1	0.9995	0.0001	Purifying
209	249	S1	0.9994	0.0000	Purifying
210	250	S1	0.9496	0.0283	Purifying
211	251	S1	0.9831	0.0011	Purifying
213	253	S1	1.0000	0.0000	Purifying
214	254	S1	1.0000	0.0000	Purifying
215	255	S1	1.0000	0.0000	Purifying

218	258	S1	0.9999	0.0000	Purifying
219	259	S1	1.0000	0.0000	Purifying
220	260	S1	0.9932	0.0000	Purifying
221	261	S1	0.9999	0.0000	Purifying
222	262	S1	1.0000	0.0000	Purifying
224	264	S1	1.0000	0.0000	Purifying
226	266	S1	1.0000	0.0000	Purifying
227	267	S1	0.9976	0.0002	Purifying
228	268	S1	1.0000	0.0000	Purifying
229	269	S1	1.0000	0.0000	Purifying
230	270	S1	1.0000	0.0000	Purifying
231	271	S1	1.0000	0.0000	Purifying
232	272	S1	1.0000	0.0000	Purifying
233	273	S1	0.9998	0.0001	Purifying
234	274	S1	1.0000	0.0000	Purifying
235	275	S1	0.9854	0.0042	Purifying
236	276	S1	1.0000	0.0000	Purifying
237	277	S1	0.9895	0.0049	Purifying
238	278	S1	0.9260	0.0016	Purifying
242	282	S1	1.0000	0.0000	Purifying
243	283	S1	1.0000	0.0000	Purifying
245	285	S1	0.9998	0.0000	Purifying
246	286	S1	1.0000	0.0000	Purifying
247	287	S1	0.9705	0.0129	Purifying
248	288	S1	1.0000	0.0000	Purifying
249	289	S1	1.0000	0.0000	Purifying
250	290	S1	1.0000	0.0000	Purifying
251	291	S1	1.0000	0.0000	Purifying
252	292	S1	0.9542	0.0008	Purifying
253	293	S1	0.9209	0.0017	Purifying
254	294	S1	1.0000	0.0000	Purifying

255	295	S1	1.0000	0.0000	Purifying
257	297	S1	0.9987	0.0004	Purifying
259	299	S1	0.9486	0.0053	Purifying
260	300	S1	1.0000	0.0000	Purifying
261	301	S1	1.0000	0.0000	Purifying
262	302	S1	1.0000	0.0000	Purifying
263	303	S1	1.0000	0.0000	Purifying
264	304	S1	0.9567	0.0010	Purifying
265	305	S1	1.0000	0.0000	Purifying
269	309	S1	1.0000	0.0000	Purifying
272	312	S1	0.9536	0.0288	Purifying
274	314	S1	0.9374	0.0014	Purifying
277	—	unmapped	0.9920	0.0030	Purifying
280	318	S1	0.9904	0.0000	Purifying
286	325	C-term	0.9843	0.0001	Purifying
289	329	C-term	0.9941	0.0000	Purifying
298	343	C-term	0.9916	0.0000	Purifying
301	346	C-term	0.9795	0.0005	Purifying
303	348	C-term	0.9247	0.0016	Purifying

Note: Unmapped codons correspond to positions in the alignment that could not be assigned to any human *TMPRSS12* residue (gambusia-specific insertions relative to the human sequence); they were grouped with the non-catalytic regions for the domain comparison.

Table S21. FEL site-specific selection results across all 146 ray-finned fish *TMPRSS12* sequences.

Codon	Human residue	Domain	α (dS)	β (dN)	dN/dS	LRT	*p*-value	Type
1	1	N-term	32.2738	0.0624	0.002	7.6696	5.6158×10^{-3}	Negative
4	8	N-term	2.8926	0.8060	0.279	9.1427	2.4971×10^{-3}	Negative
6	10	N-term	2.0217	0.8609	0.426	6.8590	8.8196×10^{-3}	Negative
11	26	N-term	2.5667	1.1162	0.435	6.2361	1.2517 ×	Negative

							10^{-2}	
22	42	N-term	3.1789	1.4595	0.459	4.5035	3.3825×10^{-2}	Negative
25	45	N-term	1.0510	0.1657	0.158	17.1622	3.4321×10^{-5}	Negative
26	46	N-term	0.6204	0.2104	0.339	6.6222	1.0072×10^{-2}	Negative
28	48	N-term	0.7180	0.2297	0.320	8.0958	4.4367×10^{-3}	Negative
30	51	N-term	0.8458	0.3536	0.418	5.8334	1.5725×10^{-2}	Negative
31	61	N-term	1.7185	0.5320	0.310	15.5949	7.8468×10^{-5}	Negative
33	68	N-term	1.3838	0.3740	0.270	17.2149	3.3381×10^{-5}	Negative
34	73	N-term	0.9765	0.1452	0.149	25.6965	3.9956×10^{-7}	Negative
35	74	N-term	1.1534	0.2425	0.210	19.0345	1.2838×10^{-5}	Negative
37	76	N-term	0.8970	0.2264	0.252	13.9122	1.9155×10^{-4}	Negative
38	77	N-term	0.6968	0.1185	0.170	15.5525	8.0245×10^{-5}	Negative
39	78	S1	1.2772	0.1399	0.110	29.6443	5.1906×10^{-8}	Negative
41	80	S1	0.9108	0.0000	0.000	45.4432	1.5713×10^{-11}	Negative
42	81	S1	1.0267	0.0000	0.000	48.8889	2.7087×10^{-12}	Negative
44	83	S1	0.8322	0.3314	0.398	4.1926	4.0600×10^{-2}	Negative
45	84	S1	0.5685	0.0362	0.064	21.8702	2.9172×10^{-6}	Negative
46	85	S1	1.1432	0.4932	0.431	7.5153	6.1177×10^{-3}	Negative
47	86	S1	1.2705	0.4477	0.352	6.0271	$1.4088 \times$	Negative

							10^{-2}	
48	87	S1	0.8872	0.0190	0.021	37.2895	1.0183×10^{-9}	Negative
49	88	S1	1.3629	0.0556	0.041	48.6204	3.1063×10^{-12}	Negative
51	90	S1	0.8378	0.0000	0.000	46.5801	8.7947×10^{-12}	Negative
53	92	S1	0.3869	0.0690	0.178	5.0844	2.4142×10^{-2}	Negative
54	93	S1	0.7789	0.0601	0.077	23.2921	1.3917×10^{-6}	Negative
55	94	S1	1.5026	0.0000	0.000	54.0903	1.9151×10^{-13}	Negative
56	95	S1	0.6042	0.1094	0.181	10.5604	1.1554×10^{-3}	Negative
62	105	S1	2.3537	0.0000	0.000	84.4717	$< 1 \times 10^{-16}$	Negative
64	107	S1	2.4722	0.0164	0.007	79.4265	$< 1 \times 10^{-16}$	Negative
65	108	S1	2.5021	0.1371	0.055	67.5906	2.2204×10^{-16}	Negative
66	109	S1	1.0626	0.0000	0.000	50.3497	1.2865×10^{-12}	Negative
67	110	S1	2.3098	0.1950	0.084	55.3101	1.0292×10^{-13}	Negative
68	111	S1	0.7309	0.1122	0.153	13.7758	2.0597×10^{-4}	Negative
74	117	S1	0.5746	0.0996	0.173	11.3170	7.6801×10^{-4}	Negative
76	119	S1	1.1542	0.2548	0.221	20.0264	7.6380×10^{-6}	Negative
77	120	S1	1.0126	0.0574	0.057	37.9121	7.4004×10^{-10}	Negative
79	122	S1	0.3879	0.0000	0.000	16.2870	5.4436×10^{-5}	Negative
80	123	S1	0.7702	0.0000	0.000	28.0114	1.2061×10^{-7}	Negative
81	—	unmapped	0.4984	0.0473	0.095	10.0228	$1.5462 \times$	Negative

							10^{-3}	
92	133	S1	1.1906	0.1450	0.122	24.6213	6.9777×10^{-7}	Negative
94	135	S1	0.4665	0.0609	0.131	12.0819	5.0915×10^{-4}	Negative
96	137	S1	0.6877	0.0923	0.134	20.2142	6.9237×10^{-6}	Negative
97	138	S1	0.4421	0.0000	0.000	24.8471	6.2064×10^{-7}	Negative
98	139	S1	1.1346	0.3778	0.333	10.1922	1.4103×10^{-3}	Negative
99	140	S1	1.5777	0.8056	0.511	4.0323	4.4637×10^{-2}	Negative
101	142	S1	1.5802	0.2435	0.154	29.2293	6.4298×10^{-8}	Negative
102	143	S1	1.6500	0.2360	0.143	33.7750	6.1868×10^{-9}	Negative
104	145	S1	0.9348	0.3896	0.417	7.1192	7.6264×10^{-3}	Negative
107	148	S1	1.2521	0.5293	0.423	5.9043	1.5104×10^{-2}	Negative
109	150	S1	0.3033	0.0000	0.000	11.4196	7.2674×10^{-4}	Negative
111	152	S1	0.7933	0.1626	0.205	14.0308	1.7984×10^{-4}	Negative
113	154	S1	0.5748	0.2333	0.406	4.0788	4.3425×10^{-2}	Negative
114	155	S1	1.8179	0.8223	0.452	5.4094	2.0029×10^{-2}	Negative
116	157	S1	1.1143	0.5320	0.477	4.5027	3.3842×10^{-2}	Negative
118	159	S1	2.6439	0.6463	0.244	11.0702	8.7727×10^{-4}	Negative
119	160	S1	1.5764	0.0876	0.056	32.8440	9.9857×10^{-9}	Negative
122	163	S1	1.5855	0.1436	0.090	24.8536	$6.1853 \times$	Negative

							10^{-7}	
123	164	S1	1.8799	0.8478	0.451	6.0025	1.4286×10^{-2}	Negative
125	166	S1	0.7211	1.5796	2.191	5.4254	1.9846×10^{-2}	Positive
126	167	S1	1.2732	0.4549	0.357	10.0266	1.5430×10^{-3}	Negative
129	170	S1	2.6898	0.2311	0.086	41.2275	1.3550×10^{-10}	Negative
130	171	S1	2.9472	0.0000	0.000	104.1935	$< 1 \times 10^{-16}$	Negative
132	173	S1	1.5655	0.2629	0.168	30.3912	3.5313×10^{-8}	Negative
133	174	S1	0.8071	0.0217	0.027	34.6583	3.9296×10^{-9}	Negative
136	177	S1	0.9152	0.0840	0.092	25.0622	5.5511×10^{-7}	Negative
139	180	S1	1.1906	0.0895	0.075	36.7300	1.3568×10^{-9}	Negative
140	181	S1	1.2696	0.2805	0.221	16.1650	5.8056×10^{-5}	Negative
141	182	S1	0.3168	1.1570	3.652	5.7014	1.6951×10^{-2}	Positive
142	183	S1	0.9662	0.0787	0.081	18.9857	1.3170×10^{-5}	Negative
143	184	S1	1.0683	0.4009	0.375	8.6553	3.2612×10^{-3}	Negative
145	186	S1	1.8407	0.4882	0.265	13.8207	2.0111×10^{-4}	Negative
146	187	S1	0.6557	0.2207	0.337	5.9904	1.4384×10^{-2}	Negative
147	188	S1	1.8918	0.0868	0.046	37.8775	7.5329×10^{-10}	Negative
148	189	S1	1.3530	0.0523	0.039	52.8495	3.6016×10^{-13}	Negative
150	191	S1	0.9881	0.0176	0.018	30.0254	4.2642×10^{-8}	Negative

154	194	S1	1.9344	0.2970	0.153	28.4001	9.8662×10^{-8}	Negative
157	197	S1	2.2383	0.2015	0.090	34.7690	3.7123×10^{-9}	Negative
158	198	S1	1.2710	0.0481	0.038	28.8076	7.9938×10^{-8}	Negative
161	201	S1	1.0665	0.0829	0.078	31.0635	2.4973×10^{-8}	Negative
163	203	S1	0.5572	0.1358	0.244	6.5220	1.0655×10^{-2}	Negative
164	204	S1	0.9539	0.2530	0.265	7.9559	4.7931×10^{-3}	Negative
166	206	S1	1.5905	0.0332	0.021	51.6714	6.5614×10^{-13}	Negative
167	207	S1	0.7117	0.0484	0.068	16.1317	5.9086×10^{-5}	Negative
169	209	S1	1.3710	0.4344	0.317	13.7638	2.0729×10^{-4}	Negative
170	210	S1	0.6416	0.0000	0.000	35.2609	2.8836×10^{-9}	Negative
171	211	S1	590.5198	0.0205	0.000	10.6705	1.0886×10^{-3}	Negative
172	212	S1	1.0828	0.0206	0.019	43.3939	4.4758×10^{-11}	Negative
178	218	S1	1.4021	0.0193	0.014	56.0147	7.1942×10^{-14}	Negative
181	221	S1	2.0676	0.6235	0.301	10.0768	1.5015×10^{-3}	Negative
184	224	S1	1.0955	0.0973	0.089	30.4283	3.4644×10^{-8}	Negative
185	225	S1	0.7881	0.1521	0.193	9.3442	2.2369×10^{-3}	Negative
186	226	S1	1.6371	0.0628	0.038	47.2064	6.3890×10^{-12}	Negative
187	227	S1	0.6843	0.0732	0.107	21.0282	4.5258×10^{-6}	Negative

188	228	S1	0.3981	0.0628	0.158	5.7516	1.6474×10^{-2}	Negative
189	229	S1	0.4833	0.0397	0.082	14.7107	1.2533×10^{-4}	Negative
191	231	S1	0.6978	0.0789	0.113	19.0433	1.2779×10^{-5}	Negative
192	232	S1	1.4388	0.0798	0.056	44.4830	2.5657×10^{-11}	Negative
194	234	S1	1.1905	0.3684	0.309	11.1477	8.4137×10^{-4}	Negative
196	236	S1	1.3497	0.4357	0.323	14.2845	1.5715×10^{-4}	Negative
197	237	S1	0.8947	0.0000	0.000	38.1588	6.5216×10^{-10}	Negative
198	238	S1	0.2847	0.0000	0.000	12.4455	4.1901×10^{-4}	Negative
203	243	S1	1.6147	0.0893	0.055	33.0614	8.9292×10^{-9}	Negative
205	245	S1	0.6563	0.1584	0.241	9.6601	1.8831×10^{-3}	Negative
208	248	S1	0.9788	0.2240	0.229	16.5879	4.6447×10^{-5}	Negative
209	249	S1	2.1929	0.4506	0.205	19.0310	1.2861×10^{-5}	Negative
210	250	S1	0.5989	0.2005	0.335	3.9360	4.7262×10^{-2}	Negative
213	253	S1	1.1252	0.0165	0.015	42.7119	6.3425×10^{-11}	Negative
214	254	S1	1.4541	0.0179	0.012	68.4020	1.1102×10^{-16}	Negative
215	255	S1	0.7504	0.0000	0.000	40.3145	2.1620×10^{-10}	Negative
218	258	S1	2.4792	0.5461	0.220	19.7697	8.7357×10^{-6}	Negative
219	259	S1	0.9647	0.0000	0.000	49.5540	1.9299×10^{-12}	Negative

220	260	S1	1.2356	0.5383	0.436	7.3932	6.5472×10^{-3}	Negative
221	261	S1	1.6668	0.4514	0.271	18.8328	1.4269×10^{-5}	Negative
222	262	S1	1.8939	0.0309	0.016	58.1623	2.4092×10^{-14}	Negative
224	264	S1	0.5183	0.0000	0.000	24.7895	6.3945×10^{-7}	Negative
226	266	S1	1.3342	0.0000	0.000	65.5406	5.5511×10^{-16}	Negative
227	267	S1	0.1953	0.0000	0.000	7.9794	4.7311×10^{-3}	Negative
228	268	S1	3.6624	0.0000	0.000	102.0869	$< 1 \times 10^{-16}$	Negative
229	269	S1	0.7683	0.0000	0.000	39.4608	3.3471×10^{-10}	Negative
230	270	S1	1.6485	0.0197	0.012	67.0672	2.2204×10^{-16}	Negative
231	271	S1	0.9809	0.0354	0.036	39.4287	3.4025×10^{-10}	Negative
232	272	S1	0.4750	0.0000	0.000	24.6670	6.8140×10^{-7}	Negative
233	273	S1	0.7166	0.0726	0.101	13.0364	3.0549×10^{-4}	Negative
234	274	S1	1.1947	0.0000	0.000	44.8433	2.1345×10^{-11}	Negative
235	275	S1	0.2913	0.0363	0.124	5.0112	2.5184×10^{-2}	Negative
236	276	S1	1.7042	0.0280	0.016	52.8494	3.6016×10^{-13}	Negative
237	277	S1	0.6180	0.1439	0.233	6.0012	1.4296×10^{-2}	Negative
238	278	S1	1.4616	0.5338	0.365	6.2549	1.2385×10^{-2}	Negative
241	281	S1	0.8723	0.3075	0.352	4.2176	4.0007×10^{-2}	Negative
242	282	S1	0.9196	0.0313	0.034	27.1856	1.8483 ×	Negative

							10^{-7}	
243	283	S1	1.0293	0.0357	0.035	28.7693	8.1531×10^{-8}	Negative
245	285	S1	1.2642	0.2686	0.212	19.0610	1.2661×10^{-5}	Negative
246	286	S1	0.7891	0.0409	0.052	30.3188	3.6656×10^{-8}	Negative
248	288	S1	1.3165	0.0172	0.013	65.2552	6.6613×10^{-16}	Negative
249	289	S1	0.9840	0.0000	0.000	42.5912	6.7461×10^{-11}	Negative
250	290	S1	1.8853	0.0955	0.051	43.7991	3.6387×10^{-11}	Negative
251	291	S1	0.9422	0.0000	0.000	47.9043	4.4753×10^{-12}	Negative
254	294	S1	2.6297	0.0000	0.000	86.4472	$< 1 \times 10^{-16}$	Negative
255	295	S1	0.7304	0.0625	0.086	24.2600	8.4171×10^{-7}	Negative
257	297	S1	0.6798	0.1823	0.268	10.7688	1.0323×10^{-3}	Negative
259	299	S1	0.9067	0.3292	0.363	6.8583	8.8231×10^{-3}	Negative
260	300	S1	1.6789	0.0172	0.010	76.4511	$< 1 \times 10^{-16}$	Negative
261	301	S1	1.0797	0.0190	0.018	44.9332	2.0388×10^{-11}	Negative
262	302	S1	0.6165	0.0605	0.098	16.5114	4.8357×10^{-5}	Negative
263	303	S1	1.8672	0.0183	0.010	55.4360	9.6589×10^{-14}	Negative
264	304	S1	0.9323	0.3414	0.366	9.2950	2.2978×10^{-3}	Negative
265	305	S1	1.0374	0.1471	0.142	24.2032	8.6687×10^{-7}	Negative
269	309	S1	1.3121	0.0837	0.064	32.0478	1.5042×10^{-8}	Negative
277	—	unmapped	1.0856	0.2455	0.226	8.8271	$2.9680 \times$	Negative

							10^{-3}	
282	320	C-term	0.8888	0.3694	0.416	6.6769	9.7668×10^{-3}	Negative
286	325	C-term	1.2967	0.6195	0.478	4.3204	3.7659×10^{-2}	Negative
289	329	C-term	1.3126	0.6974	0.531	4.3285	3.7479×10^{-2}	Negative
301	346	C-term	2.4155	0.7159	0.296	5.7499	1.6489×10^{-2}	Negative
303	348	C-term	1.1218	0.4042	0.360	7.6134	5.7935×10^{-3}	Negative

Note: Only sites with LRT $p < 0.05$ (uncorrected) are listed. Sites not listed should not be interpreted as having been demonstrated to be neutral; they simply did not reach the reporting threshold.

Table S22. Domain enrichment of MEME positive-selection sites across all 146 ray-finned fish *TMPRSS12* sequences.

Domain	Total codons	MEME sites	Permutation p	≥90% cov obs/exp (p)	≥95% cov obs/exp (p)
N-term	37	10	0.042 (enriched)	3 / 0.79 (0.037)	3 / 0.73 (0.030)
S1 (catalytic)	237	5	0.002 (depleted)	5 / 10.95 (≈1)	5 / 10.14 (≈1)
C-term	23	5	0.027 (enriched)	5 / 1.07 (0.002)	4 / 0.95 (0.010)
unmapped	6	0	—	—	—

Note: The expected numbers of MEME sites per domain shown in Figure 5E were calculated by distributing the 20 significant sites in proportion to domain length across 297 mappable codons (37 N-terminal, 237 S1 catalytic domain, and 23 C-terminal codons). To account for variation in alignment coverage, statistical significance was assessed using a coverage-stratified permutation test with 10,000 iterations. The test compared the observed number of significant sites in each domain with a coverage-matched null distribution while preserving the coverage profile of the significant sites, using two-sided tests for enrichment or depletion. Domain-specific *p* values are reported for the three predefined domains. The "≥90% coverage" and "≥95% coverage" categories indicate sites covered by at least 90% and 95% of the 146 sequences, corresponding to 279 and 274 retained sites, respectively.

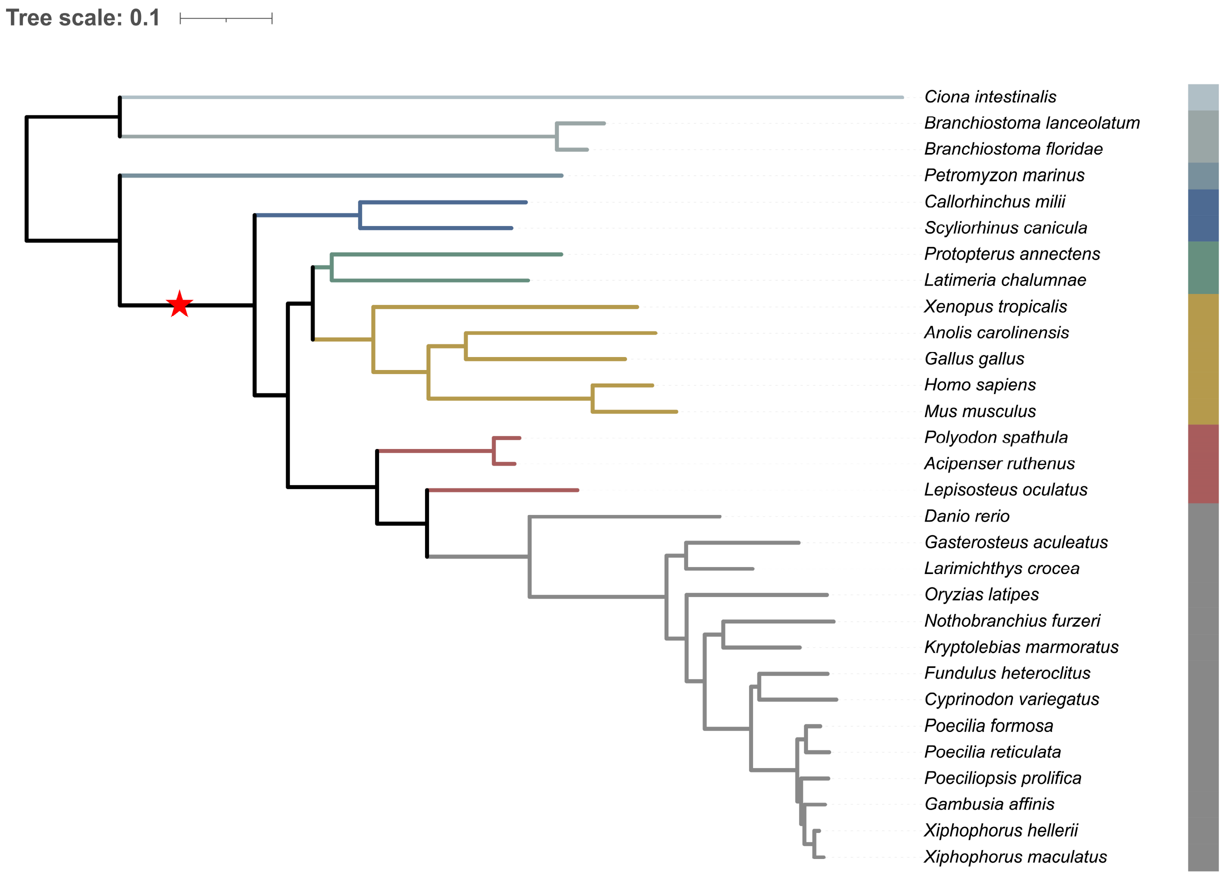

Figure S1. OrthoFinder species tree and the *ACR–TMPRSS12* duplication node.

The rooted species tree of the 30 species analyzed, with cephalochordates (*Branchiostoma floridae* and *Branchiostoma lanceolatum*), the urochordate *Ciona intestinalis*, and the cyclostome *Petromyzon marinus* as outgroups. Colored bars next to each tip show its clade: Cephalochordata, Urochordata, Cyclostomata, Chondrichthyes, Sarcopterygii, Tetrapoda,basal Actinopterygii, and Teleostei. The red star marks the jawed vertebrate ancestor node, where the duplication that separated *ACR* and *TMPRSS12* took place.

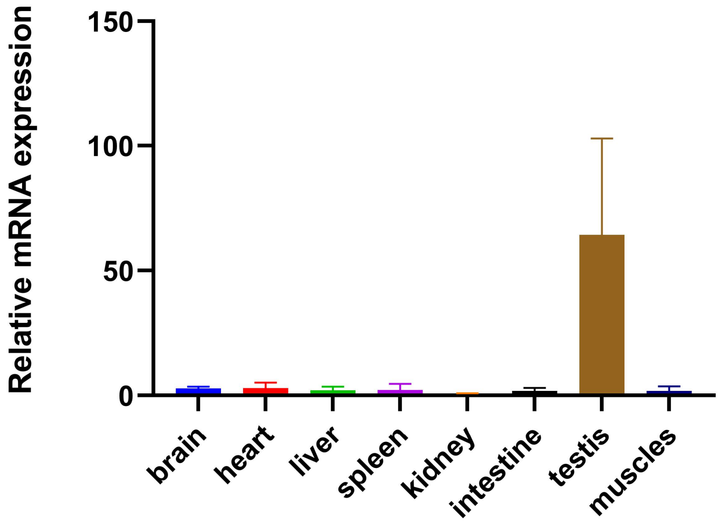

Figure S2. *tmprss12* mRNA expression across eight tissues of adult male *Gambusia affinis*.

RT-qPCR analysis of relative *tmprss12* mRNA expression in brain, heart, liver, spleen, kidney, intestine, testis, and muscle. Tissues were collected from the same three biological individuals. Data are presented as mean ± SD (n = 3 biological replicates). Relative expression was calculated by the $2^{-\Delta\Delta Ct}$ method using *β-actin* as the reference gene and muscle as the calibrator tissue. The mean relative expression levels were 2.86 ± 0.72 in brain, 2.99 ± 2.23 in heart, 2.08 ± 1.54 in liver, 2.16 ± 2.52 in spleen, 0.75 ± 0.18 in kidney, 1.83 ± 1.29 in intestine, 64.28 ± 38.64 in testis, and 1.76 ± 1.97 in muscle.

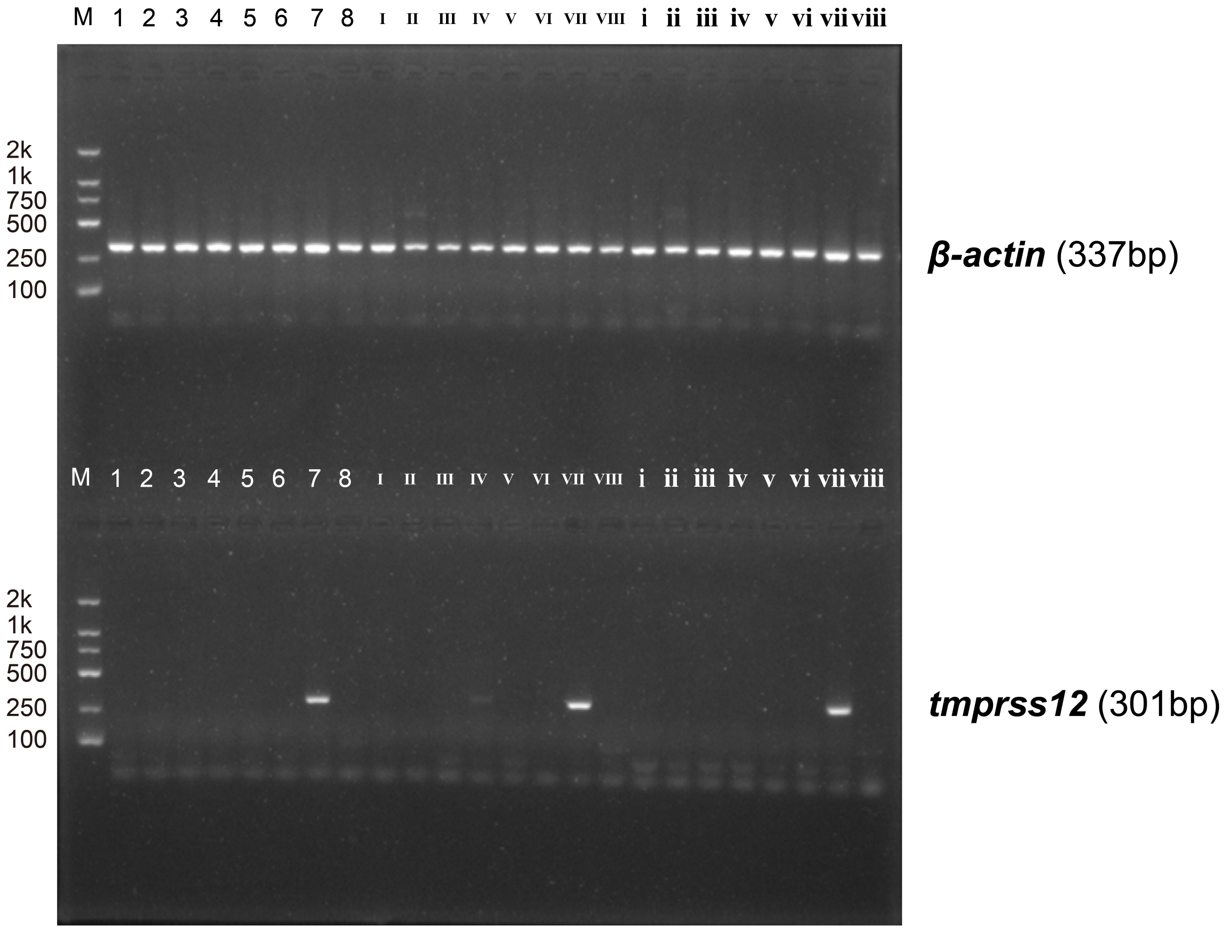

Figure S3. Agarose gel electrophoresis of RT-PCR amplification products of *tmprss12* and *β-actin* in eight tissues of adult male *Gambusia affinis*.

Eight tissues from three adult males were examined. For each individual, the lanes from left to right represent brain, heart, liver, spleen, kidney, intestine, testis, and muscle. Lanes 1–8, I–VIII, and i–viii correspond to the three biological replicates, respectively. The upper panel shows *β-actin* (337 bp), and the lower panel shows *tmprss12* (301 bp). RT-PCR primer sequences were *tmprss12*-F, 5′-CTGAGCAACTGCTTCATCACC-3′; *tmprss12*-R, 5′-GCTGGTTTTCGAATAGACTCCTG-3′; *β-actin*-F, 5′-TGGAGAAGATCTGGCATCACAC-3′; and *β-actin*-R, 5′-GTCAGGATTTTCATGAGGTAGTCTG-3′. M, DNA marker.

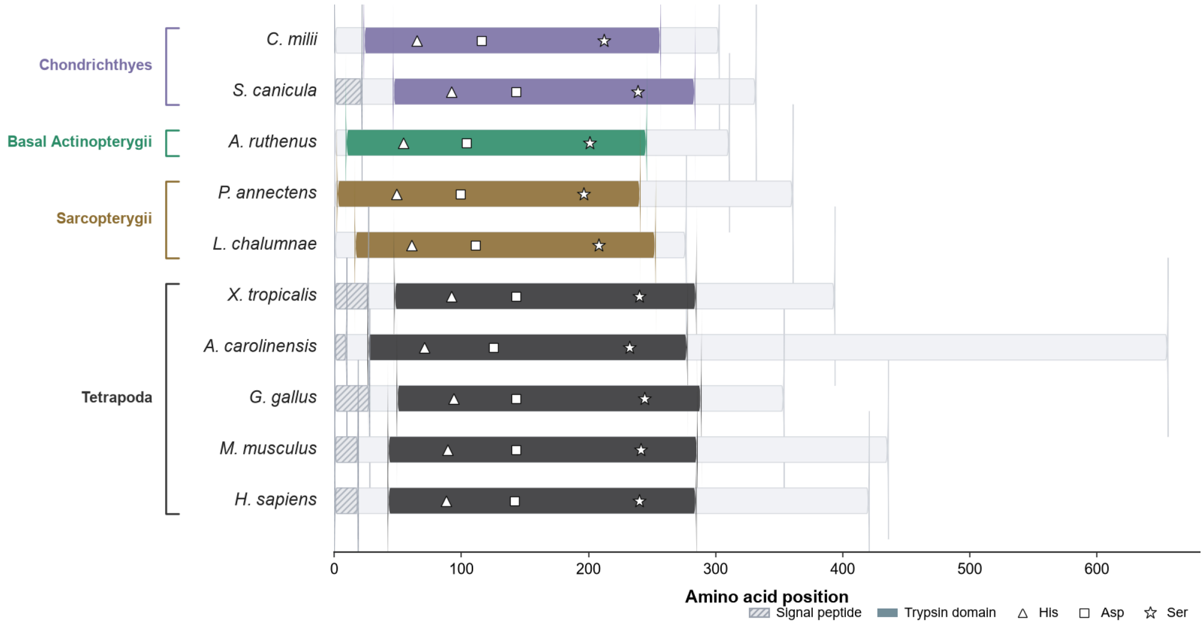

Figure S4. Domain organization of *ACR* proteins from ten vertebrate species.

Each protein is shown as a horizontal bar, with the N-terminal signal peptide, when present, and the trypsin-like serine protease domain (Pfam PF00089) indicated as shown in the legend. The catalytic triad residues are marked at their respective amino acid positions using distinct symbols, with a triangle for His, a square for Asp, and a star for Ser. Colored brackets on the left indicate the taxonomic groups of the species.

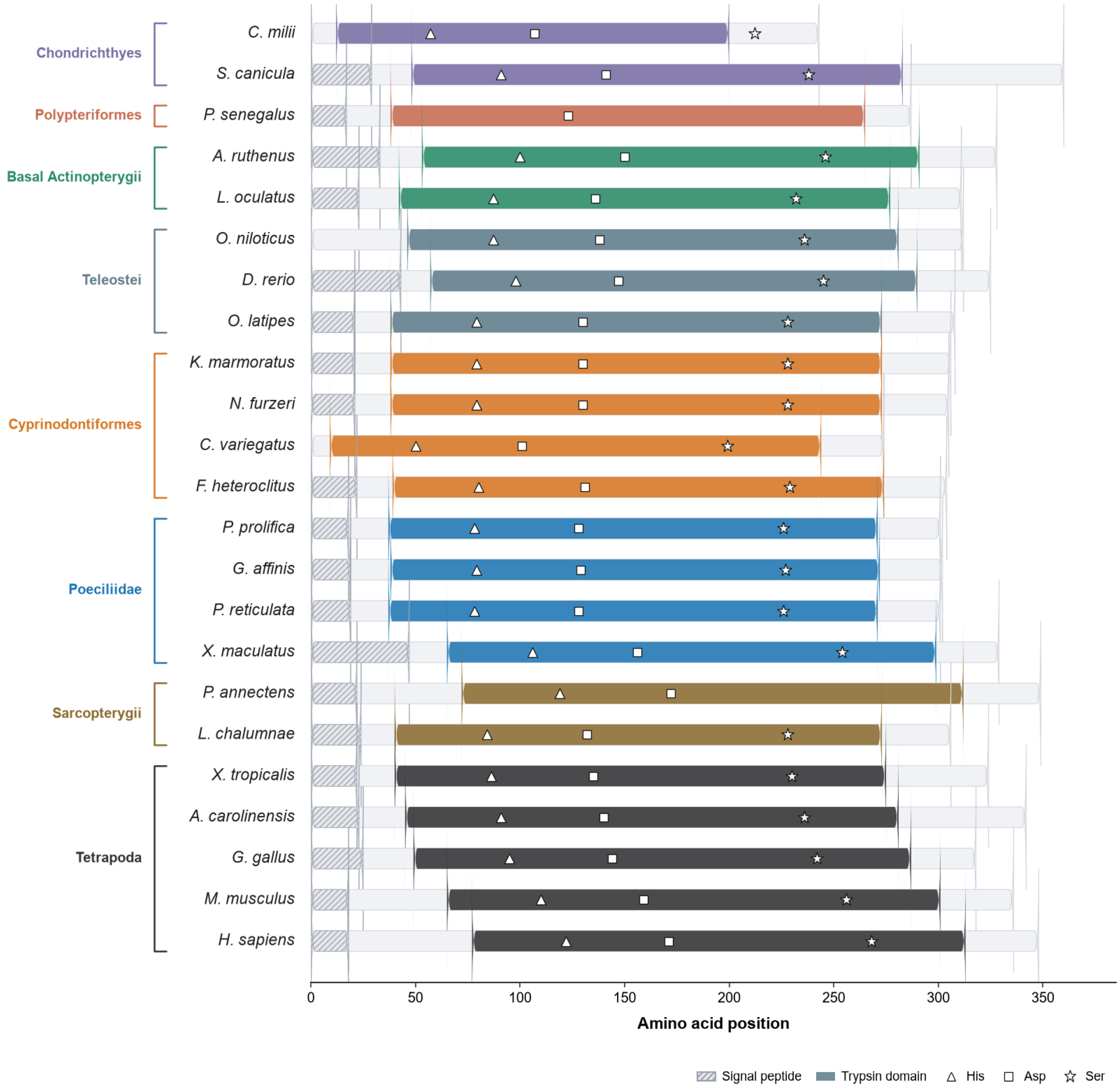

Figure S5. Domain organization of *TMPRSS12* proteins from 23 vertebrate species.

Each protein is shown as a horizontal bar, with the N-terminal signal peptide, when present, and the trypsin-like serine protease domain (Pfam PF00089) indicated as shown in the legend. The canonical catalytic triad residues are marked at their respective amino acid positions using distinct symbols, with a triangle for His, a square for Asp, and a star for Ser. The complete catalytic triad was retained in 21 of the 23 TMPRSS12 proteins. In *Polypterus senegalus*, the canonical catalytic His and Ser residues are replaced by Thr and Glu, respectively, and the corresponding triangle and star are therefore absent. In *Protopterus annectens*, the canonical catalytic Ser residue is replaced by Ile, and the corresponding star is absent. Colored brackets on the left indicate the taxonomic groups of the species.

Reference:

1. Shao, F.; Ludwig, A.; Mao, Y.; Liu, N.; Peng, Z. Chromosome-Level Genome Assembly of the Female Western Mosquitofish (*Gambusia affinis*). *GigaScience* 2020, *9*, giaa092, doi:10.1093/gigascience/giaa092.
2. Holland, N.D.; Holland, L.Z. Fine Structural Study of the Cortical Reaction and Formation of the Egg Coats in a Lancelet (= Amphioxus), *Branchiostoma floridae* (Phylum Chordata: Subphylum Cephalochordata = Acrania). *Biol. Bull.* **1989**, *176*, 111–122, doi:10.2307/1541578.
3. Woollacott, R.M. Spermatozoa of *Ciona intestinalis* and Analysis of Ascidian Fertilization. *J. Morphol.* **1977**, *152*, 77–88, doi:10.1002/jmor.1051520105.
4. Dabrowski, K.; Glogowski, J.; Ciereszko, A. Effects of Proteinase Inhibitors on Fertilization in Sea Lamprey (*Petromyzon marinus*). *Comp. Biochem. Physiol. B Biochem. Mol. Biol.* **2004**, *139*, 157–162, doi:10.1016/j.cbpc.2004.06.010.
5. Venkatesh, B.; Kirkness, E.F.; Loh, Y.-H.; Halpern, A.L.; Lee, A.P.; Johnson, J.; Dandona, N.; Viswanathan, L.D.; Tay, A.; Venter, J.C.; et al. Survey Sequencing and Comparative Analysis of the Elephant Shark (*Callorhinchus milii*) Genome. *PLoS Biol.* **2007**, *5*, e101, doi:10.1371/journal.pbio.0050101.
6. Temple-Smith, P.D.; Ravichandran, A.; Horta Nunez, F.E. Sperm: Comparative Vertebrate. In *Encyclopedia of Reproduction: Volume 6: Comparative Reproduction*, 2nd ed.; Swanson, P.; Skinner, M.K., Eds.; Elsevier: Oxford, UK, 2018; pp. 210–220, doi:10.1016/B978-0-12-809633-8.20558-X.
7. Hornsey, D.J. Permeability Coefficients of the Egg-Case Membrane of *Scyliorhinus canicula* L. *Experientia* **1978**, *34*, 1596–1597, doi:10.1007/BF02034696.
8. Knupp, C.; Chew, M.; Morris, E.; Squire, J. Three-Dimensional Reconstruction of a Collagen IV Analogue in the Dogfish Egg Case Wall. *J. Struct. Biol.* **1996**, *117*, 209–221, doi:10.1006/jsbi.1996.0085.
9. Muñoz-Baquero, M.; Marco-Jiménez, F.; García-Domínguez, X.; Ros-Santaella, J.L.; Pintus, E.; Jiménez-Movilla, M.; García-Párraga, D.; García-Vazquez, F.A. Comparative Study of Semen Parameters and Hormone Profile in Small-Spotted Catshark (*Scyliorhinus canicula*): Aquarium-Housed vs. Wild-Captured. *Animals (Basel)* **2021**, *11*, 2884, doi:10.3390/ani11102884.
10. Bartsch, P.; Britz, R. A Single Micropyle in the Eggs of the Most Basal Living Actinopterygian Fish, *Polypterus* (Actinopterygii, Polypteriformes). *J. Zool.* **1997**, *241*, 589–592, doi:10.1111/j.1469-7998.1997.tb04850.x.
11. Uribe, M.C.; Grier, H.J.; Mejía-Roa, V. Comparative Testicular Structure and Spermatogenesis in Bony Fishes. *Spermatogenesis* **2014**, *4*, e983400, doi:10.4161/21565562.2014.983400.
12. Mattei, X. Spermatozoon Ultrastructure and Its Systematic Implications in Fishes. *Can. J. Zool.* **1991**, 69, 3038–3055, doi:10.1139/z91-428.
13. Mojazi Amiri, B.; Takahashi, H. Fine Structure of Spermatogenesis in a Hybrid Sturgeon, Bester (*Huso huso* Female x *Acipenser ruthenus* Male). *J. Appl. Ichthyol.* **2006**, *22*, 406–410, doi:10.1111/j.1439-0426.2007.00995.x.
14. Żelazowska, M.; Pšenička, M. Secondary Growth Ovarian Follicles of the Pigmented Sterlet Sturgeon *Acipenser ruthenus* L. 1758 (Acipenseriformes, Chondrostei, Actinopterygii, Osteichthyes) – Microscopic Study of Oocytes, Egg Envelope and Diversification of Follicular Cells. *Micron* **2024**, *186*, 103701, doi:10.1016/j.micron.2024.103701.
15. Afzelius, B.A. Fine Structure of the Garfish Spermatozoon. *J. Ultrastruct. Res.* **1978**, *64*, 309–314, doi:10.1016/S0022-5320(78)90039-4.
16. Long, W.L.; Ballard, W.W. Normal Embryonic Stages of the Longnose Gar, *Lepisosteus osseus*. *BMC Dev. Biol.* **2001**, *1*, 6, doi:10.1186/1471-213X-1-6.
17. Lou, Y.-H.; Takahashi, H. Spermiogenesis in the Nile Tilapia *Oreochromis niloticus* with Notes on a Unique Pattern of Nuclear Chromatin Condensation. *J. Morphol.* **1989**, *200*, 321–330, doi:10.1002/jmor.1052000307.

18. Zaki, M.I.; Aziz, F.K.; Abou Shabana, N.M. Ultrastructural Composition of Steroid Producing Cells in Oocyte Wall, and Micropylar Apparatus as a Sperm Guidance System in *Oreochromis niloticus* Reared in Freshwater Environment. *Egypt. J. Aquat. Res.* **2005**, *31*, 297-308.
19. Rupik, W.; Huszno, J.; Klag, J. Cellular Organisation of the Mature Testes and Stages of Spermiogenesis in *Danio rerio* (Cyprinidae; Teleostei)—Structural and Ultrastructural Studies. *Micron* **2011**, *42*, 833–839, doi:10.1016/j.micron.2011.05.006.
20. Yi, X.; Yu, J.; Ma, C.; Dong, G.; Shi, W.; Li, H.; Li, L.; Luo, L.; Sampath, K.; Ruan, H.; et al. The Effector of Hippo Signaling, Taz, Is Required for Formation of the Micropyle and Fertilization in Zebrafish. *PLoS Genet.* **2019**, *15*, e1007408, doi:10.1371/journal.pgen.1007408.
21. Sakai, Y.T. Spermiogenesis of the Teleost, *Oryzias latipes*, with Special Reference to the Formation of Flagellar Membrane. *Dev. Growth Differ.* **1976**, *18*, 1–13, doi:10.1111/j.1440-169X.1976.00001.x.
22. Hosokawa, K. Scanning Electron Microscopic Observations of the Micropyle in *Oryzias latipes*. *Jpn. J. Ichthyol.* **1979**, *26*, 94–99, doi:10.11369/jji1950.26.94.
23. Gao, D.; Zhang, X.; Li, W.; Zhang, P. Comparative Study on the Surface Ultrastructures of Fertilized Eggs of Three *Sebastes* Species. *Mar. Biol. Res.* **2018**, *14*, 345–353, doi:10.1080/17451000.2018.1425457.
24. Zhang, Y.; Qi, X.; Wang, X.; Li, J.; Lü, L.; Yao, Y.; Wen, H. Ultrastructure and Ultra-Low Temperature Freezing Effect of Spermatozoon in Black Rockfish *Sebastes schlegelii*. *J. Dalian Fish. Univ.* **2020**, *35*, 355–359, doi:10.16535/j.cnki.dlhyxb.2020-031.
25. Chang, Y.J.; Chang, Y.J.; Lim, H.K. Physico-Chemical Properties of Milt and Fine Structure of Cryopreserved Spermatozoa in Tiger Puffer (*Takifugu rubripes*). *J. Korean Fish. Soc.* **1998, *31*, 353–358.**
26. Yanagimachi, R.; Harumi, T.; Matsubara, H.; Yan, W.; Yuan, S.; Hirohashi, N.; Iida, T.; Yamaha, E.; Arai, K.; Matsubara, T.; et al. Chemical and Physical Guidance of Fish Spermatozoa into the Egg through the Micropyle†,‡. *Biol. Reprod.* **2017**, *96*, 780–799, doi:10.1093/biolre/iox015.
27. Kweon, H.-S.; Park, E.-H.; Peters, N. Spermatozoon Ultrastructure in the Internally Self-Fertilizing Hermaphroditic Teleost, *Rivulus marmoratus* (Cyprinodontiformes, Rivulidae). *Copeia* **1998**, *1998*, 1101–1106, doi:10.2307/1447367.
28. Sakakura, Y.; Soyano, K.; Noakes, D.; Hagiwara, A. Gonadal Morphology in the Self-Fertilizing Mangrove Killifish, *Kryptolebias marmoratus*. *Ichthyol. Res.* **2006**, *53*, 427–430, doi:10.1007/s10228-006-0362-2.
29. Kwon, J.K.; Jung, H.S.; Kim, D.H. Comparative Ultrastructures of the Fertilized Egg Envelopes in *Nothobranchius guentheri* and *Nothobranchius patrizii*, Nothobranchiidae, Teleostei. *Appl. Microsc.* **2015**, *45*, 144–149, doi:10.9729/AM.2015.45.3.144.
30. Kuntz, A. Notes on the Embryology and Larval Development of Five Species of Teleostean Fishes. *Bull. U. S. Bur. Fish.* **1916**, *34*, 409–429.
31. Teves, M.E.; Roldan, E.R.S. Sperm Bauplan and Function and Underlying Processes of Sperm Formation and Selection. *Physiol. Rev.* **2022**, *102*, 7–60, doi:10.1152/physrev.00009.2020.
32. Dumont, J.N.; Brummet, A.R. The Vitelline Envelope, Chorion, and Micropyle of *Fundulus heteroclitus* Eggs. *Gamete Res.* **1980**, *3*, 25–44, doi:10.1002/mrd.1120030105.
33. Selman, K.; Wallace, R.A. Gametogenesis in *Fundulus heteroclitus*. *Am. Zool.* **1986**, *26*, 173–192, doi:10.1093/icb/26.1.173.
34. Uribe, M.C.; Grier, H.J. Insemination, Intrafollicular Fertilization and Development of the Fertilization Plug during Gestation in *Heterandria formosa* (Poeciliidae). *J. Morphol.* **2018**, *279*, 970–980, doi:10.1002/jmor.20827.
35. Mattei, X.; Thiaw, O.T. Acrosome-like Structures in the Spermatozoa of Teleost Fishes. *Can. J. Zool.* **1993**, *71*, 883–888, doi:10.1139/z93-115.
36. Kobayashi, H.; Iwamatsu, T. Fine Structure of the Storage Micropocket of Spermatozoa in the Ovary of the Guppy *Poecilia reticulata*. *Zool. Sci.* **2002**, *19*, 545–555, doi:10.2108/zsj.19.545.

37. Jespersen, S. Fine Structure of the Spermatozoon of the Australian Lungfish *Neoceratodus forsteri* (Krefft). *J. Ultrastruct. Res.* **1971**, *37*, 178–185, doi:10.1016/S0022-5320(71)80049-7.
38. Mattei, X.; Siau, Y.; Seret, B. Etude ultrastructurale du spermatozoïde du coelacanthe: *Latimeria chalumnae*. *J. Ultrastruct. Mol. Struct. Res.* **1988**, *101*, 243–251, doi:10.1016/0889-1605(88)90015-8.
39. Ueda, Y.; Yoshizaki, N.; Iwao, Y. Acrosome Reaction in Sperm of the Frog, *Xenopus laevis*: Its Detection and Induction by Oviductal Pars Recta Secretion. *Dev. Biol.* **2002**, *243*, 55–64, doi:10.1006/dbio.2001.0541.
40. Scheltinga, D.M.; Jamieson, B.G.M.; Espinoza, R.E.; Orrell, K.S. Descriptions of the Mature Spermatozoa of the lizards *Crotaphytus bicinctores*, *Gambelia wislizenii* (Crotaphytidae), and *Anolis carolinensis* (Polychrotidae) (Reptilia, Squamata, Iguania). *J. Morphol.* **2001**, *247*, 160–171, doi:10.1002/1097-4687(200102)247:2<160::AID-JMOR1010>3.0.CO;2-R.
41. Okamura, F.; Nishiyama, H. The Passage of Spermatozoa through the Vitelline Membrane in the Domestic Fowl, *Gallus gallus*. *Cell Tissue Res.* **1978**, *188, 497–508, doi:10.1007/BF00219787.*
42. Bleil, J.D.; Wassarman, P.M. Sperm-Egg Interactions in the Mouse: Sequence of Events and Induction of the Acrosome Reaction by a Zona Pellucida Glycoprotein. *Dev. Biol.* **1983**, *95*, 317–324, doi:10.1016/0012-1606(83)90032-5.
43. Sathananthan, A.A.H.; Trounson, A.O.; Wood, C.; Leeton, J.F. Ultrastructural Observations on the Penetration of Human Sperm into the Zona Pellucida of the Human Egg in vitro. *J. Androl.* **1982**, *3*, 356–364, doi:10.1002/j.1939-4640.1982.tb00702.x.
44. Takahashi, C.; Okabe, E.; Nono, M.; Kishimoto, S.; Matsui, H.; Ishitani, T.; Yamamoto, T.; Uno, M.; Nishida, E. Single Housing of Juveniles Accelerates Early-Stage Growth but Extends Adult Lifespan in African Turquoise Killifish. *Aging* 2024, *16*, 12443–12472, doi:10.18632/aging.206111.
45. Wang, X.; Zhao, N.; Wang, T.; Du, S.; Liu, Q.; Li, J. Transcriptome Analysis Provides Insights into Copulation, Fertilization, and Gestation in *Sebastes schlegelii*. *Genes* 2022, *13*, 1812, doi:10.3390/genes13101812.
46. Zhou, H.; Sun, Y.; Li, X.; Zhou, Z.; Ma, K.; Guo, W.; Liang, Y.; Xie, X.; Zhang, J.; Wang, Q.; et al. A Transcriptomic Analysis of Gonads from the Low-Temperature-Induced Masculinization of *Takifugu rubripes*. *Animals* 2021, *11*, 3419, doi:10.3390/ani11123419.
47. Ruijter, J.M.; Ramakers, C.; Hoogaars, W.M.H.; Karlen, Y.; Bakker, O.; van den Hoff, M.J.B.; Moorman, A.F.M. Amplification Efficiency: Linking Baseline and Bias in the Analysis of Quantitative PCR Data. *Nucleic Acids Res.* 2009, *37*, e45, doi:10.1093/nar/gkp045.
48. Bustin, S.A.; Ruijter, J.M.; van den Hoff, M.J.B.; Kubista, M.; Pfaffl, M.W.; Shipley, G.L.; Tran, N.; Rödiger, S.; Untergasser, A.; Mueller, R.; et al. MIQE 2.0: Revision of the Minimum Information for Publication of Quantitative Real-Time PCR Experiments Guidelines. *Clin. Chem.* 2025, *71*, 634–651, doi:10.1093/clinchem/hvaf043.
49. Singh, P.P.; Isambert, H. OHNOLOGS v2: A Comprehensive Resource for the Genes Retained from Whole Genome Duplication in Vertebrates. *Nucleic Acids Res.* 2020, *48*, D724–D730, doi:10.1093/nar/gkz909.
